# Conformational Analysis of 1,3-Difluorinated Alkanes

**DOI:** 10.1021/acs.joc.4c00670

**Published:** 2024-05-31

**Authors:** William G. Poole, Florent Peron, Stephen J. Fox, Neil Wells, Chris-Kriton Skylaris, Jonathan W. Essex, Ilya Kuprov, Bruno Linclau

**Affiliations:** † School of Chemistry, University of Southampton, Highfield, Southampton SO17 1BJ, U.K.; ‡ Department of Organic and Macromolecular Chemistry, Ghent University, Krijgslaan 281-S4, 9000 Ghent, Belgium

## Abstract

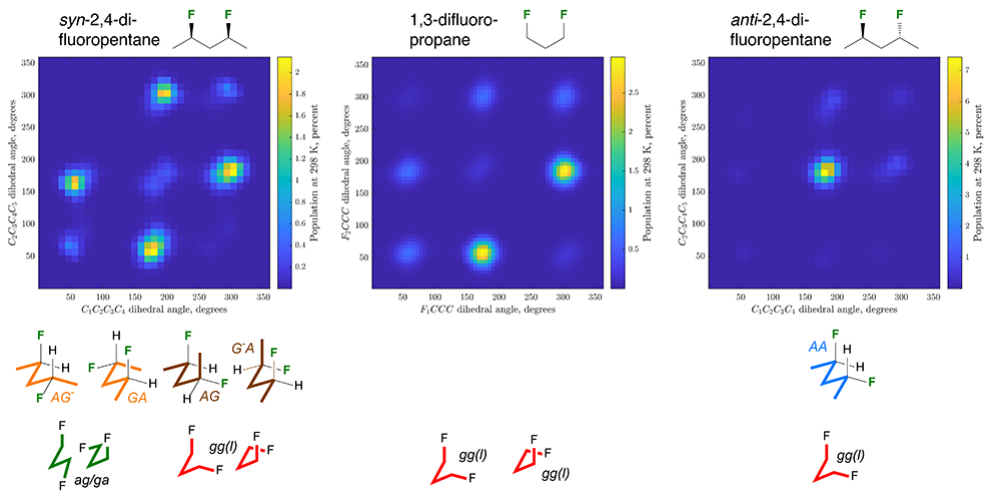

Fluorine substitution can have a profound impact on molecular conformation. Here, we present a detailed conformational analysis of how the 1,3-difluoropropylene motif (–CHF–CH_2_–CHF–) determines the conformational profiles of 1,3-difluoropropane, *anti*- and *syn*-2,4-difluoropentane, and *anti*- and *syn*-3,5-difluoroheptane. It is shown that the 1,3-difluoropropylene motif strongly influences alkane chain conformation, with a significant dependence on the polarity of the medium. The conformational effect of 1,3-fluorination is magnified upon chain extension, which contrasts with vicinal difluorination. Experimental evidence was obtained from NMR analysis, where polynomial complexity scaling simulation algorithms were necessary to enable *J*-coupling extraction from the strong second-order spectra, particularly for the large 16-spin systems of the difluorinated heptanes. These results improve our understanding of the conformational control toolkit for aliphatic chains, yield simple rules for conformation population analysis, and demonstrate quantum mechanical time-domain NMR simulations for liquid state systems with large numbers of strongly coupled spins.

## Introduction

Molecular conformation is closely linked to properties and function of bioactive compounds,^1^ catalysts,^2^ and organic materials such as liquid crystals.^3^ Consequently, conformational control is important for molecular property optimization. Conformational control along C–C single bonds has proven to be very effective when fluorine is introduced close to a polar functional group; this has attracted much interest.^4^ Conformational control of aliphatic chains and rings solely by introducing C–F bonds, for example, by vicinal and geminal difluorination,^5^ as well as by multivicinal polyfluorination,^6^ has also been demonstrated.^7^

**Figure 1:**
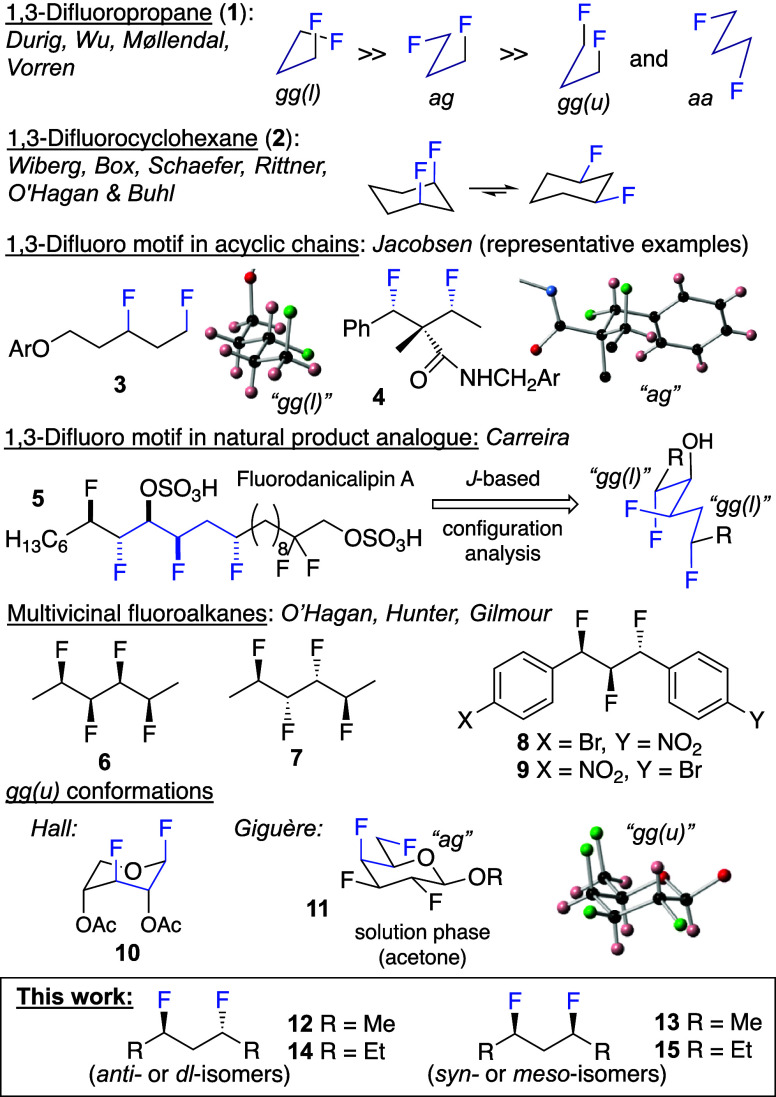
Summary of prior art on the 1,3-difluoropropylene motif. In conformation indices, *l* refers to *like* (both dihedrals have the same sign) and *u* refers to *unlike* (different signs).

The 1,3-difluoropropylene motif (–CHF–CR_2_–CHF–) has so far been studied mainly in its simplest representative, 1,3-difluoropropane **1** (Figure 1), in which the *gg(l)* conformation dominates, followed by the *ag* conformation.^8^ Notably, the *gg*(*u*) conformation with two parallel C−F bonds was calculated to have virtually no population, which has been explained by F···F repulsion. By the same reasoning, in the more constrained *cis*-1,3-difluorocyclohexane **2**, the diequatorial chair conformation was calculated to be the most populated in vacuum.^9^ Crystal structures of larger 1,3-propylene-containing compounds (e.g., **3** and **4**) show the C–F groups in *gg(l)* and *ag* conformations.^10^

For fluorodanicalipin A **5**, a fluorinated analogue of the naturally occurring chlorinated danicalipin A, *J*-coupling analysis^11^ indicated that the ground-state conformation of the corresponding alcohol in chloroform contains two *gg(l)* orientations (Figure 1) that were also present in danicalipin and the hexabrominated analogue.^12^ The Giguère group showed that the 1,3-difluoropropylene motif in 2,3,4-trideoxy-2,4-difluoroallitol displays an *ag* conformation (not shown).^13^ In the multivicinal alkane **6**, which also features a 1,3-difluoro motif, the stereochemistry dictates a bent, and for **7**, a linear zigzag chain, which was explained by the avoidance of 1,3-F···F (parallel C–F bonds) and of 1,3-F···CH_3_ interactions as the primary factors, and by the *gauche* effect (σ_C–H_ → σ*_C–F_ stabilization) as the secondary factors.^6a,6b,14^ Multivicinal fluoroalkanes containing up to six fluorine atoms have been investigated,^6b^ but most applications so far feature the 1,2,3-trifluoro-1,3-propylene group (–CHF–CHF–CHF–), *e.g*., in liquid crystals,^14a^ δ-amino acids as dipeptide mimics,^15^ and 1,3-diphenylpropanes such as **8** and **9** as 2-benzyl dihydrobenzofuran mimics.^16^ Interestingly, the conformational profiles of **8** and **9** are very different, illustrating the subtle influence of substituents.

Structures containing a 1,3-difluoro *gg(u)* motif have also been reported. For example, in solution (CDCl_3_/CFCl_3_), xylose derivative **10** existed almost exclusively in the ^1^*C*_4_ conformation with a *gg(u)* 1,3-difluoro conformation due to the anomeric effect.^17^ Crystal forces can also lead to 1,3-difluoro *gg(u)* conformations;^18^ an example is the tetrafluorinated galactose derivative **11**. Interestingly, in solution (acetone), it was proposed that its major conformer is *ag*.^18b^

Despite the interest in the conformational consequences of 1,3-difluoro motif, to the best of our knowledge, there are no reports describing a detailed conformational analysis of the 1,3-difluoropropylene (–CHF–CH_2_–CHF–) motif embedded in longer alkyl chains, *e.g*., as in **12**–**15**, although Weigert predicted, using molecular mechanics, a nonlinear conformation for **13**, again attributed to the repulsive 1,3-F···F Coulomb interaction.^19^ Even for 1,3-difluoropropane, only gas-phase calculations have been reported.

Here, we report a detailed investigation into the conformational profile of the 1,3-difluoropropylene motif as it appears in **1** and in the chain-extended **12**–**15**, in the gas phase as well as in polar solvents. Symmetric substrates were selected to simplify conformational analysis.

A key requirement in such studies is the ability to combine electronic structure theory data with experimental NMR data. The latter is useful because the Karplus equation^20^ connects NMR *J*-couplings to the dihedral angles. Hence, following the computational analysis of **1, 12**–**15** in different media, **12**–**15** were chemically synthesized to allow experimental determination of *J*-couplings.

A particular problem associated with symmetric molecules is that non-first-order NMR spectra require quantum mechanical simulations to extract *J*-coupling values. For small spin systems such as **6** and **7**, this is straightforward, but for molecules with more than 14 spins, such as in **14/15**, conventional NMR simulations would be feats of massive algebraic complexity because spin Hamiltonian matrix dimensions scale exponentially with the number of spins in the system, and matrices must be factorized during conventional simulations.^21^ The number of floating-point multiplications required for a factorization is the cube of the dimension; the total cost of the simulation is therefore of the order of 2^3*n*^, where *n* is the number of spins. For *n* = 14, *O*(2^42^) multiplications are required, which take about a week on a modern CPU, which is manageable. For *n* = 16, however, *O*(2^48^) multiplications would take months. Combined with the need to run hundreds of simulation instances in the iterative least-squares spectral fitting procedure, even with sparse time-domain techniques,^22^ this puts the estimated calculation time in the region of years. This is a formidable problem: with conventional algorithms, an iterative NMR fitting procedure is not practically feasible for a 16-spin system, regardless of the quality of the initial guess values for the *J*-couplings and chemical shifts.

Hence, to enable extraction of *J*-couplings from the NMR spectra of **14** and **15**, the recent polynomial complexity scaling spin dynamics simulation algorithms^23^ based on the restricted state space approximation^24^ were used to enable NMR data fitting for systems of hitherto unprecedented size. We also comment on fundamental caveats regarding matching NMR data with calculated populations of minimum energy conformers. The parameters thus extracted from the experimental data were consistent with electronic structure theory predictions.

## Materials and Methods

### NMR Experiments

^1^H and ^19^F NMR spectra with and without decoupling were collected after drying the solutions with activated molecular sieves. NMR data were collected on a Bruker AVIII HD 500 MHz NMR spectrometer. The magnet was reshimmed for each sample until the full width at half-height for the residual CDCl_3_ solvent signal was 0.5 Hz or better. ^1^H spectra were collected with 131,072 points in the time domain signal (zero-filled to 262,144 points) and the sweep width of 14 ppm around the center frequency of 5.0 ppm. ^19^F spectra were collected with 262,144 points in the time domain signal (zero-filled to 524,288 points) and the sweep width of 50 ppm around the position of the ^19^F signal. Adiabatic decoupling of ^1^H or ^19^F nuclei was applied as necessary.

### Conformational Analysis

DFT calculations were performed using M05-2X^25^ and M06^26^ exchange–correlation functionals with 6-311+G(d,p)^27^ and cc-pVTZ^28^ basis sets and SMD implicit solvation model (CHCl_3_, H_2_O).^29^ Room temperature Boltzmann populations of the different conformers for each of the fluorinated alkanes were computed using free energies obtained from the vibrational frequency calculations. The difference between M05-2X and M06 in conformer populations was less than 3% and in energies, less than 0.25 kcal/mol. All calculations were performed using *Gaussian 09*.^30^

An extensive catalogue of energy minima was built for all compounds by sampling every staggered conformation. With two dihedral angles, such as in **1**, **12**, **13**, and pentane, this gives 9 conformers; with four dihedrals, such as in **14**, **15**, and heptane, there are 81 conformers. Energy minimizations were attempted for all conformers, all unique energy minimum structures were analyzed.

### Uncertainty Analysis for DFT Conformer Populations

To estimate the confidence intervals for the conformer populations, we must perform uncertainty propagation for the Boltzmann distribution of conformation probabilities
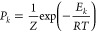
1where *Z* = ∑_*n*_ exp(−*E*_*n*_/*RT*) is the partition function. Treating all energies as relative to *E*_*k*_ and assuming that they have the same standard deviation σ_E_, we get
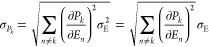
2The derivatives are easily calculated
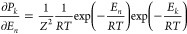
3for *n* ≠ *k*. Substitution into eq 2 and cosmetic simplification yields
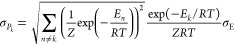
4Replacing all instances of eq 1 with the corresponding probabilities then yields
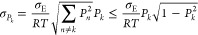
5where the upper bound is more convenient because the sum over other probabilities is not involved.

The estimation of σ_*E*_ is complicated by the fact that cancellation of errors is involved—the Boltzmann distribution operates on energy differences. Fluorine 1,3-disubstitution effects on steric energy being predominantly noncovalent and electrostatic, the absolute worst case estimate may be gleaned from the mean unsigned no-counterpoise error reported by Zhao and Truhlar^26^ in their Table 10 for the DI6/04 database of noncovalent dipole–dipole interaction complexes: σ_*E*_ = 1.1 kJ/mol. However, this figure does not account for error cancellation on subtraction—a more optimistic estimate may be obtained from comparing energy differences reported by dissimilar high-level methods, running a complete dihedral angle scan for 1,3-difluoropropane using M06/cc-pVTZ and MP2/cc-pVTZ methods in SMD chloroform and comparing the resulting energy differences results in σ_E_ = 0.42 kJ/mol. This figure was used to estimate the population uncertainties quoted in the main text.

## Results and Discussion

### Conformational Analysis of 1,3-Difluoropropane **1**

The conformational analysis of **1** was carried out at the M05-2*X*/6-311+G** level (Table 1, Chart S1), with the results in vacuum compared to Sun’s analysis^8b^ at the MP2/6-31G** level. Both analyses show that the conformational profile of **1** in vacuum is strongly biased toward the *gg(l)* conformations, with the *ga/ag* conformations present as minor components. There is a high destabilization for the *gg(u)* and *aa* conformations. These energy differences have been rationalized in terms of a combination of the number of stabilizing σ_C–H_ → σ*_C–F_ hyperconjugations and the conformer dipole moments.^8b^

**Table 1:**
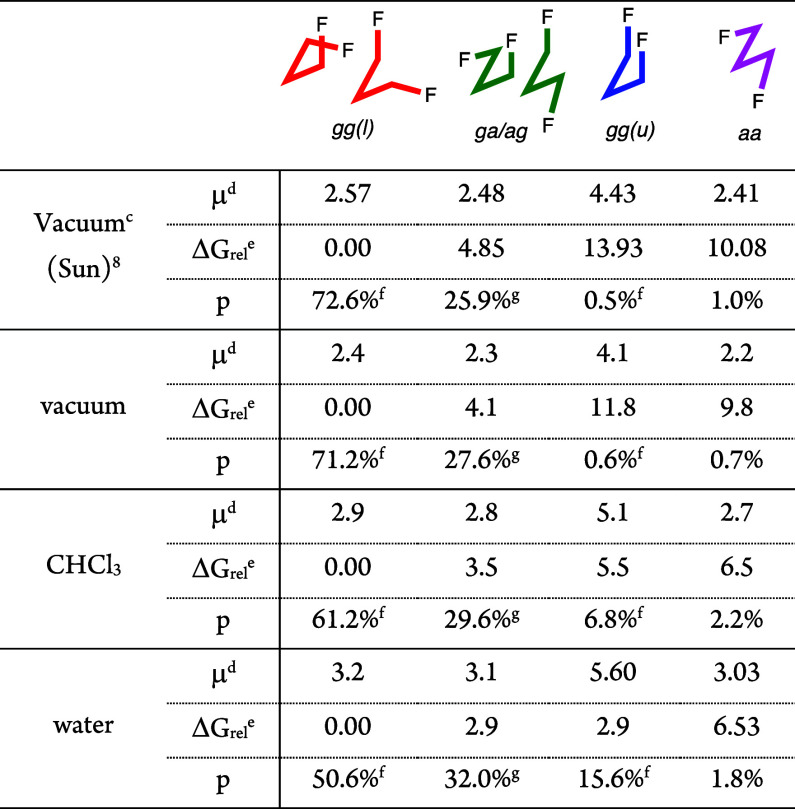
Conformational Profile of 1,3-Difluoropropane 1 (M05-2X in Combination with 6-311+G**)^a^,^b^

a Dihedral angles refer to rotation along the FC–CC bonds.

b “*l*” refers to “*like*”: the two dihedrals have the same sign. “*u*” refers to “*unlike*”.

c MP2 in combination with 6-31G**.^11^

d In Debye.

e In kJ/mol.

f Sum of 2 degenerate conformer populations.

g Sum of 4 degenerate conformer populations.

Next, conformational analysis was carried out using continuum solvation models. The *gg(l)* conformation remains the minimum energy conformation with increasing solvent polarity, but the relative destabilization of the other conformers decreases. This is especially the case for the *gg(u)* conformation, which is attributed to a stabilization from the aligned C–F dipoles. Thus, the often quoted 12 kJ/mol destabilization of two parallel C–F bonds only applies to the gas phase; this destabilization is halved in chloroform and is only 3 kJ/mol in water. This leads to a significantly increased population of the *gg(u)* conformation in water, mainly at the expense of *gg(l)* conformers.

### Conformational Analysis of 2,4-Difluoropentanes **12**, **13**

The results of the conformational analysis for the pentanes are shown in Table 2. Only those conformations that have <2 *gauche*-butane interactions are shown, with the full data shown in Figures S1, S2, Table S1, and in summary Charts S2–S4. Dihedral angles now refer to rotation around the central CC–CC bonds, with conformational descriptors indicated in capitals to distinguish from the CC–CF dihedral angles used for 1,3-difluoropropane.

**Table 2:**
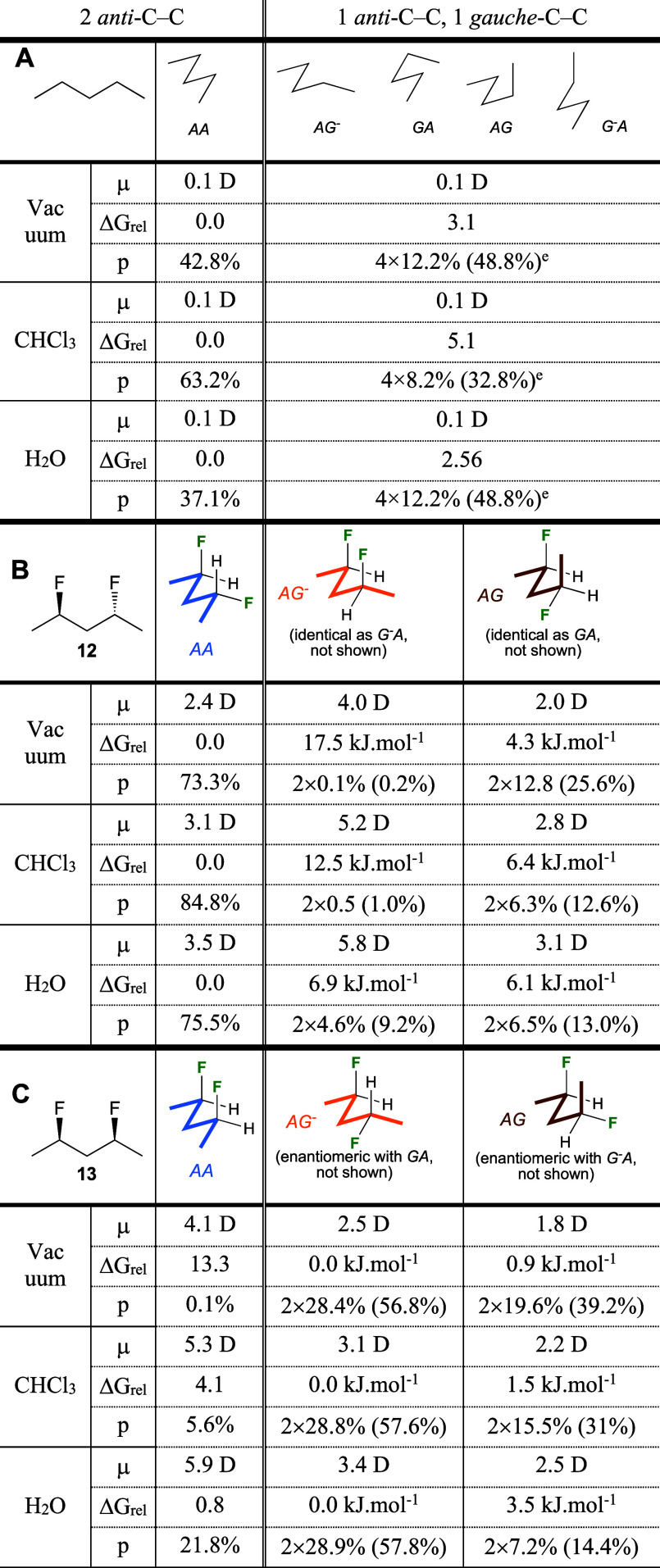
Conformational Profile of Pentane (A), 12 (B), and 13 (C) (M05-2*X*/6-311+G**)^a^,^b^

a Dihedral angles refer to rotation along the central CC–CC bonds.

b Only the most relevant conformers shown (full data: see Figures S1, S2, and Table S1).

It is instructive to compare fluorinated compounds with their parent hydrocarbon chains. For pentane (entry A), the *aa* conformer is the most stable, although with a population of only around 40% in vacuum and H_2_O, and 60% in CHCl_3_, with the rest of the population being conformers displaying one *gauche* dihedral angle. Conformations with two *gauche* dihedral angles are only populated for <7%, with those having a *syn*-pentane interaction not populated (Table S1).

The introduction of two 1,3-*anti*-configured fluorine atoms as in **12** (entry B) leads to a considerable stabilization of the linear *aa* conformer in vacuum. The introduction of this motif results in two distinct sets of degenerate conformers that feature one *gauche*-butane interaction: the *ag*^*–*^ and *g*^*–*^*a* conformations and the *ag*/*ga* conformations. The *ag*^*–*^/*g*^*–*^*a* conformations feature parallel C–F bonds, which result in a high dipole moment, and are not populated in vacuum. The *ag*/*ga* conformations then make up the rest of the population. Increasing the solvent polarity leads to a stabilization of the *ag*^*–*^/*g*^*–*^*a* conformations, which in water medium is high enough to result in an appreciable population (9.2%). In contrast, the *ag*/*ga* conformation undergoes destabilization with increasing solvent polarity.

With *syn*-difluoro substitution (**13**, entry C), the linear *aa* conformation displays parallel C–F bonds and is thus highly destabilized in vacuum. The conformers with one gauche butane interaction are now grouped into *ag*^–^/*ga* and *ag*/*g*^*–*^*a* pairs, with their degenerate constituents having an enantiomeric relationship. The *ag*^*–*^/*ga* pair is marginally the most stable pair in vacuum (ΔΔ*G*_rel_ = 0.9 kJ/mol), which increases upon increasing solvent polarity. Increasing the polarity of the medium also leads to a significant increase in the population of the polar *aa* conformation (from 0.1 to 22.6%), which is exclusively at the expense of that of the *ag*/*g*^*–*^*a* conformations, whose destabilization increases as the polarity of the medium increases.

### Conformational Analysis of the Heptanes

**Figure 2:**
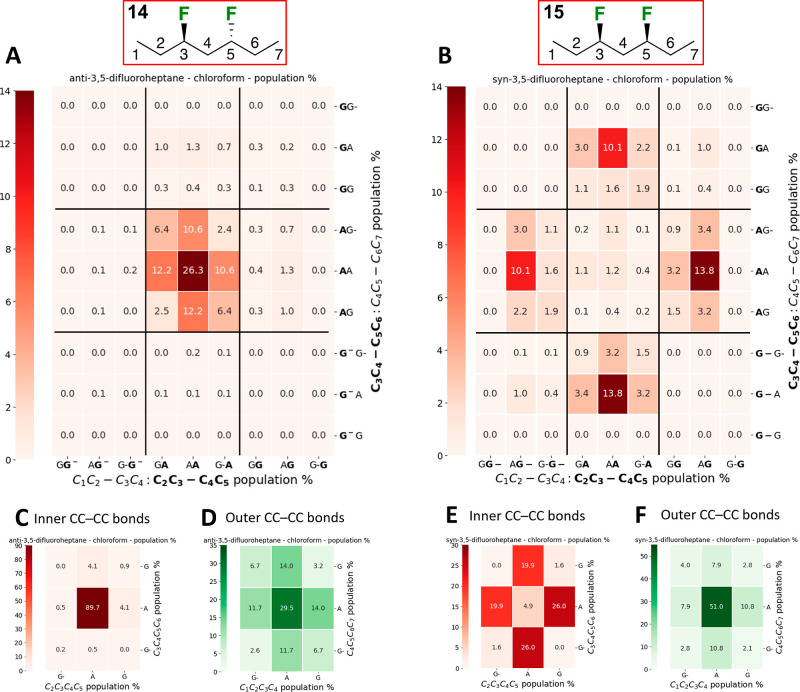
Full conformational profile of 3,5-difluoroheptanes **14** (A) and **15** (B) in chloroform. See Charts S5–S12 for complete data. The 9 × 9 grids represent conformations involving all internal C–C bonds; the inner CC–CC bond 3 × 3 grids (C,E) represent the conformations involving the central C–C bonds, summed over all 9 possible respective conformations of the respective outer internal C–C bonds and vice versa for the outer CC–CC bonds (D,F). See Figure S5 for details.

For the heptane substrates (Figure 2), there are four rotatable bonds to consider, with a set of “central” CC–CC bonds, and a set of “outer” CC–CC bonds (see color coding in Figure 2A). This leads to 81 possible conformers (Figures S3, S4), which can be pictured in 9 × 9 conformational grids as illustrated in Figure 2A,B for the populations in chloroform (see Charts S5–S7 for all populations, relative energies and dipoles in vacuum, chloroform, and water medium). The 9 × 9 grids can be condensed to two 3 × 3 grids (see Figure S5): In Figure 2C,E, each value in the grid represents the sum of the populations of the nine possible conformations for each defined conformation of the two central CC–CC bonds. Alternatively, in Figure 2D,F, each value represents the sum of the populations of the nine possible conformations for each defined conformation of the two outer CC–CC bonds. All 3 × 3 grids are shown as Charts S8–12.

Table 3 summarizes the 3 × 3 grids for the “central C–C bonds” of **14**,**15** in all media (Charts S8–S10). The same trends can be observed as for the pentane derivatives: the *anti*-difluorinated heptane **14** has the *aa* conformation of the central C–C bonds as the most populated conformation, while for the *syn*-difluorinated **15**, the *ag*^–^/*g*^*–*^*a* and *ag*/*ga* conformations are the most populated, with a strong increase of the population of the *aa*-conformation in water. The *syn*/*anti* population differences can be easily seen from the 3 × 3 grids in Figure 2C,E.

**Table 3:**
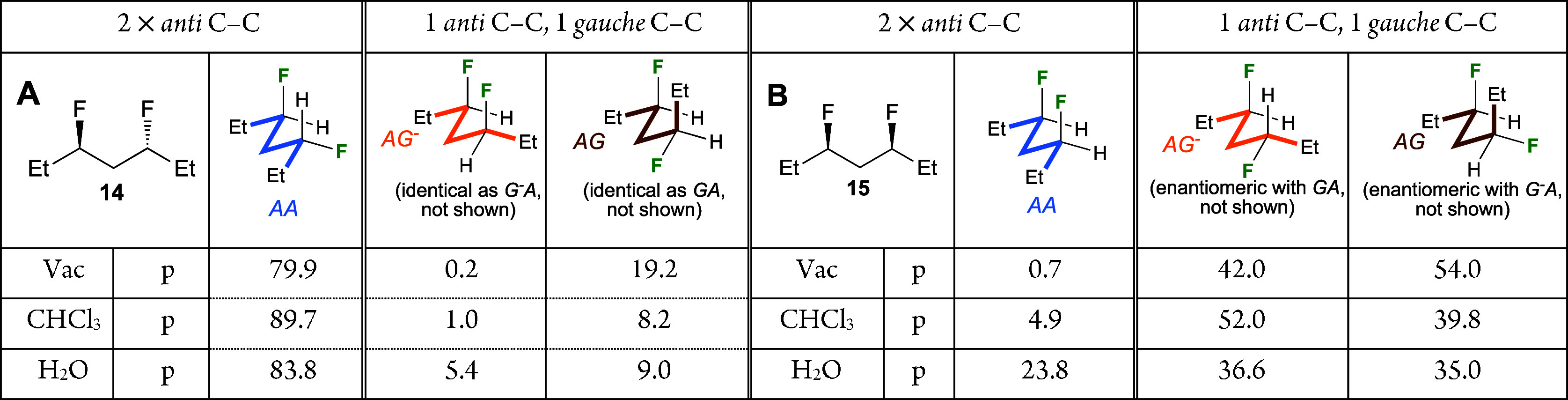
Conformational Profile of 14 (A), and 15 (B) (M05-2*X*/6-311+G**)^a^,^b^

a Dihedral angles refer to rotation along the central CC–CC bonds. Each value grid represents the sum of the populations of the nine possible conformations for each defined conformation of the two central CC–CC bond.

b Only the most relevant conformers shown (full data: see Figures S3, S4, and Table S2).

When the outer C–C bonds are included, for *anti*-3,5-difluoroheptane **14**, the 3 × 3 grid shown in Figure 2D indicates that almost 30% of all conformations have the two outer CC–CC bonds in the *antiperiplanar* conformation (in chloroform). This proportion is much larger in vacuum and water (44 and 37%, Charts S11, S12). The major individual conformers are displayed in Table 4. As expected, the conformational profile is heavily biased toward the presence of the *aa*-conformation is of the central C–C bonds, with the fully linear zigzag conformation present for a significant amount (26.3–32.0%, depending on the medium, Table 4a). This is significantly more than is calculated for the nonfluorinated heptane (6.0–15.5%, Chart S5). The conformation of **14** with central *ag*/*ga* dihedral angles, whose population in vacuum is enhanced (cf. Table 3), also features outer C–C bonds with antiperiplanar dihedral angles (*aaga*/*agaa* population of 6.3% each, not shown). Conformers with one of the outer bonds in the antiperiplanar disposition make up most of the rest of the total population (Table 4b,c), with the *g*^*–*^*aag/gaag*^*–*^ conformations (Table 4d) being the most stable conformers with two *gauche*-dihedral angles.

**Table 4:**
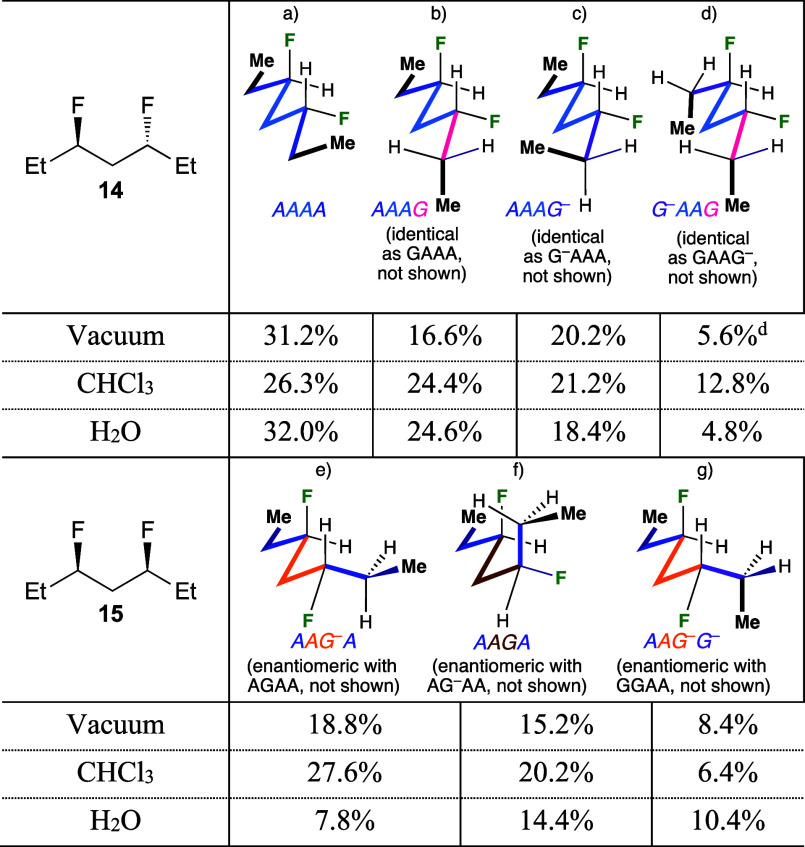
Conformational Populations of 11 and 15: Most Populated Conformations around the Outer C–C Bonds^a^

a M05-2*X*/6-311+G**.

b Dihedral angles refer to rotation along the central CC–CC bonds.

c Selection of major conformers showing all four dihedrals. Full data: Figures S3, S4, and Charts S6, S7.

d The *AAGA*/*AGAA* conformations of **14** in vacuum have populations of 6.3% each.

For *syn*-3,5-difluoroheptane **15**, the 3 × 3 grid shown in Figure 2F indicates that over 50% of all conformations have the two outer CC–CC bonds in the *antiperiplanar* conformation (in chloroform). This proportion is much smaller in vacuum and water (36 and 27%, Chart S12). The major individual conformations of **15** (Table 4) in vacuum and chloroform medium are clustered around structures with the central bonds having *ag*^*–*^/*ga* and *ag*/*g*^*–*^*a* dihedral angles. In each case, the conformations with both outer CC–CC bonds in the *antiperiplanar* disposition are the most populated (Table 4e,f), although the conformations with only one such outer C–C bond disposition are also populated, and this to a greater extent than in nonfluorinated heptane. Examples are the *aag*^*–*^*g*^*–*^/*ggaa* conformations (Table 4g). The *aag*^*–*^*g*/*g*^*–*^*gaa* conformations are not populated at all since these structures contain a *syn*-pentane disposition (not shown). The conformational profile of **15** in water is very different compared to heptane, with many more conformations having a similar population. Interestingly, the *aaga*/*ag*^*–*^*aa* and *aag*^*–*^*g*^*–*^/*ggaa* conformations now have the largest population (Table 4f,g). The larger dipole moment of the latter (3.5 D vs 2.4 D) will contribute to its enhanced stabilization in water.

Hence, these data show that *anti*-1,3-difluorosubstitution within an alkyl chain considerably stabilizes the linear zigzag conformation not only for the central C–C bonds but also for the outer C–C bonds. In contrast, *syn*-1,3-difluorosubstitution within an alkyl chain has the effect of reducing the difference between the stabilities of the various conformers.

### Correlation of Computational with Experimental Data

Next, these computational data were compared with experimental data. The synthesis of **12**–**15** is described first.

### Synthesis

**Scheme 1:**
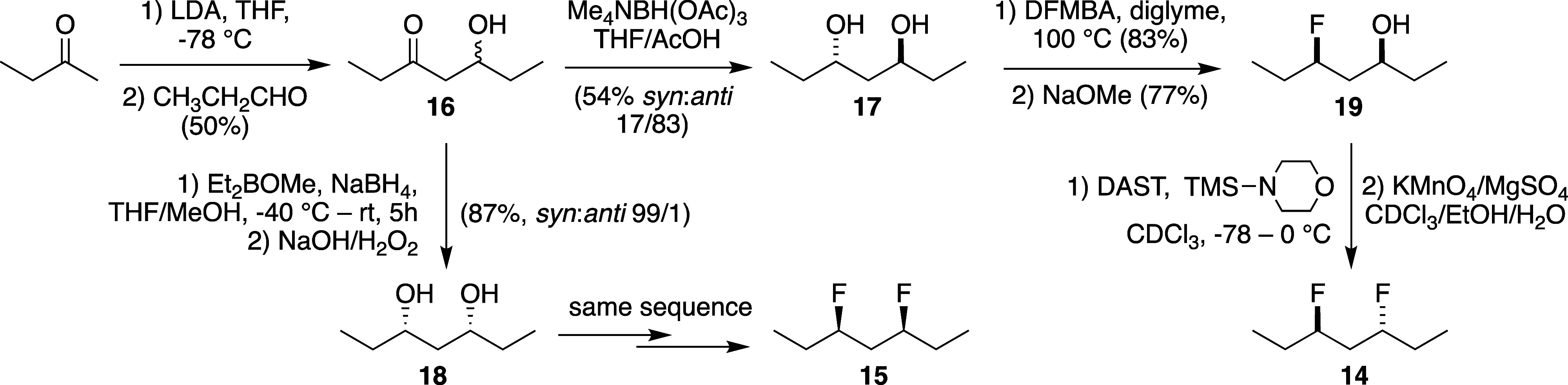
Synthesis of the 3,5-Difluoroheptanes

1,3-Difluoropropane **1** was commercially available and used without further purification. The synthesis of the *dl*- and *meso*-isomers of 2,4-difluoropentane and 3,5-difluoroheptane **12**–**15** relies on DFMBA-mediated fluorination^31^ of the corresponding 1,3-diol substrates and is illustrated in Scheme 1 for the heptanes. Regioselective deprotonation of 2-butanone followed by aldol reaction with propanal gave **16**.^32^*Anti*-selective reduction^33^ led to **17**, which was treated with DFMBA to give a *syn*-fluoroester, which, after transesterification, afforded *syn*-fluorohydrin **19**. Final fluorination was achieved with DAST and TMS-morpholine directly in CDCl_3_ as solvent due to the volatility of *anti*-3,5-difluoroheptane **14**. To remove alkene side-products, the crude reaction mixture was treated with permanganate. The *syn*-diastereomer **15** was obtained via the same sequence in similar yields from the *syn*-diol **18**, which in turn was obtained from **16** via *syn*-selective hydroxyketone reduction.^34^

### Experimental NMR Spectra

**Figure 3:**
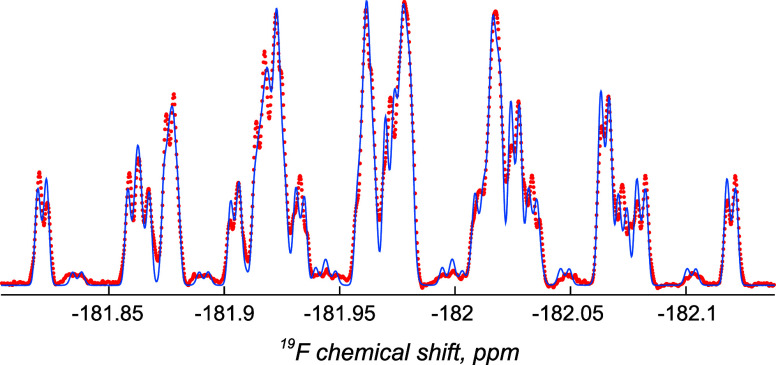
Experimental (red dots) and theoretical (blue lines) 471 MHz ^19^F NMR spectrum of meso-3,5-difluoroheptane **15** in CDCl_3_ clearly showing the lack of symmetry in the signal. See Figures S19–S22 for all ^1^H and ^19^F NMR fitting spectra.

Because the two fluorine nuclei have identical chemical shifts, but different *J*-coupling neighbors, the ^19^F NMR multiplets (Figures S10a–S13a) are the result of complicated interference between multiple homo- and heteronuclear *J*-couplings. Notable features of this complexity, indicative of a highly nontrivial spin energy level structure, are the presence of multiple nearly forbidden transitions (low intensity peaks), and the lack of reflection symmetry in the ^19^F signal, illustrated by the ^19^F NMR spectrum of **15** in Figure 3 (red dots), and likewise for protons. This is a known effect, arising when multiple Larmor frequency differences are much smaller than *J*-couplings; it is also known to require quantum mechanical simulation to extract the *J*-couplings.^35^

### Extraction of Chemical Shifts and *J*-Couplings

**Figure 4:**
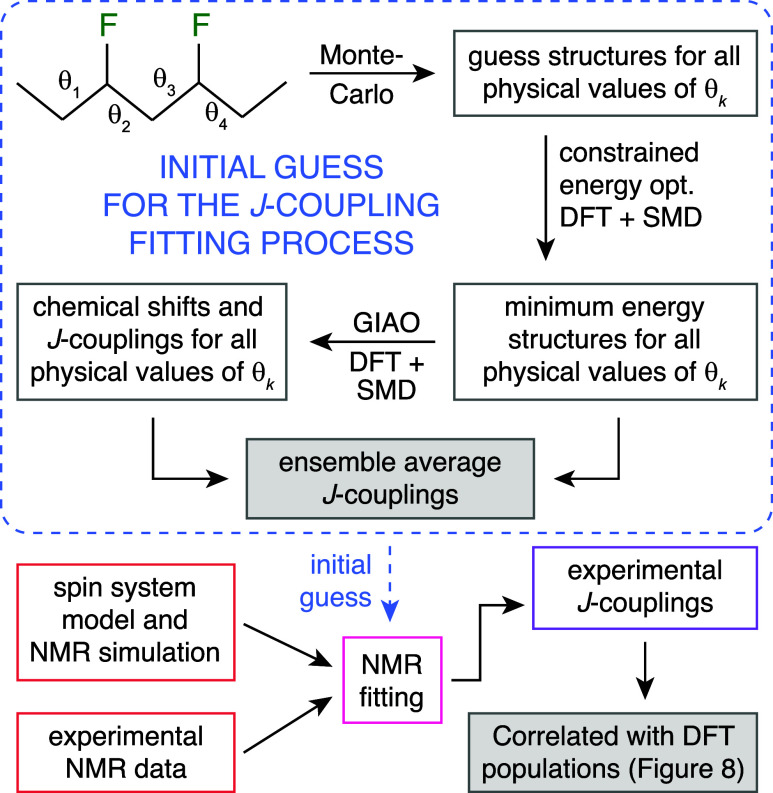
Workflow of the process to extract the experimental coupling constants from the spectra of the 3,5-difluoroheptanes. The ensemble average *J*-couplings and the experimental fitted *J*-couplings are given in Tables S4, S6, S8, S10, and S12.

The procedure used to extract chemical shifts and *J*-couplings from the complicated 3,5-difluoroheptane NMR spectra is summarized in Figure 4. Its central aspect is an elaborate initial guess that feeds into the least-squares fitting algorithm. This is necessary because error surfaces of NMR fitting problems contain many local minima.

### Initial Guess *J*-Coupling Calculation

The initial guess for *J*-coupling values in the spectral fitting procedure was obtained using the Monte Carlo averaging method: 10,000 molecular geometries with randomly selected sets of four dihedral angles were generated for each of the two 3,5-difluoroheptane isomers and screened for atomic clashes. Approximately 4000 geometries that have survived the screening were submitted for a constrained optimization, wherein all coordinates other than these internal coordinates were optimized into a minimum. The resulting set of minimum energy structures was submitted for *J*-coupling calculations using the GIAO DFT M06/cc-pVDZ method^26,36^ in SMD^29^ chloroform (basis fully uncontracted and augmented with tight functions^37^ at the Fermi contact coupling calculation stage).

The resulting set of energies and *J*-couplings was used in the Boltzmann averaging procedure that approximates, in the unbiased Monte Carlo sense, the four-dimensional integral over the dihedral angle space
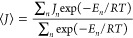
6where the sum is over the Monte Carlo instances, *J*_*n*_ is the particular *J*-coupling in the *n*-th instance, and *E*_*n*_ is the corresponding energy. For 1,3-difluororopropane **1** (which is sufficiently small), a systematic grid scan (Figure S18) followed by numerical integration was also performed to test the accuracy and convergence of the Monte Carlo procedure. The values thus obtained were used as a starting point in the NMR spectral fitting procedure discussed below, wherein the least-squares error functional was minimized numerically with respect to chemical shifts and *J*-couplings.

### Spin System Model and NMR Simulation

The large spin system simulation and fitting problem was handled using our recently developed polynomially scaling NMR simulation algorithms^24^ as implemented in *Spinach*.^38^ The simulations were carried out in Liouville space^39^—although its full dimension is very large (4^16^ ≈ 4.3 × 10^9^ for difluoroheptane), it is more amenable to truncation and screening than the corresponding 2^16^ = 65,536 dimensional Hilbert space.^24,40^ The following sequence of state space reduction stages is typical for the systems in question; 3,5-difluorofluoroheptanes are used as an example because smaller systems can still be handled with standard methods.

**Figure 5:**
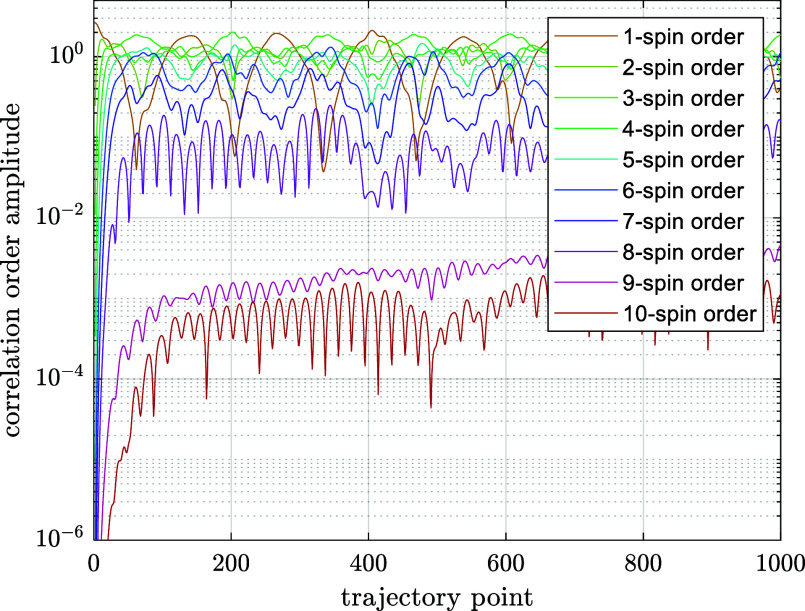
Contributions from different orders of spin correlation to the system trajectory in the pulse-acquire ^1^H NMR simulation of *anti*-3,5-difluoroheptane **14** (16 spins). Different curves correspond to the norms of the projection of the density matrix into the subspace of one-, two-, three-, etc. spin correlations.^42^ The two traces in the lower part of the figure correspond to nine- and ten-spin correlations—there are no detectable changes in the simulated spectrum when they are dropped: only correlations of up to eight spins need to be accounted for in this system.^24a^

1. Restricted state space approximation: as recently discussed by Kuprov et al.^24a,41^ very high orders of spin correlation and coherence remain unpopulated in liquid-state NMR experiments. Rigorous accuracy bounds on this assumption are available,^41^ but it may also be confirmed by direct inspection—Figure 5 shows the dynamics of the density matrix norm partitioned into contributions from subspaces with different orders of spin correlation. The amplitudes of states involving more than eight spins are 3 orders of magnitude smaller than the amplitude of states responsible for the transverse magnetization. The simulated spectrum shows no changes when they are dropped. The state space may therefore be restricted to only keep correlations of up to eight spins. This yields a reduction in the dimension of the Liouville space from 4.3 × 10^9^ to 1,564,672 for *dl*-3,5-difluoroheptane **14**.
2. Conservation law filter with respect to ^19^F nuclei: in high-field NMR spectroscopy, the quantum mechanical state of the spins that are connected to the rest of the system by **L**_Z_**S**_Z_ type Hamiltonian terms, and not pulsed directly, stays longitudinal.^40^ A longitudinal spin order filter was therefore applied in the ^19^F subspace (for proton NMR simulations), yielding a further reduction in the Liouville space dimension from 1,564,672 to 520,192. For ^19^F NMR simulations, this filter was applied in the proton subspace.
3. Conservation law filter with respect to ^1^H nuclei: in high-field NMR, the total projection quantum number of the spin system is conserved. A spin system that starts its evolution in the **L**_+_ state (at the beginning of the quadrature detection period) must remain in the *m*_Z_ = +1 subspace for the entire evolution period.^40^ Restricting the basis set to that subspace reduces the dimension further from 520,192 to 90,681. For ^19^F NMR simulations, this filter was applied with respect to fluorine projection quantum numbers.
4. Direct product symmetry factorization: the protons of the two rapidly rotating methyl groups obey an S_3_ permutation symmetry group *each*, meaning that the total system symmetry group is S_3_ × S_3_, with 36 symmetry operations and 9 irreducible representations of dimensions 1, 1, 2, 1, 1, 2, 2, 2, and 4. As we recently demonstrated,^40^ in Liouville space, only the fully symmetric irreducible representation is required. This reduces the state space dimension further from 90,681 to 58,473–a tiny fraction of the original dimension.
5. Diagonalization-free methods^43^ using Krylov propagation^43a,44^ with sparse matrix arithmetic:^22,45^ although the final Liouville space dimension (58,473) appears to be larger than the achievable Hilbert space dimension (16,384 when symmetry is taken into account), a Liouville space time propagation step (one matrix-vector multiplication, 58473^2^ ≈ 3.4 × 10^9^ flops ≈ 30 ms) is much cheaper than a Hilbert space time propagation step (two matrix–matrix multiplications, 2 × 16384^3^ ≈ 8.8 × 10^12^ flops ≈ 900 s). Given that 4096 propagation steps are required to obtain a spectrum with sufficient resolution, this difference is decisive and the improvement in simulation time from using a restricted symmetry-adapted Liouville space compared to the symmetry-adapted Hilbert space is by about 4 orders of magnitude. When sparse matrix arithmetic is used (the estimates above are given for dense matrices), the overall simulation runs in seconds. This solves the NMR simulation problem.

A documented open source implementation of the methods described above is available as a part of *Spinach* library.^23,38^ The simulations performed in this work are included in the example set.

### NMR Fitting: Experimental Chemical Shifts and *J*-Couplings

It was found that a single NMR spectrum, on either proton or fluorine, does not reliably constrain the large parameter set in question—a simultaneous fit to both the proton and the fluorine spectrum was in practice necessary. Similar procedures were carried out for all molecules reported in this paper. As discussed above, for the heptanes, we have used the *J*-coupling values obtained from GIAO DFT calculations using the M06/cc-pVDZ^26,36^ method in SMD^29^ chloroform.

The fitting of NMR spectra was performed by creating least-squares wrappers around *Spinach* simulations and feeding the least-squares error functional to the Nelder–Mead simplex^46^ minimizer supplied with *Matlab R2023b*. Fits were tested for stability by restarting several times from perturbed parameter combinations and for accuracy by simulating partially decoupled spectra, which were in complete agreement with the experimental spectra. Tight convergence tolerances (at least four decimal places for the chemical shifts and two decimal places for the *J*-couplings) are in practice needed because the system contains many near-zero energy differences that strongly affect the spectrum. An example of the fitting is shown in Figure 3 (blue lines); all fitting graphs may be found in the Supporting Information (Figures S19–22). The resulting sets of chemical shift and *J*-coupling values (“data fitted values”) are listed in Tables S3–S12. Because tiny deviations in *J*-couplings lead to nonmatching multiplets, the experimental ensemble-averaged *J*-couplings extracted by fitting the NMR spectra are accurate to ±0.1 Hz and may for our purposes here be considered exact.

As a control experiment, a simulation using the initial guess values predictably produced a completely dissimilar spectrum (Figures S23, S24), highlighting the necessity of the fitting procedure and the sensitivity of the spectrum to minor parameter variations. As a further control, ^19^F-decoupled ^1^H NMR spectra were simulated with the parameters fitted as described above and found to match the experimental spectra (Figures S25, S26).

### Correlation of the Experimental *J*-Couplings with the Calculated Populations

Finally, the calculated conformational populations discussed above could be correlated with the experimental coupling constants derived from our large-scale spin dynamics simulation fitting (Table 5). Such a correlation requires knowledge of the confidence intervals on the DFT populations, for which computed estimates were obtained as detailed in the Methods Section. It is not customary to provide error estimates for quantum chemistry calculations, but it is essential here: energy errors of even the best DFT methods relative to experimental databases can exceed *kT* by large factors. The associated uncertainties in the populations can therefore also be large.

**Table 5:**
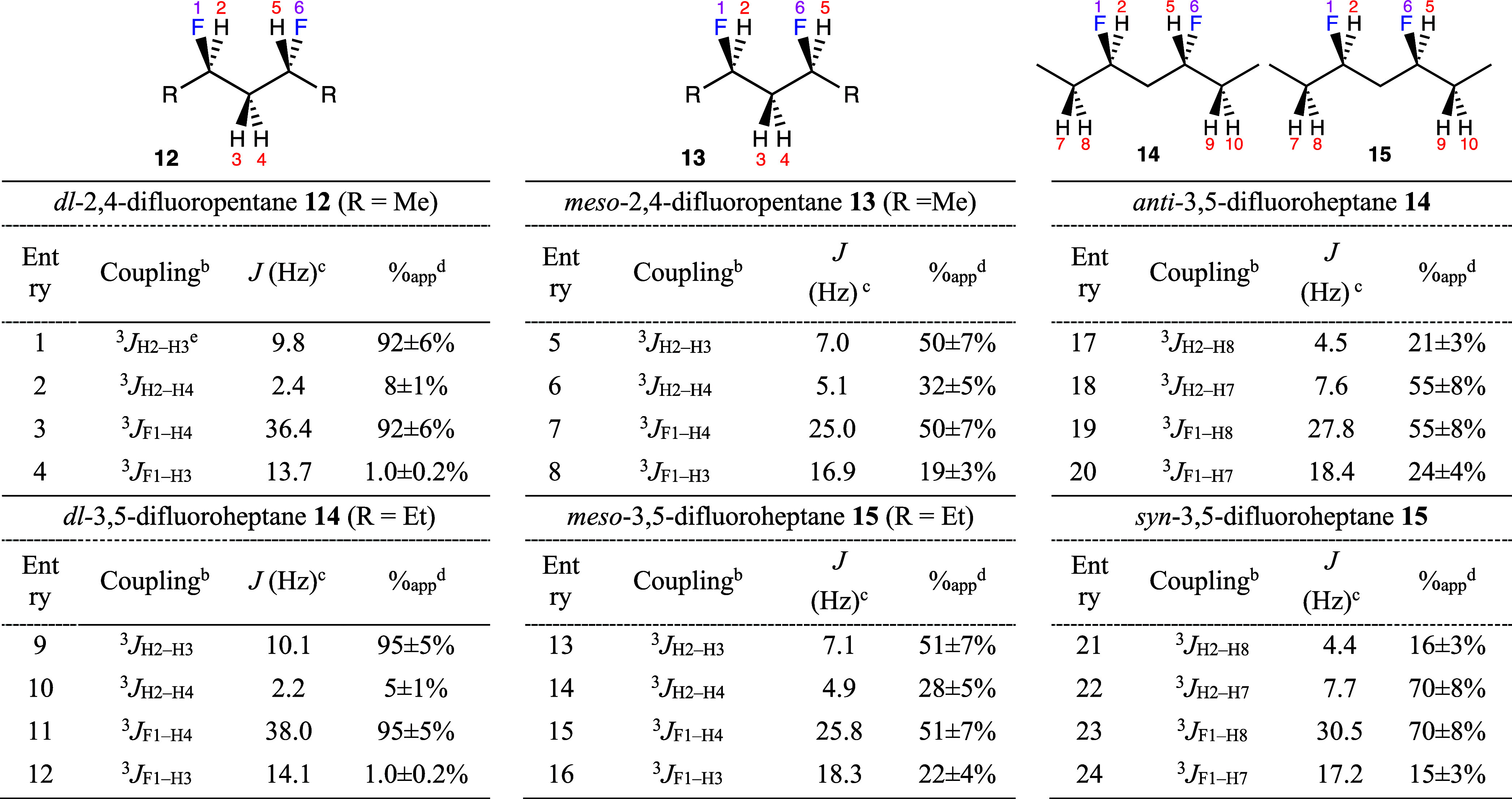
Coupling Constant Analysis for the Inner and Outer CC–CC Bonds (CDCl_3_, 298 K)^a^

a For all results, see Table S14 in the Supporting Information.

b Equivalent dihedral angles due to symmetry are indicated.

c “Data fitted values” obtained as described above. Accurate to ±0.1 Hz.

d Sum of the populations of the conformations featuring an antiperiplanar disposition of the atoms of the ^3^*J* in question. See Table 2/Figure S27.

e This coupling constant is equivalent to ^3^*J*_H4–H5_. All equivalent *J*-values are detailed in Table S14.

However, there are important caveats in correlating the population data with *J*-coupling analysis, which merit discussion. Each *J*-coupling is a probability-weighted integral over the *entire* potential energy surface, while the populations listed in Table 2 refer to the populations of the local energy minima. Hence, the match between the experimental *J*-couplings and the combined populations at the energy minima to obtain the percentage of *antiperiplanar* conformations, as given in Table 5, is necessarily approximate because the minima are shallow: at room temperature the rest of the multidimensional potential energy surface is also populated to some extent. However, systematically mapping the entire surface is clearly impractical.

**Figure 6:**
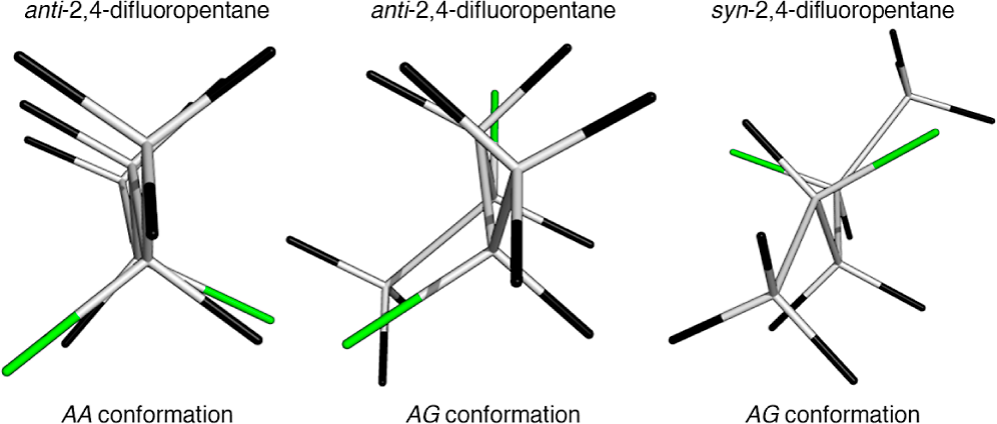
Energy minimum structures of high-population conformers of *syn*- and *anti*-2,4-difluoropentane, illustrating imperfect staggering.

In addition, not all minimum energy structures show perfect staggering. For example, the *aa* conformer of **15** features a colinear arrangement involving the C–F substituents. In the calculated minimum energy structure, the C–F bonds are not perfectly eclipsed (Figure 6), resulting in the antiperiplanar bonds having a dihedral angle of less than 180°.

**Figure 7:**
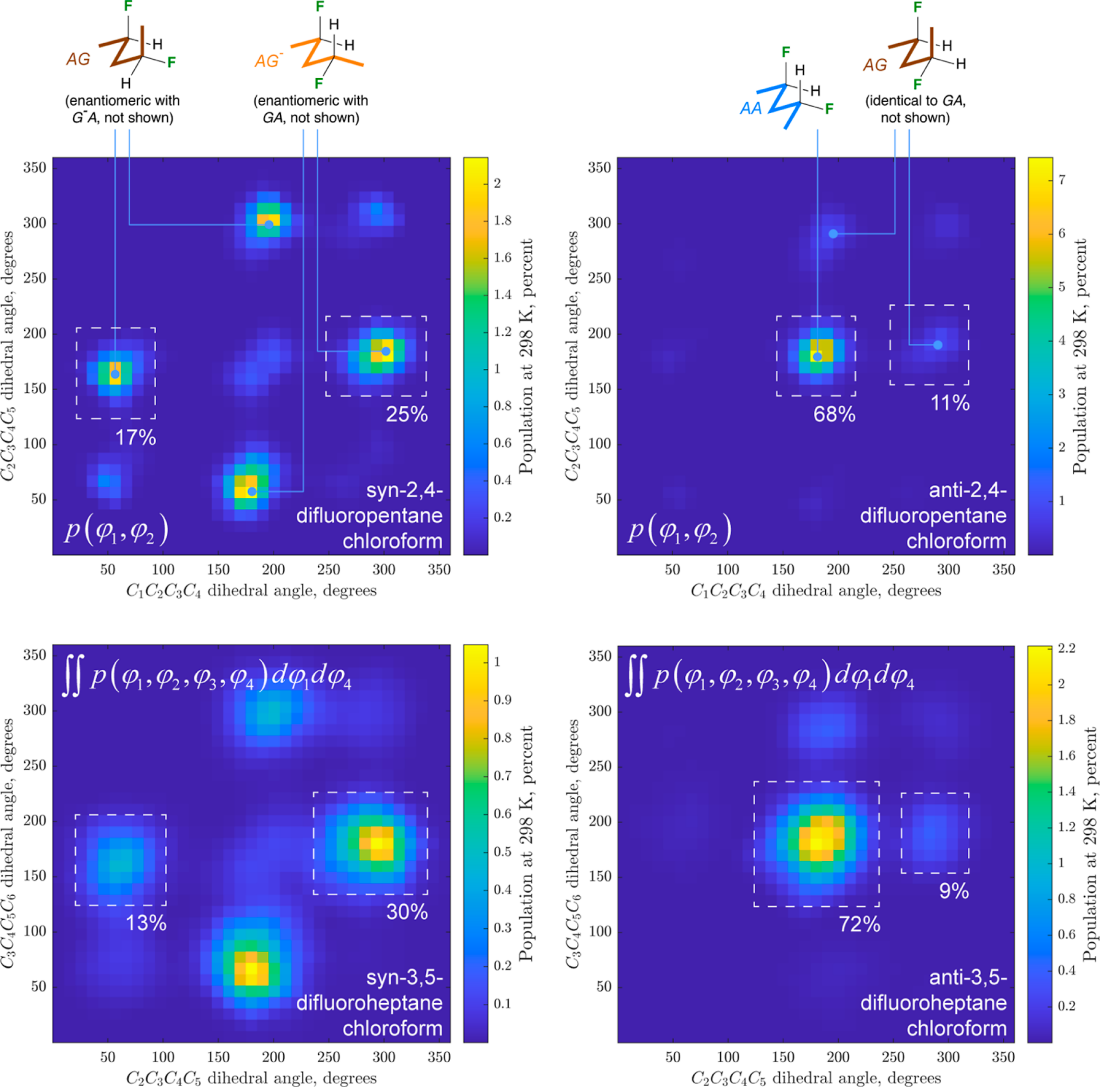
Boltzmann probability densities at 298 K obtained from a relaxed potential energy scan for *anti*-2,4-difluoropentane **12** (top right), *syn*-2,4-difluoropentane **13** (top left), *anti*-3,5-difluoroheptane **14** (bottom right), and *syn*-3,5-difluoroheptane **15** (bottom left). The calculations were performed using the DFT M06/cc-pVTZ method in SMD chloroform.

The deviation of the dihedral angle from ideal staggering was further investigated by a relaxed potential energy scan for the pentanes and heptanes (Figure 7). For the pentanes, a systematic search was performed by adjusting the central dihedral angles and allowing the rest of the molecule to relax. Each dihedral angle was scanned from 0° to 360° in 10° increments. The energies of each resulting conformation were then used to calculate populations. For the heptanes, owing to the large number of possible structures, a different approach was taken. Populations were calculated from the minimized energies of the 4000 structures, which were generated by Monte Carlo sampling as described above. The results were then plotted on a 4D grid and interpolated to cover the full 0–360 range for each dihedral angle. The 4D grid was then integrated over to reduce the plot to a 2D grid of just the inner two dihedrals. Hence, each data point represents the population within a 10-degree dihedral angle window. It can be clearly seen that, for each given conformation, there is a variation of dihedral angles, which affects the values of the averaged coupling constants. Given the significant amount of computing time, the relaxed potential energy scan was only performed in chloroform.

Integration of the population peaks indicated in Figure 7 provides the overall probability density of each of the major conformers. Despite the different calculation methodology, the populations of the relaxed potential energy scan agree well with the populations obtained based on minimum energy calculations (Table S13).

With these caveats in mind, the fitted *J*-couplings are correlated with the conformation populations obtained from the minimum energy calculations shown in Tables 2/3 (Tables S1/S2). To achieve a quantitative comparison avoiding the use of the nonlinear Karplus equation, the following methodology was adopted: a set of pertinent three-bond H–C–C–H and H–C–C–F systems is selected; then each H–C–C–H/F system is considered within each possible conformation, and the sum of the populations of those conformers for which it displays an antiperiplanar (app) disposition leads to a %_app_ value (see Figures S27–29 for details). These values are listed with their corresponding ^3^*J*-couplings in Table 5 for pertinent vicinal C–H/C–H/F bonds.

The larger the %_app_ value, the larger the expected *J*-coupling. This is the case across the board, despite the large uncertainty in the DFT populations. For example, when the central C–C bonds of the *anti*- and *syn*-stereoisomers are considered, the larger ^3^*J*_H2–H3_ value in **12** (9.8 Hz, Table 5, entry 1) compared to the equivalent coupling constant in **13** (7.0 Hz, entry 5), agrees with its higher percentage antiperiplanar orientation (92% vs 50%). Equally, the ^3^*J*_F1–H4_ value for **12** (entry 3) and the equivalent ^3^*J*_HF_ for **13** (entry 7) clearly are in accordance with the calculated fraction of antiperiplanar conformations: for **12**, the value is 36.4 Hz (95% antiperiplanar), while for **13**, this is 25.0 Hz (51% antiperiplanar), this time with nonoverlapping confidence intervals of the calculated populations. Satisfyingly, the same conclusion can be made for **14** and **15** when comparing the data in entries 9–12 and 13–16. In some cases, even subtle differences in calculated populations are consistent with experimental data, for example, the ^3^*J*_H2–H3_ and *J*_F1–H4_ values of **12** and **14** (entries 2,3,10,11). Exceptions include the ^3^*J*_H2–H7_ values of **14** and **15** (entries 18 and 22), which have very similar values despite the very different %_app_ values, although for the associated F1–C–C–H8 bonds, the ^3^*J*_F1–H8_ value are in accordance with the calculated populations.

**Figure 8:**
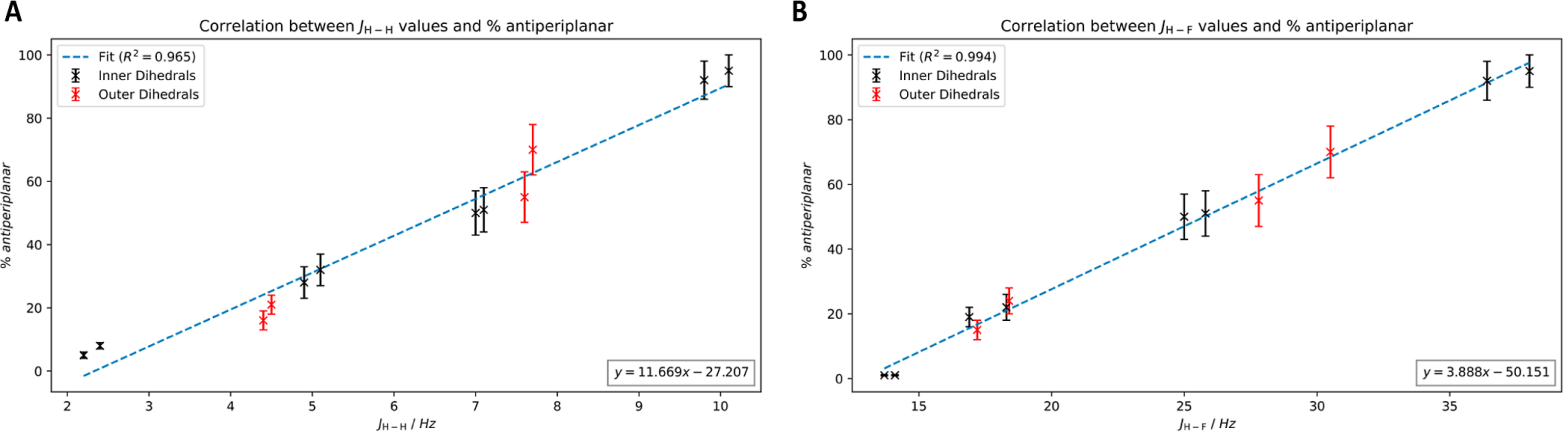
Correlation between the calculated percentage antiperiplanar conformation of a H–C–C–H (A) or H–C–C–C–F (B) unit and its corresponding experimental *J*-coupling constant, for all internal CC–CC bonds of **12–15**.

The observed *J*-coupling of the antiperiplanar conformation is a Boltzmann average over the local conformational energy minimum. Likewise, the population of the antiperiplanar conformation is an integral of the Boltzmann probability density. For the compounds investigated, both the energy and the Karplus curve are well approximated in the immediate vicinity of the 180-degree dihedral angles by a constant (energy minimum and also the minimum of the cosine wave in the Karplus relation). In this case, a strong correlation is expected between the *J*-coupling and the fraction antiperiplanar; this may occur in other substances fulfilling the same conditions on the energy surface and the Karplus curve. Indeed, experimentally, it was found that the data in Table 5 represent a linear relationship between the magnitude of a coupling constant and the amount of antiperiplanar disposition of the associated H–C–C–X (X = H,F) bonds, as shown in Figure 8, with—perhaps unexpectedly—high correlations for H–C–C–H and H–C–C–F systems (R^2^ = 0.9654 and 0.9940, respectively), which is a convincing indication of the accuracy of the calculated populations in SMD chloroform.

### Discussion of Conformation Populations

With the excellent correlation between the calculated and experimental data, differences in populations of various conformers between the *syn*-and *anti*-difluorinated substrates **12**–**15** can be discussed with confidence. Two aspects will be considered: the 1,3-difluoro-motif and the alkane conformation, including comparisons with the nonfluorinated precursors.

### Variation in 1,3-Difluoro Motif Disposition (Figure 9)

**Figure 9:**
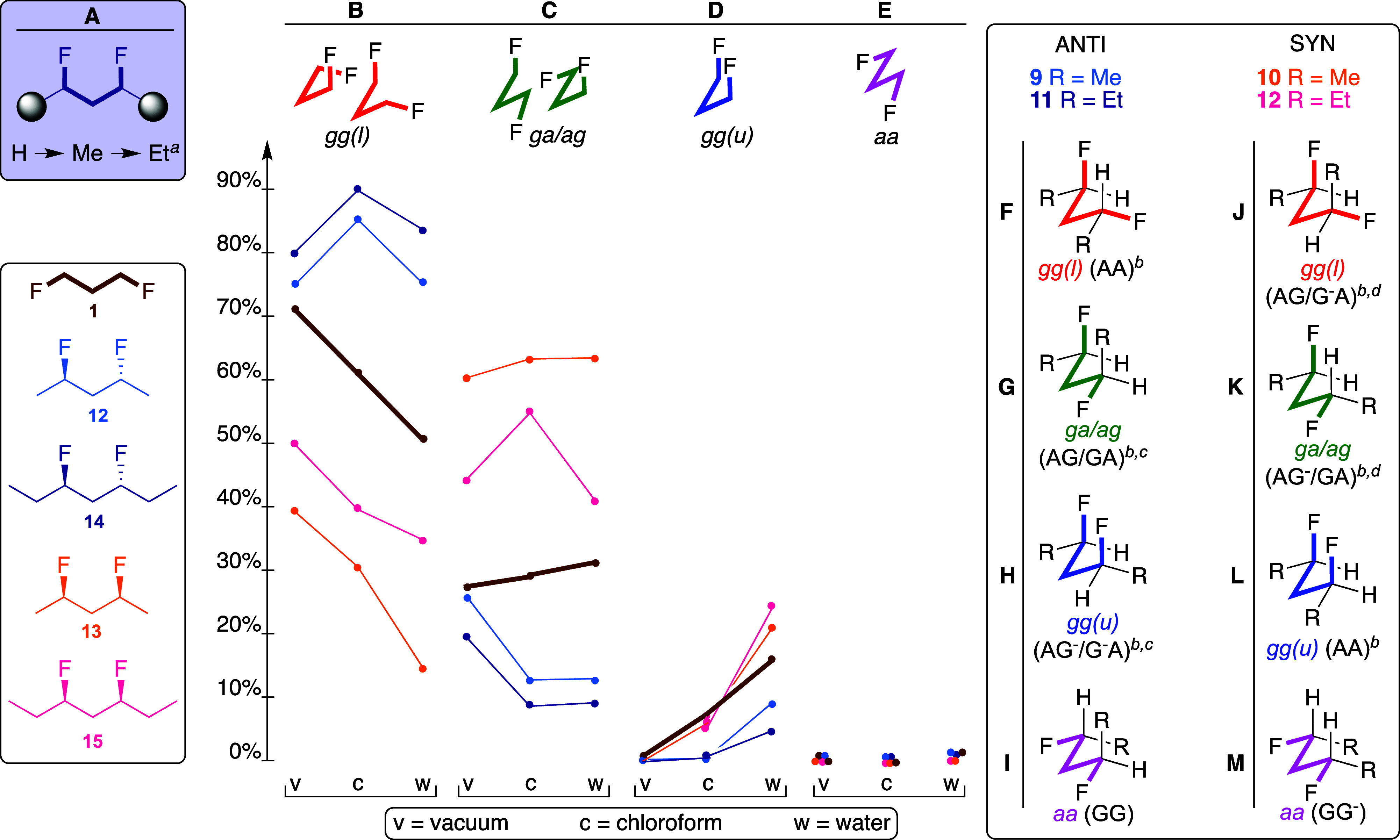
1,3-Difluoro motif conformational population changes according to the medium, for **1**, **12**–**15** (M05-2*X*/6-311+G**). The populations shown represent the sum of any degenerate structures. ^*a*^ Each population value of a given heptane conformation represents the sum of the populations of the nine possible conformations involving the outer C–C bonds. ^*b*^ Major conformation having this motif. ^*c*^ Identical conformers. ^*d*^ Enantiomeric conformers.

In this section, the discussion is focused on the relative disposition of the C–F bonds, when embedded in an alkyl chain. In earlier work, we have shown that the conformation of the vicinal difluorination motif in 1,2-difluoroethane, which is controlled by the fluorine *gauche*-effect, is very different from that of a vicinal difluorination motif when embedded in a butane chain, as steric effects become more important.^47^ Hence, it is of interest to establish how the conformation of the 1,3-difluoromotif in 1,3-difluoropropane is different from the conformations of 1,3-difluoromotifs embedded in larger alkyl chains (Figure 9A), especially with regard to the *gg(u)* conformations that feature parallel 1,3 C–F bonds.

The data in Figure 9 are grouped by C–F/C–F disposition, with color-coding as in Table 1. Each data point represents the sum of the populations of the conformers that feature this particular disposition, for each medium. To facilitate discussion, the major conformers of the pentanes/heptanes that contribute to the population of each possible disposition are shown in the right-hand box.

The populations of the 1,3-difluoro *gg(l)* conformation (Figure 9B) show a large variation according to chain length and relative stereochemistry. This is the most populated motif for 1,3-difluoropropane **1**, although its population decreases with increasing polarity of the medium. In 1,3-difluoropropane, this is the only conformation where two stabilizing σ_C–H_ → σ*_C–F_ interactions can take place involving different C–H bond donors. This conformation is even more populated when the motif is embedded in a longer alkyl chain with *anti*-stereochemistry. Clearly, this is due to the favorable situation arising from the alkyl chain being in the linear zigzag conformation (Figure 9F), with a larger stabilization when the chain is longer, while maintaining the favorable hyperconjugation situation. In contrast, for the *syn*-configured difluorides **13/15**, the populations of the *gg(l)* conformation are decreased, which can be attributed to steric hindrance between the R-group and the fluorine (Figure 9J). The abundance of the *ga/ag* conformation (Figure 9C) also significantly varies depending on chain length and relative stereochemistry. It is the second most populated conformation for 1,3-difluoropropane, and its populations are now increased for the *syn*-configured substrates and decreased for the *anti*-configured ones. This can be explained by comparing the respective major contributing conformations: for the *anti*-configured compounds, there is steric hindrance between the R-group and a fluorine atom (Figure 9G), while for the *syn*-configured substrates (Figure 9K), there is a classic *gauche*-butane type interaction between the R-group and a hydrogen. The pentanes always show a larger population than the heptanes, which agrees with the expected steric hindrance differences. Also, the relative order of the *ga/ag* populations mirrors exactly that of the *gg(l)* populations.

Interesting trends can be seen for the *gg(u)* conformation (Figure 9D). For all compounds involved, the population is almost zero in vacuum, with an increasing population when the polarity of the medium increases, due to the high dipole moment of this conformation. This increase is much larger for the *syn*-configured compounds **13**/**15**, which can be explained by the position of the R-groups in the most stable alkane zigzag conformation (Figure 9L), with a larger stabilization for the longer heptane. The *anti*-configured substrates **12**/**14** feature a classic *gauche*-butane interaction (Figure 9H), hence their smaller stabilization in polar media. The increased population of this motif in aqueous medium for the *syn*-configured compounds is significant. Their population percentage in water can be as high as 22% (for **13**). This is higher than for the corresponding 1,3-difluoropropane conformation in water (15%), and it is noteworthy that for **13**, the destabilization of this conformation in water is minimal (0.8 kJ/mol). CF··FC interactions featuring in the parallel 1,3-C–F disposition have attracted much interest. It was shown that these were not responsible for the helical conformation of perfluoroalkyl chains.^48^ Perfluoropropane has a perfectly staggered conformation (driven by a σ_C–C_ → σ*_C–F_ hyperconjugation).^48^ However, these systems contain CF_2_/CF_3_ groups, in which C–F bonds are less polarized. NCI analysis at the MP2/aug-cc-pVDZ level of all-*cis*-1,2,4,5-tetrafluorocyclohexane identified CF···FC interactions as being attractive.^9e^ However, our results do suggest that for conformationally flexible compounds, the destabilizing electrostatic repulsion component for this C–F disposition is more important, even in water. This is clearly illustrated in the conformational profile of **15**, where the *aa* conformation is only the third most abundant one, despite the presence of a full linear zigzag carbon chain.

The conformations with the *aa* 1,3-difluoromotif disposition (Figure 9E) have a very small population in all media, for all substrates involved.

### Variation in Alkane Conformation with Relative Stereochemistry and Chain Length

In this section, the discussion is focused on the alkane chain conformation. A major motivation for the incorporation of two fluorine atoms onto a flexible alkyl chain in a given molecule is to influence the conformation of the chain. For unsubstituted hydrocarbons like pentane and heptane, the *aa* conformation is the major conformer, but only with ∼40% population, the rest mostly being conformations with one *gauche*-butane interaction.

For the *anti*-isomers **12** and **14**, by far the most populated conformations feature the linear zigzag conformation (Figure S30). Clearly the introduction of two fluorines with *gg(l)* C–F disposition, which was also was found as most stable arrangement in 1,3-difluoropropane **1** (cf. Table 1), has a significant effect in the stabilization of the linear zigzag conformation. The alkane *gg(l)* C–F conformation is consistently more stabilized for the heptanes due to the presence of the larger ethyl substituent. All the other conformations have a very low population, except perhaps the *ag* conformation in vacuum, which is stabilized in this medium due to its low dipole moment.

**Figure 10:**
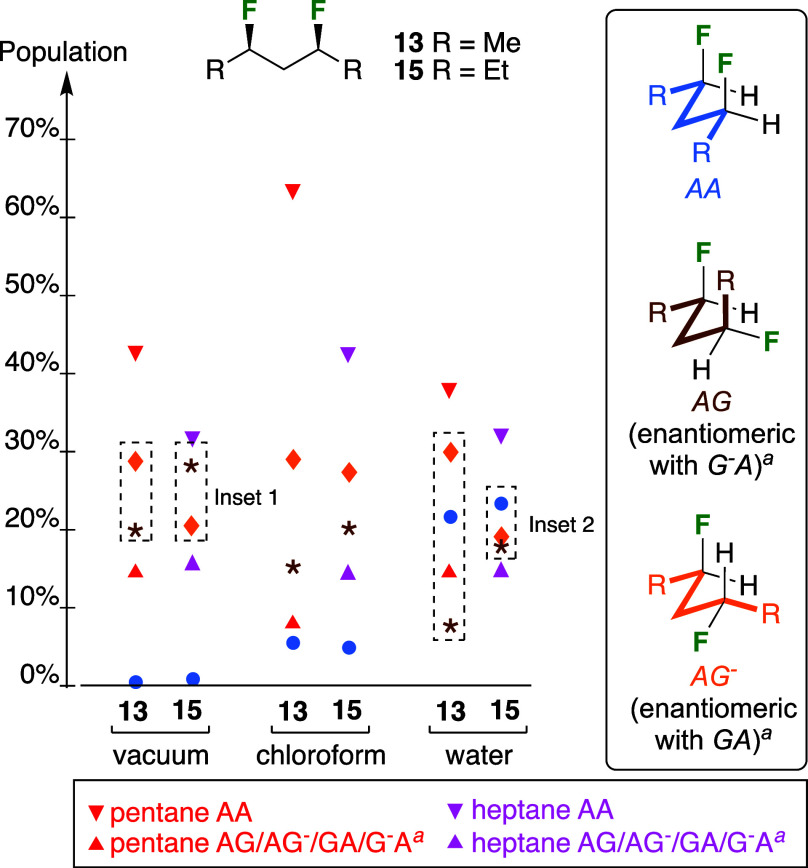
Hydrocarbon chain population changes according to the medium, for pentane, heptane, and the *syn*-substrates **13** and **15**. The data points represent the population of a single conformation, even for degenerate conformers. The data for the heptanes again refer to the central C–C bonds, with each such population representing the sum of the populations of all possible conformations involving the outer C–C bonds. Color coding is the same as in Table 2. ^*a*^The population shown refers to only one of the degenerate conformers.

**Figure 11:**
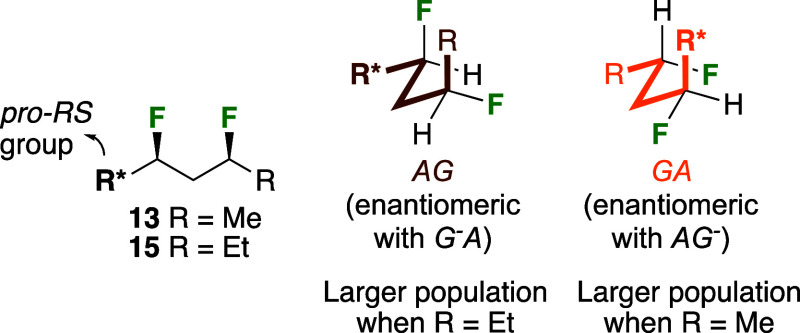
Comparison between the two possible conformers of **13**,**15** with one C–C–C–C *gauche*-interaction.

For the *syn*-isomers **13** and **15** (Figure 10), the conformational profiles are very different. The data-points represent the population of a single conformation, even for degenerate conformers: while the substrates discussed herein possess symmetry, this will generally not be the case for most applications. Hence, conformations that are degenerate for the pentanes/heptanes **13**, **15**, whether identical or enantiomeric, will be different for nonsymmetric 1,3-propylidene containing structures, and it is thus appropriate to compare populations of single conformers. In vacuum and chloroform medium, the four conformations displaying one *gauche*-butane interaction are the most populated ones, albeit not in the same order: In vacuum, the *ag*^–^/*ga* conformations of the pentane **13** are more populated than the *ag*/*g*^*–*^*a* conformations, while for the heptane **15** it is the other way round (inset 1). This is further illustrated in Figure 11. Due to the symmetry of the substrates, the *ag* and the *ga* conformations can be depicted as shown, with the carbon chain in the same orientation, revealing the difference in fluorine positions. The only apparent relevant difference between these conformations is that the R group displays a steric interaction with the F atom in the *ag* conformation, and with a H atom in the *ga* conformation, yet, in vacuum, the *ag* conformer is more populated for the heptane **15** compared to the pentane **13**. While the difference in their A-values (Me: 1.70; Et: 1.75) is small, there must be other small effects that lead to this population difference. In chloroform, the *ag*^–^/*ga* conformations are the most populated for both **13** and **15**.

The picture is very different in water (Figure 10). For the pentane **13**, the *ag*^–^/*ga* conformations are now much more populated than the *ag*/*g*^*–*^*a* conformations, with the *aa* conformation populated in between. There is a 3.5 kJ/mol energy difference between the *ag*^–^/*ga* and *ag*/*g*^*–*^*a* conformers. For the heptane **15**, the *ag*^–^/*ga* and *ag*/*g*^*–*^*a* conformers all have a very similar population, with the *aa* conformer now being the most populated. Furthermore, there is only a 0.6 kJ/mol energy difference between all the conformers, leading to a much more equal population between the conformers for the heptane chain compared to the pentane chain.

Hence, there is an overall picture that with *anti*-1,3-difluoro substitution, there is a very pronounced conformational preference of the central C–C bonds in all media (for the *aa* conformer), which is stronger when the 1,3-fluoropropylene motif is embedded in a longer chain. In contrast, introducing *syn*-1,3-difluorosubstitution leads to an increased flexibility with the different conformers having similar stabilities. This is, again, especially the case when the 1,3-fluoropropylene motif is embedded in a longer chain.

## Conclusions

A combined synthetic, NMR, and electronic structure theory approach is reported for a detailed conformational analysis of the 1,3-difluoropropylene (−CHF–CH_2_–CHF−) motif when embedded in linear aliphatic chains of increasing length, involving *J*-coupling analysis against DFT-calculated conformation populations. A polynomial complexity NMR simulation method^24a^ (as implemented in *Spinach*(43b) package) was used to enable the fitting of experimental *J*-couplings to strong second-order NMR spectra of large spin systems such as those in difluoroheptanes. Although the *J*-couplings obtained from DFT are useful as an initial guess, they do not reproduce the experimental NMR spectrum. Fitting was therefore necessary to extract the experimental values; fitting of non-first-order (meaning that a quantum mechanical simulation is unavoidable) NMR spectra on this scale (16 strongly coupled spins) has not previously been possible due to prohibitive computational complexity of the task.

The matching of experimental NMR *J*-couplings with the calculated conformation populations was achieved by correlating the *J*-couplings between antiperiplanar atoms (H–H and H–F) with the sum of the calculated populations of the conformations that feature those respective pairs in antiperiplanar arrangement. The very high correlation coefficients obtained (0.965 for H–C–C–H and 0.994 for H–C–C–F) serve as an independent confirmation of the accuracy of the conformation population calculations.

It is shown that the 1,3-parallel C–F orientation in 1,3-difluoropropane is much less destabilized in water (2.9 kJ/mol) compared to vacuum (11.8 kJ/mol), and when embedded in longer alkanes with relative *syn*-stereochemistry, the destabilization is reduced to 0.8 kJ/mol (pentane) and 1.6 kJ/mol (heptane). With *syn*-1,3-difluoro stereochemistry conformations with parallel C–F arrangement virtually unpopulated in vacuum, they become the most populated ones in *syn*-3,5-difluoroheptane in water (not taking degeneracy into account).

With regard to alkane conformation, introducing an 1,3-*anti*-difluoromotif strongly stabilizes the linear zigzag conformation, while with *syn*-1,3-difluorination, the energies of the possible conformations become very similar and therefore also their populations.

The results reported herein advance the currently very active field of aliphatic conformational control by fluorination. On the NMR conformational analysis side, polynomially scaling simulation algorithms significantly expand the substrate complexity scope: 16 spins are handled here, but the favorable computational complexity scaling of the methods involved makes it possible in principle to deal with hundreds.^40^ Just as 1,2-difluoroethane was not an accurate model to evaluate the conformational profile of longer alkanes with vicinal difluorination, this work establishes new benchmarks to evaluate the stabilities of conformations of alkyl chains with 1,3-difluoro-substitution, moving away from the hitherto commonly used simple model of 1,3-difluoropropane.

## Data Availability

The data underlying this study are available in the published article and its Supporting Information. All magnetic resonance simulation scripts are released as a part of the example set of the Spinach library (https://spindynamics.org).

## Supporting Information Available

Details of calculation methods, geometries, and energies of all conformations of all molecules studied within this work, experimental details for the synthesis and characterization of all **12**–**15**, and copies of spectra of all synthesized compounds (PDF)
SDF files of all minimized conformers and the raw thermodynamic data for each conformer. Gaussian16 input and output files (ASCII text) are available on Zenodo (10.5281/zenodo.10618876) (ZIP)

## Abbreviations

CPU: central processing unit
DAST: diethylamino sulfur trifluoride
DFMBA: *N*,*N*-diethyl-α,α-difluoro(*meta*-methylbenzyl)amine
DFT: density functional theory
GIAO: gauge-including-atomic-orbital
NMR: nuclear magnetic resonance
SMD: solvation model density
TMS: trimethylsilyl
