# Supplementary material for: Conformational Analysis of 1,3-Difluorinated Alkanes
Source: J Org Chem. 2024 May 31;89(12):8789–803. doi: 10.1021/acs.joc.4c00670 (PMC11197103; doi:10.1021/acs.joc.4c00670)
Supplement: Supplementary file 1 — jo4c00670_si_003.pdf [file jo4c00670_si_003.pdf]

SUPPORTING INFORMATION

Conformational analysis of 1,3-difluorinated alkanes

William G. Poole, ‡⊥ Florent Peron,‡⊥ Stephen J. Fox,⊥ Neil Wells,⊥ Chris-Kriton Skylaris,⊥ Jonathan W. Essex,⊥ Ilya Kuprov,*⊥ Bruno Linclau*⊥#

⊥School of Chemistry, University of Southampton, Highfield, Southampton, SO17 1BJ, UK. # Department of Organic and Macromolecular Chemistry, Ghent University, Krijgslaan 281-S4, 9000 Ghent, Belgium.

KEYWORDS: conformational analysis, large-scale NMR simulation, fluoroalkanes, 1,3-difluoropropylene: conformational analysis, large-scale NMR simulation, fluoroalkanes, 1,3-difluoropropylene.

Table of contents

1 Conformational analysis 6
1.1 Detailed methodology, discrete conformational analysis 6
1.2 Detailed methodology for conformational energy surface scan 6
1.3 Figure S1: all conformers of *anti*-3,5-difluoropentane (12) 6
1.4 Figure S2: all conformers of *syn*-3,5-difluoropentane (13) 7
1.5 Figure S3: all conformers of *anti*-3,5-difluoroheptane (11) 7
1.6 Figure S4: all conformers of *syn*-3,5-difluoroheptane (15) 10
1.7 Detailed results for the pentanes (M05-2X in combination with 6-311+G**)[a,b] (Table S1) ... 13
1.8 Detailed results for the heptanes (Table S2) 14
1.9 Summary charts S1-S10 15
1.9.1 Chart S1: 1,3-difluoropropane (1) 15
1.9.2 Chart S2: pentane 16
1.9.3 Chart S3: *anti*-2,4-difluoropentane (12) 17
1.9.4 Chart S4: *syn*-2,4-difluoropentane (13) 18
1.9.5 Chart S5: heptane (all dihedrals) 19
1.9.6 Chart S5 (cont'd): heptane (all dihedrals) 20
1.9.7 Chart S6: *anti*-3,5-difluoroheptane (14) (all dihedrals) 21
1.9.8 Chart S6 (cont'd): *anti*-3,5-difluoroheptane (14) (all dihedrals) 22
1.9.9 Chart S7: *syn*-3,5-difluoroheptane (15) (all dihedrals) 23
1.9.10 Chart S7 (cont'd): *syn*-3,5-difluoroheptane (15) (all dihedrals) 24
1.9.11 Figure S5: conversion from 9 × 9 to a 3 × 3 grid 25
1.9.12 Chart S8: heptane (inner dihedrals) 26

1.9.13 Chart S9: *anti*-3,5-difluoroheptane (14) (inner dihedrals) ... 26
1.9.14 Chart S10: *syn*-3,5-difluoroheptane (15) (inner dihedrals)... 26
1.9.15 Chart S11: *anti*-3,5-difluoroheptane (14) (outer dihedrals, population). ... 27
1.9.16 Chart S12: *syn*-3,5-difluoroheptane (15) (outer dihedrals, population). ... 27
2 Synthesis of (±)-*anti* and *syn*-2,4-difluoropentane (12 and 13)... 28
2.1 General conditions... 28
2.2 General procedure for the fluorination of fluorohydrins 12 and 13... 28
2.3 (±)-*anti*-2,4-difluoropentane (12) ... 29
2.4 *syn*-2,4-difluoropentane (13)... 29
3 Synthesis of (±)-*anti*- and *meso*-3,5-difluoroheptane (14 and 15) ... 30
3.1 General scheme ... 30
3.2 Synthesis of 3-hydroxyheptan-5-one (16)[3] ... 30
3.3 *Anti* reduction: synthesis of (±)-*anti*-heptane-3,5-diol (17)... 31
3.4 *Syn* reduction: synthesis of *syn*-heptane-3,5-diol (18) ... 31
3.5 Synthesis of (±)-*anti* and (±)-*syn*-3-(3'-methylbenzoyloxy)-5-fluoroheptane (*anti*-SI8 and *syn*-SI9) 32
3.5.1 General procedure for the mono-deoxyfluorination of diols using DFMBA in diglyme 32
3.5.2 (±)-*syn*-3-(3'-methylbenzoyloxy)-5-fluoroheptane (S3) ... 32
3.5.3 (±)-*anti-3*-(3'-methylbenzoyloxy)-5-fluoroheptane (S4)... 33
3.6 Synthesis of fluorohydrins 19 and 20... 33
3.6.1 General procedure for the hydrolysis of S3 and S4... 33
3.6.2 (±)-*syn*-5-fluoroheptan-3-ol (19) ... 33
3.6.3 (±)-*anti*-5-fluoroheptan-3-ol (20)... 34
3.7 Synthesis of (±)-*anti*- and *syn*-3,5-difluoropheptane (14 and 15)... 34
3.7.1 General procedure for the fluorination of fluorohydrins SI8 and SI9 ... 34
3.7.2 (±)-*anti*-3,5-difluoroheptane (14) ... 34
3.7.3 *syn*-3,5-difluoroheptane (15)... 35
4 Copies of the NMR spectra of compounds 9–15 (Figures S6-S17)... 36
4.1 ^{1}H NMR spectra (Figures S6-S10)... 36
4.1.1 *Anti*-2,4-difluoropentane 12 (^{1}H NMR, $CDCl_3$, 500 MHz, Figure S6a)... 36
4.1.2 *Anti*-2,4-difluoropentane 12 (^{1}H{^{19}F} NMR, $CDCl_3$, 500 MHz, Figure S6b)... 36
4.1.3 *Syn*-2,4-difluoropentane 13 (^{1}H NMR, $CDCl_3$, 500 MHz, Figure S7a)... 37
4.1.4 *Syn*-2,4-difluoropentane 13 (^{1}H{^{19}F} NMR, $CDCl_3$, 500 MHz, Figure S7b)... 37

4.1.5 *Anti*-2,4-difluoroheptane 14 (^{1}H NMR, $CDCl_3$, 500 MHz, Figure S8a)............................. 38
4.1.6 *Anti*-2,4-difluoroheptane 14 (^{1}H{^{19}F} NMR, $CDCl_3$, 500 MHz, Figure S8b)....................... 38
4.1.7 *Syn*-2,4-difluoroheptane 15 (^{1}H NMR, $CDCl_3$, 500 MHz, Figure S9a)............................. 39
4.1.8 *Syn*-2,4-difluoroheptane 15 (^{1}H{^{19}F} NMR, $CDCl_3$, 500 MHz, Figure S9b)....................... 39
4.2 ^{19}F NMR spectra (Figures S10-S13)... 40
4.2.1 *Anti*-2,4-difluoropentane 12 (^{19}F NMR, $CDCl_3$, 471 MHz, Figure S10a)........................... 40
4.2.2 *Anti*-2,4-difluoropentane 12 (^{19}F{^{1}H} NMR, $CDCl_3$, 471 MHz, Figure S10b)..................... 40
4.2.3 *Syn*-2,4-difluoropentane 13 (^{19}F NMR, $CDCl_3$, 471 MHz, Figure S11a)........................... 41
4.2.4 *Syn*-2,4-difluoropentane 13 (^{19}F{1H} NMR, $CDCl_3$, 471 MHz, Figure S11b).................... 41
4.2.5 *Anti*-2,4-difluoroheptane 14 (^{19}F NMR, $CDCl_3$, 471 MHz, Figure S12a)........................... 42
4.2.6 *Anti*-2,4-difluoroheptane 14 ^{19}F{^{1}H} NMR ($CDCl_3$, 471 MHz, Figure S12b)...................... 42
4.2.7 *Syn*-2,4-difluoroheptane 15 (^{19}F NMR, $CDCl_3$, 471 MHz, Figure S13a)........................... 43
4.2.8 *Syn*-2,4-difluoroheptane 15 (^{19}F{^{1}H} NMR, $CDCl_3$, 471 MHz, Figure S13b)..................... 43
4.3 ^{13}C{1H} NMR spectra ($CDCl_3$, 126 MHz)(Figures S14-S17)... 44
4.3.1 *Anti*-2,4-difluoropentane 12 (^{13}C{^{1}H} NMR, $CDCl_3$, 126 MHz, Figure S14)...................... 44
4.3.2 *Syn*-2,4-difluoropentane 13 (^{13}C{^{1}H} NMR, $CDCl_3$, 126 MHz, Figure S15)...................... 44
4.3.3 *Anti*-3,5-difluoroheptane 14 (^{13}C{^{1}H} NMR, $CDCl_3$, 126 MHz, Figure S16)...................... 45
4.3.4 *Syn*-3,5-difluoroheptane 15 (^{13}C{^{1}H} NMR, $CDCl_3$, 126 MHz, Figure S17)...................... 45
5 NMR fitting.. 46
5.1 Systematic grid scan of 1,3-difluoropropane dihedral angles (Figure S18).......................... 46
5.2 Fitted NMR spectra for compounds 12-15 (Figures S19-S22).. 47
5.2.1 *Anti*-2,4-difluoropentane 12 (Figure S19).. 47
5.2.2 *Syn*-2,4-difluoropentane 13 (Figure S20).. 48
5.2.3 *Anti*-3,5-difluoroheptane 14 (Figure S21).. 49
5.2.4 *Syn*-3,5-difluoroheptane 15 (Figure S22).. 50
5.3 Simulation spectra using the set of DFT *J*-couplings (for 14 and 15, Figures S23, S24). 51
5.4 Simulation of the F-decoupled H NMR spectra with the obtained fitting parameters (Figures S25, S26) 52
5.4.1 Figure S25: *anti*-3,5-difluoroheptane 14.. 52
5.4.2 Figure S26: *syn*-3,5-difluoroheptane 15.. 53
6 Full lists of “data fitted” coupling constants for 1,12-15 54
6.1 Table S3: chemical shifts of 1,3-difluoropropane (1), obtained by fitting experimental data. 54
6.2 Table S4: 1,3-difluoropropane (1), *J*-couplings (Hz)... 54

6.3 Table S5: chemical shifts of *anti*-2,4-difluoropentane (12), obtained by fitting experimental data. 55

6.4 Table S6: *anti*-2,4-difluoropentane (12), *J*-couplings (Hz). 55

6.5 Table S7: chemical shifts of *syn*-2,4-difluoropentane (13), obtained by fitting experimental data. 56

6.6 Table S8: *syn*-2,4-difluoropentane (13), *J*-couplings (Hz). 56

6.7 Table S9: chemical shifts of *anti*-3,5-difluoroheptane (14), obtained by fitting experimental data. 57

6.8 Table S10: *anti*-3,5-difluoroheptane (14), *J*-couplings (Hz). 57

6.9 Table S11. chemical shifts of *syn*-3,5-difluoroheptane (15), obtained by fitting experimental data. 58

6.10 Table S12. *syn*-3,5-difluoroheptane (15), *J*-couplings (Hz). 58

7 Table S13: Comparison between the populations derived from minimum energy calculations and relaxed potential energy scans 59

8 Figure S27: Calculations of %antiperiplanar for the dihedrals in the *J*-value analysis as in Table 5 in the manuscript (in $CHCl_3$) 60

8.1 Inner dihedrals 60

8.2 Figures S28, S29: outer dihedrals (heptanes) 61

8.2.1 *Anti*-3,5-difluoroheptane (Figure S28) 61

8.2.2 *Syn*-3,5-difluoroheptane (Figure S29) 62

See Summary Chart S12 62

9 Table S14: full coupling constant analysis (extended Table 5 in manuscript) 63

10 Figure S30: Hydrocarbon chain population changes according to the medium, for pentane, heptane, and the *anti*-substrates 12 and 14 64

11 Copies of spectra of synthetic intermediates 65

11.1 3-hydroxyheptan-5-one (SI3) 65

11.1.1 ^{1}H NMR ($CDCl_3$, 400 MHz) 65

11.1.2 $^{13}C\{1H\}$ NMR ($CDCl_3$, 101 MHz) 65

11.2 (±)-*anti*-heptane-3,5-diol (SI4) 66

11.2.1 ^{1}H NMR ($CDCl_3$, 400 MHz) 66

11.2.2 $^{13}C\{1H\}$ NMR ($CDCl_3$, 101 MHz) 66

11.3 *syn*-heptane-3,5-diol (SI5) 67

11.3.1 ^{1}H NMR ($CDCl_3$, 400 MHz) 67

11.3.2 $^{13}C\{1H\}$ NMR ($CDCl_3$, 101 MHz) 67

11.4 (±)-*syn*-3-(3'-methylbenzoyloxy)-5-fluoroheptane (SI6) 68

11.4.1 ^{1}H NMR ($CDCl_3$, 400 MHz) 68
11.4.2 $^{13}C\{1H\}$ NMR ($CDCl_3$, 101 MHz) 68
11.4.3 ^{112}F NMR ($CDCl_3$, 376 MHz) 69
11.4.4 $^{19}F\{^{1}H\}$ NMR ($CDCl_3$, 376 MHz) 69
11.5 (±)-*anti-3*-(3'-methylbenzoyloxy)-5-fluoroheptane (SI7) 70
11.5.1 ^{1}H NMR ($CDCl_3$, 400 MHz) 70
11.5.2 $^{13}C\{1H\}$ NMR ($CDCl_3$, 101 MHz) 70
11.5.3 ^{19}F NMR ($CDCl_3$, 376 MHz) 71
11.5.4 $^{19}F\{^{1}H\}$ NMR ($CDCl_3$, 376 MHz) 71
11.6 (±)-*syn*-5-fluoroheptan-3-ol (SI8) 72
11.6.1 ^{1}H NMR ($CDCl_3$, 500 MHz) 72
11.6.2 $^{13}C\{1H\}$ NMR ($CDCl_3$, 126 MHz) 72
11.6.3 ^{19}F NMR ($CDCl_3$, 471 MHz) 73
11.6.4 $^{19}F\{^{1}H\}$ NMR ($CDCl_3$, 471 MHz) 73
11.7 (±)-*anti*-5-fluoroheptan-3-ol (SI9) 74
11.7.1 ^{1}H NMR ($CDCl_3$, 500 MHz) 74
11.7.2 $^{13}C\{1H\}$ NMR ($CDCl_3$, 126 MHz) 74
11.7.3 ^{19}F NMR ($CDCl_3$, 471 MHz) 75
11.7.4 $^{19}F\{^{1}H\}$ NMR ($CDCl_3$, 471 MHz) 75

1 Conformational analysis

1.1 Detailed methodology, discrete conformational analysis

For each of the molecules studied in this work (propane, 1,3-difluoropropane, pentane, syn/anti-2,4-difluoropentane, heptane and syn/anti-3,5-difluoroheptane), initial coordinates of the known minima were generated using RDKit 2022.09.3. Each conformer was generated using dihedral angles of –60°, 60° and 180° for G–, G, and A respectively. This led to 9 distinct conformers for the propanes and pentanes (Figures S1,S2), and 81 conformers for heptanes (Figures S3, S4). Conformers were then subjected to a DFT minimization using the M05-2X functional with the 6-311+G** basis set. Energy minimisations were performed in *vacuo* and the SMD implicit solvent models of water and chloroform. Vibrational frequency calculations were performed, including numerical differentiation to compute an-harmonic frequencies and zero-point free energies. All calculations were performed using *Gaussian16*.

A dihedral angle was assigned to G–, G or A based on whether the measured angle was within a 30° range of its initial angle (G–: –30° to –90°, G: 30° to 90°, A: 150° to 210°/–150° to –210°).

The energies of each conformer were counted relative to the lowest energy conformer; populations were calculated using Boltzmann's law at 298 K.

1.2 Detailed methodology for conformational energy surface scan

DFT energy surface scans were performed using M06[1] exchange-correlation functional with cc-pVTZ[2] basis set and SMD implicit solvation model ($CHCl_3$, H_2O)[3]. Conformational surfaces of 1,3-difluoropro-pane and 1,3-difluoropentane were sampled systematically by performing a relaxed energy scan over the two pertinent dihedral angles. The four-dimensional conformational surface of 1,3-difluoroheptane (which has four pertinent dihedral angles) was sampled using the Monte-Carlo method: 10,000 molec-ular geometries with randomly selected sets of four dihedral angles were generated for each of the two 3,5-difluoroheptane isomers and screened for atomic clashes. Approximately 4,000 geometries that have survived the screening were submitted for a constrained optimisation, wherein all coordinates other than those dihedral angles were optimised into a minimum. The resulting sets of geometries was sub-mitted for *J*-coupling calculations using the GIAO DFT M06/cc-pVDZ method[1, 4] in SMD[3] chloroform (basis decontracted and augmented with tight functions[5] at the Fermi contact coupling calculation stage). All calculations were performed using *Gaussian16*[6].

1.3 Figure S1: all conformers of *anti*-3,5-difluoropentane (12)

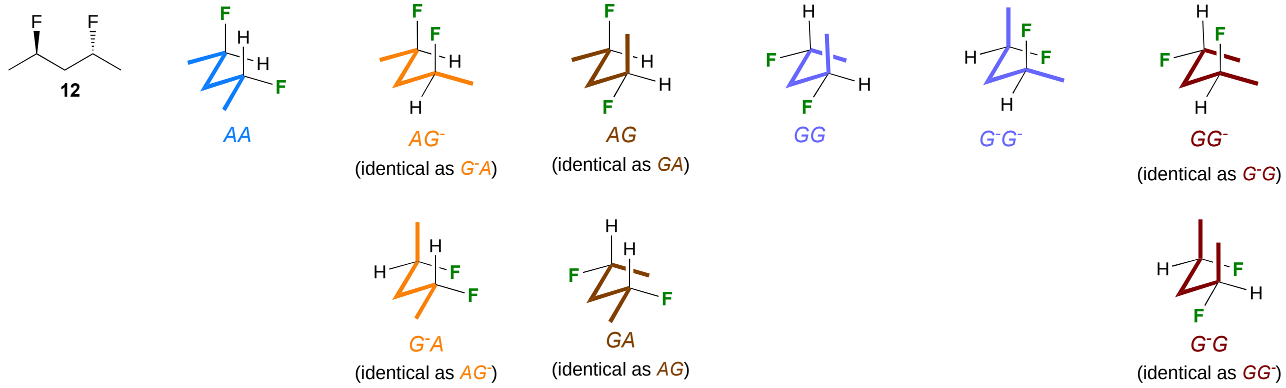

1.4 Figure S2: all conformers of *syn*-3,5-difluoropentane (13)

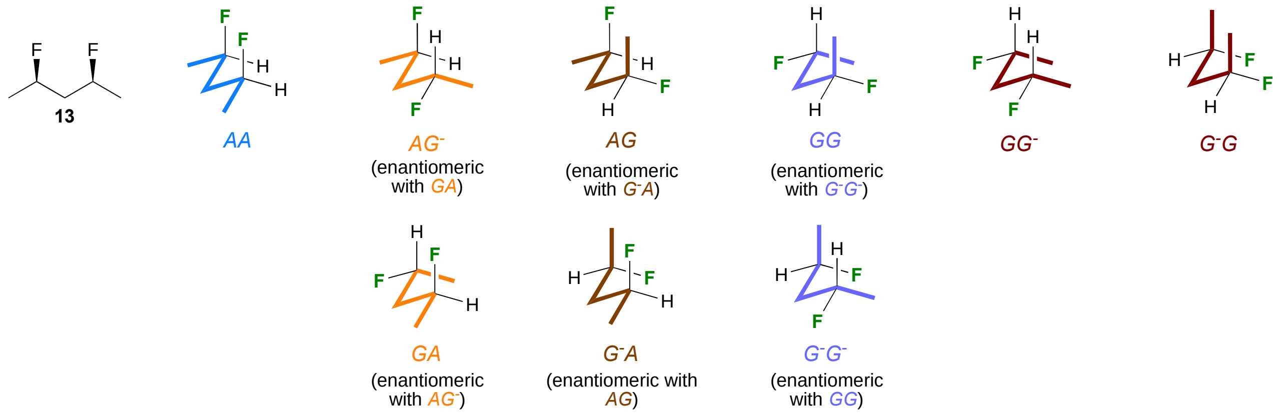

1.5 Figure S3: all conformers of *anti*-3,5-difluoroheptane (11)

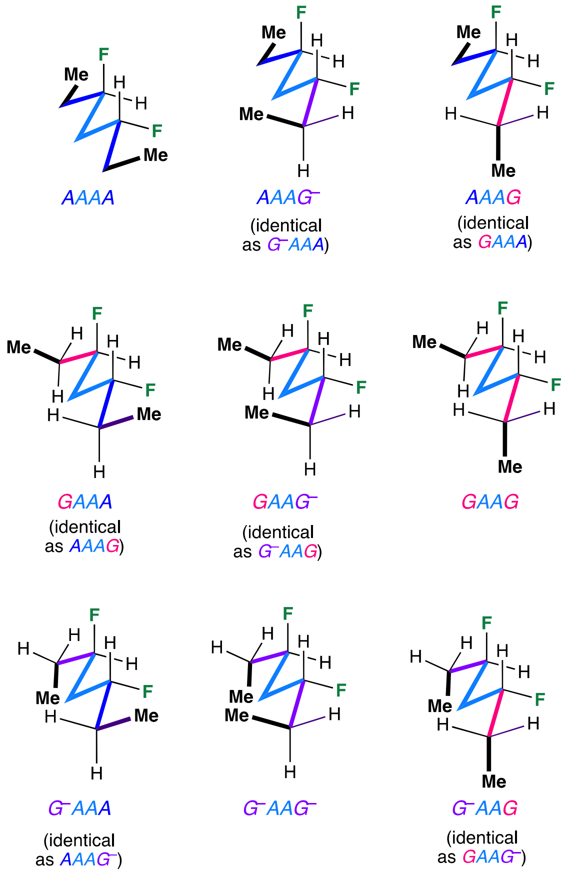

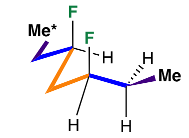

$AAG^{-}A$
(identical as $AG^{-}AA$)

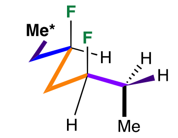

$AAG^{-}G^{-}$
(identical as $G^{-}G^{-}AA$)

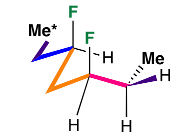

$AAG^{-}G$
(identical as $GG^{-}AA$)

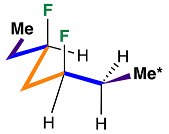

$AG^{-}AA$
(identical as $AAG^{-}A$)

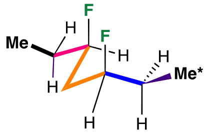

$AG^{-}AG$
(identical as $GAG^{-}A$)

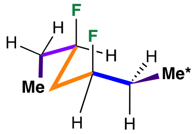

$AG^{-}AG^{-}$
(identical as $G^{-}AG^{-}A$)

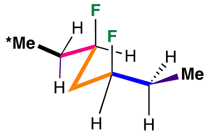

$GAG^{-}A$
(identical as $AG^{-}AG$)

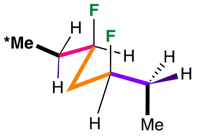

$GAG^{-}G^{-}$
(identical as $G^{-}G^{-}AG$)

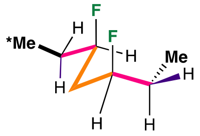

$GAG^{-}G$
(identical as $GG^{-}AG$)

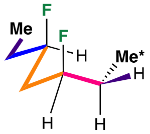

$GG^{-}AA$
(identical as $AAG^{-}G$)

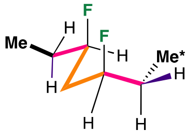

$GG^{-}AG$
(identical as $GAG^{-}G$)

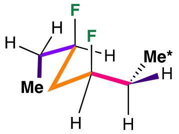

$GG^{-}AG^{-}$
(identical as $G^{-}AG^{-}G$)

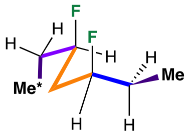

$G^{-}AG^{-}A$
(identical as $AG^{-}AG^{-}$)

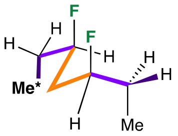

$G^{-}AG^{-}G^{-}$
(identical as $G^{-}G^{-}AG^{-}$)

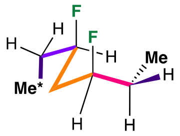

$G^{-}AG^{-}G$
(identical as $GG^{-}AG^{-}$)

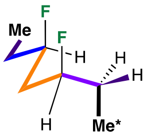

$G^{-}G^{-}AA$
(identical as $AAG^{-}G^{-}$)

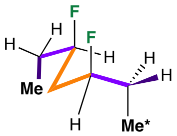

$G^{-}G^{-}AG^{-}$
(identical as $G^{-}AG^{-}G^{-}$)

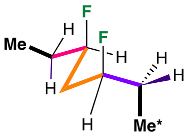

$G^{-}G^{-}AG$
(identical as $GAG^{-}G^{-}$)

Figure S3 continued:

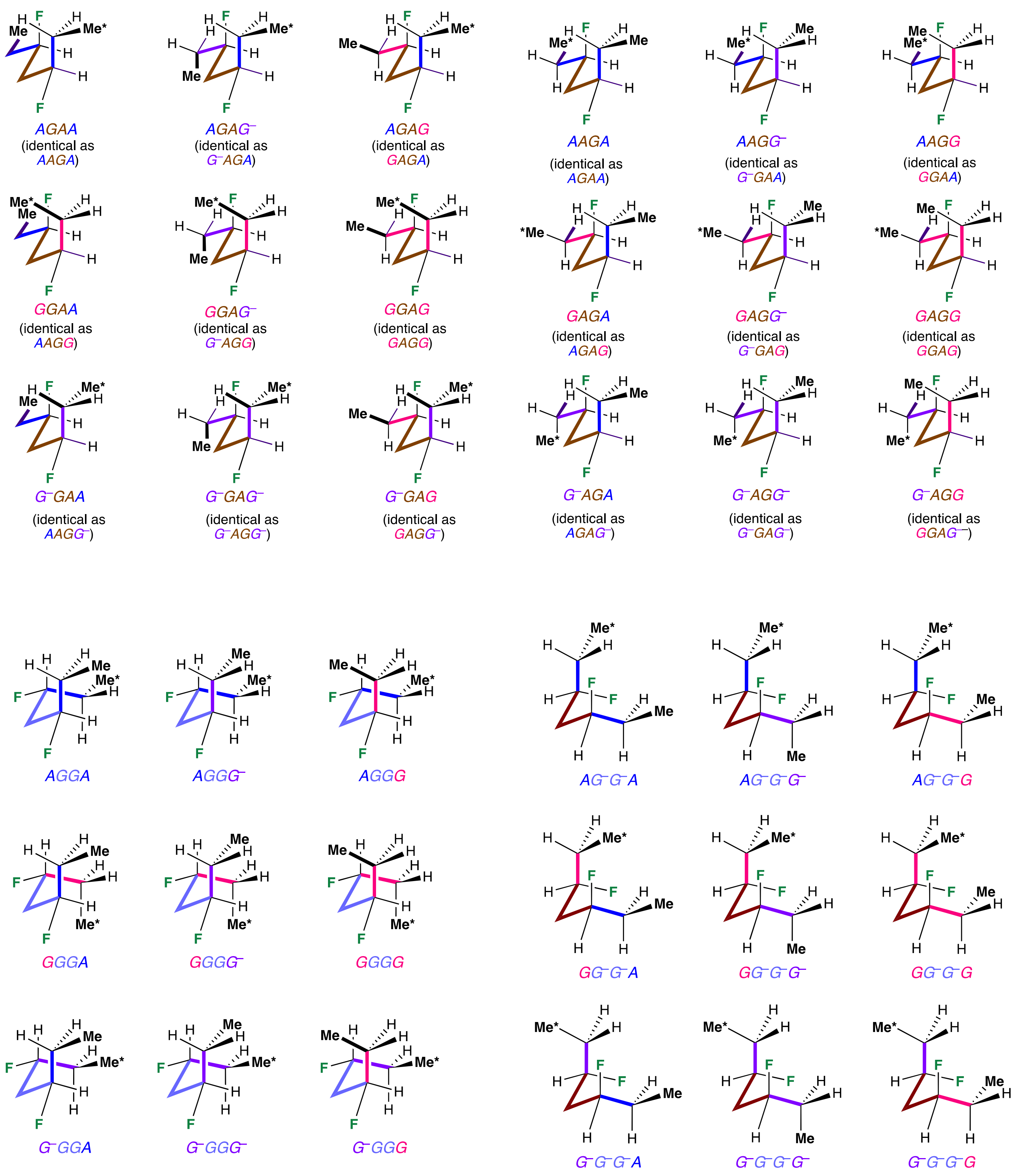

Figure S3 continued:

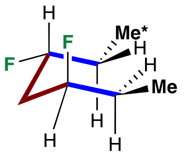
$AGG^{-}A$ (identical as $AG^{-}GA$)

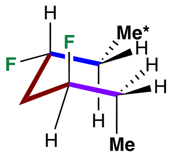
$AGG^{-}G^{-}$ (identical as $G^{-}G^{-}GA$)

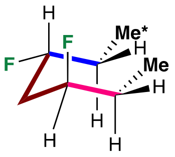
$AGG^{-}G$ (identical as $GG^{-}GA$)

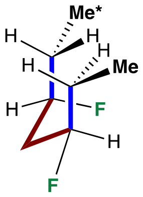
$AG^{-}GA$ (identical as $AGG^{-}A$)

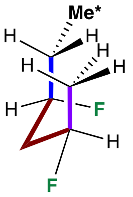
$AG^{-}GG^{-}$ (identical as $G^{-}GG^{-}A$)

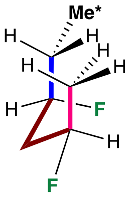
$AG^{-}GG$ (identical as $GGG^{-}A$)

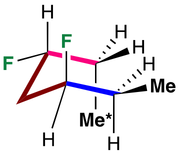
$GGG^{-}A$ (identical as $AG^{-}G^{-}G$)

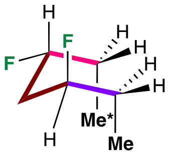
$GGG^{-}G^{-}$ (identical as $G^{-}G^{-}GG$)

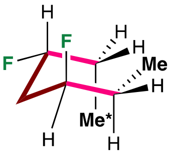
$GGG^{-}G$ (identical as $GG^{-}GG$)

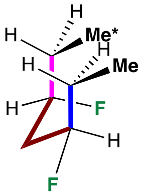
$GG^{-}GA$ (identical as $AGG^{-}G$)

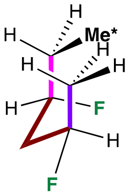
$GG^{-}GG^{-}$ (identical as $G^{-}GG^{-}G$)

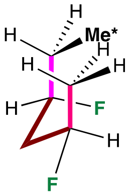
$GG^{-}GG$ (identical as $GGG^{-}G$)

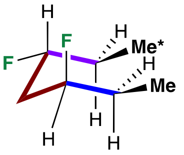
$G^{-}GG^{-}A$ (identical as $AG^{-}GG^{-}$)

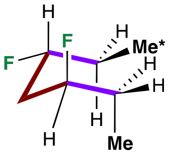
$G^{-}GG^{-}G^{-}$ (identical as $G^{-}G^{-}GG^{-}$)

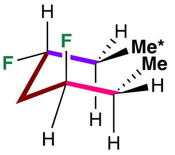
$G^{-}GG^{-}G$ (identical as $GG^{-}GG^{-}$)

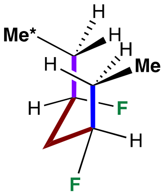
$G^{-}G^{-}GA$ (identical as $AGG^{-}G^{-}$)

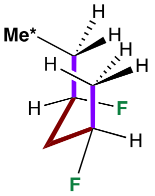
$G^{-}G^{-}GG^{-}$ (identical as $G^{-}GG^{-}G^{-}$)

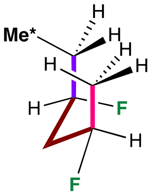
$G^{-}G^{-}GG$ (identical as $GGG^{-}G^{-}$)

1.6 Figure S4: all conformers of *syn*-3,5-difluoroheptane (15)

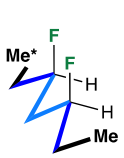
$AAAA$

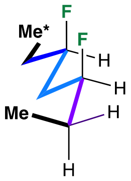
$AAAG^{-}$

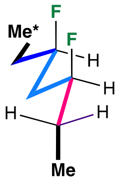
$AAAG$

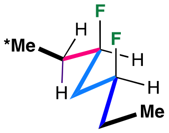
$GAAA$

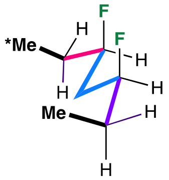
$GAAG^{-}$

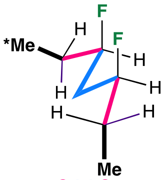
$GAAG$

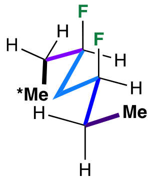
$G^{-}AAA$

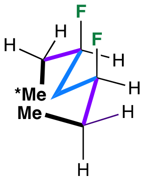
$G^{-}AAG^{-}$

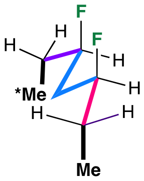
$G^{-}AAG$

Figure S4 continued:

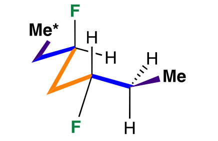

$AAG^{-}A$
(enantiomeric with
$AGAA$)

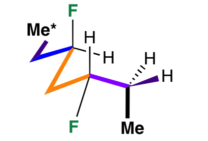

$AAG^{-}G^{-}$
(enantiomeric with
$GGAA$)

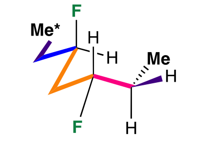

$AAG^{-}G$
(enantiomeric with
$G^{-}GAA$)

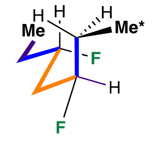

$AGAA$
(enantiomeric with
$AAG^{-}A$)

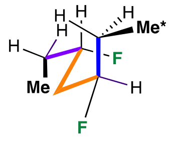

$AGAG^{-}$
(enantiomeric with
$GAG^{-}A$)

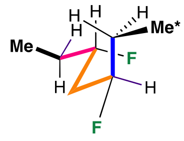

$AGAG$
(enantiomeric with
$G^{-}AG^{-}A$)

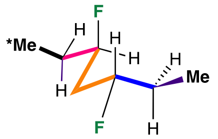

$GAG^{-}A$
(enantiomeric with
$AGAG^{-}$)

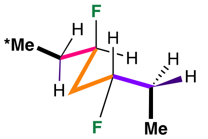

$GAG^{-}G^{-}$
(enantiomeric with
$GGAG^{-}$)

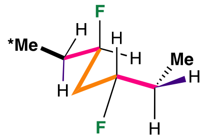

$GAG^{-}G$
(enantiomeric with
$G^{-}GAG^{-}$)

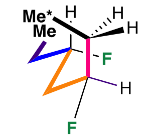

$GGAA$
(enantiomeric with
$AAG^{-}G^{-}$)

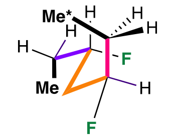

$GGAG^{-}$
(enantiomeric with
$GAG^{-}G^{-}$)

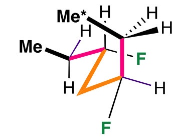

$GGAG$
(enantiomeric with
$G^{-}AG^{-}G^{-}$)

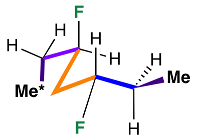

$G^{-}AG^{-}A$
(enantiomeric with
$AGAG$)

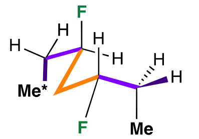

$G^{-}AG^{-}G^{-}$
(enantiomeric with
$GGAG$)

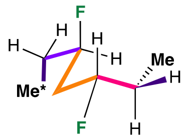

$G^{-}AG^{-}G$
(enantiomeric with
$G^{-}GAG$)

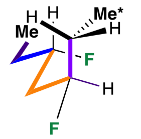

$G^{-}GAA$
(enantiomeric with
$AAG^{-}G$)

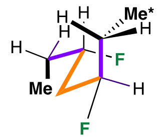

$G^{-}GAG^{-}$
(enantiomeric with
$GAG^{-}G$)

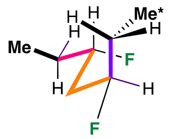

$G^{-}GAG$
(enantiomeric with
$G^{-}AG^{-}G$)

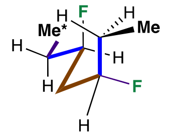

$AAGA$
(enantiomeric with
$AG^{-}AA$)

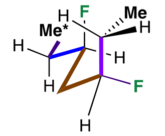

$AAGG^{-}$
(enantiomeric with
$GG^{-}AA$)

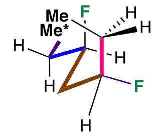

$AAGG$
(enantiomeric with
$G^{-}G^{-}AA$)

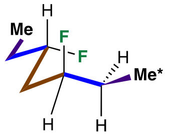

$AG^{-}AA$
(enantiomeric with
$AAGA$)

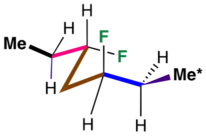

$AG^{-}AG$
(enantiomeric with
$G^{-}AGA$)

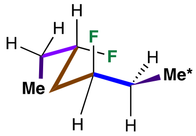

$AG^{-}AG^{-}$
(enantiomeric with
$GAGA$)

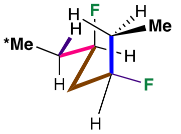

$GAGA$
(enantiomeric with
$AG^{-}AG^{-}$)

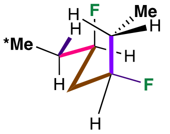

$GAGG^{-}$
(enantiomeric with
$GG^{-}AG^{-}$)

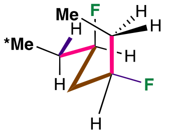

$GAGG$
(enantiomeric with
$G^{-}G^{-}AG^{-}$)

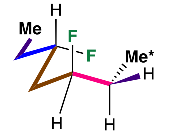

$GG^{-}AA$
(enantiomeric with
$AAGG^{-}$)

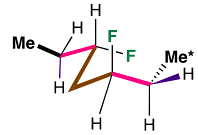

$GG^{-}AG$
(enantiomeric with
$G^{-}AGG^{-}$)

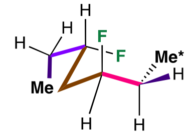

$GG^{-}AG^{-}$
(enantiomeric with
$GAGG^{-}$)

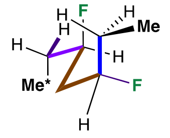

$G^{-}AGA$
(enantiomeric with
$AG^{-}AG$)

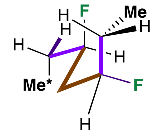

$G^{-}AGG^{-}$
(enantiomeric with
$GG^{-}AG$)

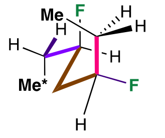

$G^{-}AGG$
(enantiomeric with
$G^{-}G^{-}AG$)

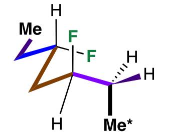

$G^{-}G^{-}AA$
(enantiomeric with
$AAGG$)

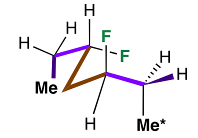

$G^{-}G^{-}AG^{-}$
(enantiomeric with
$GAGG$)

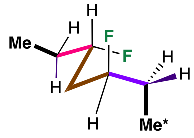

$G^{-}G^{-}AG$
(enantiomeric with
$G^{-}AGG$)

Figure S4 continued:

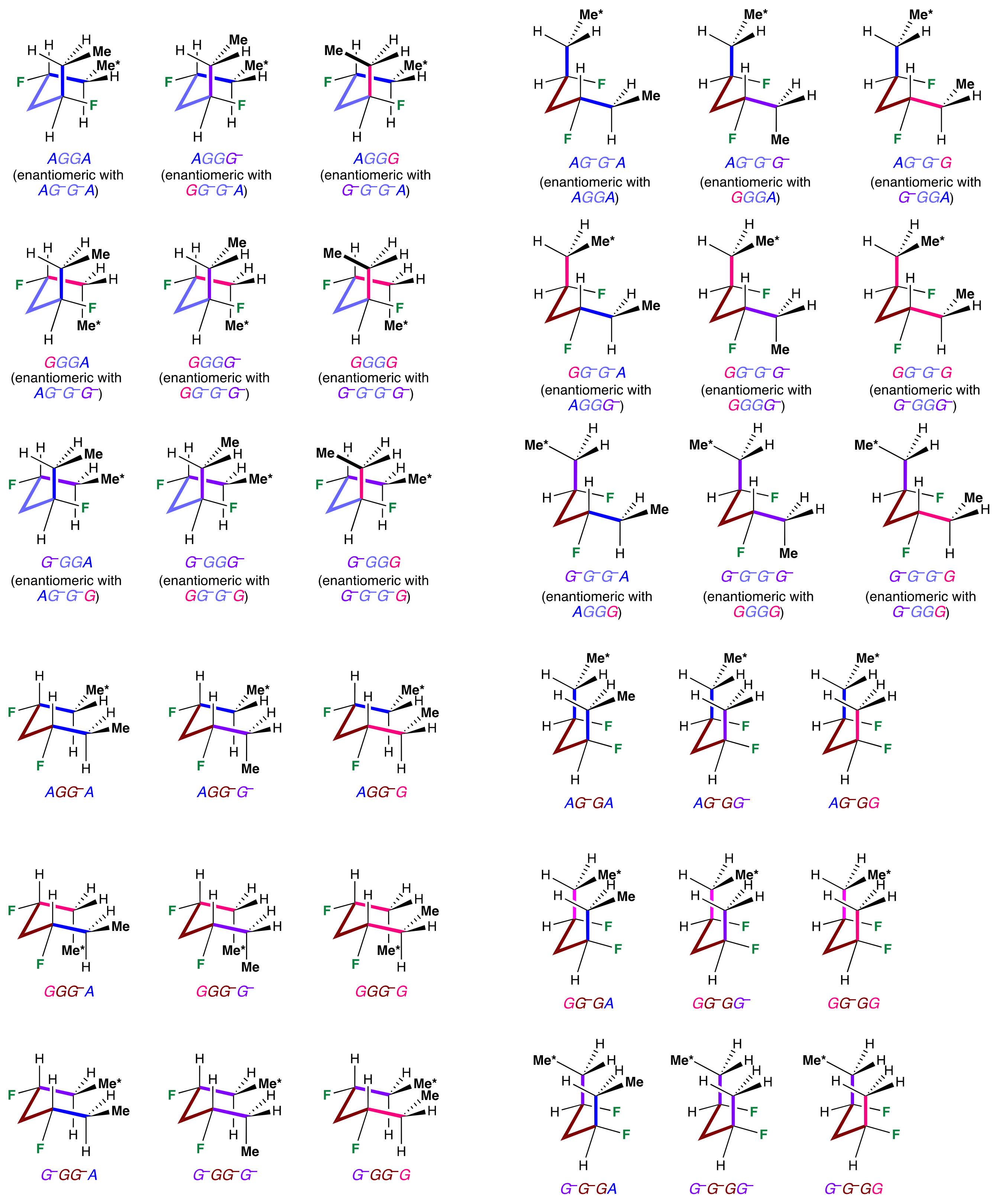

1.7 Detailed results for the pentanes (M05-2X in combination with 6-311+G**)[a,b] (Table S1)

Table S1. Conformational profile of pentane (A), *dl*-difluoropentane 12 (B), and *meso*-difluoropentane 13 (C) Dihedral angles refer to rotation along the central CC–CC bonds.

	2 x anti C-C	1 anti C-C, 1 gauche C-C	2 x gauche C-C	

A

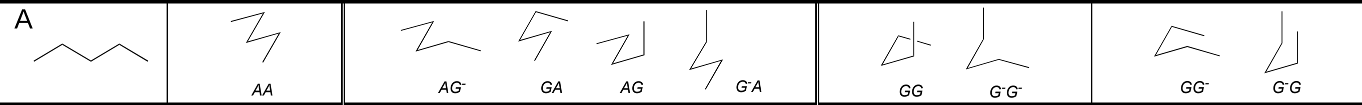

		AA	AG⁻, GA, AG, G⁻A	GG, G⁻G⁻	GG⁻, G⁻G
Vacuum	μ	0.1 D	0.1 D	0.1 D	[d]
	E_{rel}	0.0	3.1	5.7	[d]
	p	42.8%	4×12.2% (48.8%)[c]	2×4.2% (8.2%)[c]	0%
$CHCl_3$	μ	0.1 D	0.1 D	0.1 D	[d]
	E_{rel}	0.0	5.1	8.5	[d]
	p	63.2%	4×8.2% (32.8%)[c]	2×2.0% (4.0%)[c]	0%
H_2O	μ	0.1 D	0.1 D	0.1 D	[d]
	E_{rel}	0.0	2.8	4.1	[d]
	p	37.1%	4×12.2% (48.8%)[c]	2×7.0% (14.0%)[c]	0%

B

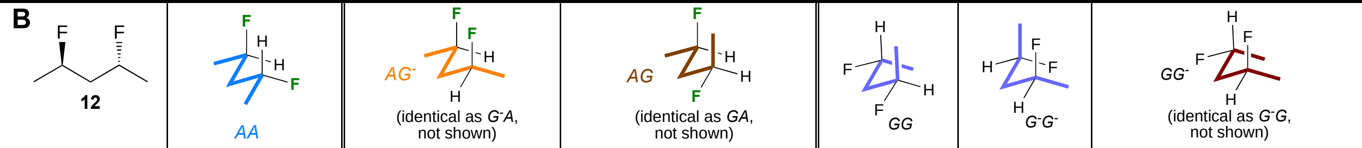

		AA	AG⁻	AG	GG	G⁻G⁻	GG⁻
Vacuum	μ	2.4 D	4.0 D	2.0 D	2.8 D	1.7 D	[d]
	E_{rel}	0.0	17.5	4.3	11.8	13.5	[d]
	p	73.3%	0.1% (0.2%)[c]	12.8 (25.6)[c]	0.6%	0.3%	0.0%
$CHCl_3$	μ	3.1 D	5.2 D	2.8 D	3.5 D	2.1 D	[d]
	E_{rel}	0.0	12.5	6.4	10.4	15.4	[d]
	p	84.8%	0.5 (1.0%)[c]	6.3% (12.6%)[c]	1.3%	0.2%	0.0%
H_2O	μ	3.5 D	5.8 D	3.1 D	3.8 D	2.4 D	[d]
	E_{rel}	0.0	6.9	6.1	8.8	14.2	[d]
	p	75.5%	4.6% (9.2%)[c]	6.5% (13.0%)[c]	2.1%	0.2%	0.0%

C

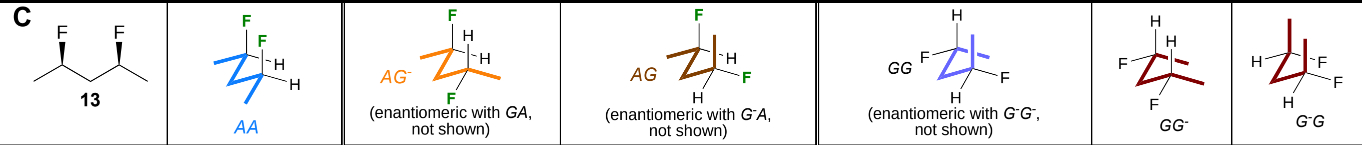

		AA	AG⁻	AG	GG	GG⁻	G⁻G
Vacuum	μ	4.1 D	2.5 D	1.8 D	2.3 D	[d]	3.9 D
	E_{rel}	13.3	0.0	0.9	6.6	[d]	26.2
	p	0.1%	28.4% (56.8%)[c]	19.6% (39.2%)[c]	2.0% (4.0%)[c]	0.0%	0.0%
$CHCl_3$	μ	5.3 D	3.1 D	2.2 D	2.8 D	[d]	5.0 D
	E_{rel}	4.1	0.0	1.5	5.6	[d]	22.3
	p	5.6%	28.8% (57.6%)[c]	15.5% (31%)[c]	3.0 (6.0%)[c]	0.0%	0.0%
H_2O	μ	5.9 D	3.4 D	2.5 D	3.2 D	[d]	5.7 D
	E_{rel}	0.8	0.0	3.5	6.7	[d]	17.5
	p	21.8%	29.9% (59.8%)[c]	7.2% (14.4%)[c]	2.0% (4.0%)[c]	0.0%	0.0%

[a] Energies in kJ.mol^{-1}. [b] Degenerate structures are shown. [c] Sum of populations of the degenerate conformations. [d] Structure not obtained in energy minimisation.

1.8 Detailed results for the heptanes (Table S2)

Table S2. Conformational profile of pentane (A), *dl*-difluoropentane 12 (B), and *meso*-difluoropentane 13 (C) (M05-2X in combination with 6-311+G).[a,b] Dihedral angles refer to rotation along the central CC–CC bonds.**

		2 x anti C-C	1 anti C-C, 1 gauche C-C		2 x gauche C-C			
**A** 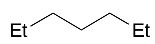		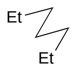 *AA*	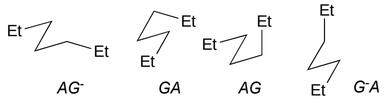 *AG⁻*, *GA*, *AG*, *G⁻A*		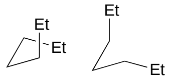 *GG*, *G⁻G⁻*		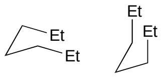 *GG⁻*, *G⁻G*	
Vacuum	**P**[d]	31.5	14.9% (59.6%)[c]		4.5% (9.0%)[c]		0.0	
$CHCl_3$	P[d]	42.4	13.3% (54.2%)[c]		2.2% (4.4%)[c]		0.0	
H_2O	**P**[d]	31.7	13.8% (55.2%)[c]		6.6% (13.2%)[c]		0.0	
**B** 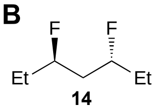 **14**		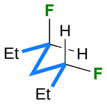 *AA*	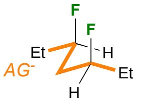 *AG⁻* (identical as *G⁻A*, not shown)	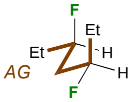 *AG* (identical as *GA*, not shown)	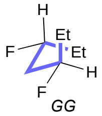 *GG*	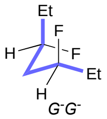 *G⁻G⁻*	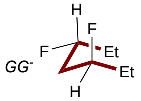 *GG⁻* (identical as *G⁻G*, not shown)	
Vacuum	**p**[d]	79.9	0.1% (0.2%)[c]	9.6% (19.2%)[c]	0.5	0.3	0.0	
$CHCl_3$	p[d]	89.7	0.5% (1.0%)[c]	4.1% (8.2%)[c]	0.9	0.2	0.0	
H_2O	**p**[d]	83.8	2.8% (5.6%)	4.5% (9.0%)[c]	1.3	0.2	0.0	
**C** 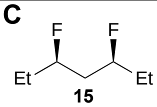 **15**		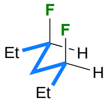 *AA*	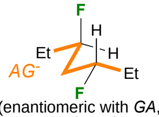 *AG⁻* (enantiomeric with *GA*, not shown)	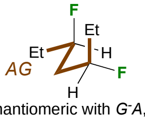 *AG* (enantiomeric with *G⁻A*, not shown)	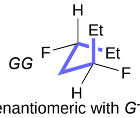 *GG* (enantiomeric with *G⁻G⁻*, not shown)		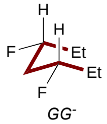 *GG⁻*	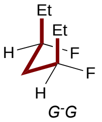 *G⁻G*
Vacuum	**p**[d]	0.7	2 x 21% (42.0%)[c]	2 x 27.0% (54.0%)[c]	2 x 1.7% (3.4%)[c]		0.0	0.0
$CHCl_3$	**p**[d]	4.9	2 x 26% (52.0%)[c]	2 x 19.9% (39.8%)[c]	2 x 1.6% (3.2%)[c]		0.0	0.0
H_2O	**p**[d]	23.8	2 x 18.3% (36.6%)[c]	2 x 17.5% (35.0%)[c]	2 x 2.3% (4.6%)[c]		0.0	0.0

[a] Energies in kJ.mol^{-1}, (weighted average of all 9 possible conformers for each defined conformation of the two central CC–CC bond). [b] Degenerate structures are shown. [c] Sum of populations of the degenerate conformations. [d] Structure not obtained in energy minimisation.

1.9 Summary charts S1-S10

1.9.1 Chart S1: 1,3-difluoropropane (1)

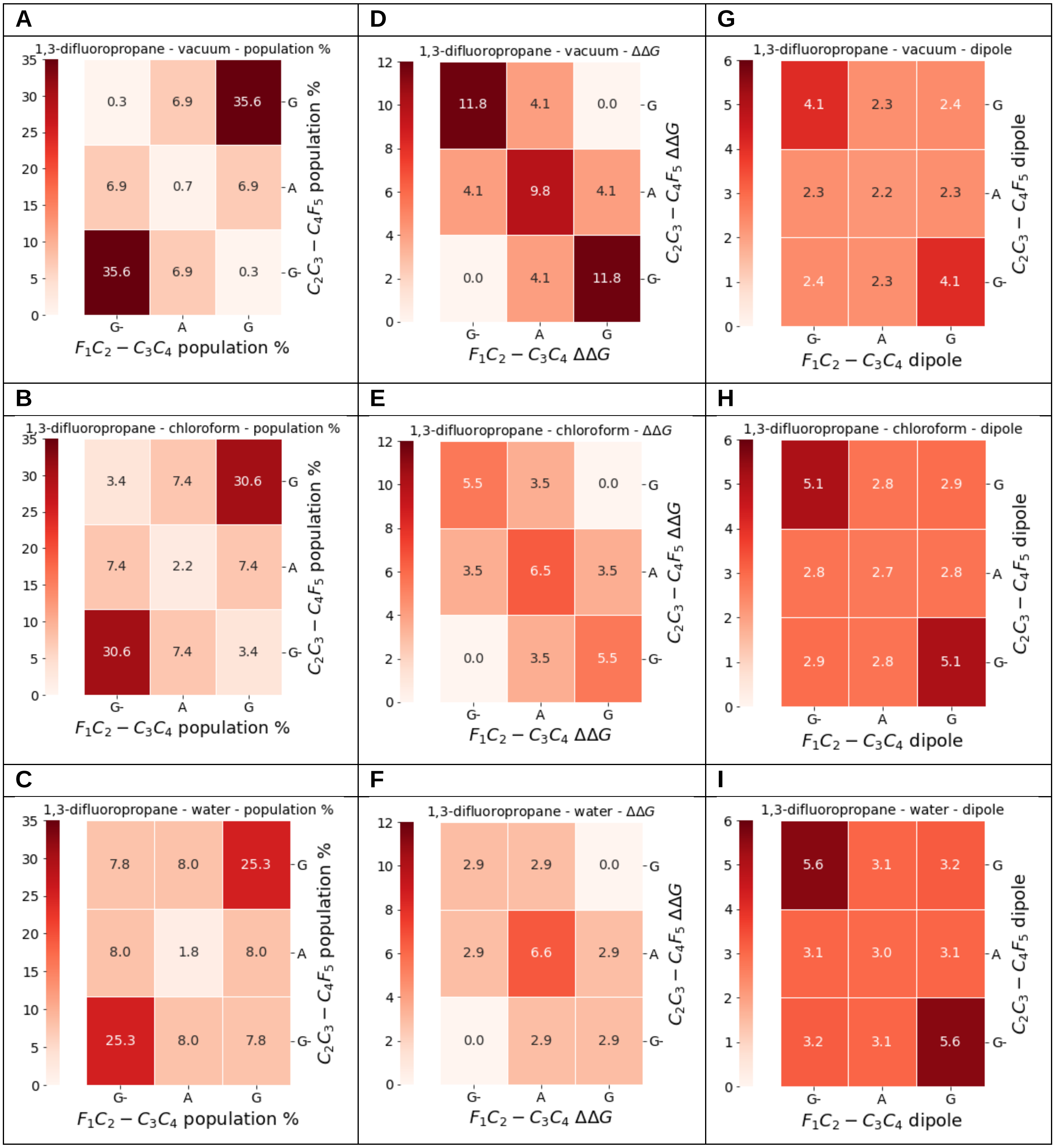

1.9.2 Chart S2: pentane

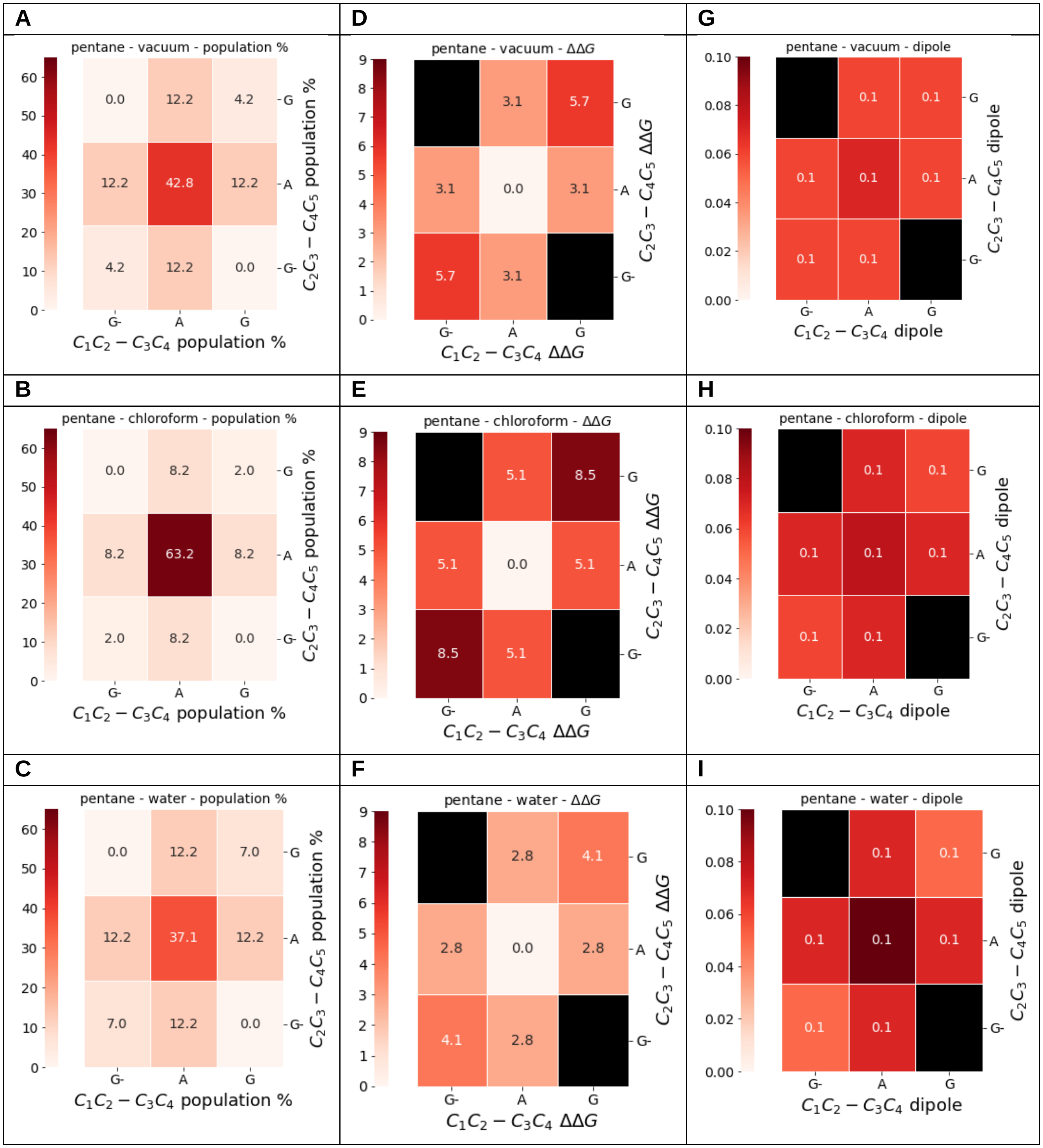

1.9.3 Chart S3: *anti*-2,4-difluoropentane (12)

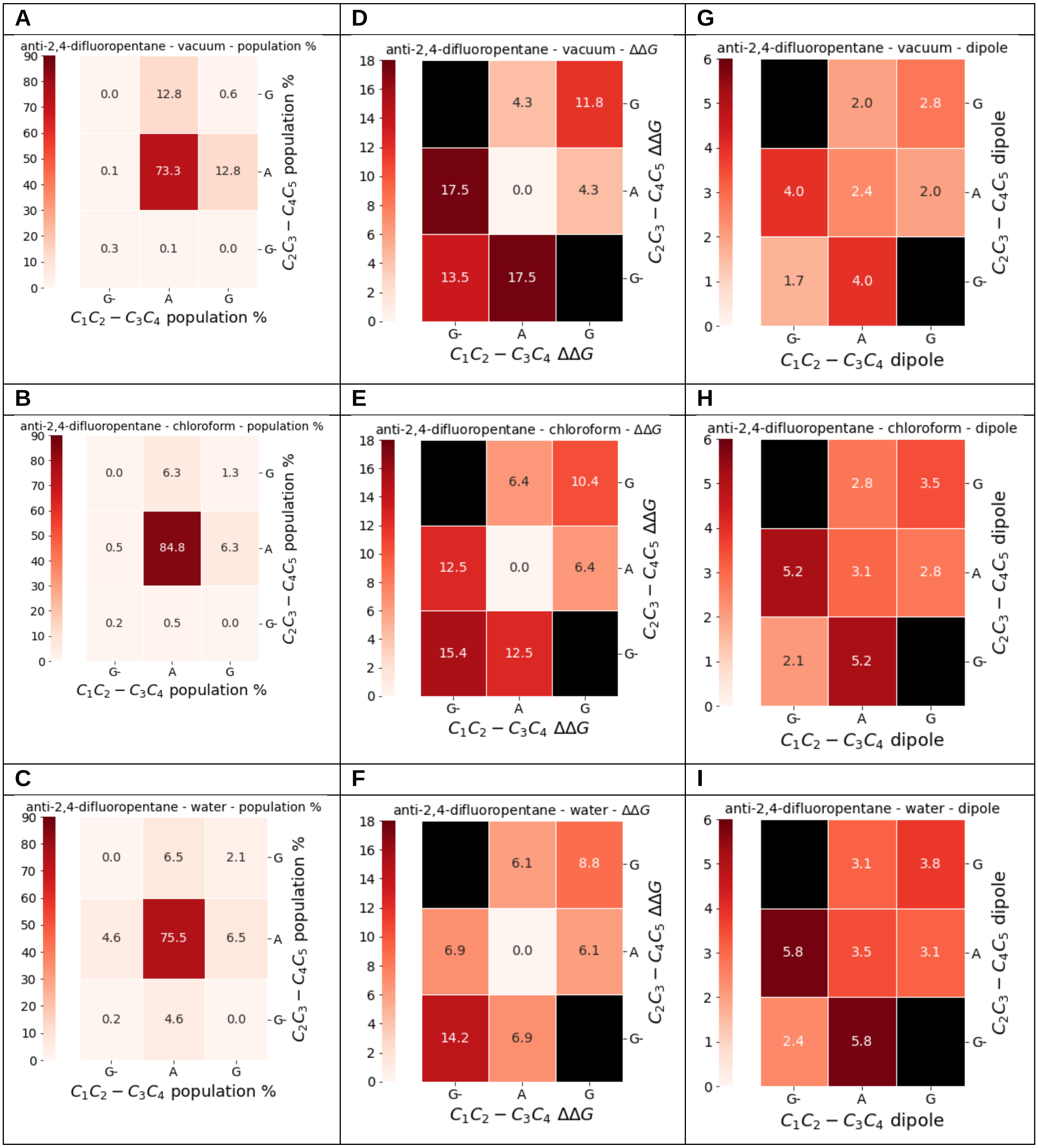

1.9.4 Chart S4: *syn*-2,4-difluoropentane (13)

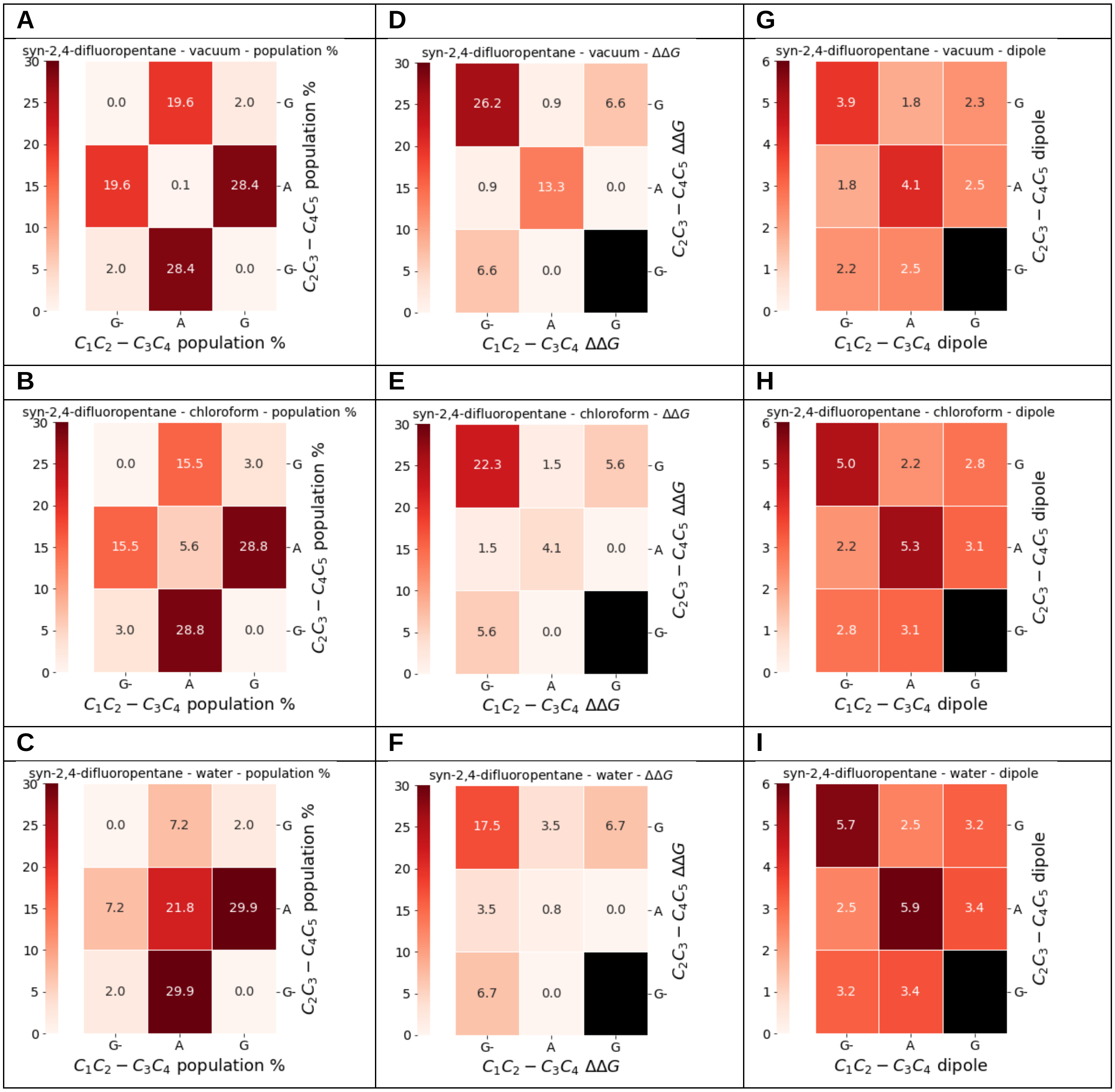

1.9.5 Chart S5: heptane (all dihedrals)

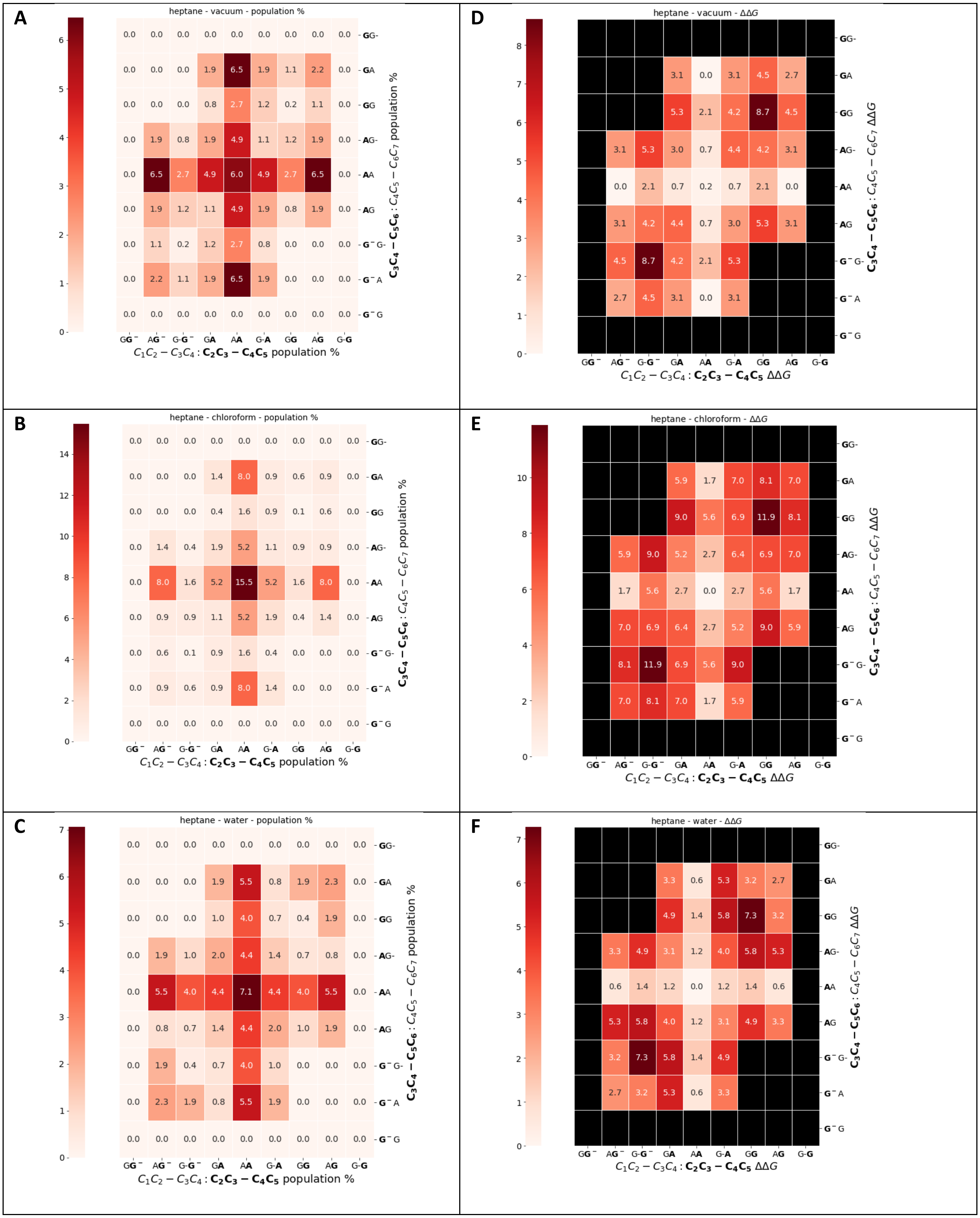

1.9.6 Chart S5 (cont'd): heptane (all dihedrals)

G

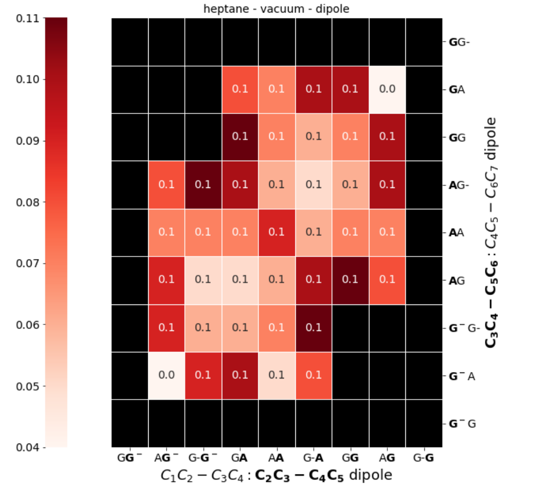

H

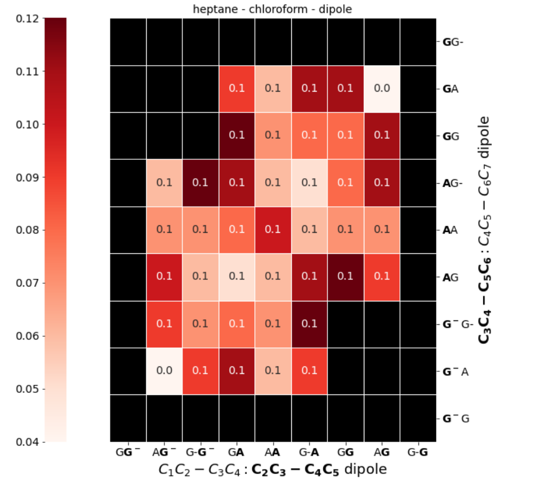

I

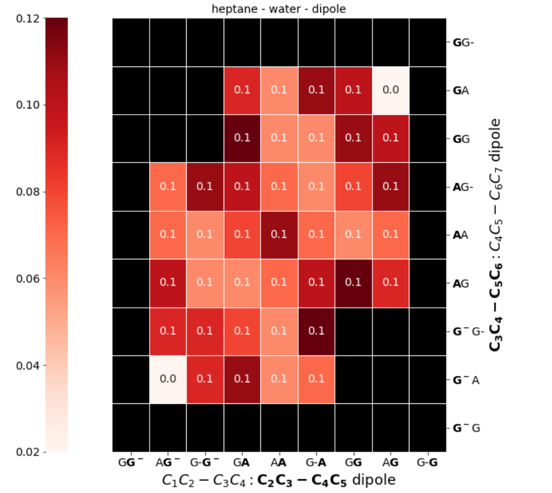

1.9.7 Chart S6: *anti*-3,5-difluoroheptane (14) (all dihedrals)

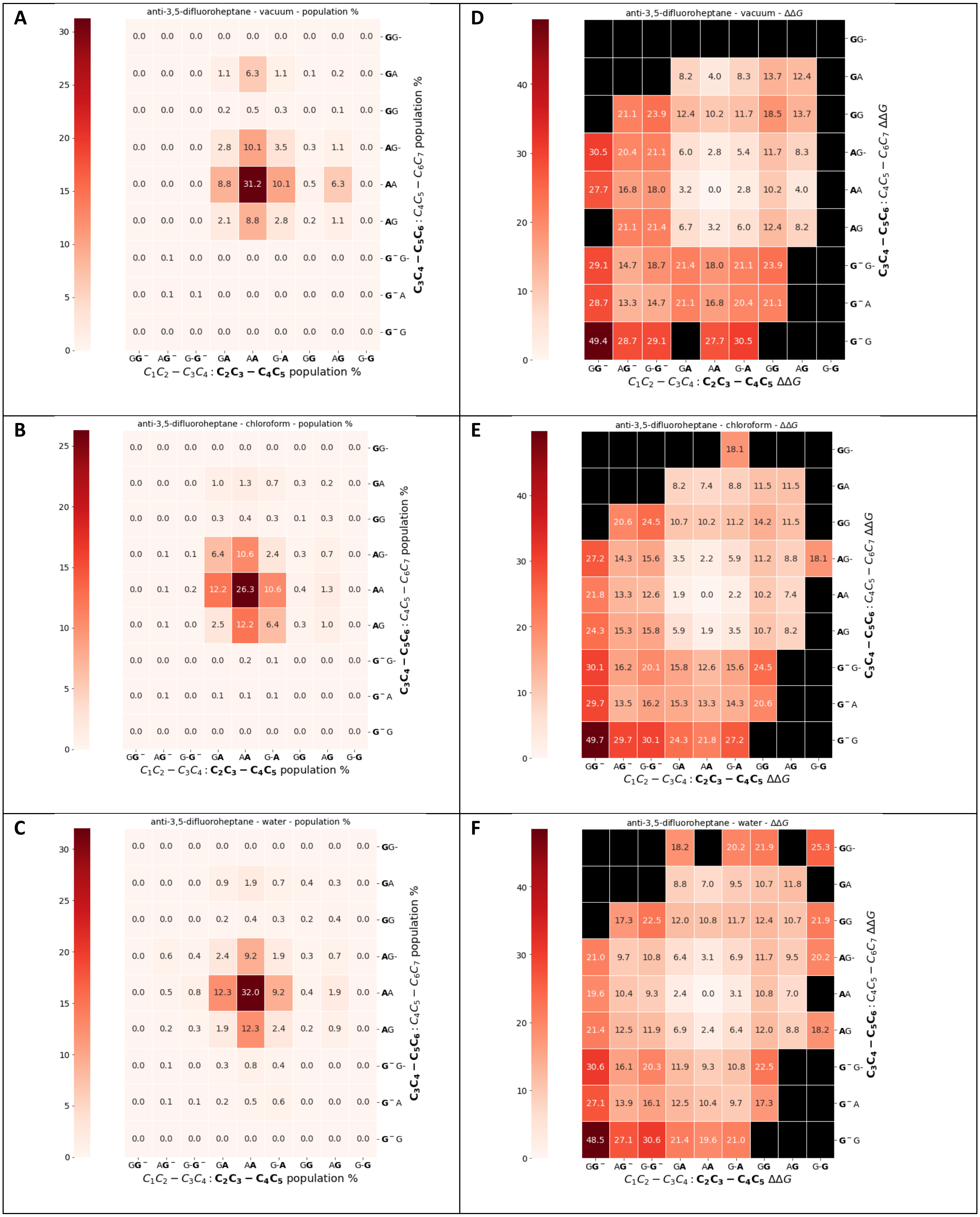

1.9.8 Chart S6 (cont'd): *anti*-3,5-difluoroheptane (14) (all dihedrals)

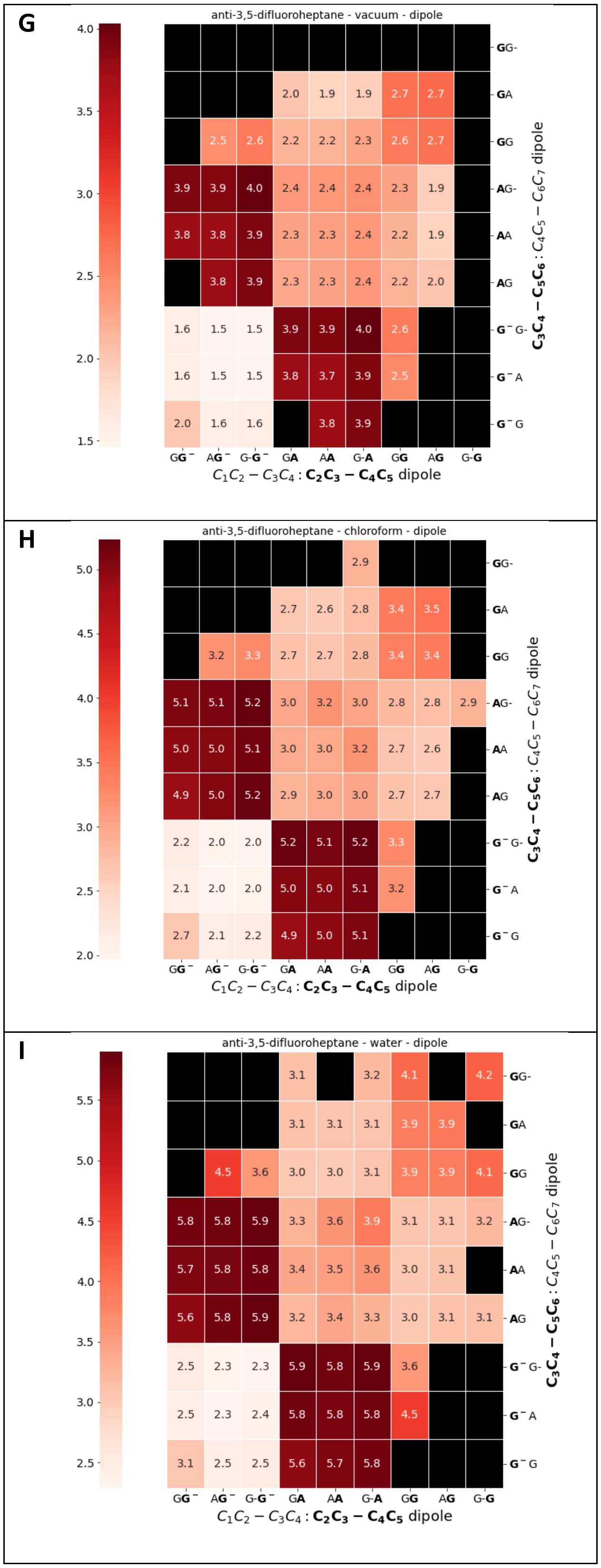

1.9.9 Chart S7: *syn*-3,5-difluoroheptane (15) (all dihedrals)

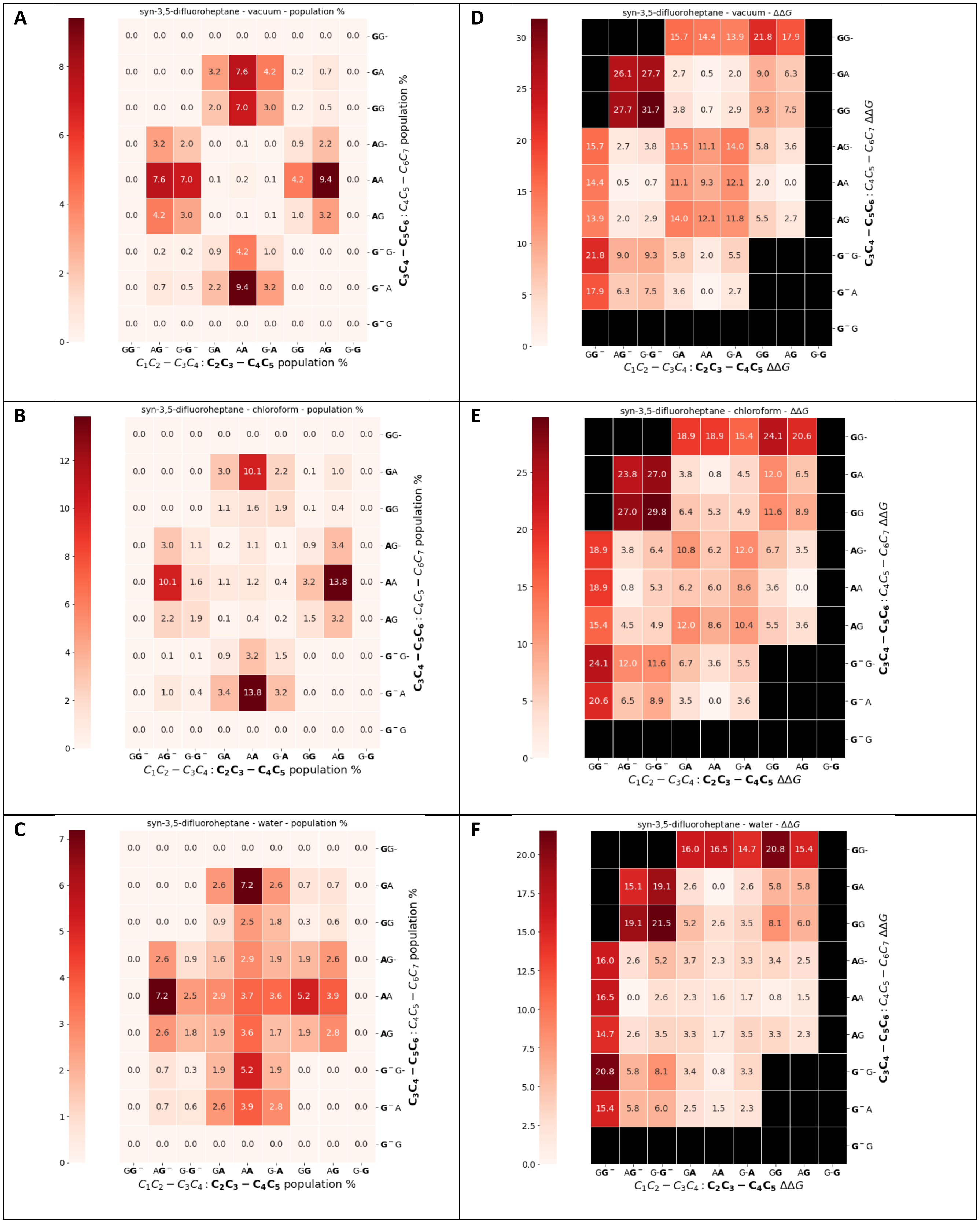

1.9.10 Chart S7 (cont'd): *syn*-3,5-difluoroheptane (15) (all dihedrals)

G

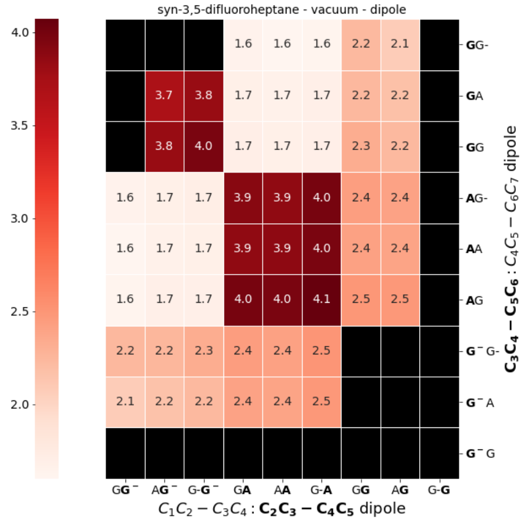

H

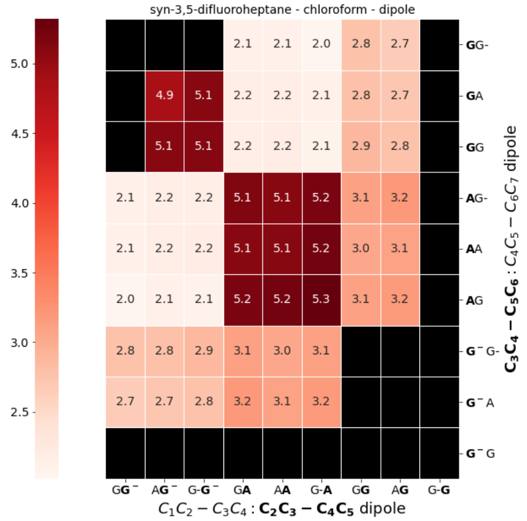

I

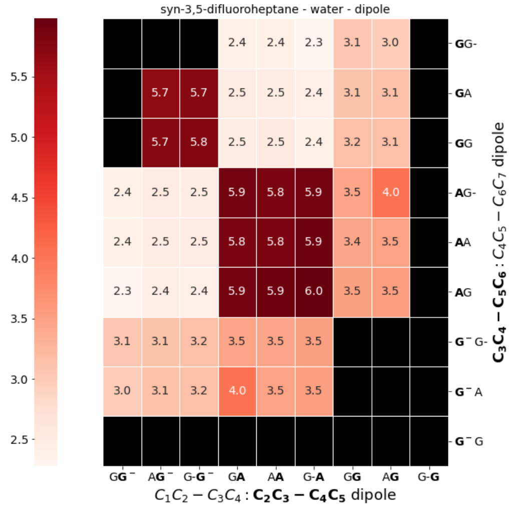

1.9.11 Figure S5: conversion from 9 × 9 to a 3 × 3 grid

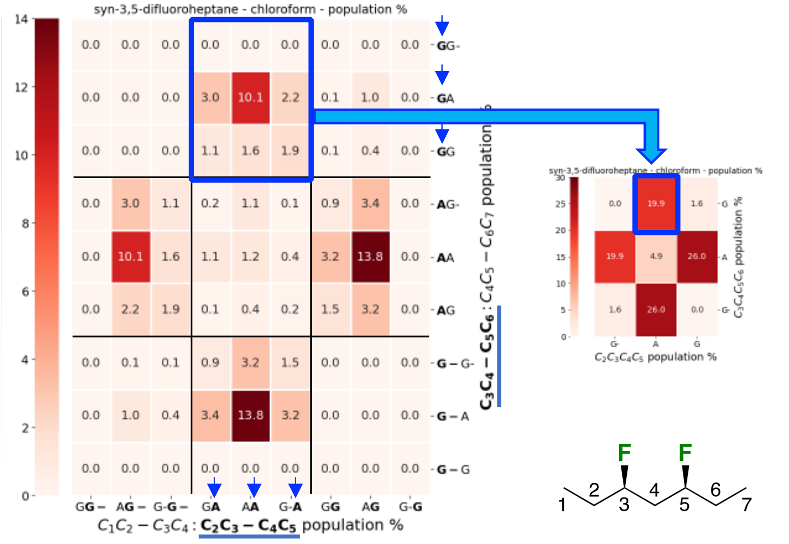

- Each box of 9 values refers to 9 conformations all having the same dihedral angles of the central bonds (here the *AG* conformations)
- The individual populations are summated to yield one value in the condensed 3 x 3 grid
- For the energies: the weighted sum is taken

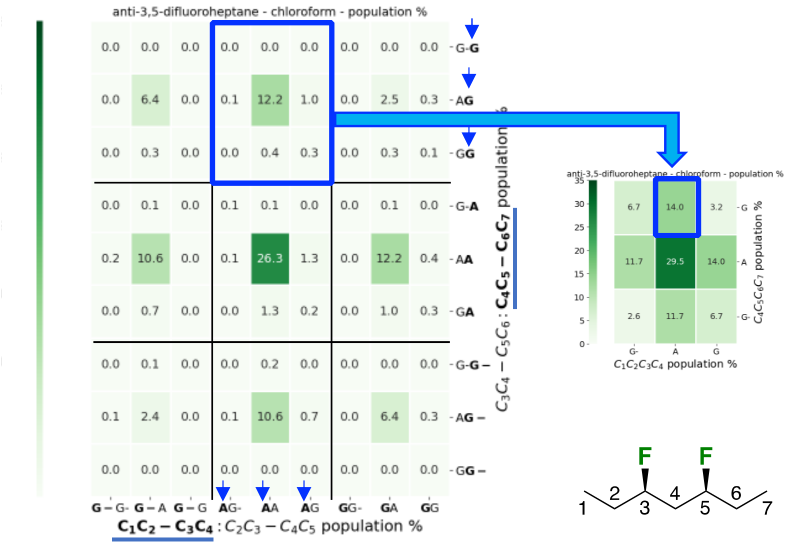

For the outer dihedrals: same principle, now starting from the 9 x 9 grid organised so each box of 9 values refers to 9 conformations all having the same dihedral angles of the outer bonds (here the *AG* conformations)

1.9.12 Chart S8: heptane (inner dihedrals)

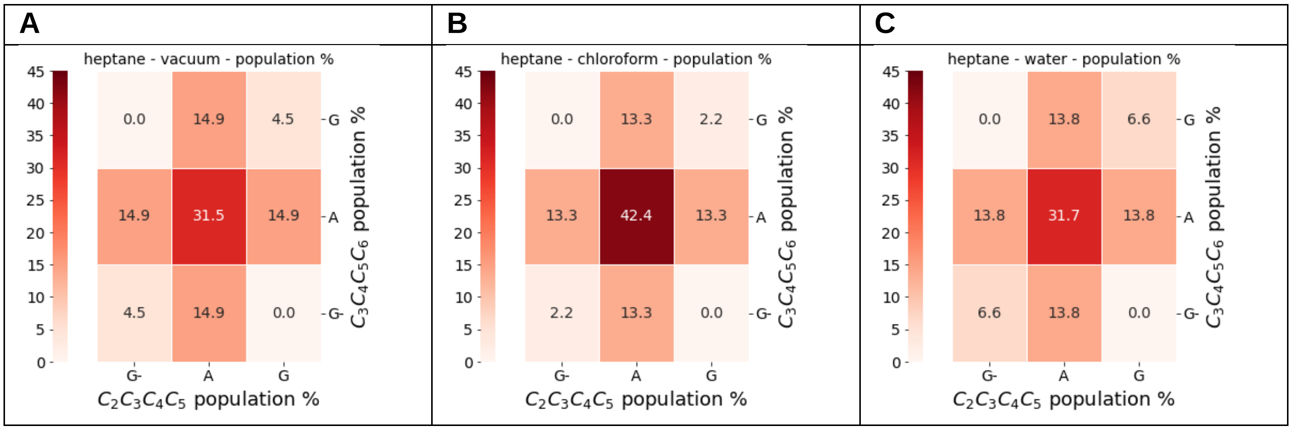

1.9.13 Chart S9: *anti*-3,5-difluoroheptane (14) (inner dihedrals)

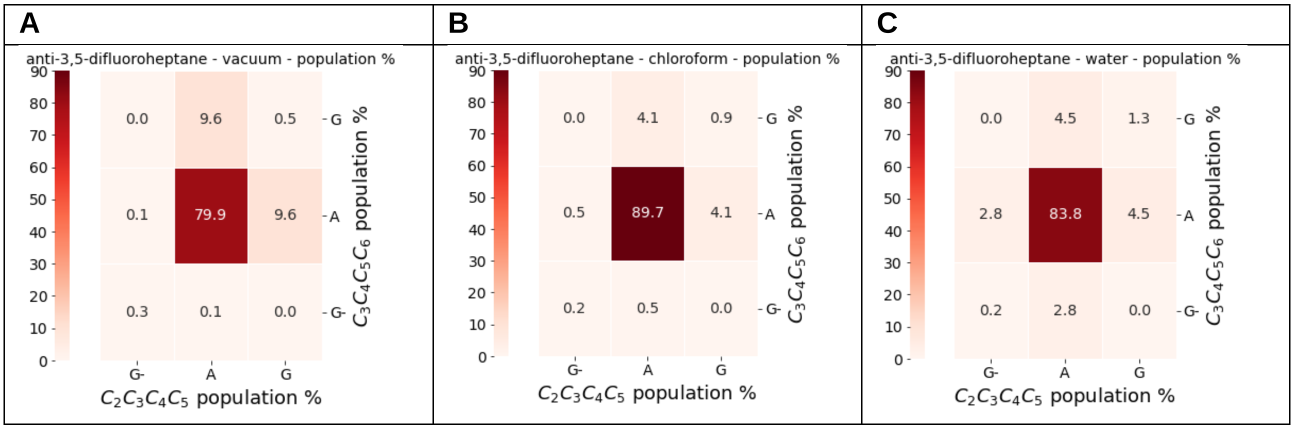

1.9.14 Chart S10: *syn*-3,5-difluoroheptane (15) (inner dihedrals)

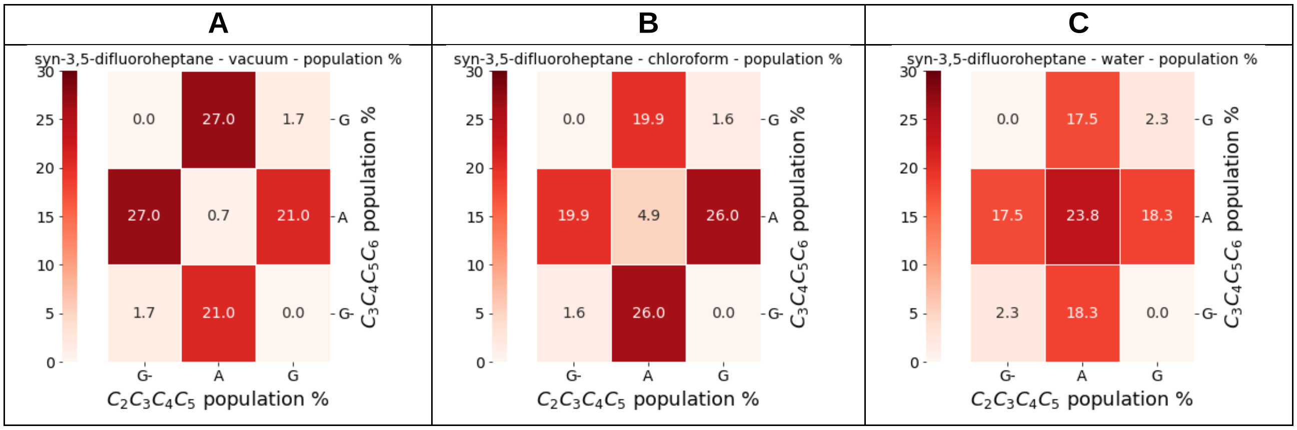

1.9.15 Chart S11: *anti*-3,5-difluoroheptane (14) (outer dihedrals, population).

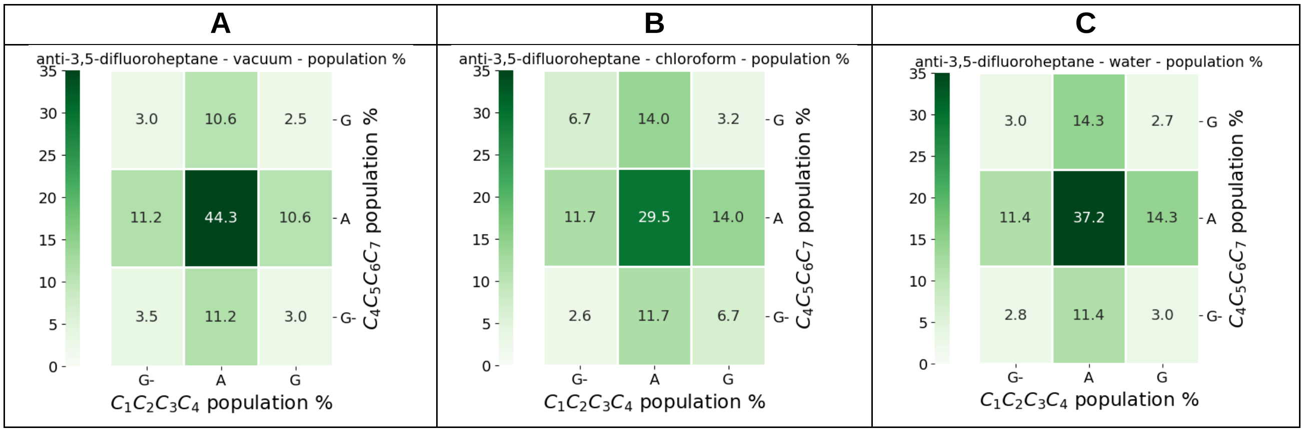

1.9.16 Chart S12: *syn*-3,5-difluoroheptane (15) (outer dihedrals, population).

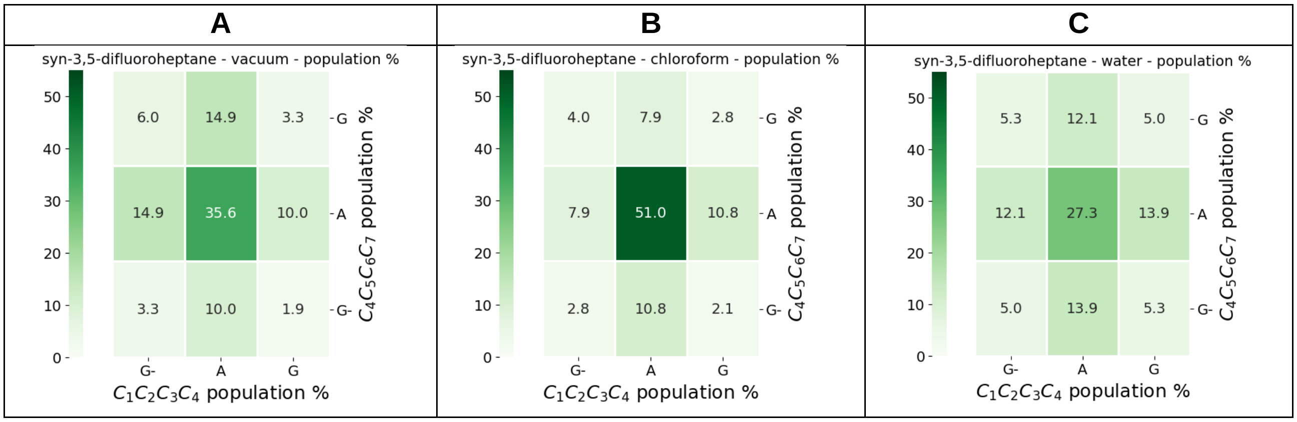

2 Synthesis of (±)-*anti* and *syn*-2,4-difluoropentane (12 and 13)

A mixture of *syn*- and *anti*-2,4-difluoropentane has been synthesised before as byproducts from the reaction of 1-iodopentane with hexachloromelamine or with NBS, although incompletely and inconclusively characterised.[1] The starting fluorohydrins **(±)-*anti*-SI-1 and (±)-*syn*-SI-2** were prepared according to the general route as previously described.[2]

2.1 General conditions

All reaction vessels were flame dried under vacuum and cooled under nitrogen prior to use. All water sensitive experiments were carried out under nitrogen or argon atmosphere, using dry solvents. CH_2Cl_2, Et_3N and EtOAc were distilled from CaH_2; THF, Benzene and Et_2O from Na/benzophenone. MeCN was dried over molecular sieves. PE = light petroleum ether. All reactions were monitored by TLC (Kiesgel 60 F_{254} MERCK Art. 5735 aluminium sheet).

Column chromatography was performed on silica gel (60 Å). Particle size 35–70 µm, or 40–63 µm. Preparative HPLC was carried out using Biorad Bio-Sil D 90-10 columns (250 × 22 mm at 15–20 mL min^{-1} and 250 × 10 mm at 5 mL min^{-1}).

NMR spectra were recorded on a BRUKER AV300 at 300 MHz (^{1}H) and 75 MHz (^{13}C), or on a BRUKER AV400 at 400 MHz (^{1}H) and 100 MHz (^{13}C) using the residual solvent as the internal standard. The coupling constants (*J*) are expressed in Hertz and the chemical shift in ppm. In the assignment of spectra, the an „*“ indicates that there is an ambiguity in the assignment between two or more atoms. IR spectra were recorded on a THERMO MATSON Fourier Transform spectrometer. The wave numbers (υ) are given in cm^{-1}. LRMS spectra were accomplished with ThermoQuest Trace MS, single quadrupol GC. This instrument was used for electron ionisation (EI) and chemical ionisation (CI) spectra. HRMS spectra were recorded on a VG Analytical 70-250-SE normal geometry, double focusing. This apparatus was used for all the HRMS. Optical rotations were recorded on an Optical Activity Polaar 2001 polarimeter at 589nm. Melting points were recorded on a Gallenkamp electrothermal melting point apparatus.

2.2 General procedure for the fluorination of fluorohydrins 12 and 13

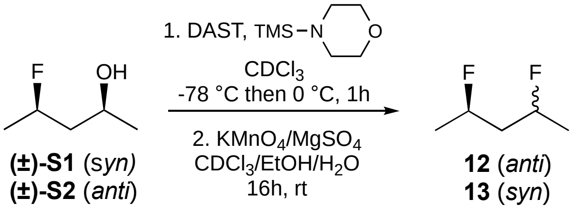

To a solution of DAST (3.3 equiv.) in $CDCl_3$ (0.4 mL/mmol of DAST) was slowly added *N*-(trimethylsilyl)morpholine (3.4 equiv.) at -78 °C under argon. The resulting solution was stirred at room temperature for 2.5 hours. The reaction mixture was further cooled to -78 °C and a solution of **(±)-S1** or **(±)-S2** (1 equiv.) in $CDCl_3$ (1.3 mL/mmol of fluorohydrin) was added dropwise. The resulting solution was stirred for 1.25 hours at 0 °C and then poured into a saturated aqueous $NaHCO_3$ solution (2.7 mL/mmol). After 15 min, water (1.4 mL/mmol) was added, the organic layer was separated and the aqueous layer was extracted with $CDCl_3$ (0.9 mL/mmol). The combined organic layers were washed with 1.0 M HCl (2.0 mL/mmol), then with brine (2.0 mL/mmol) and dried over $MgSO_4$. The crude mixture was diluted in EtOH (5.4 mL/mmol) and a solution of $KMnO_4$ (1.2 equiv.) and $MgSO_4$ (1 equiv.) in water (2.0 mL/mmol) was

added at 0 °C. The resulting solution was stirred overnight at 8 °C and then quenched with a saturated aqueous $NaHSO_3$ solution. The organic layer was separated and the aqueous layer was extracted with $CDCl_3$ (0.9 mL/mmol). The combined organic layers were washed five times with brine (1.3 mL/mmol) and then dried over $MgSO_4$. The residue was filtered on silica gel (in Pasteur pipette) and rinsed with $CDCl_3$ (1.3 mL/mmol), resulting in a $CDCl_3$ solution of the impure 2,4-difluoropentane.

2.3 (±)-*anti*-2,4-difluoropentane (12)

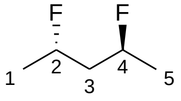

Starting from **(±)-SI1** (0.100 g, 0.94 mmol), using general procedure described above, **12** was obtained impure as colorless liquid and then submitted to NMR studies. **IR** (neat) 2978(m), 2938(w), 1458(w), 1382(m), 1130(s),1005(m), 790(s) cm^{-1}. **^{1}H NMR** (500 MHz, $CDCl_3$) δ 4.97–4.81 (m, a coupling constant of 49.4 Hz can be observed which disappears upon F decoupling, 2H, **H-2**, **H-4**), 1.90–1.73 (m, 2H, **H-3**), 1.37 (dd, $^{3}J_{H1/5\text{-}F}$ = 24.3 Hz, $^{3}J_{H1\text{-}H2}$ = 6.2 Hz, 6H, **H-1**, **H-5**) ppm. **^{13}C{^{1}H} NMR** (126 MHz, $CDCl_3$) δ 87.3 (dd, J = 164.3 Hz, J = 3.3 Hz, 2C, **C-2**, **C-4**), 44.6 (t, J = 20.5 Hz, **C-3**), 21.4 (d, J = 22.4 Hz, 2C, **C-1**, **C-5**) ppm. **^{19}F NMR** (470 MHz, $CDCl_3$) δ -175.8 – -175.4 (m, 2F, **F-2**, **F-4**) ppm. **^{19}F(^{1}H} NMR** (470 MHz, $CDCl_3$) δ -175.6 (s, 2F, **F-2**, **F-4**) ppm.

2.4 *syn*-2,4-difluoropentane (13)

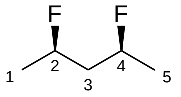

Starting from **(±)-S2** (0.100 g, 0.94 mmol), using general procedure described above, **13** was obtained impure as colorless liquid and then submitted to NMR studies. **IR** (neat) 2975(m), 2930(w), 1456(w), 1380(m), 1128(s), 1008(m), 788(s) cm^{-1}. **^{1}H NMR** (500 MHz, $CDCl_3$) δ 4.92–4.75 (m, a coupling constant of 48.1 Hz can be observed which disappears upon F decoupling, 2H, **H-2**, **H-4**), 2.14 (tdt, $^{3}J_{H3'\text{-}F}$ = 17.0 Hz, $^{2}J_{H3'\text{-}H3}$ = 14.2 Hz, $^{3}J_{H3'\text{-}H2+4}$ = 7.1 Hz, 1H, **H-3'**), 1.78 (tdt, $^{3}J_{H3\text{-}F}$ = 24.9 Hz, $^{2}J_{H3\text{-}H3'}$ = 14.3 Hz, $^{3}J_{H3\text{-}H2+4}$ = 5.2 Hz, 1H, **H-3**), 1.40 (dd, $^{3}J_{(H1/5\text{-}F)}$ = 24.9 Hz, $^{3}J_{H1\text{-}H2 \text{ and } H5\text{-}H4}$ = 6.2 Hz, 6H, **H-1**, **H-5**) ppm. **^{13}C{^{1}H} NMR** (126 MHz, $CDCl_3$) δ 87.8 (dd, J = 164.5 Hz, J = 6.2 Hz, 2C, **C-2**, **C-4**), 43.5 (t, J = 20.7 Hz, **C-3**), 20.9 (d, J = 22.9 Hz, 2C, **C-1**, **C-5**) ppm. **^{19}F NMR** (470 MHz, $CDCl_3$) δ -173.6 – -173.3 (m, 2F, **F-2**, **F-4**) ppm. **^{19}F(^{1}H} NMR** (470 MHz, $CDCl_3$) δ -173.5 (s, 2F, **F-2** + **F-4**) ppm.

3 Synthesis of (±)-*anti*- and *meso*-3,5-difluoroheptane (14 and 15)

3.1 General scheme

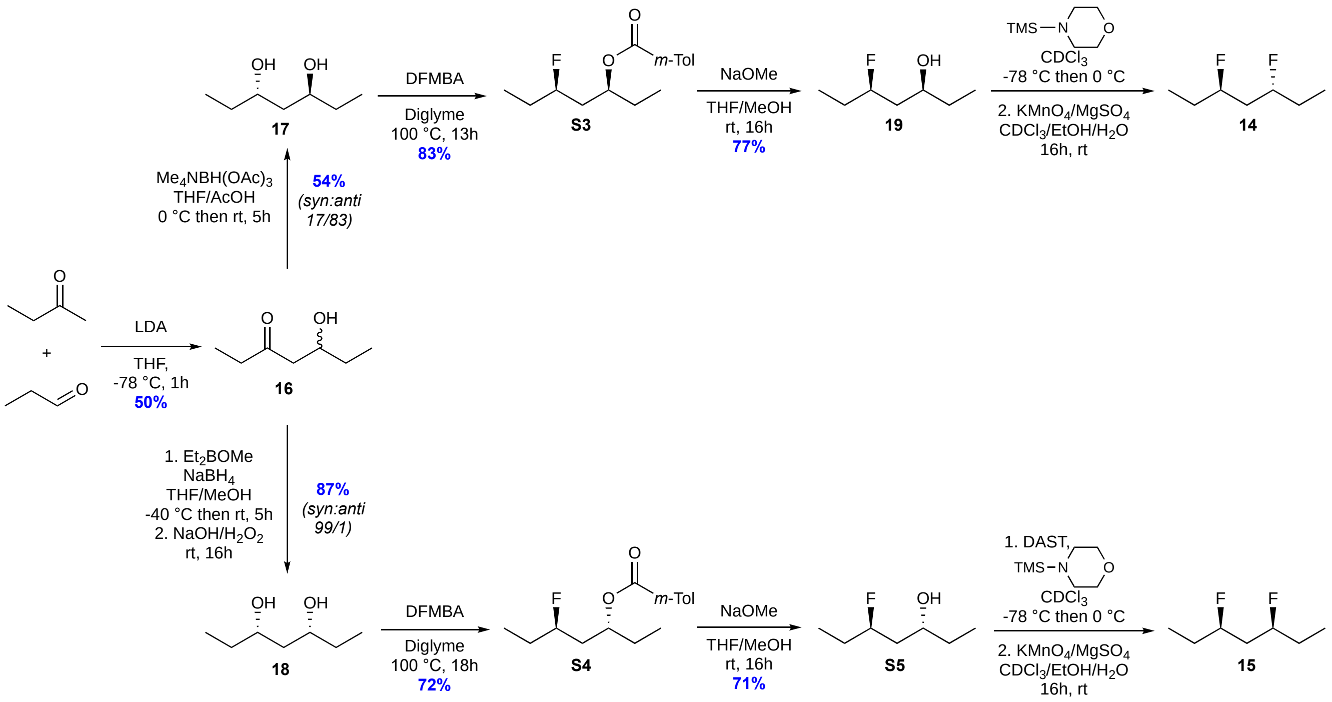

3.2 Synthesis of 3-hydroxyheptan-5-one (16)[3]

At 0 °C, a solution of *n*-butyllithium (2.5 M in hexane, 39 mL, 97.1 mmol, 1 equiv.) was added dropwise to a solution of diisopropylamine (21 mL, 145.6 mmol, 1.5 equiv.) in THF (90 mL) under argon. After 30 minutes at 0 °C, the temperature was lowered to -78 °C and a solution of 2-butanone (8.7 mL, 97.1 mmol, 1 equiv.) in THF (18 mL) was added dropwise. The mixture was stirred at -78 °C for 1 hour before a solution of propanal (7.0 mL, 97.1 mmol, 1 equiv.) in THF (14 mL) was added dropwise and the resulting mixture was kept at the same temperature for 40 minutes. An aqueous saturated NH_4Cl solution (100 mL) was added to the reaction mixture which was allowed to warm to room temperature and then extracted three times with diethyl ether (3x150 mL). The combined organic phases were washed twice with brine, dried over $MgSO_4$ and concentrated. The residue was purified by flash chromatography (gradient 0 to 10% of ethyl acetate in hexane) affording **16** as colorless oil (6.27 g, 48.2 mmol, 50%). **IR** (neat) 3040(br), 2967(s), 2937(m), 1704(s), 1411(w), 1117(m), 970(m) cm^{-1}. **^{1}H NMR** (400 MHz, $CDCl_3$): δ 4.01–3.93 (m, 1H, **H-5**), 3.06 (d, *J* = 3.4 Hz, 1H, **OH**), 2.64–2.44 (m, 4H, **H-2**, **H-4**), 1.58–1.39 (m, 2H, **H-6**), 1.06 (t, *J* = 7.3 Hz, 3H, **H-1**) 0.95 (t, *J*=7.5 Hz, 3H, **H-7**) ppm. **^{13}C{^{1}H}NMR** (101 MHz, $CDCl_3$): δ 212.8 (**C-3**), 69.0 (**C-5**) 48.1 (**C-4**) 36.8 (**C-2**) 29.3 (**C-6**), 9.8 (**C-7**), 7.5 (**C-1**) ppm. **MS(**ESI+) *m/z* 153 [M+Na]$^+$. NMR data corresponded with published data.[3]

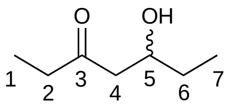

3.3 *Anti* reduction: synthesis of (±)-*anti*-heptane-3,5-diol (17)

A solution of $Me_4NBH(OAc)_3$ (10.6 g, 40.3 mmol, 2.1 equiv.) in a mixture THF/AcOH (50:50 mL) was stirred under argon at room temperature for 30 minutes. Then a solution of **16** (2.50 g, 19.2 mmol, 1 equiv.) in THF (70 mL) was slowly added and the resulting mixture was stirred under argon at room temperature for 5 hours. The reaction was quenched with an aqueous solution of Rochelle's salt (20%, 90 mL) and stirred at room temperature for 20 minutes. Subsequently, water (100 mL) was added to the reaction and the mixture was extracted with CH_2Cl_2 (3x150 mL). The organic extracts were combined and neutralised by adding water (150 mL) and solid $NaHCO_3$ until pH > 7. Then the aqueous layer was separated and the organic layer was washed with brine (150 mL), dried over $MgSO_4$ and concentrated *in vacuo*. The crude residue was purified by flash chromatography (slow gradient from 0 to 15% of ethyl acetate in hexane) providing **17** as a white solid (1.37 g, 10.4 mmol, 54%). **Mp** 38–40 °C. **IR** (neat) 3312(br), 2962(m), 2934(m), 1461(m), 1114(w), 976(w) cm^{-1}. **^{1}H NMR** (400 MHz, $CDCl_3$): δ 3.88–3.81 (m, 2H, **H-3**, **H-5**), 2.88 (brs, 2H, **OH**), 1.63–1.42 (m, 6H, **H-2, H-4**, **H-6**), 0.94 (t, J = 7.5 Hz, 6H, **H-1**, **H-7**) ppm. **^{13}C{^{1}H} NMR** (101 MHz, $CDCl_3$): δ 70.6 (2C, **C-3**, **C-5**), 41.4 (**C-4**), 30.2 (2C, **C-2**, **C-6**), 10.1 (2C, **C-1**, **C-7**) ppm. **MS(**ESI+) *m/z* 155 [M+Na]$^+$. **HRMS**(ESI+) calcd for $C_7H_{16}O_2Na$ [M+Na]$^+$ 155.1043, found 155.1043. 1H NMR data corresponded with published data.[4]

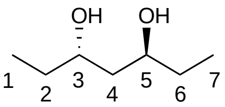

3.4 *Syn* reduction: synthesis of *syn*-heptane-3,5-diol (18)

To a solution of **16** (2.90 g, 22.3 mmol, 1 equiv.) in a mixture THF/MeOH (150:40 mL) was slowly added Et_2BOMe (5.85 mL, 44.6 mmol, 2 equiv.) at -30 °C, under argon. The solution was stirred at -10 °C for 45 minutes before the addition of $NaBH_4$ (1.69 g, 44.6 mmol, 2 equiv.). After 5 hours at room temperature, the reaction was quenched by addition of acetic acid (30 mL) and stirred for 20 minutes. Then, an aqueous saturated $NaHCO_3$ solution was added and the mixture was extracted using CH_2Cl_2 (3x150 mL). The organic phases were combined, dried over $MgSO_4$ and concentrated *in vacuo*. The residue was treated with a 5M NaOH (200 mL) and H_2O_2 (70 mL) and the solution was stirred at room temperature overnight. Water (100 mL) was added and the aqueous phase was extracted into CH_2Cl_2 (3x250 mL). The organic phases were combined, dried over $MgSO_4$ and concentrated *in vacuo*. The residue was purified by flash chromatography (slow gradient from 0 to 15% of ethyl acetate in hexane) affording **18** as colorless oil (2.56 g, 19.4 mmol, 87%). **IR** (neat) 3316(br), 2963(m), 2935(m), 1460(m), 1150(w), 984(w) cm^{-1}. **^{1}H NMR** (400 MHz, $CDCl_3$): δ 3.81–3.75 (m, 2H, **H-3**, **H-5**), 3.34 (d, J = 2.4 Hz, 2H, **OH**), 1.64–1.38 (m, 6H, **H-2**, **H-4**, **H-6**), 0.93 (t, J = 7.5 Hz, 6H, **H-1**, **H-7**) ppm. **^{13}C{^{1}H} NMR** (101 MHz, $CDCl_3$): δ 74.4 (2C, **C-3**, **C-5**), 41.7 (**C-4**), 30.9 (2C, **C-2**, **C-6**), 9.6 (2C, **C-1**, **C-7**) ppm. **MS(**ESI+) *m/z* 155 [M+Na]$^+$. **HRMS**(ESI+) calcd for $C_7H_{16}O_2Na$ [M+Na]$^+$ 155.1043, found 155.1044.

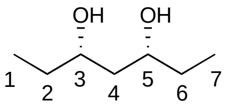

Note : Assignment of relative configuration of diastereoisomers:

1) It was reported that, for a pair of diastereoisomers, the signals of the α-hydroxy carbons appear at higher field for the *anti*-diols than for the *syn*-diols.[5-8] For **18** these signals appear at δ = 74.4 ppm and for **17**, at δ = 70.6 ppm.
2) The assignment of relative configuration of both 1,3-diols **17 and 18** was achieved using "^{13}C acetonide method" reported by Rychnovsky and co-workers.[9,10] Therefore, the corresponding *anti* and *syn*-1,3-diol acetonides were synthesised from **17** and **18**, respectively. *syn*-1,3-Diol acetonide

prefers chair conformations, where one of the acetal methyl groups is axial and the other methyl group is equatorial. The axial methyl group has a ^{13}C chemical shift of δ = 19.8 ppm, while the equatorial methyl has a chemical shift of δ = 30.3 ppm. *anti*-1,3-Diol acetonide adopts *twist*-boat conformations, where the two methyl groups are in nearly identical environments and thus both have ^{13}C chemical shifts of δ = 24.5 ppm. The quaternary C-acetal ^{13}C chemical shift was also indicative of the change in conformation where <u>syn</u> and *anti*-acetonides had ^{13}C chemical shifts of 98.2 and 99.8 ppm, respectively.

3.5 Synthesis of (±)-*anti* and (±)-*syn*-3-(3'-methylbenzoyloxy)-5-fluoroheptane (*anti*-SI8 and *syn*-SI9)

3.5.1 General procedure for the mono-deoxyfluorination of diols using DFMBA in diglyme

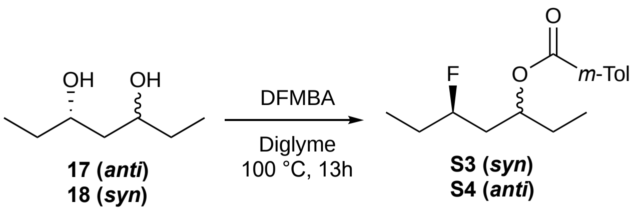

To diol **17** or **18** (1 equiv.) was added a solution of *N,N*-diethyl-α,α-difluoro(*meta*-methylbenzyl)amine (DFMBA, 2 equiv.)[11] in diglyme (0.4 mL/mmol of diol) at room temperature under argon. The reaction mixture was stirred and heated at 100 °C for 18 hours (oil bath) under argon. After cooling, the reaction was quenched with an aqueous saturated solution of $NaHCO_3$ (1.2 mL/mmol) and stirred for 20 minutes. Subsequently, water (1.2 mL/mmol) was added to the reaction and the mixture was extracted with di-ethyl ether three times (2.1 mL/mmol). The combined organic phases were washed with brine (3.5 mL/mmol), dried over $MgSO_4$ and concentrated *in vacuo*. The crude mixture was purified by flash chro-matography (gradient 0 to 3% of ethyl acetate in hexane) and preparative HPLC (1.5% of ethyl acetate in hexane).

3.5.2 (±)-*syn*-3-(3'-methylbenzoyloxy)-5-fluoroheptane (S3)

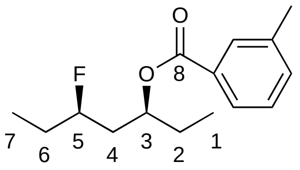

Starting from **17** (1.32 g, 9.98 mmol), using general procedure described above, **S3** was obtained as colorless oil (2.09 g, 8.27 mmol, 83%). **IR** (neat) 2969(m), 2881(w), 1713(s), 1462(w), 1274(s), 1198(s), 1103(m), 744(s) cm^{-1}. **^{1}H NMR** (400 MHz, $CDCl_3$) δ 7.88–7.84 (m, 2H, **H_{Ar}**), 7.40–7.31 (m, 2H, **H_{Ar}**), 5.26–5.20 (m, 1H, **H-3**), 4.69–4.51 (m, a coupling constant of 48.9 Hz can be observed,1H, **H-5**), 2.42 (s, 3H, **$C\underline{H}_{3\text{-Ar}}$**), 2.14 (ddt, *J* = 16.6, 14.8, 7.4 Hz, 1H, **H-4'**), 1.91 (ddt, *J* = 27.9, 14.8, 4.8 Hz 1H, **H-4**), 1.83–1.60 (m, 4H, **H-6**, **H-2**), 0.99 (t, *J* = 7.5 Hz, 6H, **H-7** or **H-1**), 0.98 (t, *J* = 7.5 Hz, 6H, **H-1** or **H-7**) ppm. **^{13}C{^{1}H} NMR** (101 MHz, $CDCl_3$) δ 166.3 (**C-8**), 138.1 (**C_{qAr}**), 133.6 (**$\underline{C}H_{Ar}$**), 130.5 (**C_{qAr}**), 130.1 (**$\underline{C}H_{Ar}$**), 128.2 (**$\underline{C}H_{Ar}$**), 126.7 (**$\underline{C}H_{Ar}$**), 92.7 (d, ^1J =168.0 Hz, **C-5**), 73.1 (d, ^3J = 5.1 Hz, **C-3**), 38.5 (d, ^2J = 20.5 Hz, **C-4**), 28.2 (d, ^2J = 21.3 Hz, **C-6**), 27.2 (**C-2**), 21.3 (**$\underline{C}H_{3\text{-Ar}}$**), 9.6 (**C-1**), 9.2 (d, ^3J = 5.9 Hz, **C-7**) ppm. **^{19}F NMR** (376 MHz, $CDCl_3$) δ -181.8 – -181.4 (m) ppm. **^{19}F{^{1}H} NMR** (376 MHz, $CDCl_3$) δ -181.6 (s, 1F) ppm. **MS** (ESI+) *m/z* 253 [M+H]$^+$. **HRMS**(ESI+) calcd for $C_{15}H_{21}FO_2Na$ [M+Na]$^+$ 275.1418, found 275.1423.

3.5.3 (±)-*anti-3*-(3'-methylbenzoyloxy)-5-fluoroheptane (S4)

Starting from **18** (2.20 g, 16.6 mmol), using general procedure described above, **S4** was obtained as colorless oil (3.01 g, 11.9 mmol, 72%). **IR** (neat) 2969(m), 2937(w), 1713(s), 1462(w), 1273(s), 1198(s), 1105(m), 744(s) cm^{-1}. **^{1}H NMR** (400 MHz, $CDCl_3$) δ 7.89–7.83 (m, 2H, **H$_{Ar}$**), 7.41–7.30 (m, 2H, **H$_{Ar}$**), 5.34–5.27 (m, 1H, **H-3**), 4.63–4.44 (m, a coupling constant of 49.4 Hz can be observed,1H, **H-5**), 2.42 (s, 3H, **C<u>H</u>$_{3\text{-}Ar}$**), 2.00–1.81 (m, 2H, **H-4**), 1.81–1.72 (m, 2H, **H-2**), 1.73–1.53 (m, 2H, **H-6**), 0.98 (t, *J* = 7.5 Hz, 6H, **H-1**, **H-7**) ppm. **^{13}C{^{1}H} NMR** (101 MHz, $CDCl_3$) δ 166.3 (**C-8**), 138.1 (**C$_{qAr}$**), 133.6 (**<u>C</u>H$_{Ar}$**), 130.5 (**C$_{qAr}$**), 130.1 (**<u>C</u>H$_{Ar}$**), 128.3 (**<u>C</u>H$_{Ar}$**), 126.7 (**<u>C</u>H$_{Ar}$**), 92.0 (d, ^1J =169.5 Hz, **C-5**), 72.7 (d, ^3J = 2.2 Hz, **C-3**), 39.2 (d, ^2J = 20.5 Hz, **C-4**), 28.5 (d, ^2J = 21.3 Hz, **C-6**), 27.7 (**C-2**), 21.3 (**<u>C</u>H$_{3\text{-}Ar}$**), 9.42 (**C-1**), 9.2 (d, ^3J = 5.1 Hz, **C-7**) ppm. **^{19}F NMR** (376 MHz, $CDCl_3$) δ -183.0 – -182.6 (m) ppm. **^{19}F{^{1}H} NMR** (376 MHz, $CDCl_3$) δ -182.8 (s, 1F) ppm. **MS** (ESI+) *m/z* 253 $[M+H]^+$. **HRMS**(ESI+) calcd for $C_{15}H_{21}FO_2Na$ $[M+Na]^+$ 275.1418, found 275.1414.

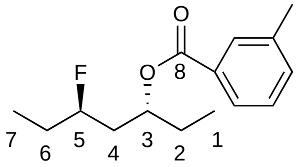

3.6 Synthesis of fluorohydrins 19 and 20

3.6.1 General procedure for the hydrolysis of S3 and S4

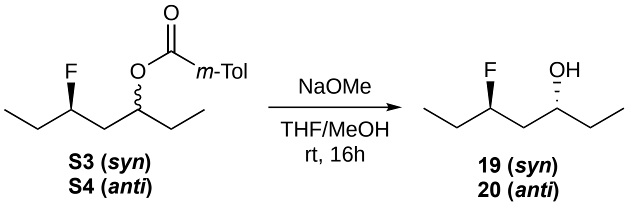

S3 (***syn***)
S4 (***anti***)
19 (***syn***)
20 (***anti***)

To a solution of **S3** or **S4** (1 equiv.) in dry THF (2.5 mL/mmol) was added a solution of sodium methoxide in methanol (25 wt. %, 2 equiv.) at 0 °C under argon. The reaction mixture was stirred at room temperature for 16 hours and then neutralised with Amberlite® CG-50. The resin was filtered off and rinsed with diethyl ether (4 mL/mmol). The filtrate was washed two times with an aqueous saturated solution of potassium carbonate (4 mL/mmol), then with brine (4 mL/mmol), dried over $MgSO_4$ and concentrated *in vacuo* (P>700 mbar). The crude mixture was purified by flash chromatography (gradient 0 to 15% of diethyl ether in pentane).

3.6.2 (±)-*syn*-5-fluoroheptan-3-ol (19)

Starting from **S3** (2.06 g, 8.18 mmol), using general procedure described above, **19** was obtained as colorless oil (0.845 g, 6.29 mmol, 77%). **IR** (neat) 3382(br), 2967(m), 2940(m), 2881(w), 1463(m), 1115(m), 969(s), 812(m) cm^{-1}. **^{1}H NMR** (500 MHz, $CDCl_3$) δ 4.65 (ddddd, *J* = 49.8, 8.8, 7.0, 4.9, 3.7 Hz, 1H, **H-5**), 3.80–3.74 (m, 1H, **H-3**), 2.09–2.05 (m, 1H, **OH**), 1.81 (tdd, *J* = 14.6, 8.9, 8.2 Hz, 1H, **H-4'**), 1.75–1.46 (m, 5H, **H-4**, **H-6**, **H-2**), 0.99 (t, *J* = 7.5 Hz, 3H, **H-7**), 0.96 (t, *J* = 7.5 Hz, 3H, **H-1**) ppm. **^{13}C{^{1}H} NMR** (126 MHz, $CDCl_3$) δ 95.7 (d, ^1J = 164.5 Hz, **C-5**), 71.6 (d, ^3J = 3.3 Hz, **C-3**), 41.4 (d, ^2J = 19.1 Hz, **C-4**), 30.1 (**C-2**), 28.5 (d, ^2J = 21.2 Hz, **C-6**), 9.7 (**C-1**), 9.2 (d, ^3J = 6.0 Hz, **C-7**) ppm. **^{19}F NMR** (471 MHz, $CDCl_3$) δ -181.2 – -180.9 (m) ppm. **^{19}F{^{1}H} NMR** (471 MHz, $CDCl_3$) δ -181.0 (s, 1F) ppm. **HRMS**(ESI+) calcd for $C_7H_{15}FONa$ $[M+Na]^+$ 157.0999, found 157.0999

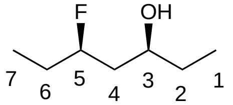

3.6.3 (±)-*anti*-5-fluoroheptan-3-ol (20)

Starting from **S4** (3.00 g, 11.9 mmol), using general procedure described above, **20** was obtained as colorless oil (1.13 g, 8.44 mmol, 71%). **IR** (neat) 3360(br), 2967(m), 2941(m), 2881(w), 1463(m), 1114(m), 969(s), 812(m) cm^{-1}. **^{1}H NMR** (500 MHz, $CDCl_3$) δ 4.72 (ddddd, *J* = 49.9, 9.8, 7.4, 4.8, 2.3 Hz, 1H, **H-5**), 3.84–3.77 (m, 1H, **H-3**), 1.81 (brs, 1H, **OH**), 1.80–1.46 (m, 6H, **H-4**, **H-6**, **H-2**), 0.98 (t, *J* = 7.4 Hz, 3H, **H-7**), 0.96 (t, *J* = 7.4 Hz, 3H, **H-1**) ppm. **^{13}C{^{1}H} NMR** (126 MHz, $CDCl_3$) δ 92.9 (d, ^{1}J = 165.0 Hz, **C-5**), 69.3 (d, ^{3}J = 2.6 Hz, **C-3**), 41.7 (d, ^{2}J = 19.8 Hz, **C-4**), 30.7 (**C-2**), 28.5 (d, ^{2}J = 21.2 Hz, **C-6**), 9.8 (**C-1**), 9.3 (d, ^{3}J = 6.0 Hz, **C-7**) ppm. **^{19}F NMR** (471 MHz, $CDCl_3$) δ -184.0 – -183.6 (m) ppm. **^{19}F{^{1}H} NMR** (471 MHz, $CDCl_3$) δ -183.8 (s, 1F) ppm. **HRMS**(EI) calcd for $C_7H_{15}FO$ $[M]^{+\cdot}$ 134.1102, found 134.1066.

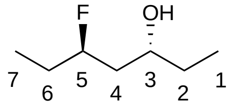

3.7 Synthesis of (±)-*anti*- and *syn*-3,5-difluoropheptane (14 and 15)

3.7.1 General procedure for the fluorination of fluorohydrins SI8 and SI9

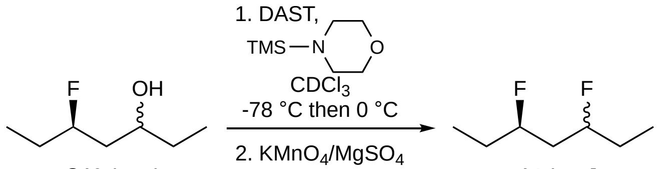

S19 (*syn*)
20 (*anti*)

14 (*anti*)
15 (*syn*)

To a solution of DAST (3.3 equiv.) in $CDCl_3$ (0.4 mL/mmol of DAST) was slowly added *N*-(trimethylsilyl)morpholine (3.4 equiv.) at -78 °C under argon. The resulting solution was stirred at room temperature for 2.5 hours. The reaction mixture was further cooled to -78 °C and a solution of **19** or **20** (1 equiv.) in $CDCl_3$ (1.3 mL/mmol of fluorohydrin) was added dropwise. The resulting solution was stirred for 1.25 hours at 0 °C and then poured into a saturated aqueous $NaHCO_3$ solution (2.7 mL/mmol). After 15 minutes, water (1.4 mL/mmol) was added, the organic layer was separated and the aqueous layer was extracted with $CDCl_3$ (0.9 mL/mmol). The combined organic layers were washed with 1.0 M HCl (2.0 mL/mmol), then with brine (2.0 mL/mmol) and dried over $MgSO_4$. The crude mixture was diluted in EtOH (5.4 mL/mmol) and a solution of $KMnO_4$ (1.2 equiv.) and $MgSO_4$ (1 equiv.) in water (2.0 mL/mmol) was added at 0 °C. The resulting solution was stirred overnight at 8 °C and then quenched with a saturated aqueous $NaHSO_3$ solution. The organic layer was separated and the aqueous layer was extracted with $CDCl_3$ (0.9 mL/mmol). The combined organic layers were washed five times with brine (1.3 mL/mmol) and then dried over $MgSO_4$. The residue was filtered on silica gel (in Pasteur pipette) and rinsed with $CDCl_3$ (1.3 mL/mmol) resulting in a CDCl3 solution of the impure 3,5-difluoroheptane.

3.7.2 (±)-*anti*-3,5-difluoroheptane (14)

Starting from **19** (50 mg, 0.37 mmol), using general procedure described above, **14** was obtained impure as colorless liquid and then submitted to NMR studies. **IR** (neat) 2976(m), 2936(w), 1458(w), 1383(m), 1181(m), 1132(s),1006(m), 807(s) cm^{-1}. **^{1}H NMR** (500 MHz, $CDCl_3$) δ 4.74–4.60 (m, a coupling constant of 49.9 Hz can be observed which disappears upon F decoupling, 2H, **H-3**, **H-5**), 1.85–1.59 (m, 6H, **H-4**, **H-2**, **H-6**), 1.00 (t, $^{3}J_{H1 \text{ and } 7\text{-}H2 \text{ and } 6}$ = 7.5 Hz, 6H, **H-1**, **H-7**) ppm. **^{13}C{^{1}H} NMR** (126 MHz, $CDCl_3$) δ 91.7 (dd, ^{1}J = 167.4 Hz, ^{3}J = 2.9 Hz, 2C, **C-3**, **C-5**), 40.3 (t, ^{2}J = 20.0 Hz, **C-4**), 28.5 (d, ^{2}J = 21.2 Hz, 2C, **C-2**, **C-6**), 9.2

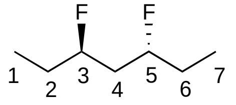

(d, ^3J = 5.7 Hz, 2C, **C-1**, **C-7**) ppm. **^{19}F NMR** (470 MHz, $CDCl_3$) δ -184.3– -184.0 (m, 2F, **F-3**, **F-5**) ppm. **^{19}F{^{1}H} NMR** (471 MHz, $CDCl_3$) δ -184.2 (s, 2F, **F-3**, **F-5**) ppm.

3.7.3 *syn*-3,5-difluoroheptane (15)

Starting from **20** (0.100 g, 0.75 mmol), using general procedure described above, **15** was obtained impure as colorless liquid and then submitted to NMR studies. **IR** (neat) 2978(m), 2934(w), 1456(w), 1382(m), 1194(m), 1128(s),1005(m), 793(s) cm^{-1}. **^{1}H NMR** (500 MHz, $CDCl_3$) δ 4.67–4.53 (m, a coupling constant of 48.6 Hz can be observed which disappears upon F decoupling, 2H, **H-3**, **H-5**), 2.07 (tdt, $^3J_{H4'-F}$ = 18.3 Hz, $^2J_{H4'-H4}$ = 14.8 Hz, $^3J_{H4'-\ H3\ and\ 5}$ = 7.1 Hz, 1H, **H-4'**), 1.83 (tdt, $^3J_{H4-F}$ = 25.8 Hz, $^2J_{H4-H4'}$ = 15.0 Hz, $^3J_{H4-H3\ and\ 5}$ = 4.9 Hz, 1H, **H-4**), 1.75–1.64 (m, 4H, **H-2**, **H-6**), 1.00 (t, $^3J_{H1\ and\ 7-H2\ and\ 6}$ = 7.4 Hz, 6H, **H-1**, **H-7**) ppm. **^{13}C{^{1}H} NMR** (126 MHz, $CDCl_3$) *δ* 92.4 (dd, ^1J = 167.4 Hz, ^3J = 5.5 Hz, 2C, **C-3**, **C-5**), 39.4 (t, ^2J = 21.0 Hz, **C-4**), 27.9 (d, ^2J = 21.7 Hz, 2C, **C-2**, **C-6**), 9.2 (d, ^3J = 6.0 Hz, 2C, **C-1**, **C-7**) ppm. **^{19}F NMR** (471 MHz, $CDCl_3$) *δ* -182.1– -181.8 (m, 2F, **F-3**, **F-5**) ppm. **^{19}F{^{1}H} NMR** (471 MHz, $CDCl_3$) δ -181.9 (s, 2F, **F-3**, **F-5**) ppm.

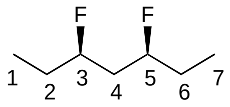

4 Copies of the NMR spectra of compounds 9–15 (Figures S6-S17)

4.1 ^{1}H NMR spectra (Figures S6-S10)

4.1.1 *Anti*-2,4-difluoropentane 12 (^{1}H NMR, $CDCl_3$, 500 MHz, Figure S6a)

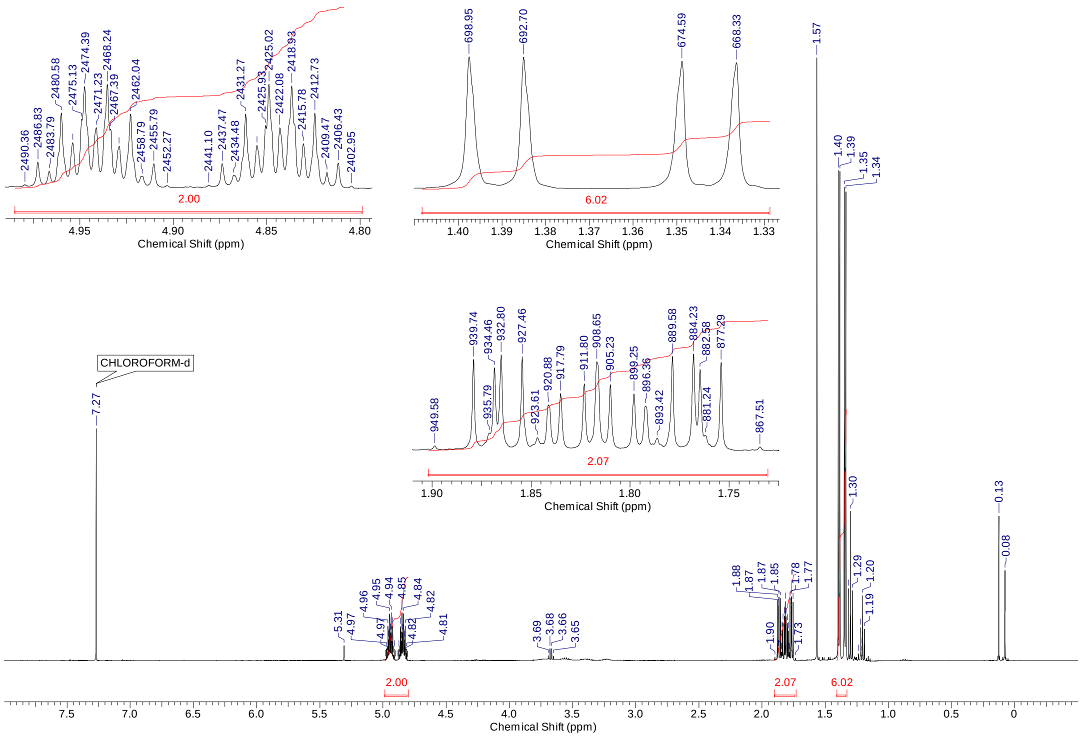

4.1.2 *Anti*-2,4-difluoropentane 12 (^{1}H{^{19}F} NMR, $CDCl_3$, 500 MHz, Figure S6b)

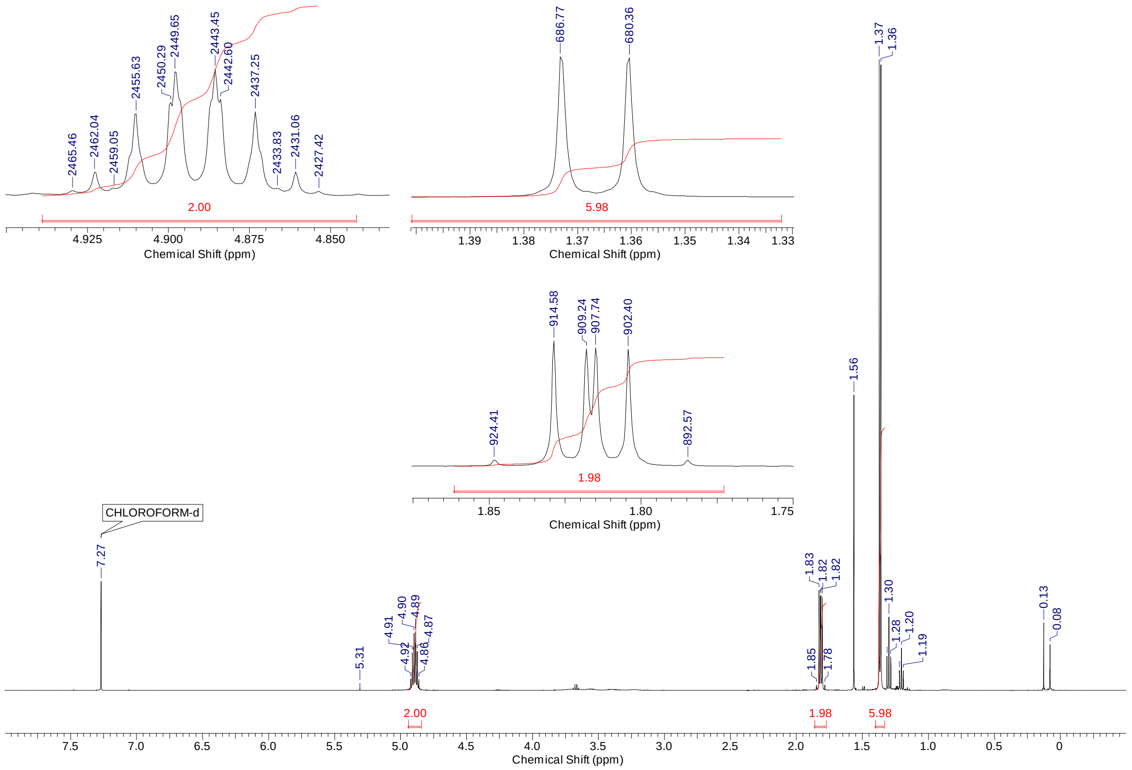

4.1.3 *Syn*-2,4-difluoropentane 13 (^{1}H NMR, $CDCl_3$, 500 MHz, Figure S7a)

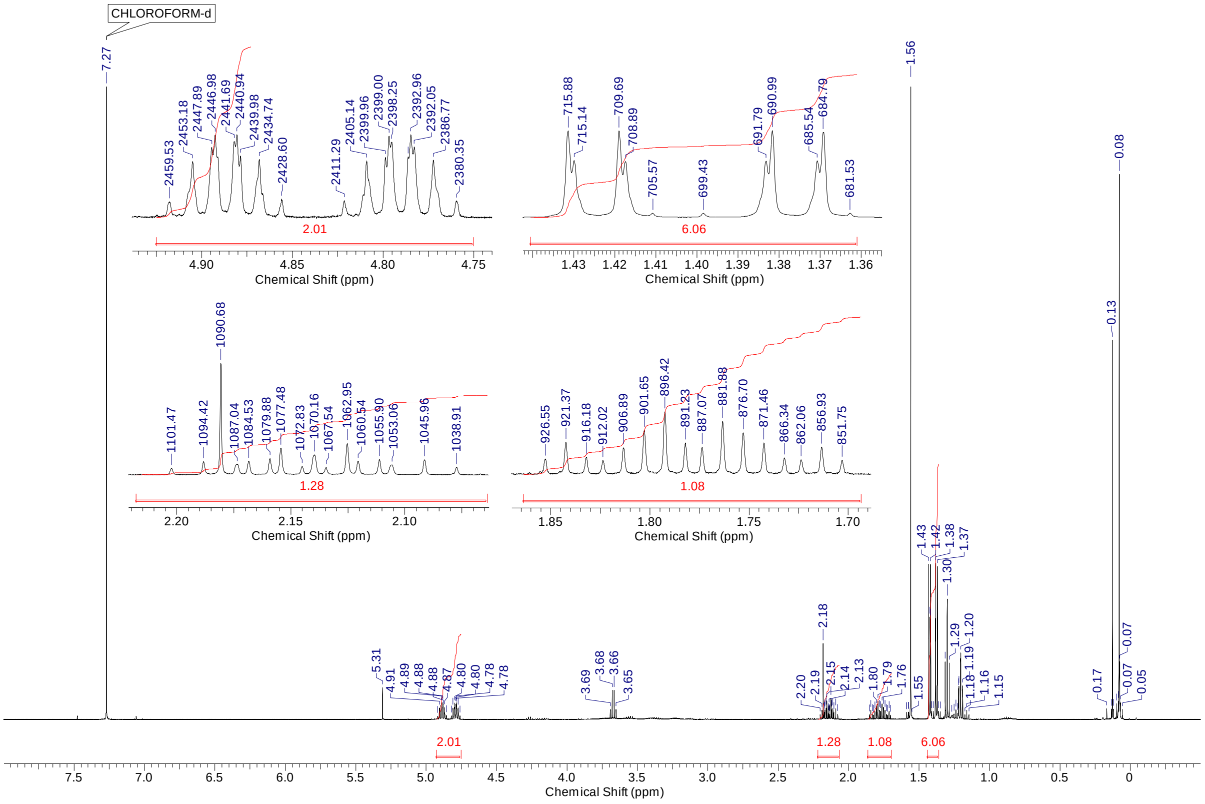

4.1.4 *Syn*-2,4-difluoropentane 13 (^{1}H{^{19}F} NMR, $CDCl_3$, 500 MHz, Figure S7b)

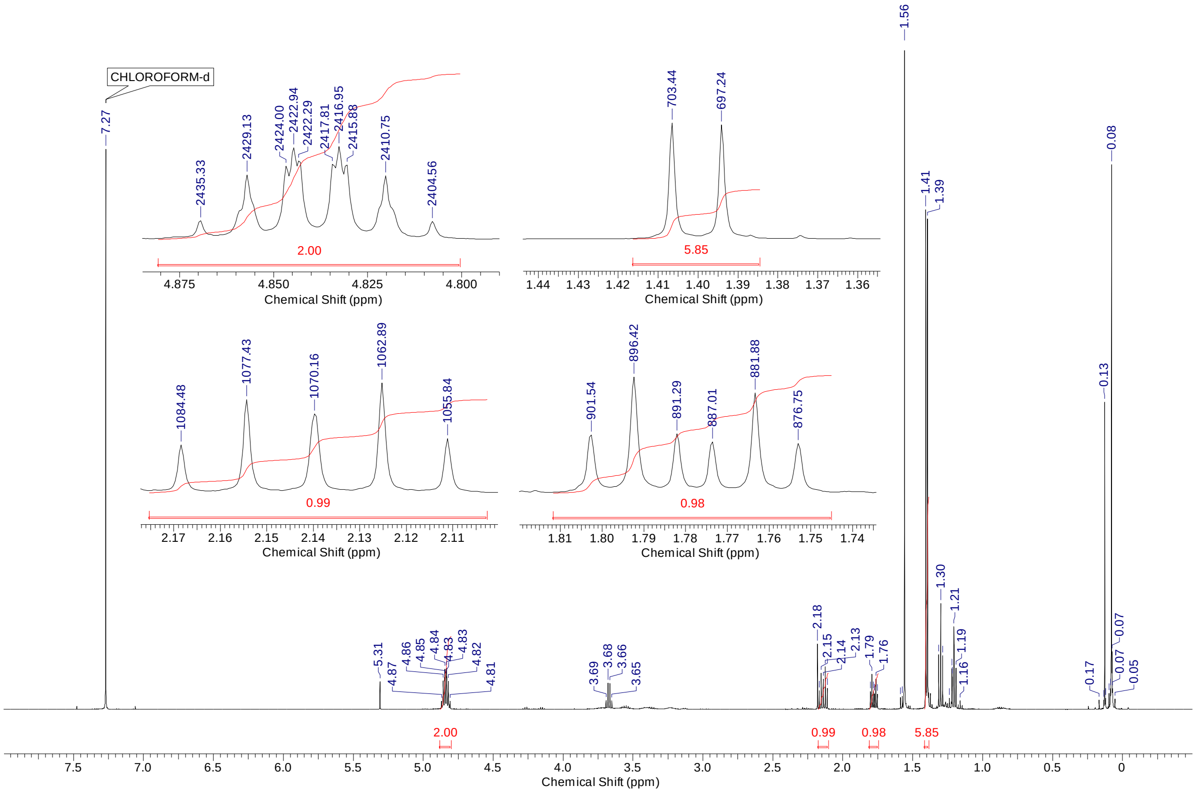

4.1.5 *Anti*-2,4-difluoroheptane 14 (^{1}H NMR, $CDCl_3$, 500 MHz, Figure S8a)

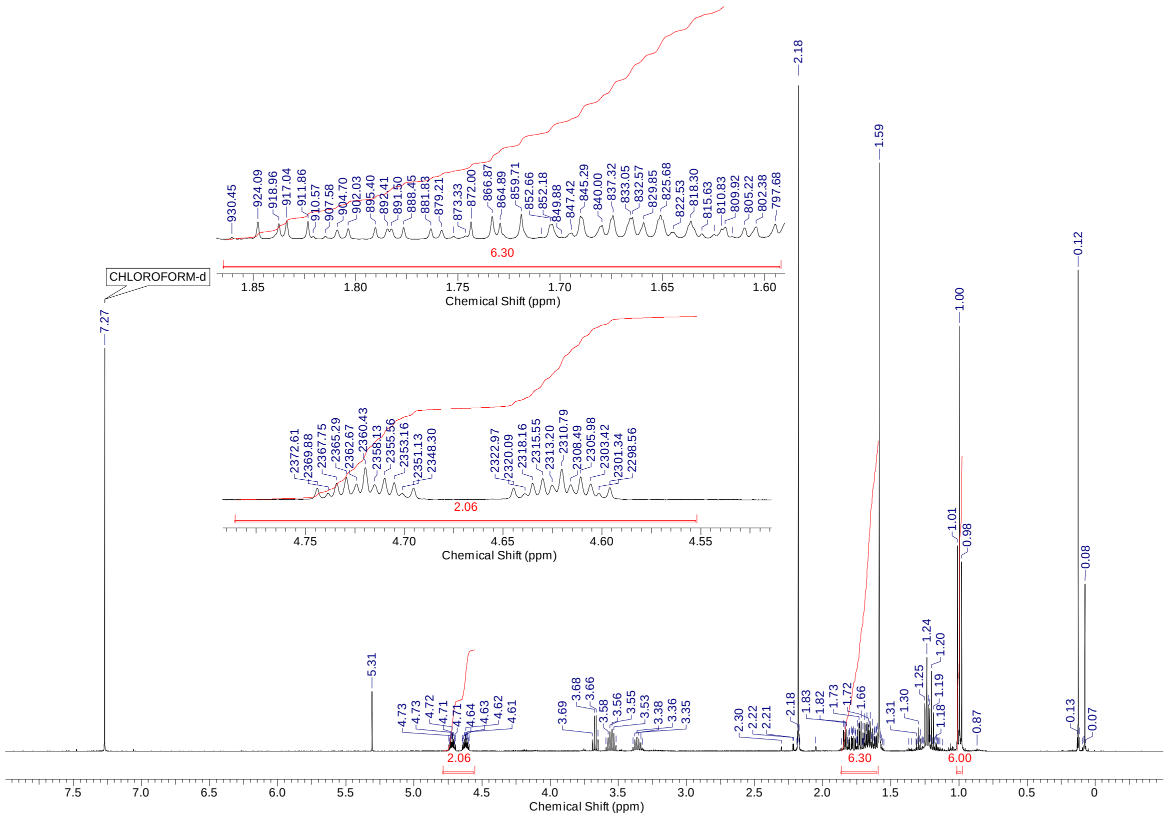

4.1.6 *Anti*-2,4-difluoroheptane 14 (^{1}H{^{19}F} NMR, $CDCl_3$, 500 MHz, Figure S8b)

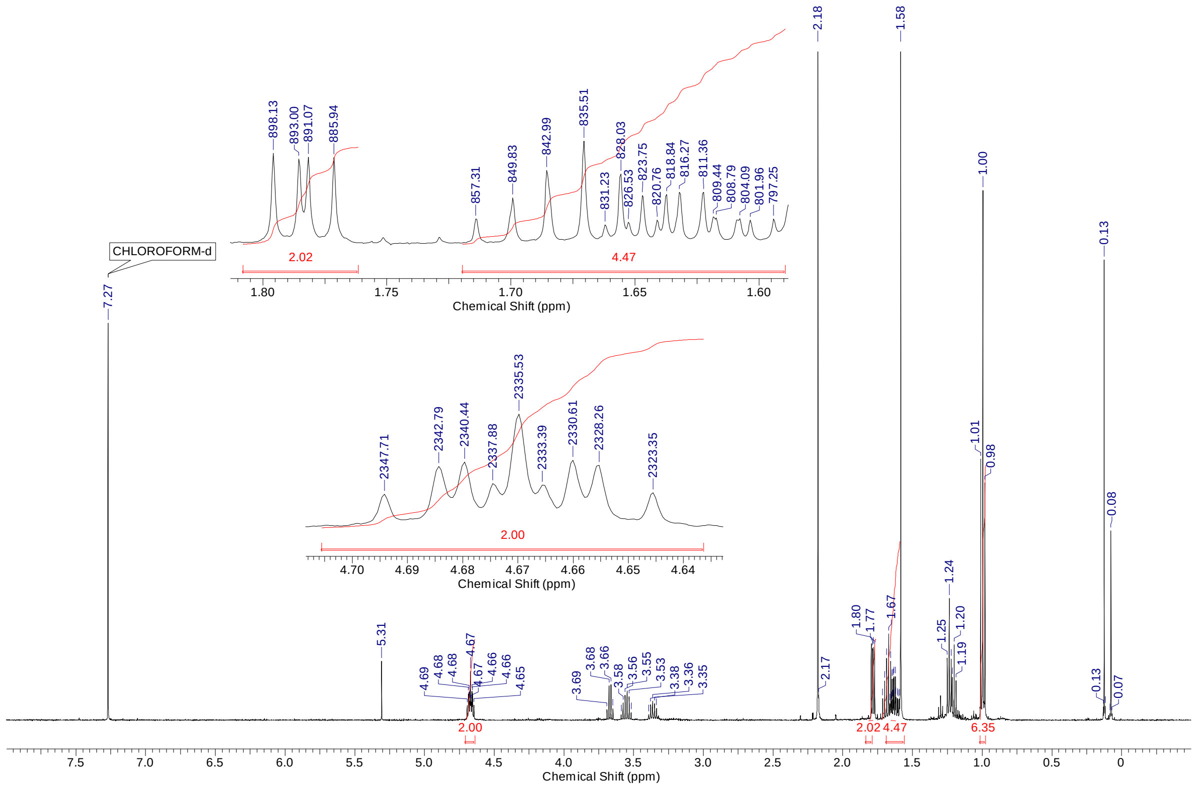

4.1.7 *Syn*-2,4-difluoroheptane 15 (^{1}H NMR, $CDCl_3$, 500 MHz, Figure S9a)

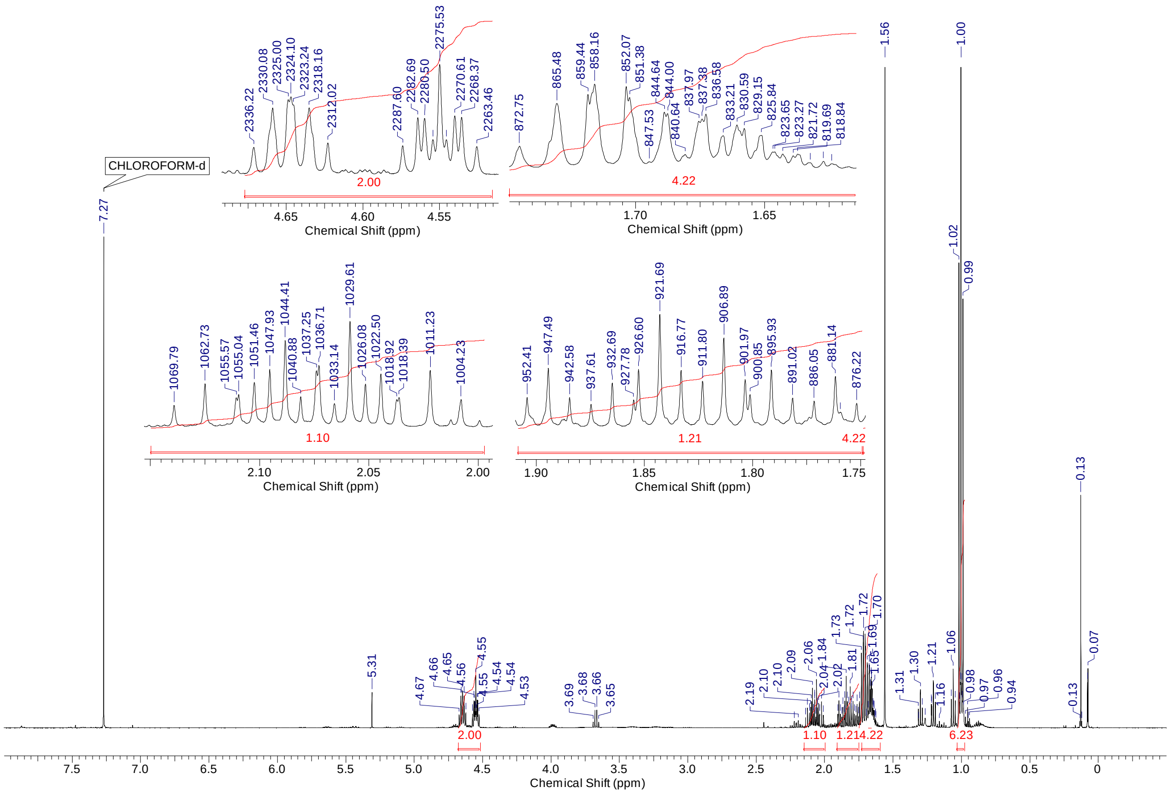

4.1.8 *Syn*-2,4-difluoroheptane 15 (^{1}H{^{19}F} NMR, $CDCl_3$, 500 MHz, Figure S9b)

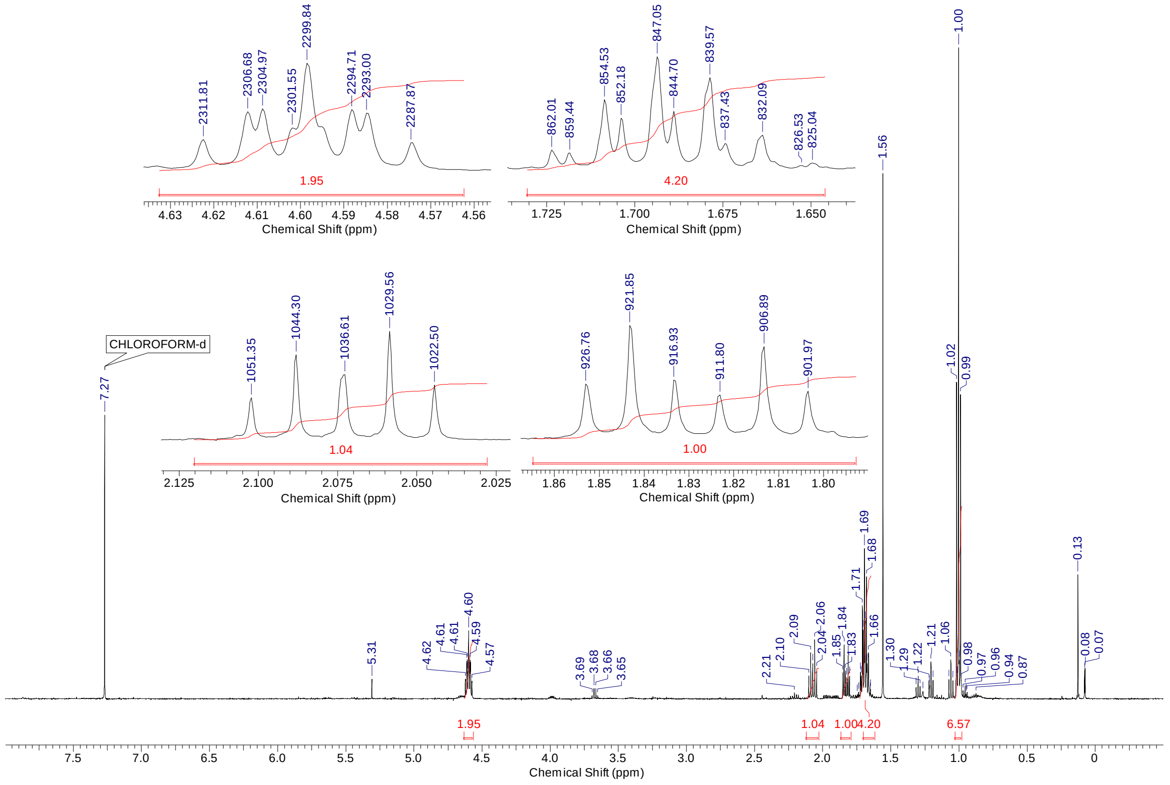

4.2 ^{19}F NMR spectra (Figures S10-S13)

4.2.1 *Anti*-2,4-difluoropentane 12 (^{19}F NMR, $CDCl_3$, 471 MHz, Figure S10a)

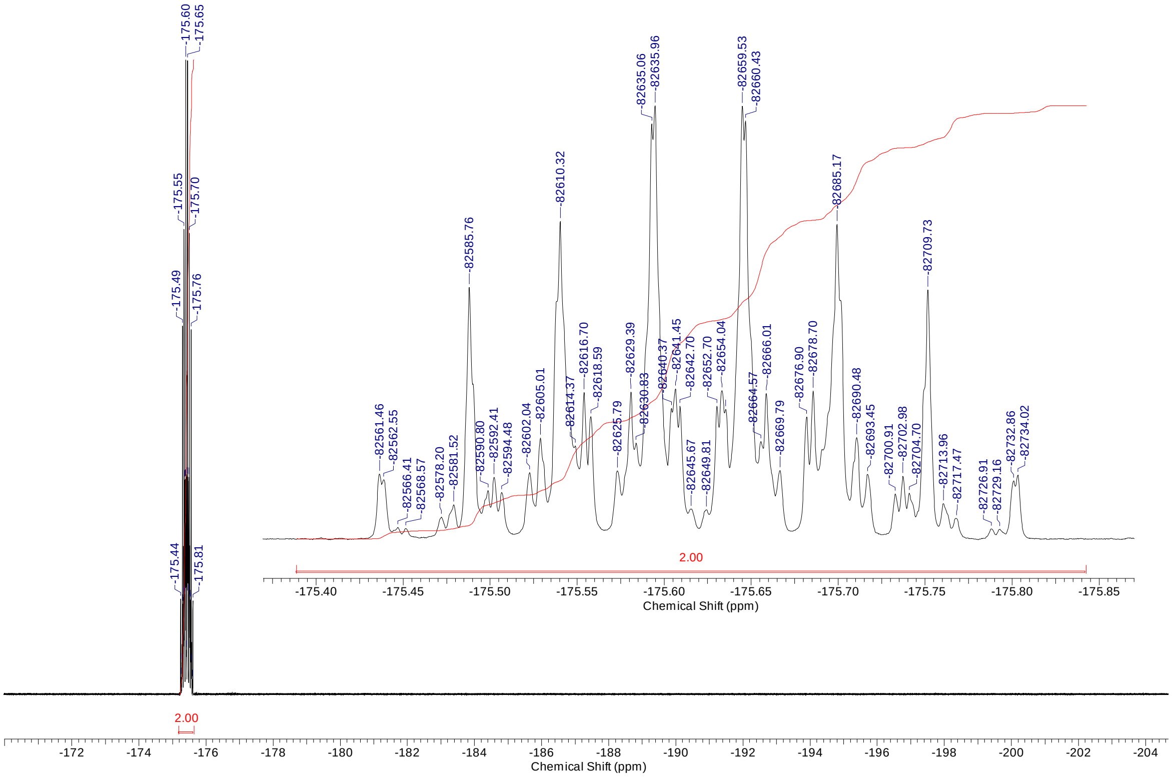

4.2.2 *Anti*-2,4-difluoropentane 12 (^{19}F{^{1}H} NMR, $CDCl_3$, 471 MHz, Figure S10b)

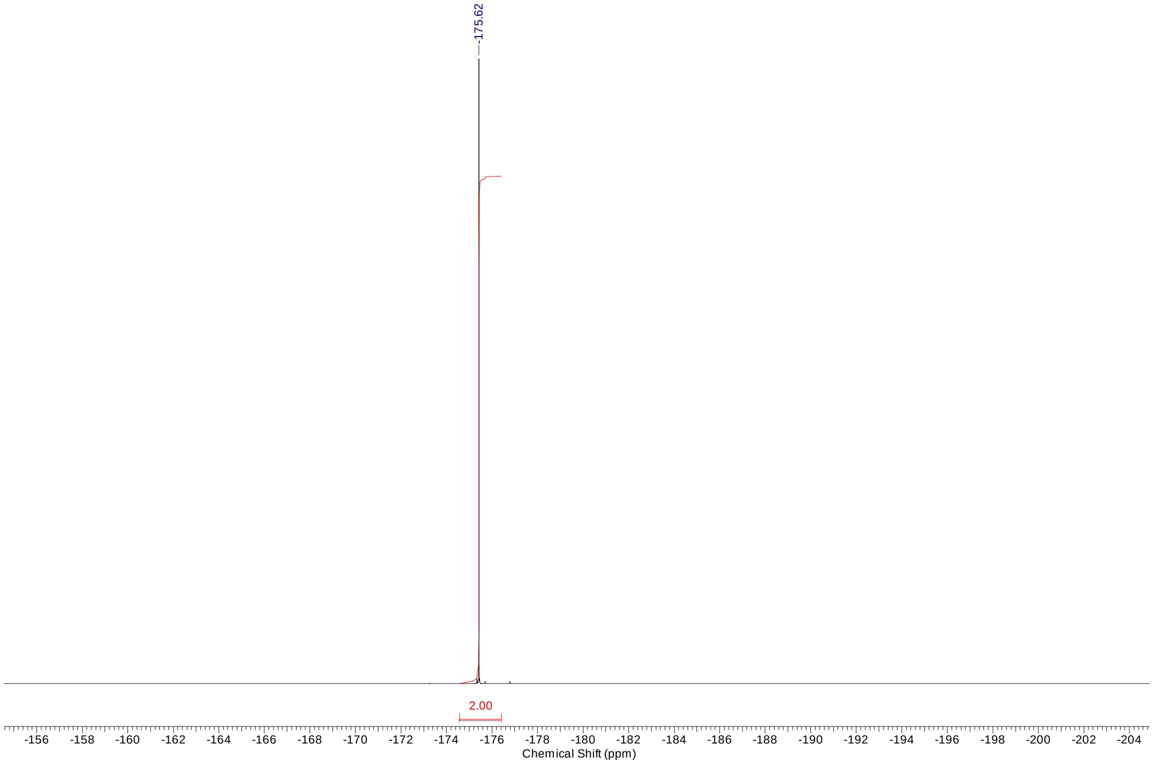

4.2.3 *Syn*-2,4-difluoropentane 13 (^{19}F NMR, $CDCl_3$, 471 MHz, Figure S11a)

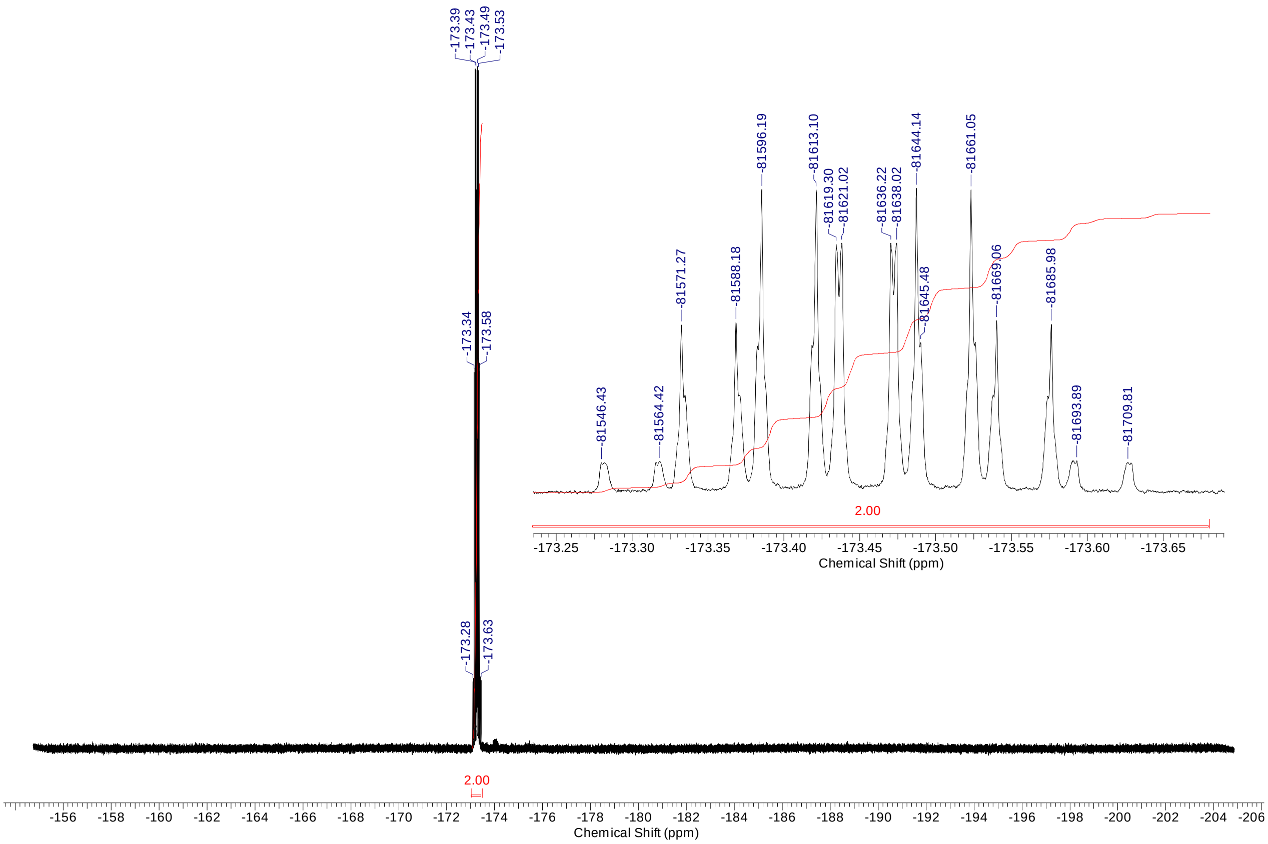

4.2.4 *Syn*-2,4-difluoropentane 13 (^{19}F{1H} NMR, $CDCl_3$, 471 MHz, Figure S11b)

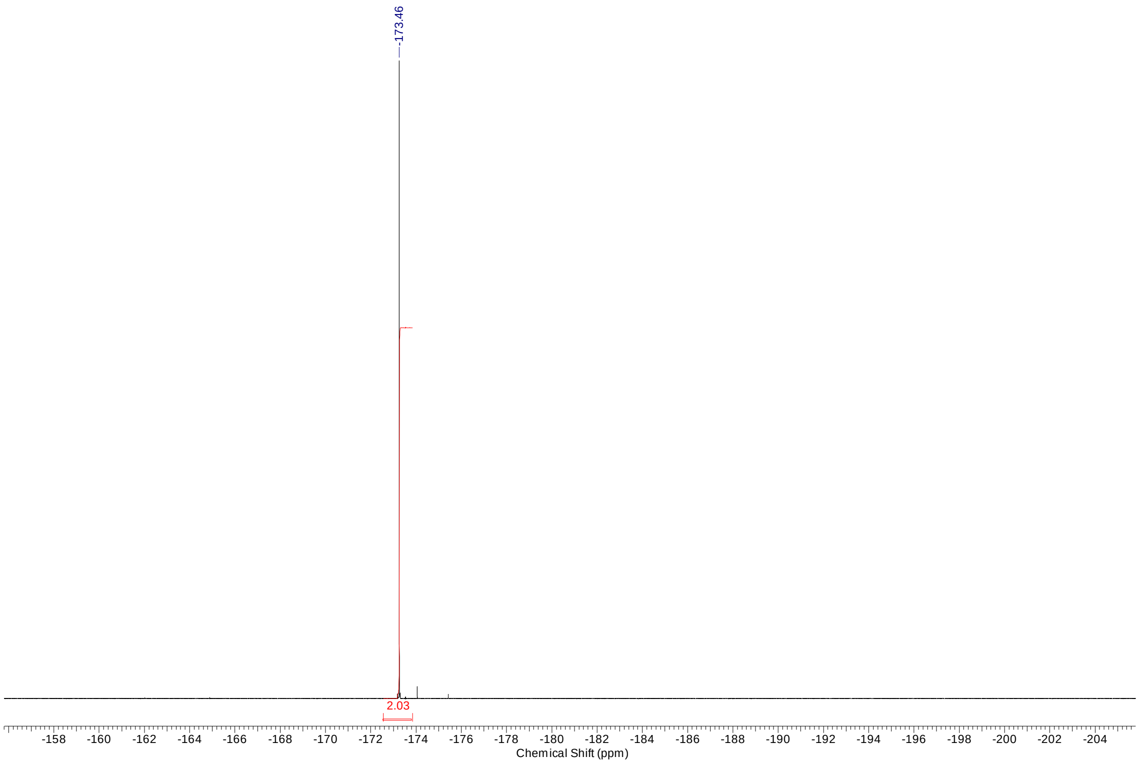

4.2.5 *Anti*-2,4-difluoroheptane 14 (^{19}F NMR, $CDCl_3$, 471 MHz, Figure S12a)

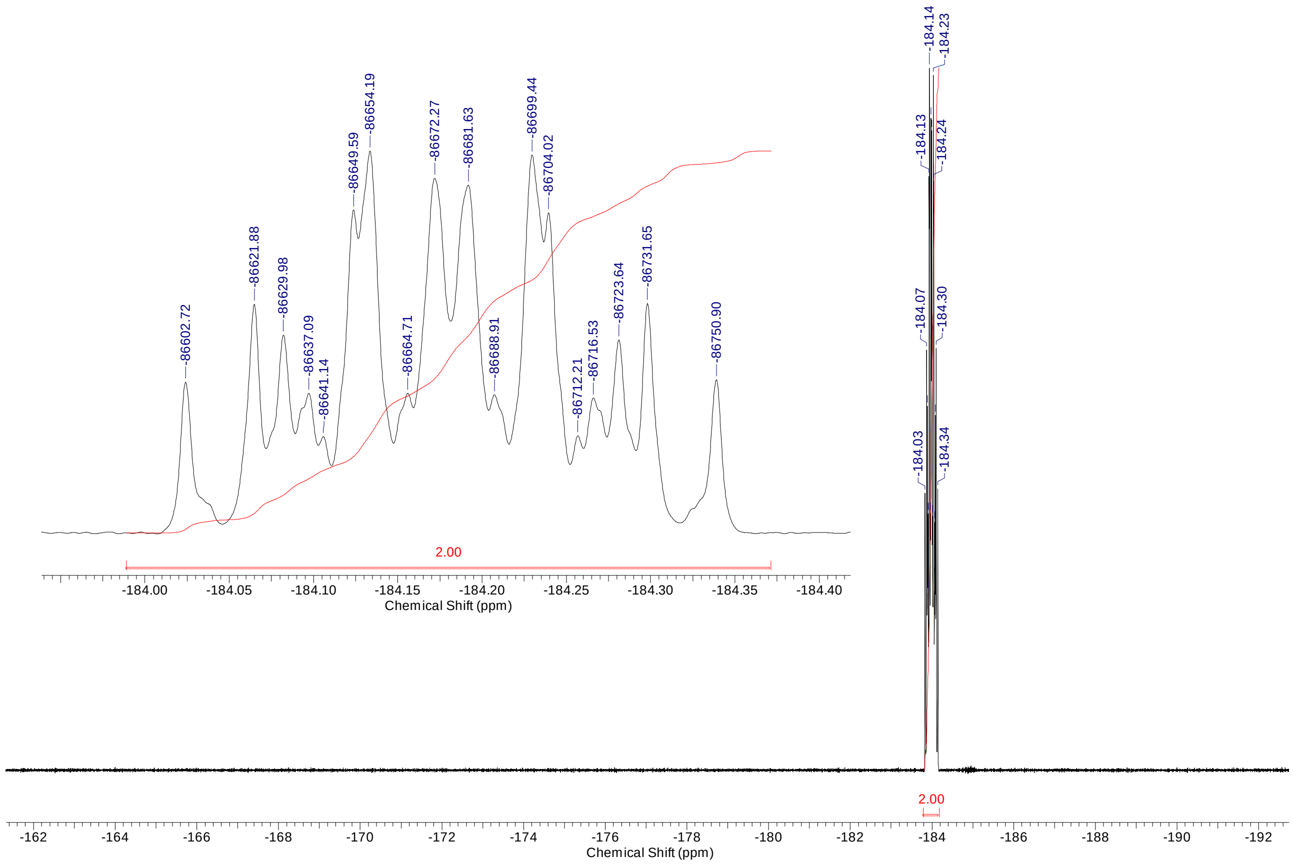

4.2.6 *Anti*-2,4-difluoroheptane 14 ^{19}F{^{1}H} NMR ($CDCl_3$, 471 MHz, Figure S12b)

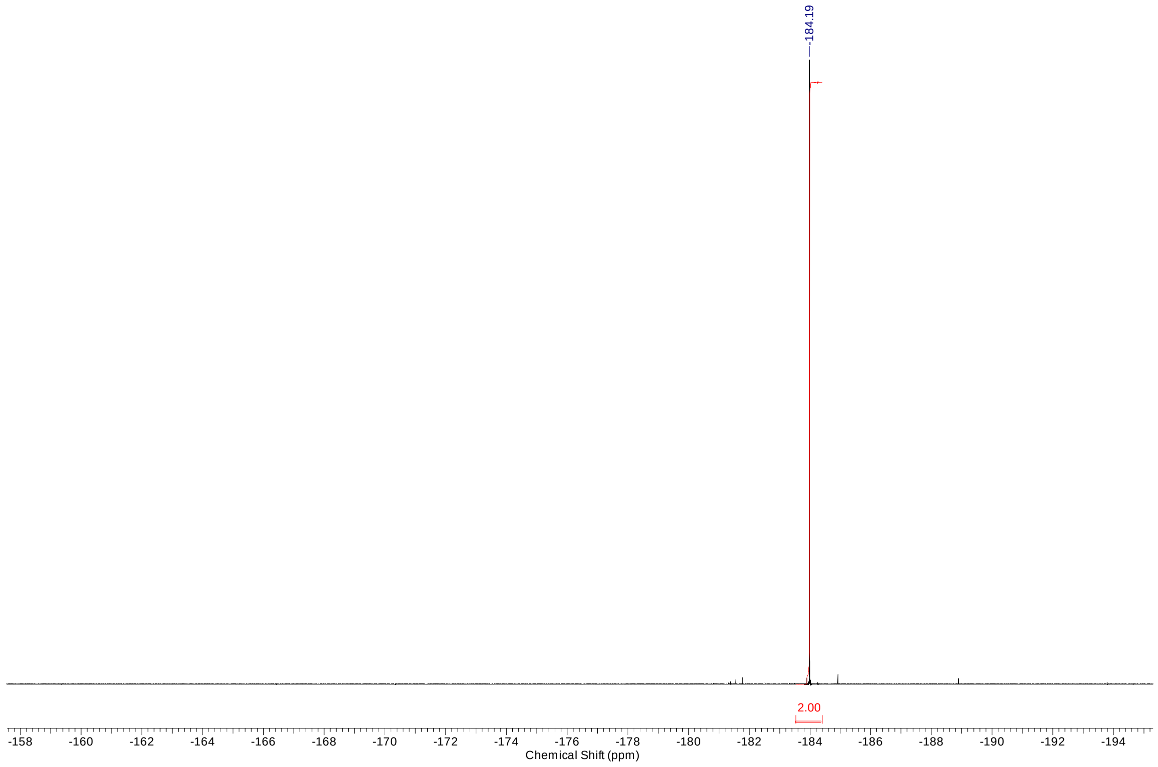

4.2.7 *Syn*-2,4-difluoroheptane 15 (^{19}F NMR, $CDCl_3$, 471 MHz, Figure S13a)

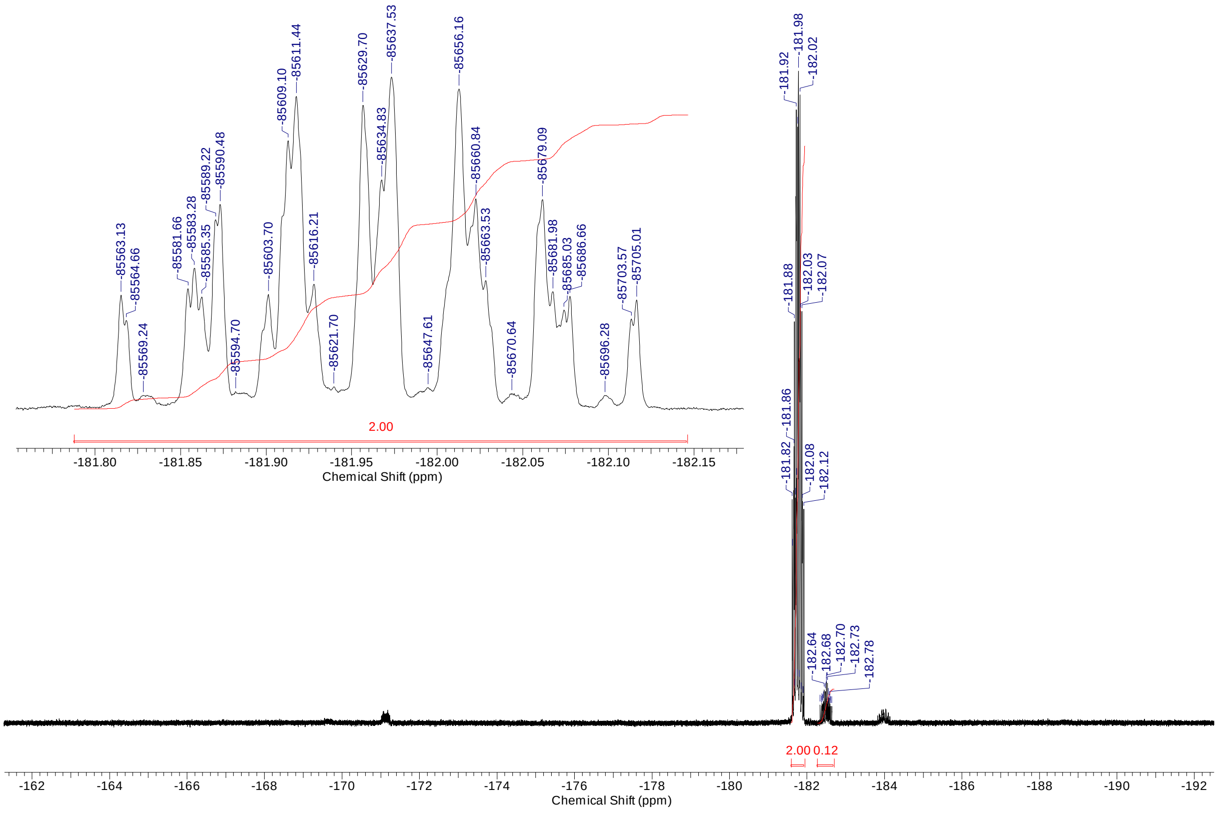

4.2.8 *Syn*-2,4-difluoroheptane 15 (^{19}F{^{1}H} NMR, $CDCl_3$, 471 MHz, Figure S13b)

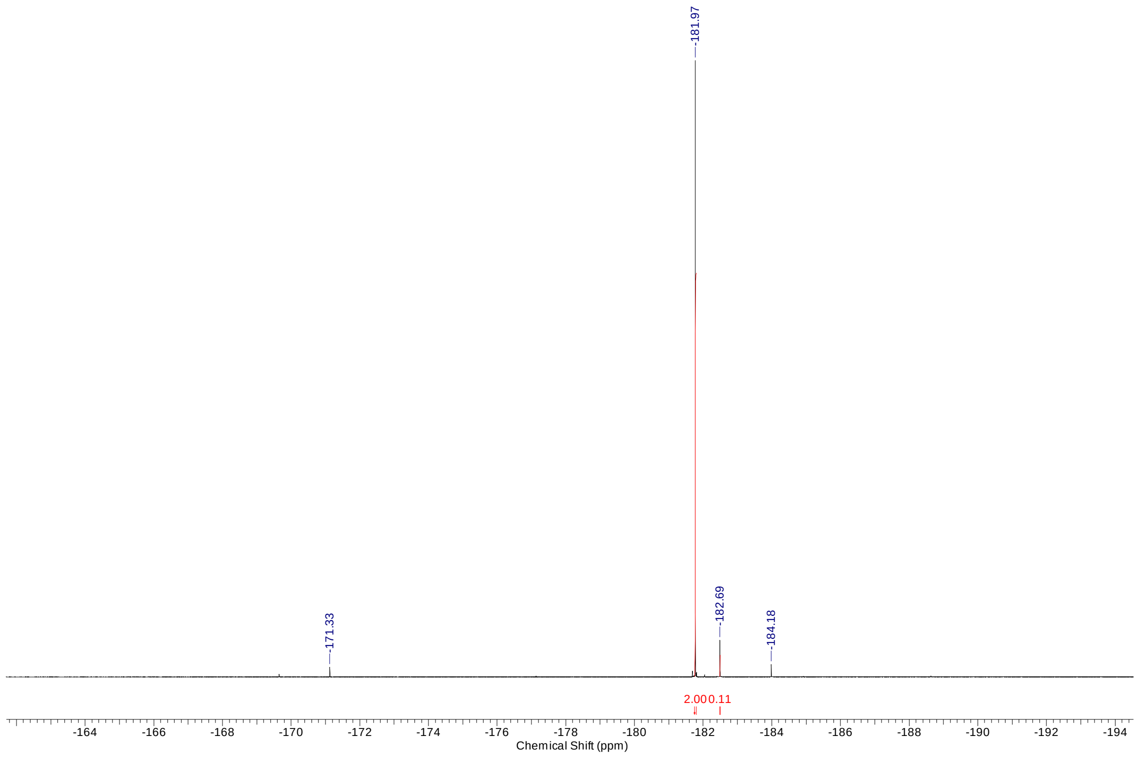

4.3 ^{13}C{1H} NMR spectra ($CDCl_3$, 126 MHz)(Figures S14-S17)

4.3.1 *Anti*-2,4-difluoropentane 12 (^{13}C{^{1}H} NMR, $CDCl_3$, 126 MHz, Figure S14)

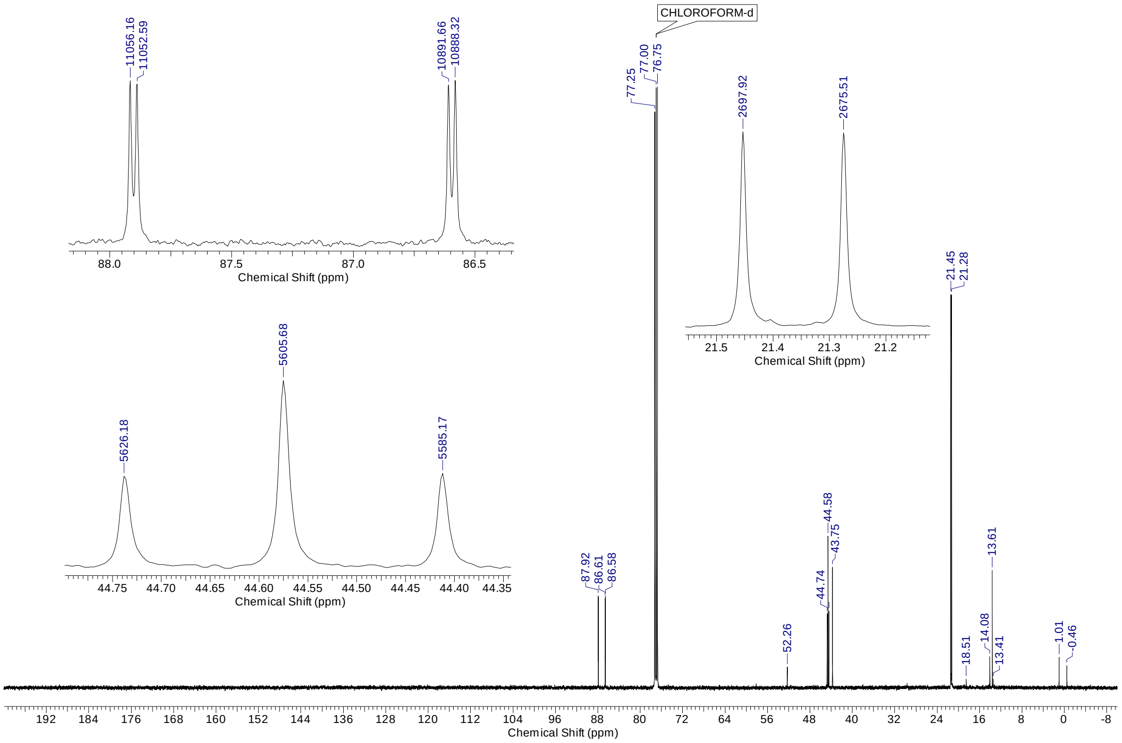

4.3.2 *Syn*-2,4-difluoropentane 13 (^{13}C{^{1}H} NMR, $CDCl_3$, 126 MHz, Figure S15)

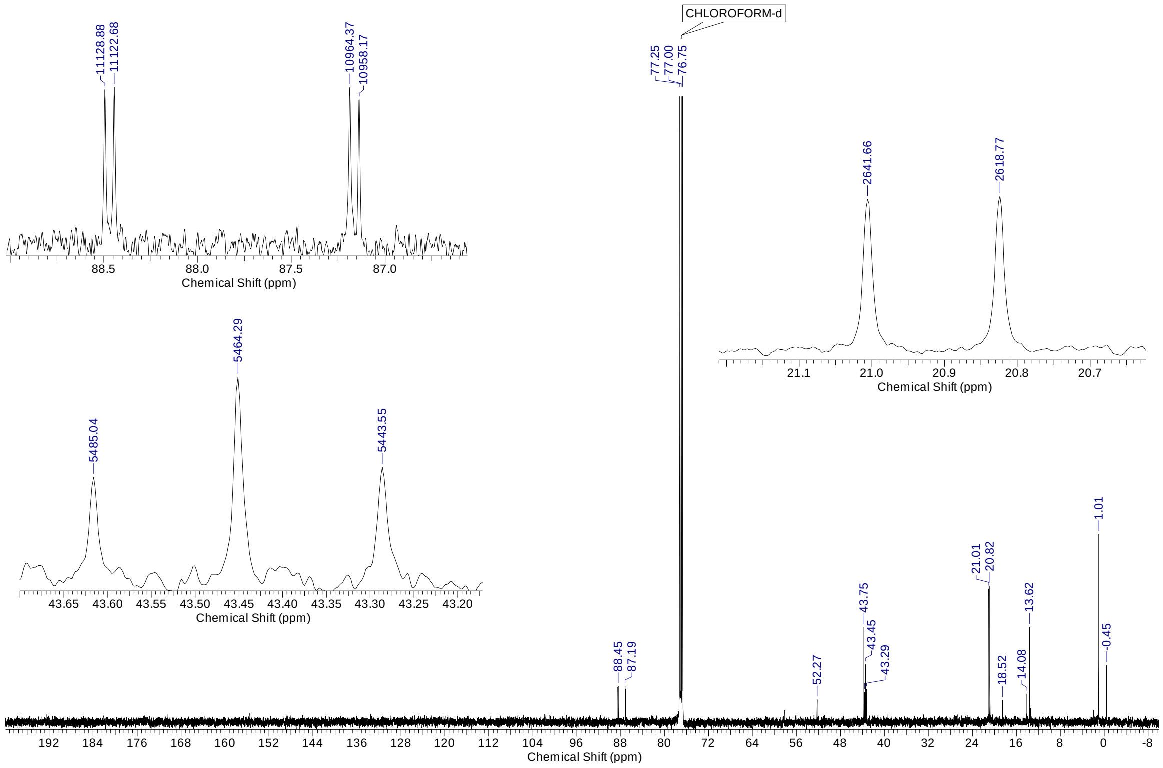

4.3.3 *Anti*-3,5-difluoroheptane 14 ($^{13}C\{^1H\}$ NMR, $CDCl_3$, 126 MHz, Figure S16)

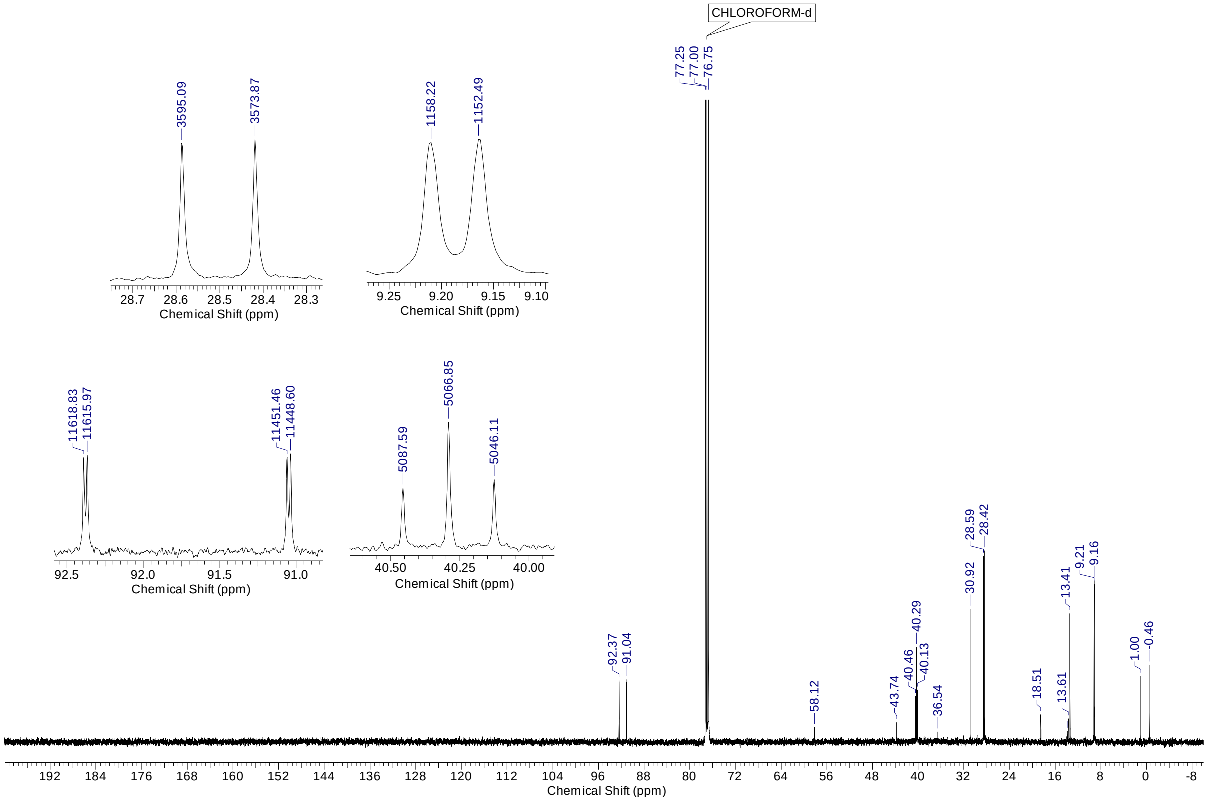

4.3.4 *Syn*-3,5-difluoroheptane 15 ($^{13}C\{^1H\}$ NMR, $CDCl_3$, 126 MHz, Figure S17)

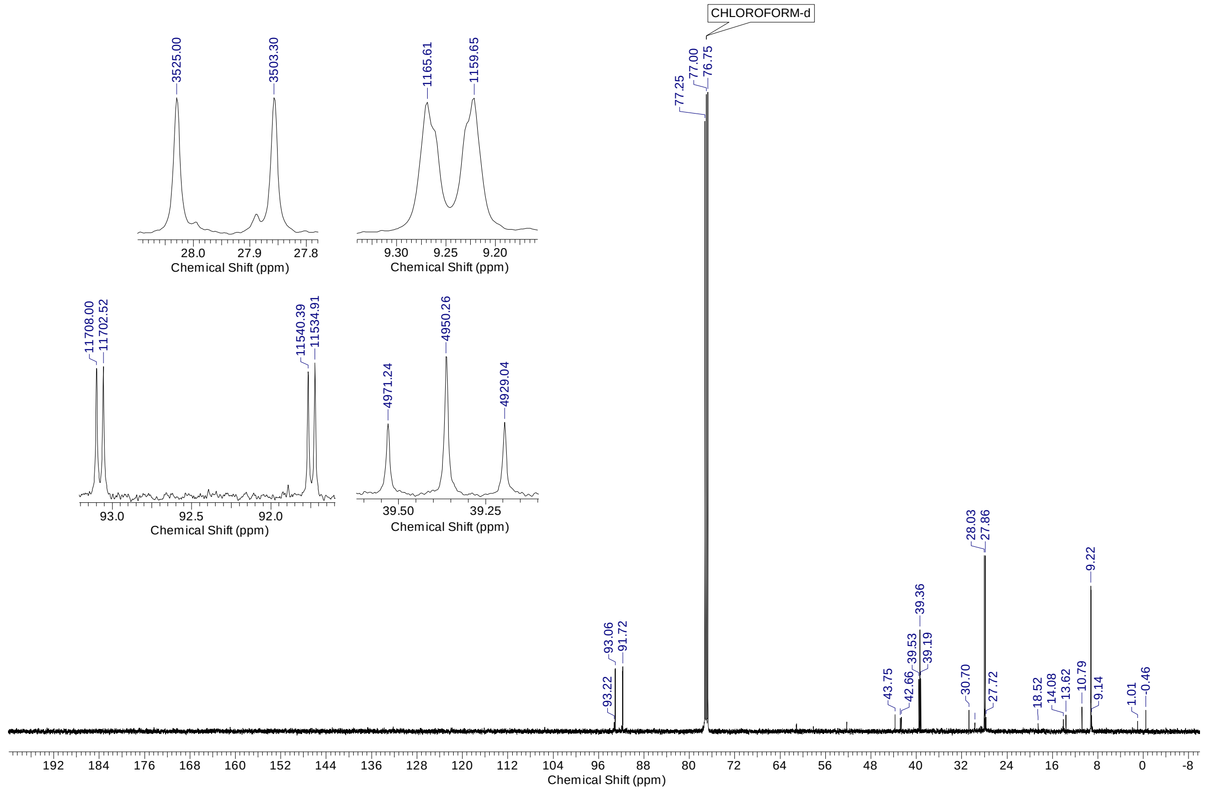

5 NMR fitting

5.1 Systematic grid scan of 1,3-difluoropropane dihedral angles (Figure S18)

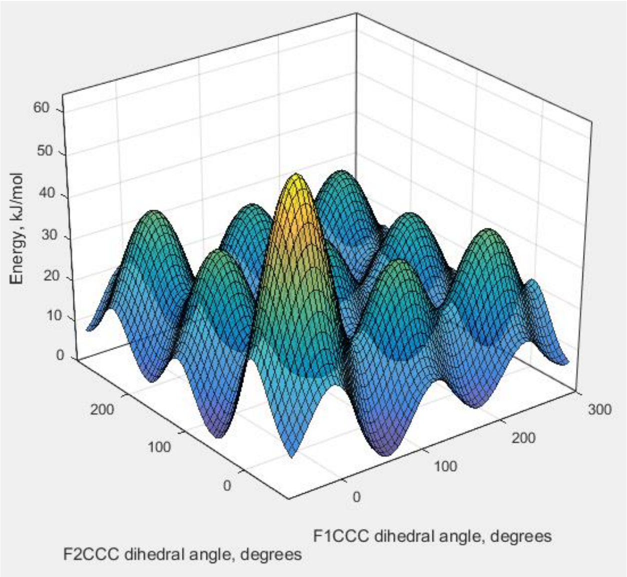

Figure S18. Dihedral angle dependence of the total energy in 1,3-difluoropropane. The calculation was performed using M06/cc-pVTZ method in SMD chloroform as described in the main text.

5.2 Fitted NMR spectra for compounds 12-15 (Figures S19-S22)

5.2.1 *Anti*-2,4-difluoropentane 12 (Figure S19)

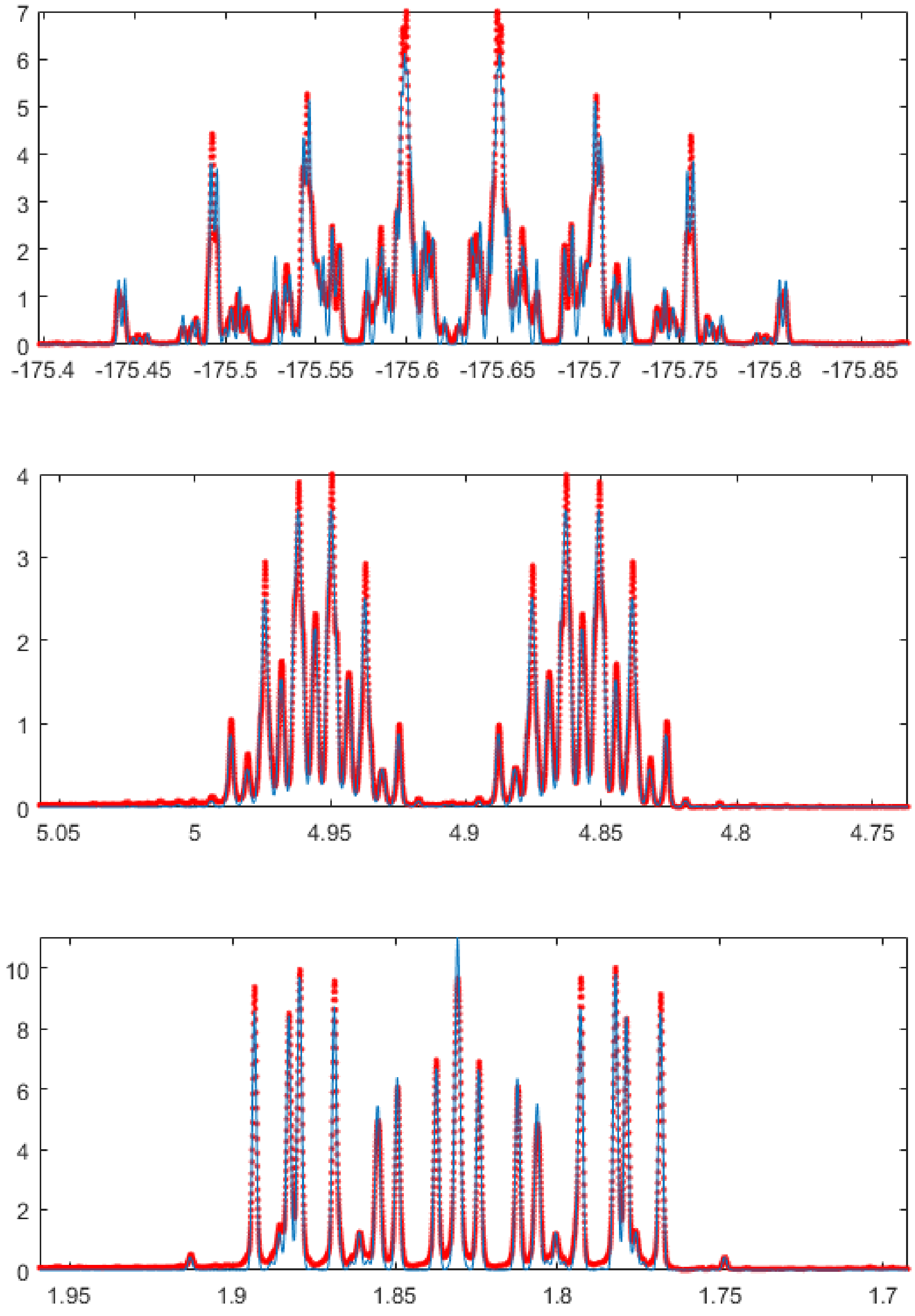

Figure S19. *Experimental (red dots) and theoretical (blue lines) ^{19}F and ^{1}H NMR spectra of anti-2,3-difluoro-pentane **12** in $CDCl_3$ at 11.75 Tesla magnetic induction.*

5.2.2 *Syn*-2,4-difluoropentane 13 (Figure S20)

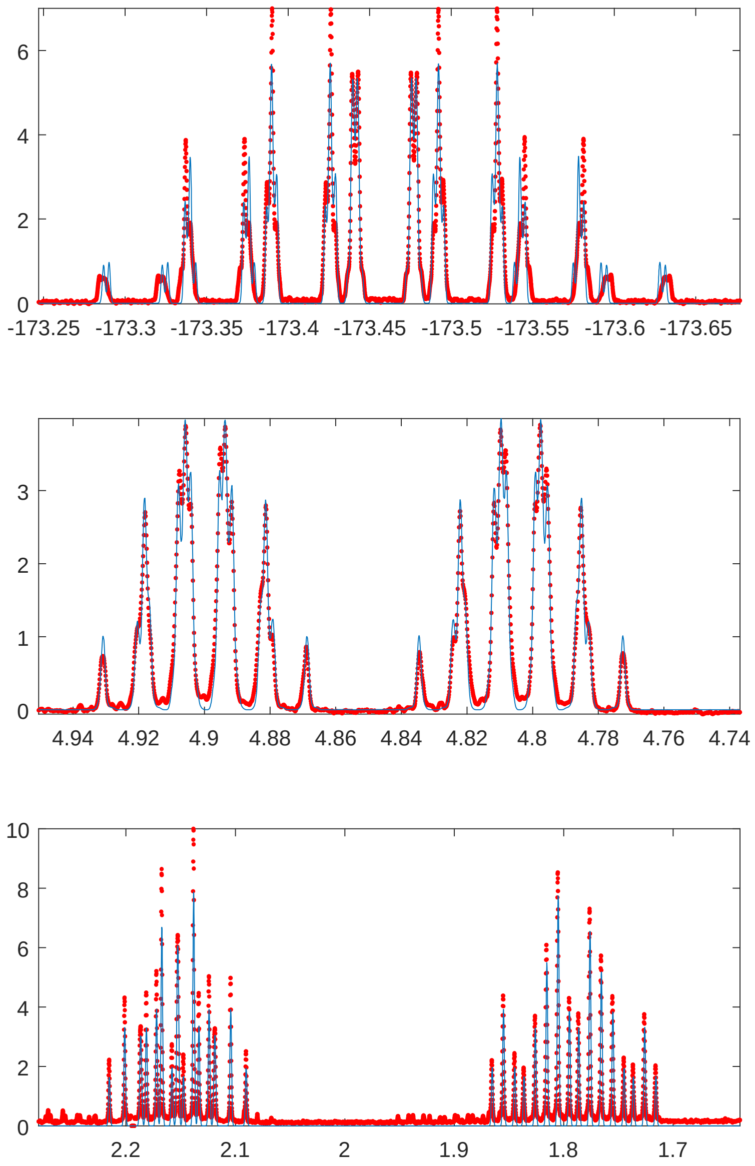

Figure S20. *Experimental (red dots) and theoretical (blue lines)* ^{19}*F and* ^{1}*H NMR spectra of syn-2,3-difluoropentane* ***13*** *in* $CDCl_3$ *at 11.75 Tesla magnetic induction.*

5.2.3 *Anti*-3,5-difluoroheptane 14 (Figure S21)

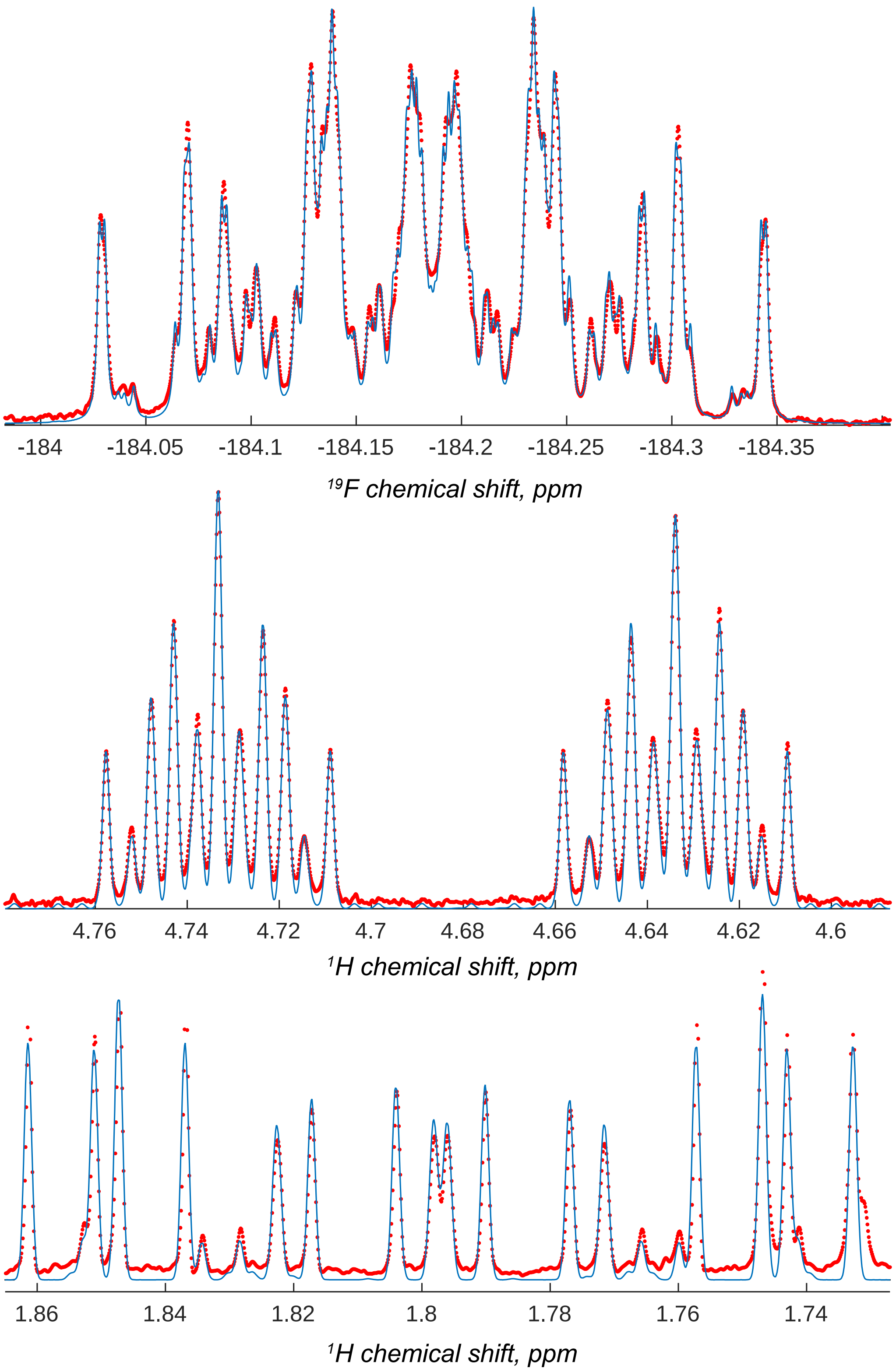

Figure S21. *Experimental (red dots) and theoretical (blue lines)* ^{19}F *and* ^{1}H *NMR spectra of anti-3,5-difluoroheptane* ***14*** *in* $CDCl_3$ *at 11.75 Tesla magnetic induction.*

5.2.4 *Syn*-3,5-difluoroheptane 15 (Figure S22)

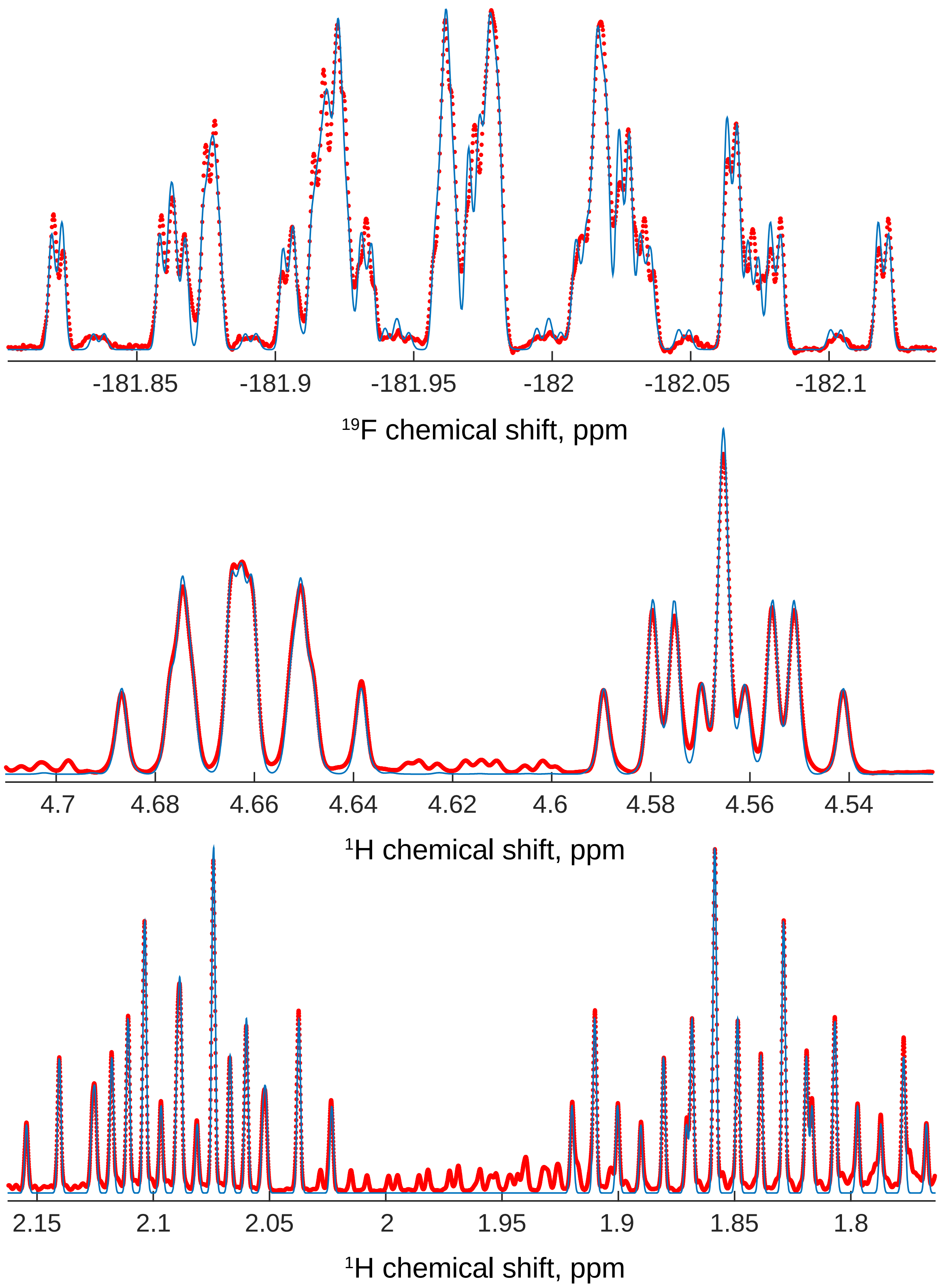

Figure S22. *Experimental (red dots) and theoretical (blue lines)* ^{19}*F and* ^{1}*H NMR spectra of syn-3,5-difluoroheptane* ***15*** *in chloroform at 11.75 Tesla magnetic induction*

5.3 Simulation spectra using the set of DFT *J*-couplings (for 14 and 15, Figures S23, S24).

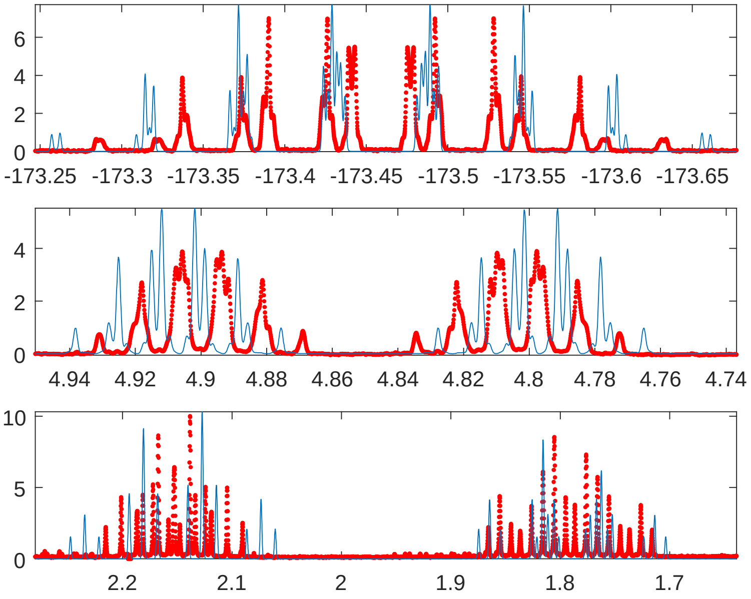

Figure S23. *Experimental (red dots) and theoretical (blue lines) ^{19}F and ^{1}H NMR spectra of syn-3,5-difluoroheptane **14** in chloroform at 11.75 Tesla magnetic induction*

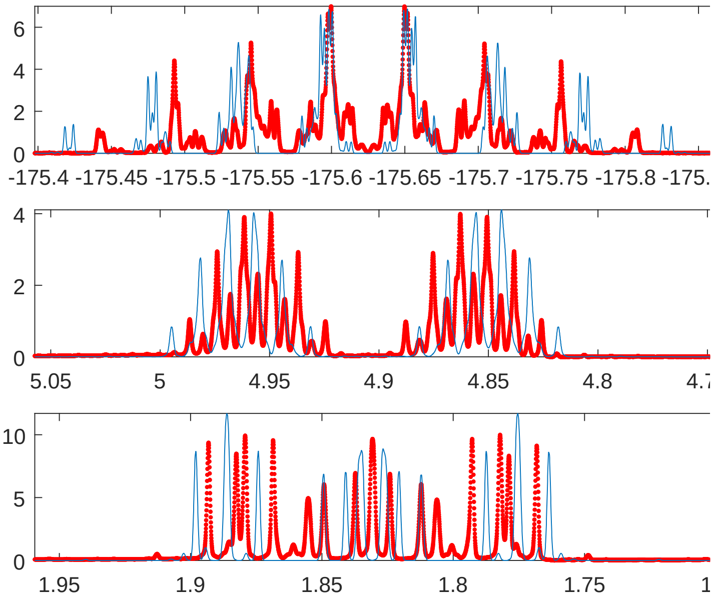

Figure S24. *Experimental (red dots) and theoretical (blue lines) ^{19}F and ^{1}H NMR spectra of anti-3,5-difluoroheptane **15** in chloroform at 11.75 Tesla magnetic induction*

5.4 Simulation of the F-decoupled H NMR spectra with the obtained fitting parameters (Figures S25, S26)

We use the same parameters and switch on the decoupling matches the experimental spectra without additional fitting. This is strong proof that the fitting is correct as it would be highly unlikely that otherwise the still complex $^{1}H\{^{19}F\}$ spectrum would be reproduced

5.4.1 Figure S25: *anti*-3,5-difluoroheptane 14

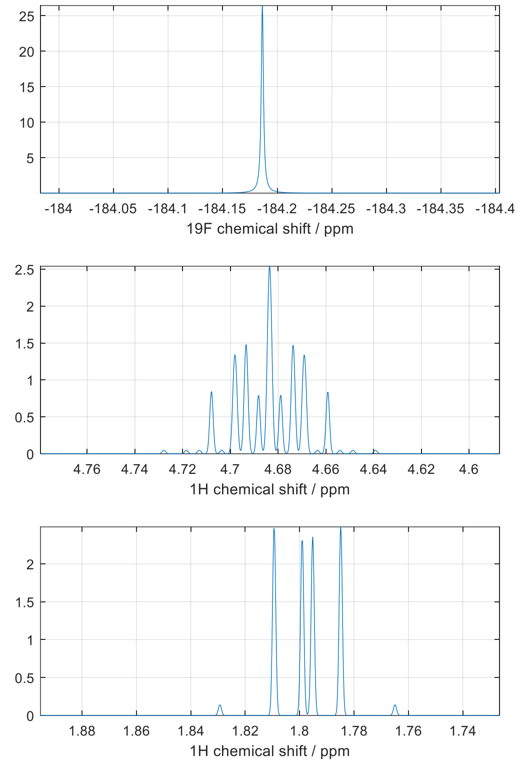	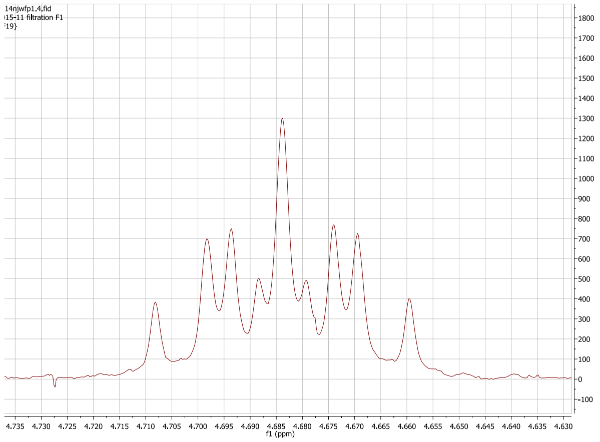<br>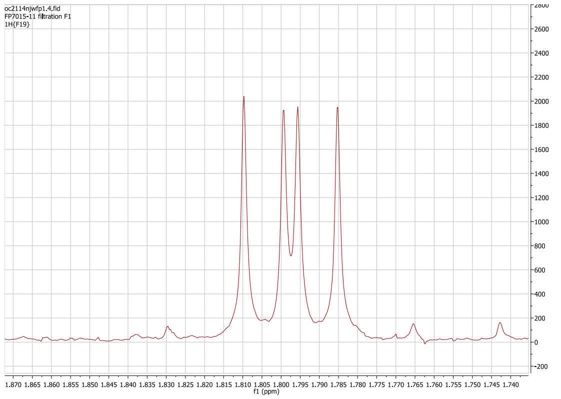
Theoretical decoupled $\{^{1}H\}^{19}F$ and $\{^{19}F\}^{1}H$ NMR spectra of *anti*-3,5-difluoroheptane in chloroform at 11.75 Tesla magnetic induction, computed using the parameters produced during the fitting of the non-decoupled spectra	Expansions of the experimental $\{^{19}F\}^{1}H$ NMR spectrum.

5.4.2 Figure S26: *syn*-3,5-difluoroheptane 15

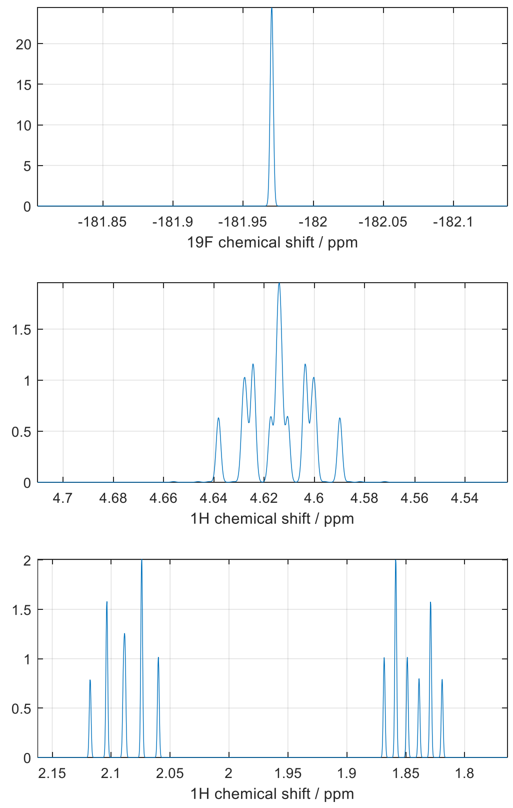	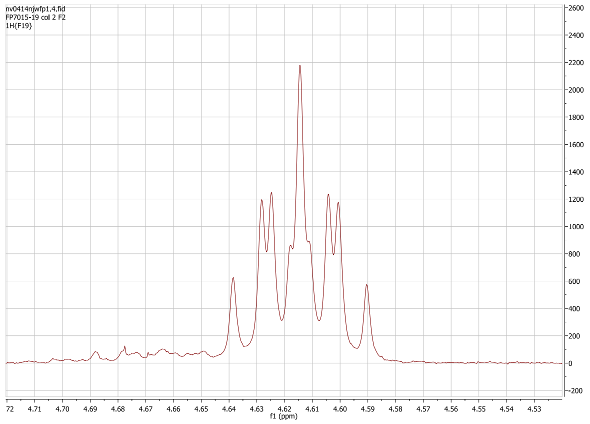<br>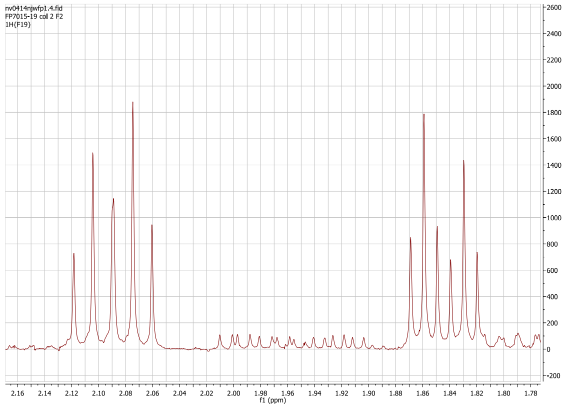
Theoretical decoupled {^{1}H}^{19}F and {^{19}F}^{1}H NMR spectra of *syn*-3,5-difluoroheptane in chloroform at 11.75 Tesla magnetic induction, computed using the parameters produced during the fitting of the non-decoupled spectra	Expansions of the experimental {^{19}F}^{1}H NMR spectrum.

6 Full lists of "data fitted" coupling constants for 1,12-15

6.1 Table S3: chemical shifts of 1,3-difluoropropane (1), obtained by fitting experimental data.

Atoms	Experimental chemical shift / ppm
F	–223.5314
CH_2F (protons)	4.6075
CH_2	2.1011

6.2 Table S4: 1,3-difluoropropane (1), *J*-couplings (Hz).

Atoms	GIAO DFT[b]	NMR fitting[c]
F-H (2-bond)	54.7	47.01
F-H (3-bond)	29.3	25.77
H-H (3-bond)	4.9	5.79

[a] Coupling dominated by non-contact terms, DFT is known to be a poor predictor.[12] [b] Ensemble average *J*-couplings. [c] Experimental fitted *J*-couplings.

6.3 Table S5: chemical shifts of *anti*-2,4-difluoropentane (12), obtained by fitting experimental data.

anti-3,5-difluoropentane **12**

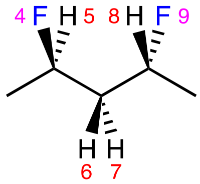

Atoms	Experimental chemical shift / ppm
1,2,3,10,11,12 (CH_3 groups)	1.3810
4,8 (CHF protons)	4.9062
6,7 (middle CH_2)	1.8307
4,9 (fluorines)	-175.6250

6.4 Table S6: *anti*-2,4-difluoropentane (12), *J*-couplings (Hz).

Atoms	GIAO DFT Monte-Carlo[c]	Monte-Carlo st. dev.[d]	NMR fitting[e]
5-(1,2,3), 8-(10,11,12) (CHF proton to CH_3)	6.6	0.5	6.2
4-(1,2,3), 9-(10,11,12) (fluorine to CH_3)	26.6	2.5	23.9
5-6, 7-8 (CHF proton to CH_2, *anti*[a])	8.7	1.0	9.8
5-7, 6-8 (CHF proton to CH_2, *syn*[a])	3.2	0.3	2.4
4-6, 9-7 (F to CH_2, *syn*[a])	17.7	1.3	13.7
4-7, 9-6 (F to CH_2, *anti*[a])	37.6	4.2	36.4
4-5, 8-9 (F to H, geminal)	56.5	5.4	49.3
4-9 (between the F spins)	2.7	0.2	1.6
6-7 (geminal)[b]	-26.6	2.6	–15.1

[a] as shown on the zigzag structure. [b] Coupling dominated by non-contact terms, DFT is known to be a poor predictor.[12] [c] Ensemble average *J*-couplings.[d] Does not include systematic errors of DFT. [e] Experimental fitted *J*-couplings.

6.5 Table S7: chemical shifts of *syn*-2,4-difluoropentane (13), obtained by fitting experimental data.

syn-3,5-difluoropentane **13**

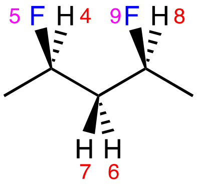

Atoms	Experimental chemical shift / ppm
1,2,3,10,11,12 (CH_3 groups)	1.4135
4, 8 (CHF protons)	4.8515
7 (CH_2, *syn*[a] to F)	2.1519
6 (CH_2, *anti*[a] from F)	1.7910
5, 9 (fluorines)	−181.9705

[a] as shown on the zigzag structure.

6.6 Table S8: *syn*-2,4-difluoropentane (13), *J*-couplings (Hz).

Atoms	GIAO DFT Monte-Carlo[c]	Monte-Carlo st. dev.[d]	NMR fitting[e]
4-(1,2,3), 8-(10,11,12) (CHF proton to CH_3)	6.6	0.5	6.3
5-(1,2,3), 9-(10,11,12) (fluorine to CH_3)	27.0	2.4	23.5
4-6, 8-6 (CHF proton to CH_2, *syn*[a])	5.0	0.5	5.1
4-7, 8-7 (CHF proton to CH_2, *anti*[a])	6.5	0.6	7.0
5-7, 9-7 (F to CH_2, *syn*[a])	24.4	2.5	16.9
5-6, 9-6 (F to CH_2, *anti*[a])	27.0	2.9	25.0
4-5, 8-9 (F to H, geminal)	55.2	4.9	48.0
5-9 (between the F spins)	2.4	0.4	1.6
6-7 (geminal)[b]	−26.6	2.3	−14.5

[a] as shown on the zigzag structure. [b] Coupling dominated by non-contact terms, DFT is known to be a poor predictor.[12] [c] Ensemble average *J*-couplings.[d] Does not include systematic errors of DFT. [e] Experimental fitted *J*-couplings.

6.7 Table S9: chemical shifts of *anti*-3,5-difluoroheptane (14), obtained by fitting experimental data.

anti-3,5-difluoroheptane **14**

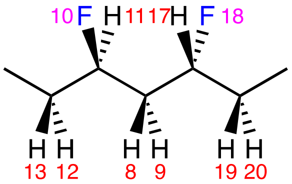

Atoms	Experimental chemical shift / ppm
CH_3 groups	1.0092
12, 19 (outer CH_2, *anti*[a] to F)	1.6370
13, 20 (outer CH_2, *syn*[a] from F)	1.6942
11, 17 (CHF protons)	4.6834
8, 9 (geminal)	1.7970
10, 18 (fluorines)	−184.1865

[a] as shown on the zigzag structure.

6.8 Table S10: *anti*-3,5-difluoroheptane (14), *J*-couplings (Hz).

Atoms	GIAO DFT Monte-Carlo[c]	Monte-Carlo st. dev.[d]	NMR fitting[e]
19-20, 12-13 (within outer CH_2)	−26.0[a]	5.1	−14.44
17-8, 11-9 (CHF proton to inner CH_2, *syn*[a])	3.1	0.5	2.18
17-9, 11-8 (CHF proton to inner CH_2, *anti*[a])	8.4	1.9	10.06
17-19, 11-12 (CHF proton to outer CH_2, *syn*[a])	5.7	1.5	4.53
17-20, 11-13 (CHF proton to outer CH_2, *anti*[a])	6.2	1.5	7.57
8-9 (inner CH_2, geminal)	−26.9[a]	7.6	−15.12
10-8, 18-9 (F to inner CH_2, *syn*[a])	20.9	4.4	14.05
10-9, 18-8 (F to inner CH_2, *anti*[a])	37.8	9.1	38.01
10-11, 18-17 (F to H, geminal)	56.4	11.4	49.63
10-12, 18-19 (F to outer CH_2, *anti*[a])	24.5	6.2	27.79
10-13, 18-20 (F to outer CH_2, *syn*[a])	24.8	4.9	18.43
10-18 (between the F spins)[b]	3.8	0.8	1.23

[a] as shown on the zigzag structure. [b] Coupling dominated by non-contact terms, DFT is known to be a poor predictor.[12] [c] Ensemble average *J*-couplings.[d] Does not include systematic errors of DFT. [e] Experimental fitted *J*-couplings.

6.9 Table S11. chemical shifts of *syn*-3,5-difluoroheptane (15), obtained by fitting experimental data.

syn-3,5-difluoroheptane **15**

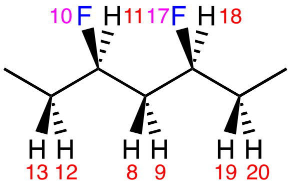

Atoms	Experimental chemical shift / ppm
14, 15, 16, 21, 22, 23 (CH_3 groups)	1.0189
13, 19 (outer CH_2, *syn*[a] to F)	1.7132
12, 20 (outer CH_2, *anti*[a] from F)	1.7018
11, 18 (CHF protons)	4.6138
8 (middle CH_2, *syn*[a] to F)	2.0878
9 (middle CH_2, *anti*[a] from F)	1.8445
10, 17 (fluorines)	−181.9705

[a] as shown on the zigzag structure.

6.10 Table S12. *syn*-3,5-difluoroheptane (15), *J*-couplings (Hz).

Atoms	GIAO DFT Monte-Carlo[c]	Monte-Carlo st. dev.[d]	NMR fitting[e]
12-13, 19-20 (outer CH_2, geminal)	−26.3[a]	3.2	−13.81
9-11, 9-18 (CHF proton to inner CH_2, *syn*[a])	5.4	0.8	4.89
8-11, 8-18 (CHF proton to inner CH_2, *anti*[a])	6.8	1.0	7.08
11-12, 18-20 (CHF proton to outer CH_2, *syn*[a])	4.5	0.6	4.44
11-13, 18-19 (CHF proton to outer CH_2, *anti*[a])	7.3	1.0	7.73
8-9 (within inner CH_2)	−26.62[a]	4.5	−14.79
8-10, 8-17 (F to inner CH_2, *syn*[a])	21.4	2.7	18.33
9-10, 9-17 (F to inner CH_2, *anti*[a])	28.0	4.2	25.83
10-11, 17-18 (F to H, geminal)	55.9	6.9	48.57
10-13, 17-19 (F to outer CH_2, *syn*[a])	21.8	3.2	17.20
10-12, 17-20 (F to outer CH_2, *anti*[a])	32.1	4.8	30.49
10-17 (between the F spins)[b]	1.9	0.6	1.86

[a] as shown on the zigzag structure. [b] Coupling dominated by non-contact terms, DFT is known to be a poor predictor.[12] [c] Ensemble average *J*-couplings. [d] Does not include systematic errors of DFT. [e] Experimental fitted *J*-couplings.

7 Table S13: Comparison between the populations derived from minimum energy calculations and relaxed potential energy scans

		Minimum energy calculations (Tables S1, S2)	Relaxed potential energy scan (Figure 7)
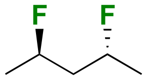 **12**	AA	85%	68%
	AG/GA	6%	11%
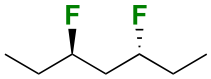 **14**	AA	90%	72%
	AG/GA	4%	9%
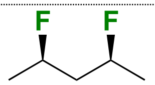 **13**	AG$^-$/GA	29%	25%
	G$^-$A/AG	16%	17%
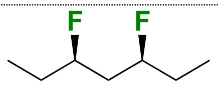 **15**	AG$^-$/GA	26%	30%
	G$^-$A/AG	20%	13%

8 Figure S27: Calculations of %antiperiplanar for the dihedrals in the *J*-value analysis as in Table 5 in the manuscript (in $CHCl_3$)

8.1 Inner dihedrals

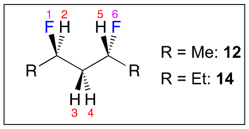

% antiperiplanar

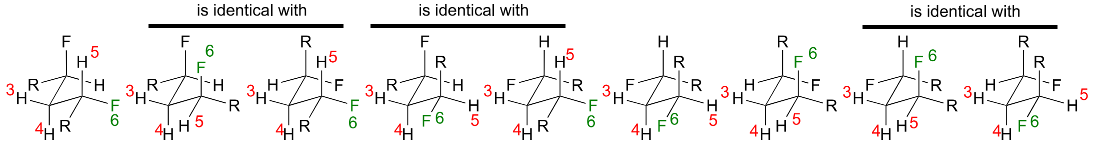

Dihedral:	*AA*	*AG*$^-$	*G*$^-$*A*	*AG*	*GA*	*GG*	*G*$^-$*G*$^-$	*GG*$^-$	*G*$^-$*G*	total (%$_{app}$ value)
H4 - H5	Me: 84.8%<br>Et: 89.7%		Me: 0.5%<br>Et: 0.5%		Me: 6.3%<br>Et: 4.1%					Me: 91.6%<br>Et: 94.5%
H3 - H5				Me: 6.3%<br>Et: 4.1%		Me: 1.3%<br>Et: 0.9%			Me: 0.0%<br>Et: 0.0%	Me: 7.6%<br>Et: 5.0%
H3 - F6	Me: 84.8%<br>Et: 89.7%		Me: 0.5%<br>Et: 0.5%		Me: 6.3%<br>Et: 4.1%					Me: 91.6%<br>Et: 94.5%
H4 - F6		Me: 0.5%<br>Et: 0.5%					Me: 0.2%<br>Et: 0.2%	Me: 0.0%<br>Et: 0.0%		Me: 0.7%<br>Et: 0.7%

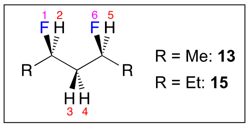

% antiperiplanar

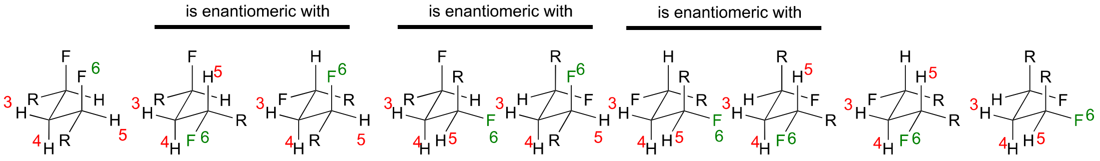

Dihedral:	*AA*	*AG*$^-$	*GA*	*AG*	*G*$^-$*A*	*GG*	*G*$^-$*G*$^-$	*GG*$^-$	*G*$^-$*G*	total (%$_{app}$ value)
H3 - H5	Me: 5.6%<br>Et: 4.9%		Me: 28.8%<br>Et: 26.0%		Me: 15.5%<br>Et: 19.9%					Me: 49.9%<br>Et: 50.8%
H4 - H5		Me: 28.8%<br>Et: 26.0%					Me: 3.0%<br>Et: 1.6%	Me: 0.0%<br>Et: 0.0%		Me: 31.8%<br>Et: 27.6%
H4 - F6	Me: 5.6%<br>Et: 4.9%		Me: 28.8%<br>Et: 26.0%		Me: 15.5%<br>Et: 19.9%					Me: 49.9%<br>Et: 50.8%
H3 - F6				Me: 15.5%<br>Et: 19.9%		Me: 3.0%<br>Et: 1.6%			Me: 0.0%<br>Et: 0.0%	Me: 18.5%<br>Et: 21.5%

8.2 Figures S28, S29: outer dihedrals (heptanes)

8.2.1 *Anti*-3,5-difluoroheptane (Figure S28)

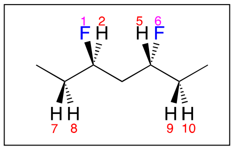

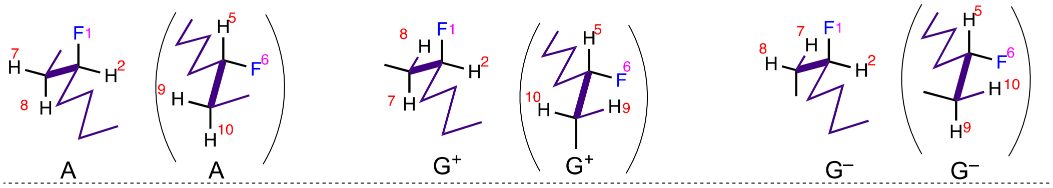

Antiperiplanar dihedrals: H2 – H7 (=H5 – H10) ... H2 – H8 (=H5 – H9)

H8 – F1 (=H9 – F6) ... H7 – F1 (=H10 – F6)

		% population with indicated dihedral antiperiplanar			
Conformation	Population[a]	H2 – H8	H2 – H7	H8 – F1	H7 – F1
**G**G⁻XX	0.0				0.0%
**A**G⁻XX	0.4		0.4%	0.4%	
**G⁻**G⁻XX	0.3	0.3%			
**G**AXX	22.5				22.5%
**A**AXX	51.1		51.1%	51.1%	
**G⁻**AXX	20.6	20.6%			
**G**GXX	1.4				1.4%
**A**GXX	3.5		3.5%	3.5%	
**G⁻**GXX	0.0	0.0%			
**Total (%$_{app}$ value)**		**20.9%**	**55.0%**	**55.0%**	**23.9%**

[a] See summary Chart S11

8.2.2 *Syn*-3,5-difluoroheptane (Figure S29)

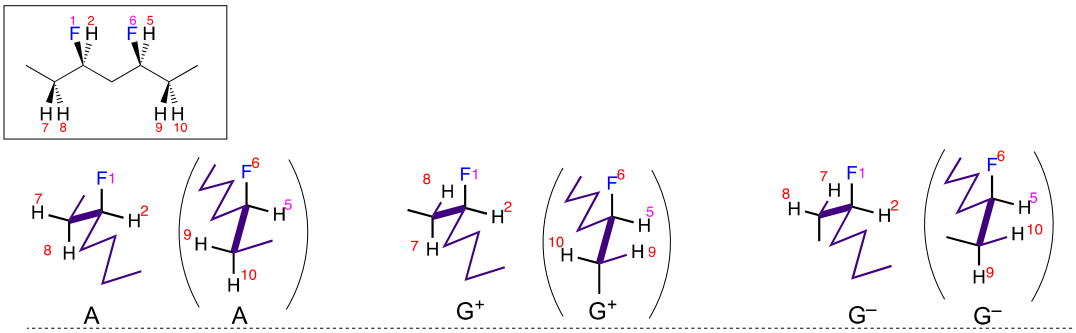

Antiperiplanar dihedrals:

A	A	G+	G+	G−	G−
H2 – H7 (=H5 – H9)			H5 – H10	H2 – H8	
H8 – F1 (=H10 – F6)		H7 – F1			H9 – F6

		% population with indicated dihedral antiperiplanar			
Conformation	Population[a]	H2 – H8	H2 – H7	H8 – F1	H7 – F1
GG-	0.0%	0.0%			
AG-	16.4%		16.4%	16.4%	
G-G-	5.1%				5.1%
GA	9.8%	9.8%			
AA	31.4%		31.4%	31.4%	
G-A	9.5%				9.5%
GG	5.8%	5.8%			
AG	21.8%		21.8%	21.8%	
G-G	0.0%				0.0%
**Total (%$_{app}$ value)**		**15.6%**	**69.6%**	**69.6%**	**14.6%**

See Summary Chart S12

9 Table S14: full coupling constant analysis (extended Table 5 in manuscript)

($CDCl_3$, 298 K).

anti-3,5-difluoropentane **12**

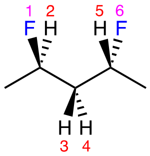

syn-3,5-difluoropentane **13**

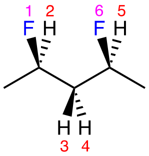

anti-3,5-difluoroheptane **14**

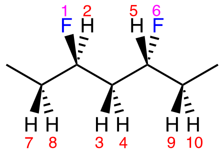

syn-3,5-difluoroheptane **15**

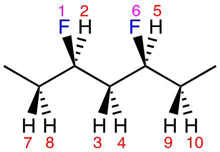

anti-2,4-difluoropentane **12**

Entry	Coupling[a]	J (Hz)[b]	$\%_{app}$
1	$^{3}J_{\mathrm{H2-H3}} = {}^{3}J_{\mathrm{H4-H5}}$	9.8	92±6%
2	$^{3}J_{\mathrm{H2-H4}} = {}^{3}J_{\mathrm{H3-H5}}$	2.4	8±1%
3	$^{3}J_{\mathrm{F1-H4}} = {}^{3}J_{\mathrm{H3-F6}}$	36.4	92±6%
4	$^{3}J_{\mathrm{F1-H3}} = {}^{3}J_{\mathrm{H4-F6}}$	13.7	1.0±0.2%
5	$^{2}J_{\mathrm{F1-H2}} = {}^{2}J_{\mathrm{H5-F6}}$	49.3	-
6	$^{2}J_{\mathrm{H3-H4}}$	-15.1	-
7	$^{4}J_{\mathrm{F-F}}$	1.6	-

syn-2,4-difluoropentane **13**

Entry	Coupling[a]	J (Hz)[b]	$\%_{app}$
8	$^{3}J_{\mathrm{H2-H3}} = {}^{3}J_{\mathrm{H3-H5}}$	7.0	50±7%
9	$^{3}J_{\mathrm{H2-H4}} = {}^{3}J_{\mathrm{H4-H5}}$	5.1	32±5%
10	$^{3}J_{\mathrm{F1-H4}} = {}^{3}J_{\mathrm{H4-F6}}$	25.0	50±7%
11	$^{3}J_{\mathrm{F1-H3}} = {}^{3}J_{\mathrm{H3-F6}}$	16.9	19±3%
12	$^{2}J_{\mathrm{H2-F1}} = {}^{2}J_{\mathrm{H5-F6}}$	48.0	-
13	$^{2}J_{\mathrm{H3-H4}}$	-14.5	-
14	$^{4}J_{\mathrm{F-F}}$	1.6	-

anti-3,5-difluoroheptane **14**

Entry	Coupling[a]	J (Hz)[b]	$\%_{app}$
15	$^{3}J_{\mathrm{H2-H3}} = {}^{3}J_{\mathrm{H4-H5}}$	10.1	95±5%
16	$^{3}J_{\mathrm{H2-H4}} = {}^{3}J_{\mathrm{H3-H5}}$	2.2	5±1%
17	$^{3}J_{\mathrm{F1-H4}} = {}^{3}J_{\mathrm{H3-F6}}$	38.0	95±5%
18	$^{3}J_{\mathrm{F1-H3}} = {}^{3}J_{\mathrm{H4-F6}}$	14.1	1.0±0.2%
19	$^{3}J_{\mathrm{H2-H8}} = {}^{3}J_{\mathrm{H5-H9}}$	4.5	21±3%
20	$^{3}J_{\mathrm{H2-H7}} = {}^{3}J_{\mathrm{H5-H10}}$	7.6	55±8%
21	$^{3}J_{\mathrm{F1-H8}} = {}^{3}J_{\mathrm{F6-H9}}$	27.8	55±8%
22	$^{3}J_{\mathrm{F1-H7}} = {}^{3}J_{\mathrm{F6-H10}}$	18.4	24±4%
23	$^{2}J_{\mathrm{F1-H2}} = {}^{2}J_{\mathrm{H5-F6}}$	50.0	-
24	$^{2}J_{\mathrm{H3-H4}}$	-15.1	-
25	$^{2}J_{\mathrm{H8-H7}} = {}^{2}J_{\mathrm{H9-H10}}$	-14.4	-
26	$^{4}J_{\mathrm{F-F}}$	1.2	-

syn-3,5-difluoroheptane **15**

Entry	Coupling[a]	J (Hz)[b]	$\%_{app}$
27	$^{3}J_{\mathrm{H2-H3}} = {}^{3}J_{\mathrm{H3-H5}}$	7.1	51±7%
28	$^{3}J_{\mathrm{H2-H4}} = {}^{3}J_{\mathrm{H4-H5}}$	4.9	28±5%
29	$^{3}J_{\mathrm{F1-H4}} = {}^{3}J_{\mathrm{H4-F6}}$	25.8	51±7%
30	$^{3}J_{\mathrm{F1-H3}} = {}^{3}J_{\mathrm{H3-F6}}$	18.3	22±4%
31	$^{3}J_{\mathrm{H2-H8}}$, $^{3}J_{\mathrm{H5-H10}}$	4.4	16±3%
32	$^{3}J_{\mathrm{H2-H7}}$, $^{3}J_{\mathrm{H5-H9}}$	7.7	70±8%
33	$^{3}J_{\mathrm{F1-H8}}$, $^{3}J_{\mathrm{F6-H10}}$	30.5	70±8%
34	$^{3}J_{\mathrm{F1-H7}}$, $^{3}J_{\mathrm{6-H9}}$	17.2	15±3%
35	$^{2}J_{\mathrm{F1-H2}} = {}^{2}J_{\mathrm{H5-F6}}$	48.6	-
36	$^{2}J_{\mathrm{H3-H4}}$	-14.8	-
37	$^{2}J_{\mathrm{H8-H7}} = {}^{2}J_{\mathrm{H9-H10}}$	-13.8	-
38	$^{4}J_{\mathrm{F-F}}$	1.9	-

[a] The equivalent dihedral angles due to symmetry are indicated; [b] "Data fitted values" obtained as described above. Accurate to ±0.1 Hz. [c] Sum of the populations of the conformations featuring an antiperiplanar disposition of the atoms of the ^{3}J in question. See Figures S27–29 in the Supporting Information

10 Figure S30: Hydrocarbon chain population changes according to the medium, for pentane, heptane, and the *anti*-substrates 12 and 14

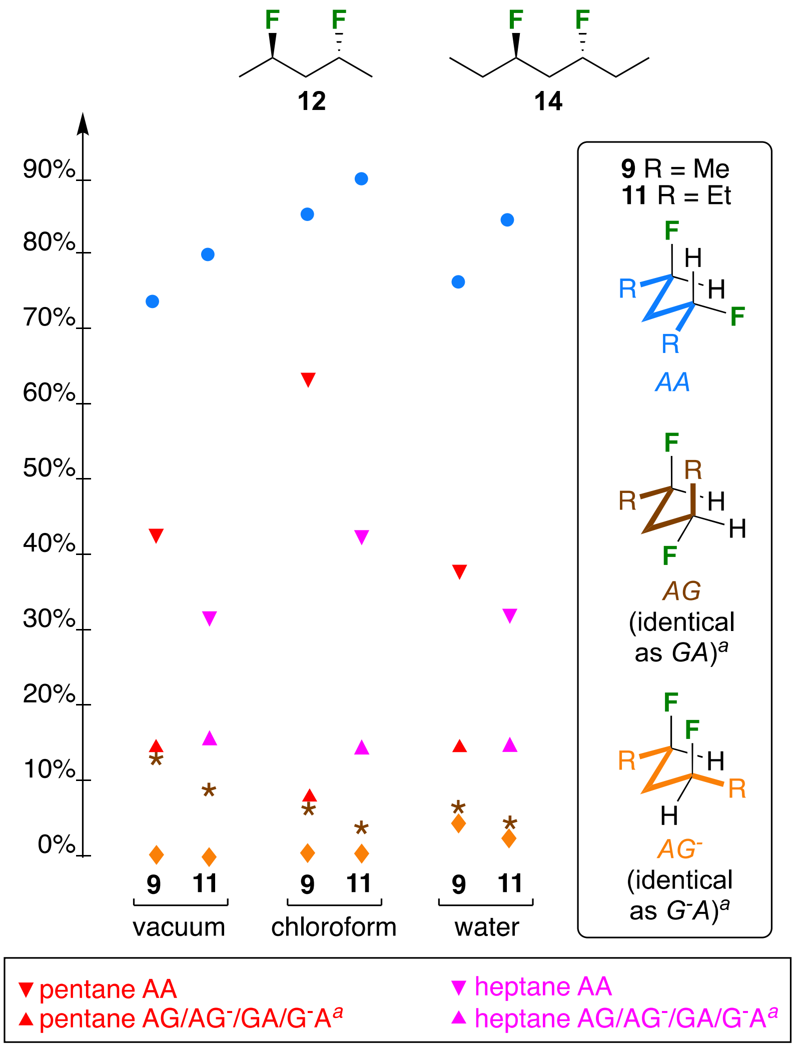

11 Copies of spectra of synthetic intermediates

11.1 3-hydroxyheptan-5-one (SI3)

11.1.1 ^1H NMR ($CDCl_3$, 400 MHz)

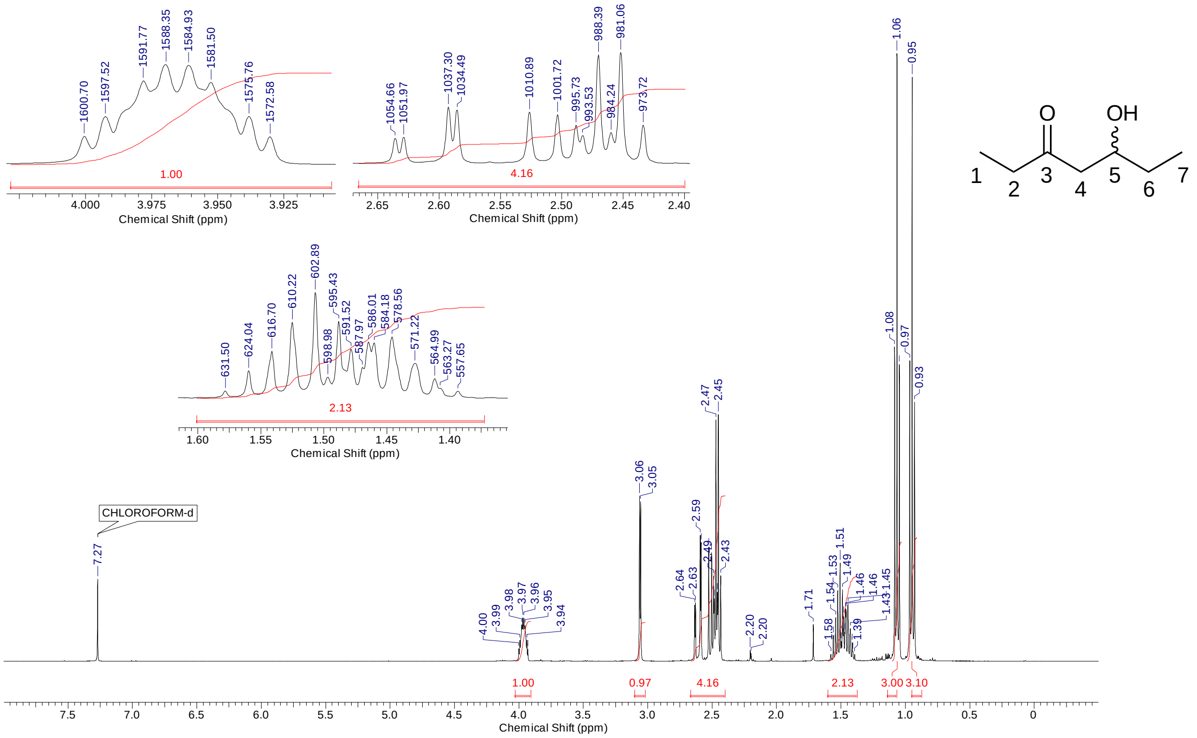

11.1.2 $^{13}C\{1H\}$ NMR ($CDCl_3$, 101 MHz)

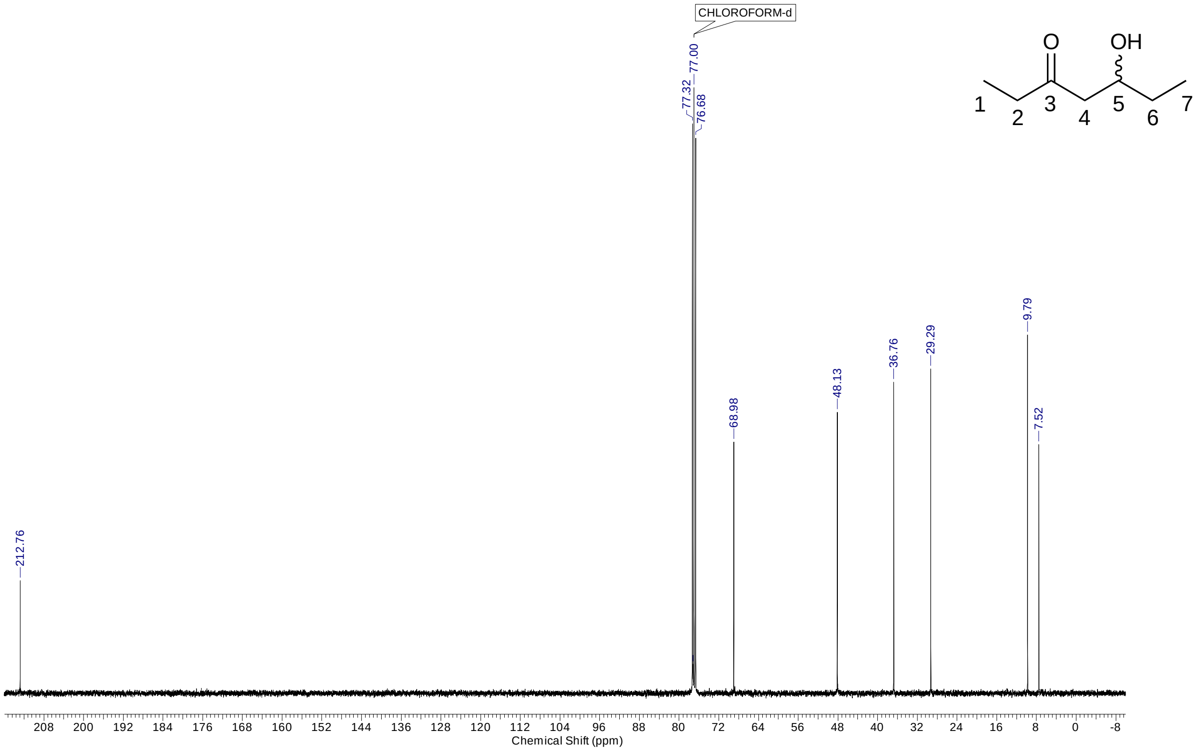

11.2 (±)-*anti*-heptane-3,5-diol (SI4)

11.2.1 ^{1}H NMR ($CDCl_3$, 400 MHz)

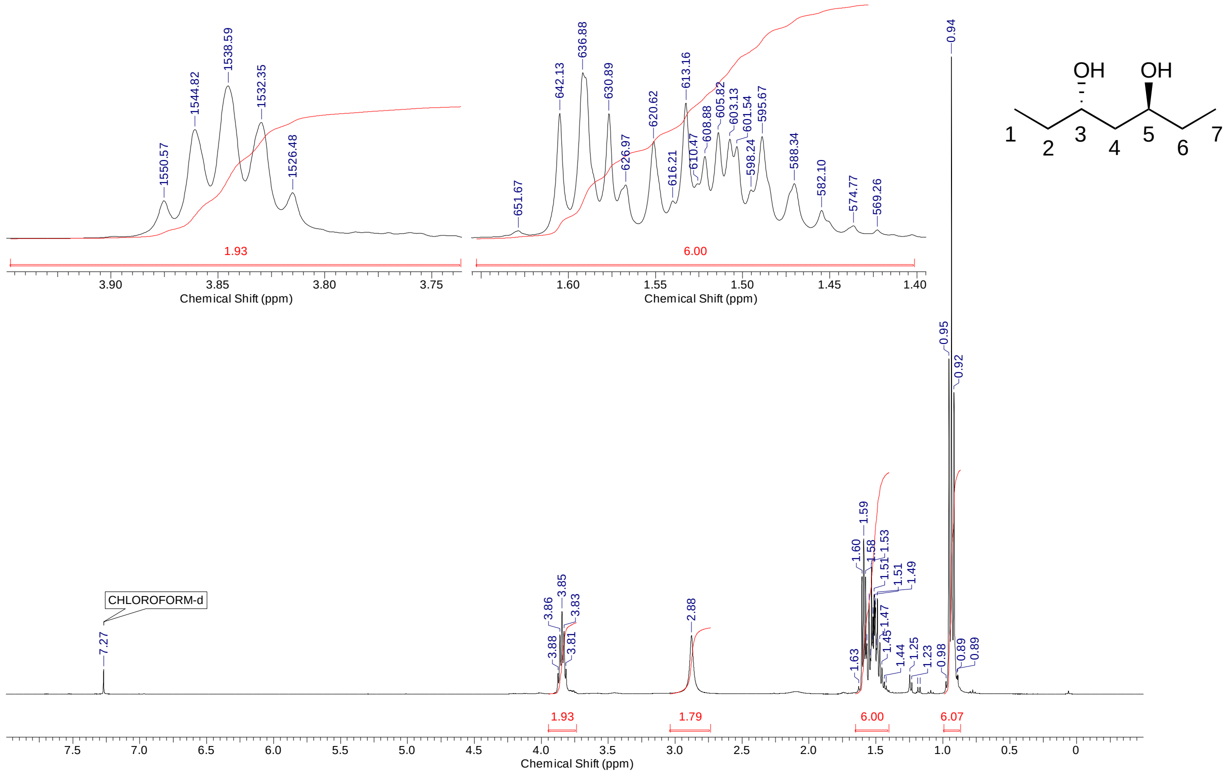

11.2.2 ^{13}C{1H} NMR ($CDCl_3$, 101 MHz)

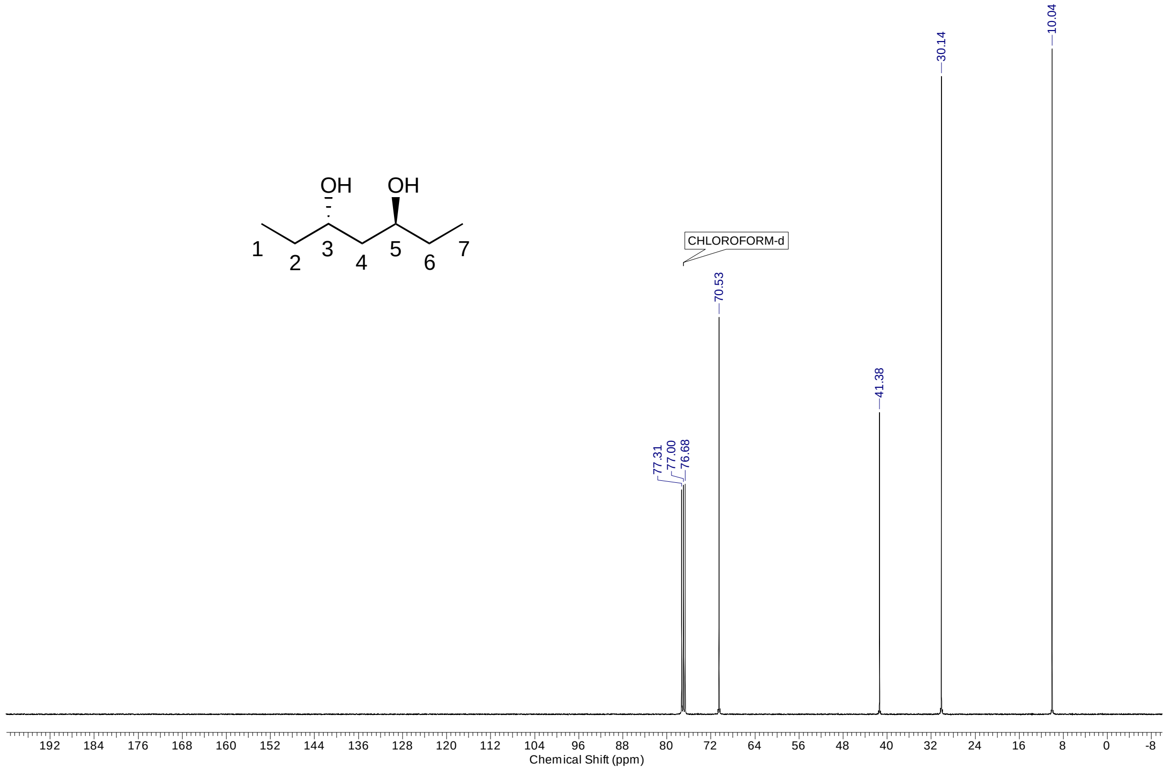

11.3 *syn*-heptane-3,5-diol (SI5)

11.3.1 ^{1}H NMR ($CDCl_3$, 400 MHz)

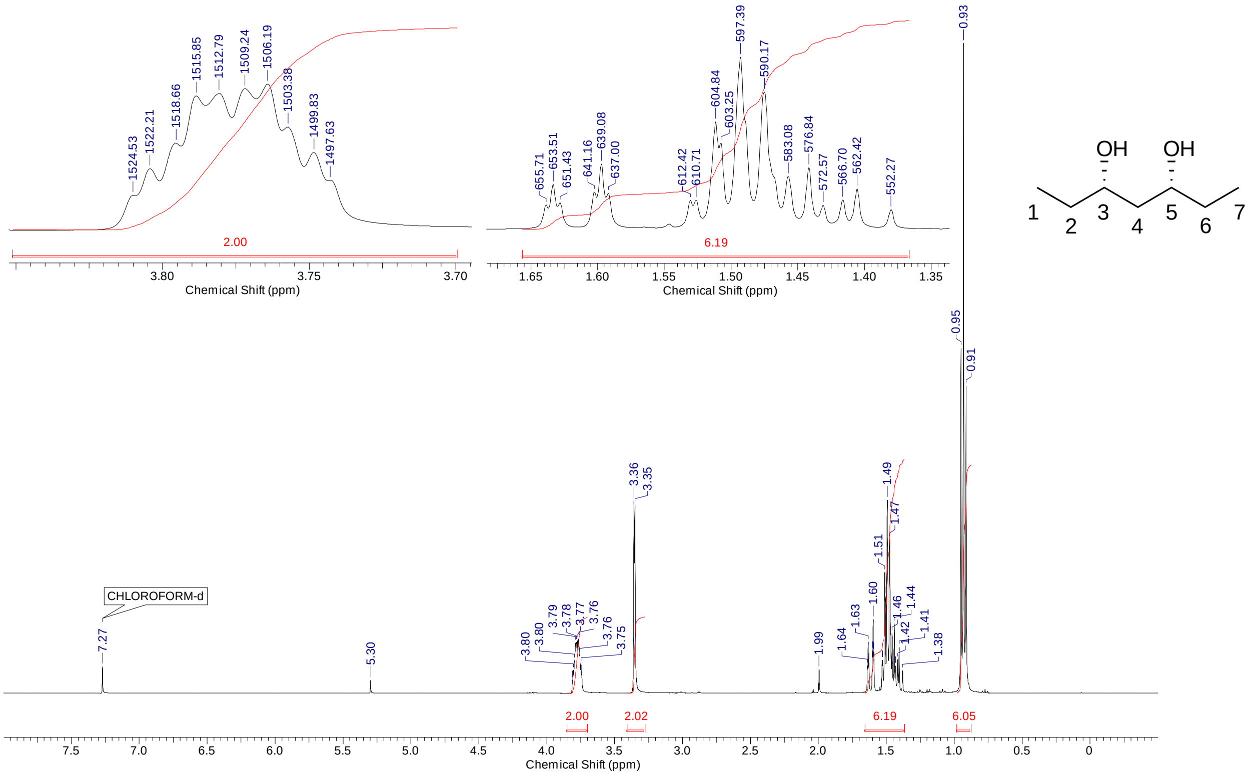

11.3.2 ^{13}C{1H} NMR ($CDCl_3$, 101 MHz)

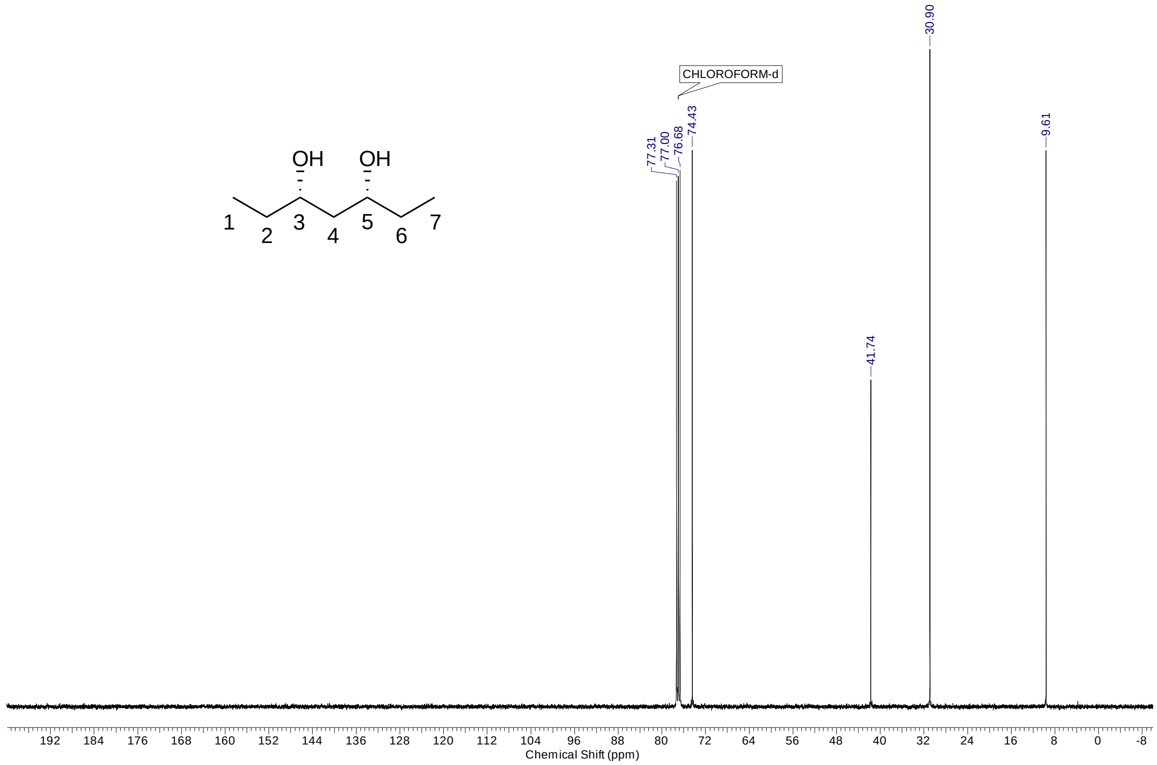

11.4 (±)-*syn*-3-(3'-methylbenzoyloxy)-5-fluoroheptane (SI6)

11.4.1 ^{1}H NMR ($CDCl_3$, 400 MHz)

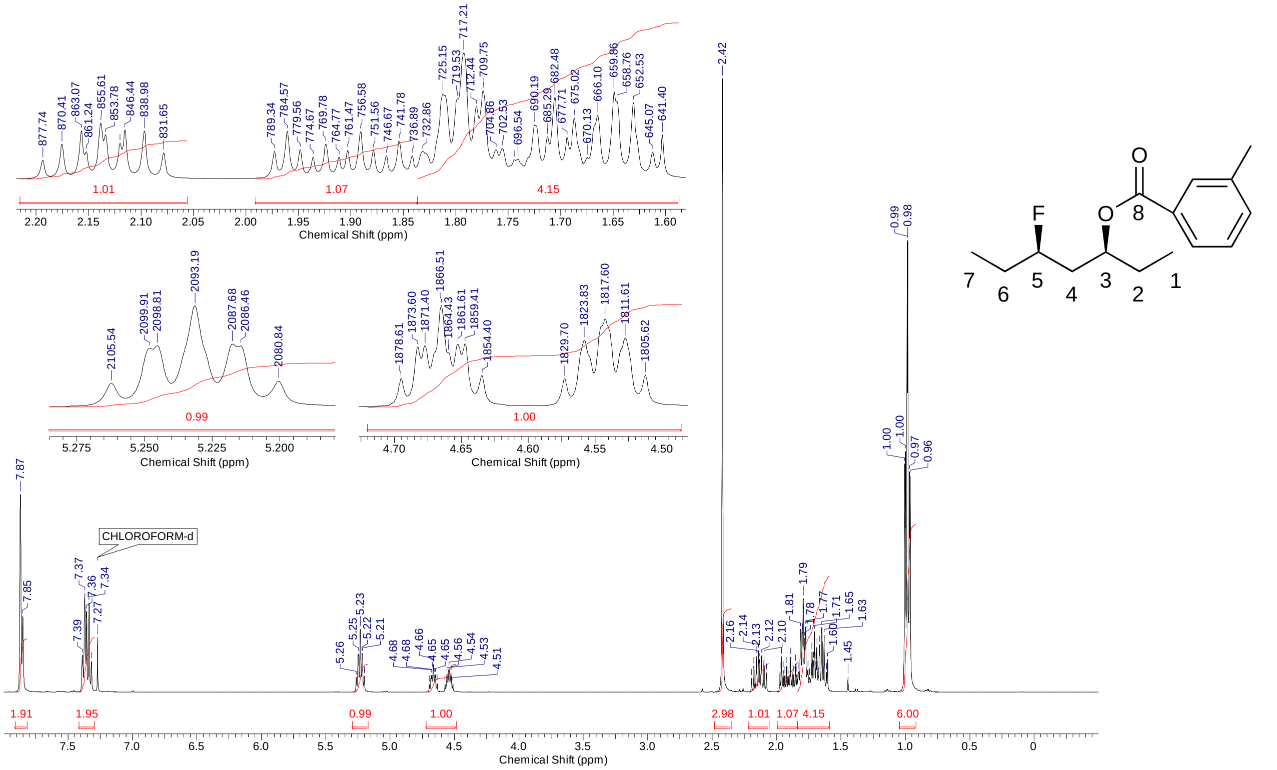

11.4.2 ^{13}C{1H} NMR ($CDCl_3$, 101 MHz)

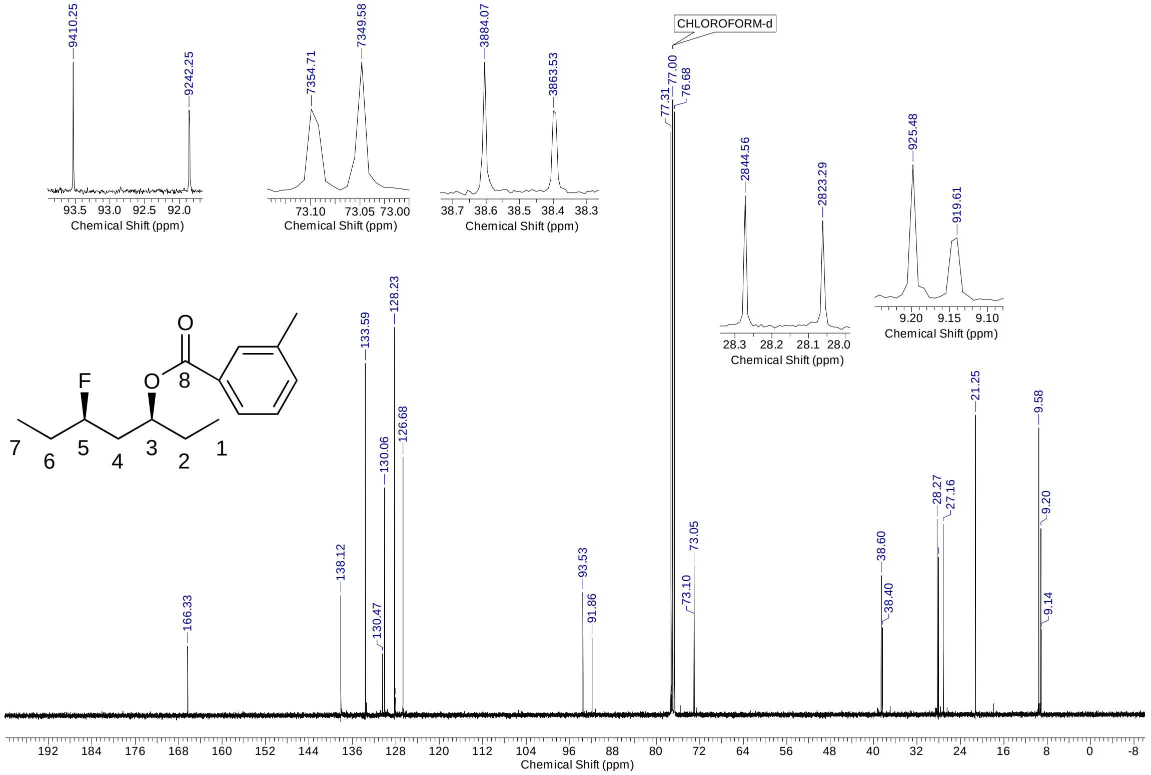

11.4.3 ^{112}F NMR ($CDCl_3$, 376 MHz)

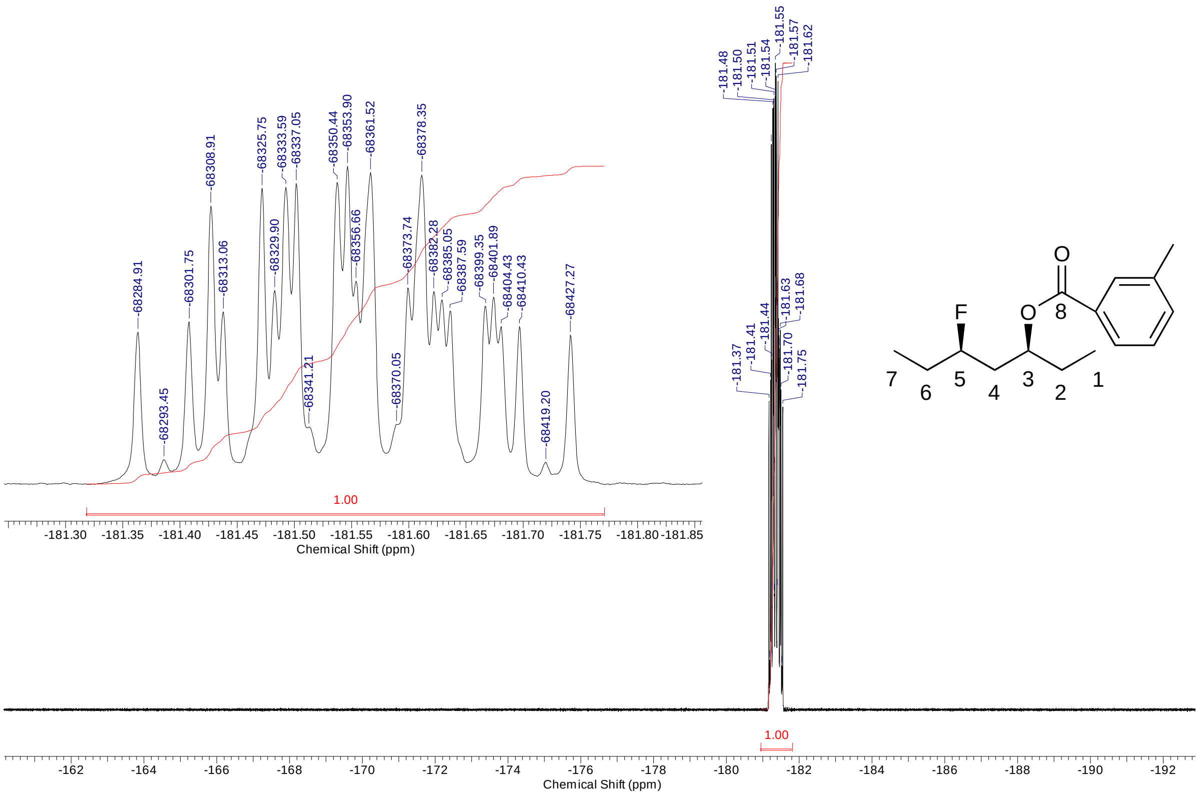

11.4.4 ^{19}F{^{1}H} NMR ($CDCl_3$, 376 MHz)

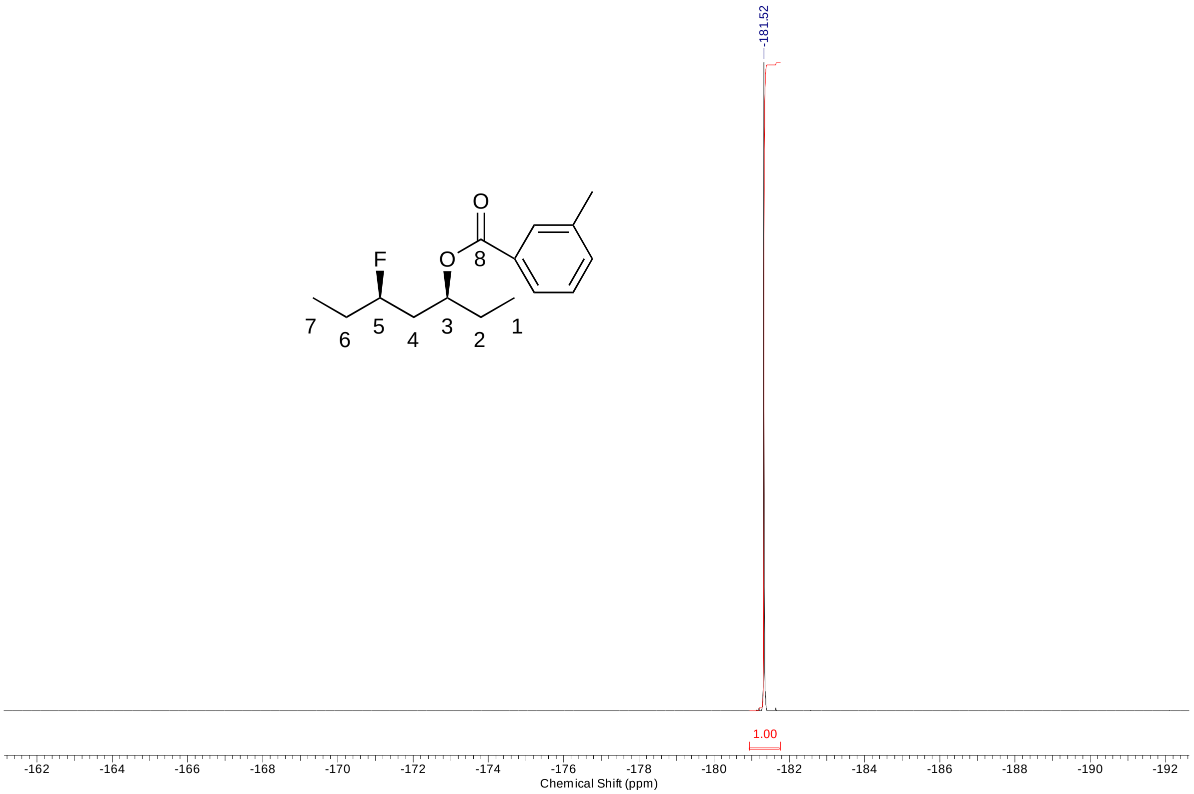

11.5 (±)-*anti*-3-(3'-methylbenzoyloxy)-5-fluoroheptane (SI7)

11.5.1 ^{1}H NMR ($CDCl_3$, 400 MHz)

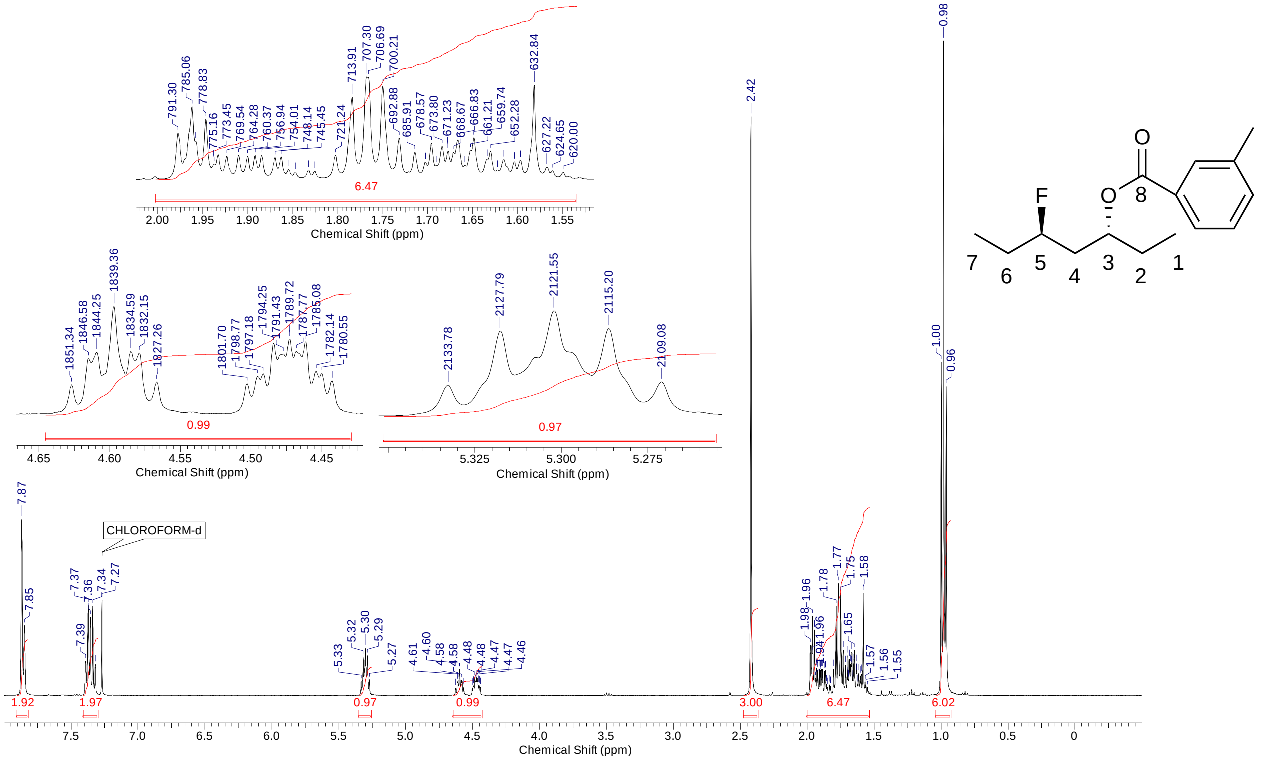

11.5.2 ^{13}C{1H} NMR ($CDCl_3$, 101 MHz)

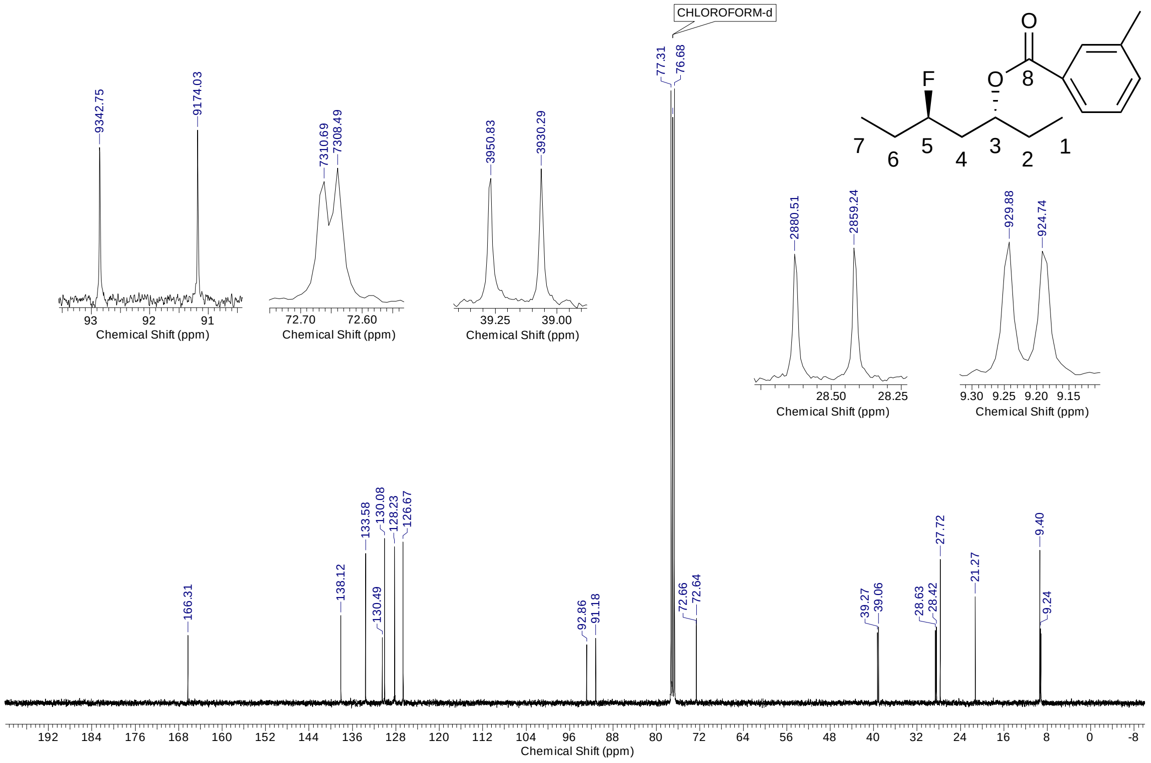

11.5.3 ^{19}F NMR ($CDCl_3$, 376 MHz)

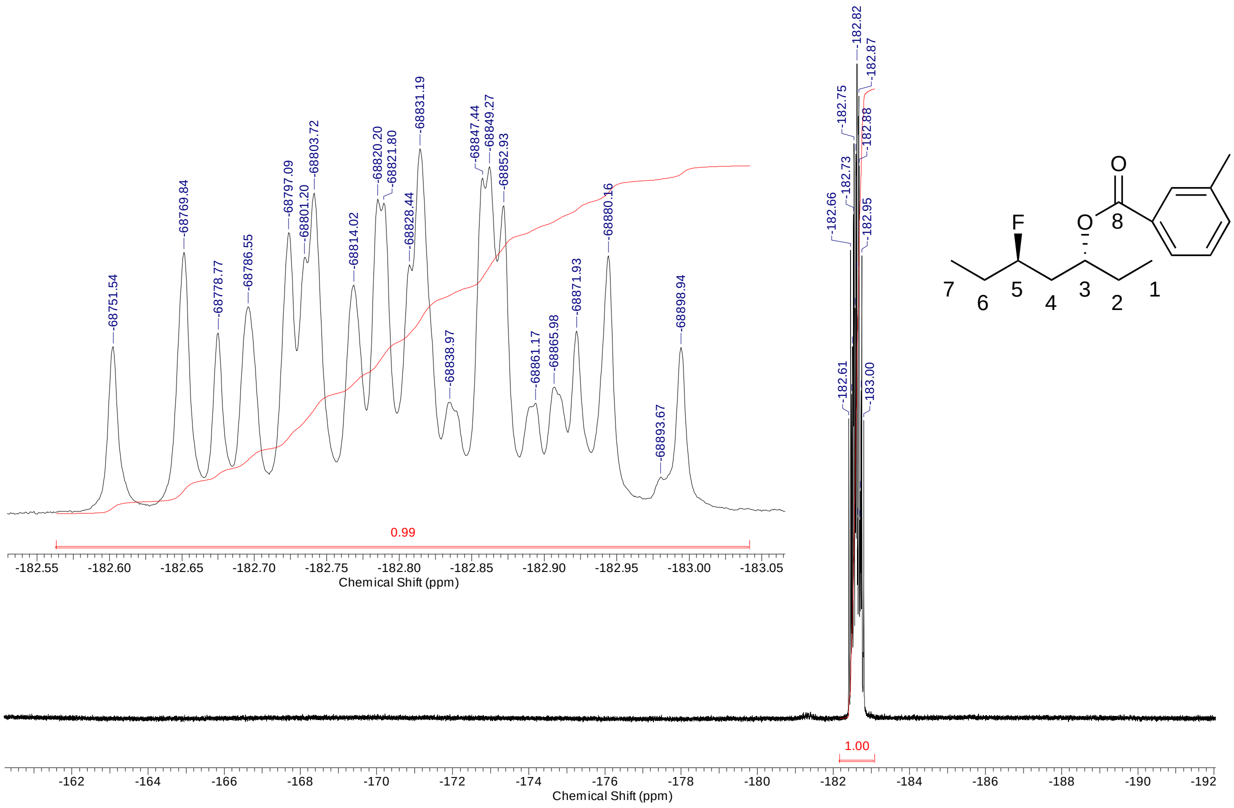

11.5.4 $^{19}F\{^1H\}$ NMR ($CDCl_3$, 376 MHz)

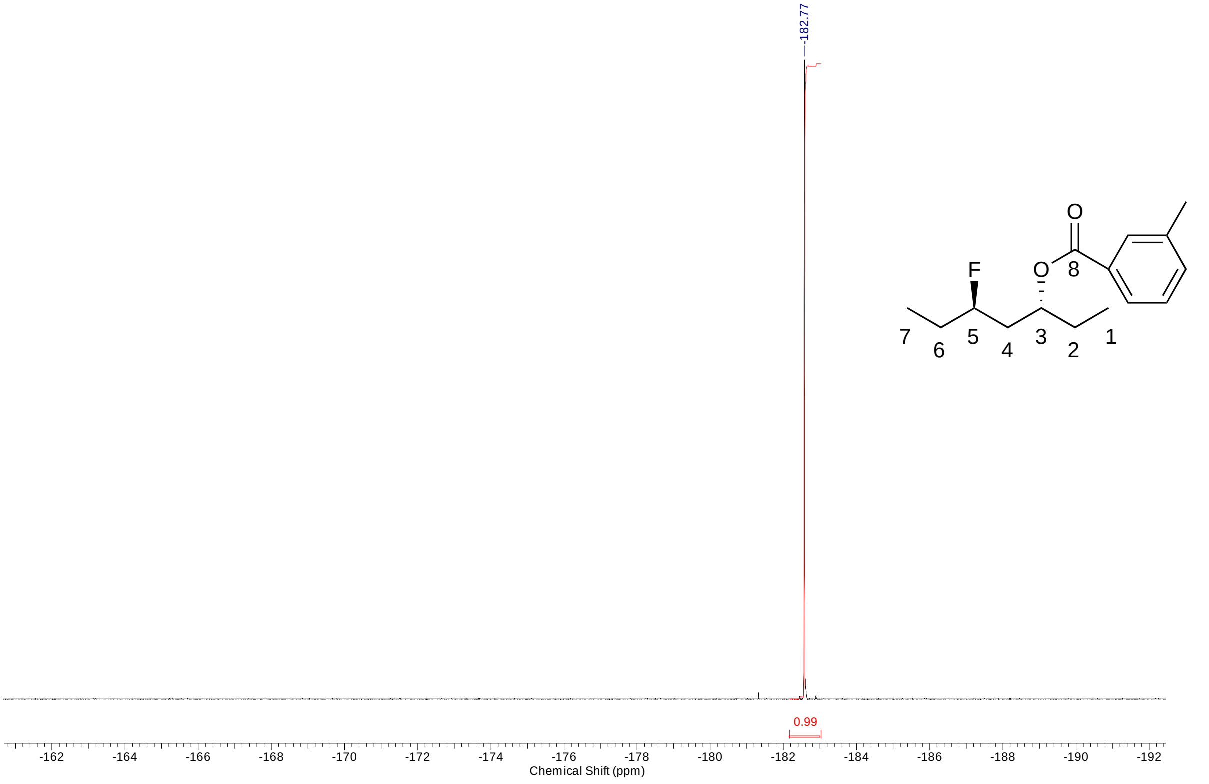

11.6 (±)-*syn*-5-fluoroheptan-3-ol (SI8)

11.6.1 ^{1}H NMR ($CDCl_3$, 500 MHz)

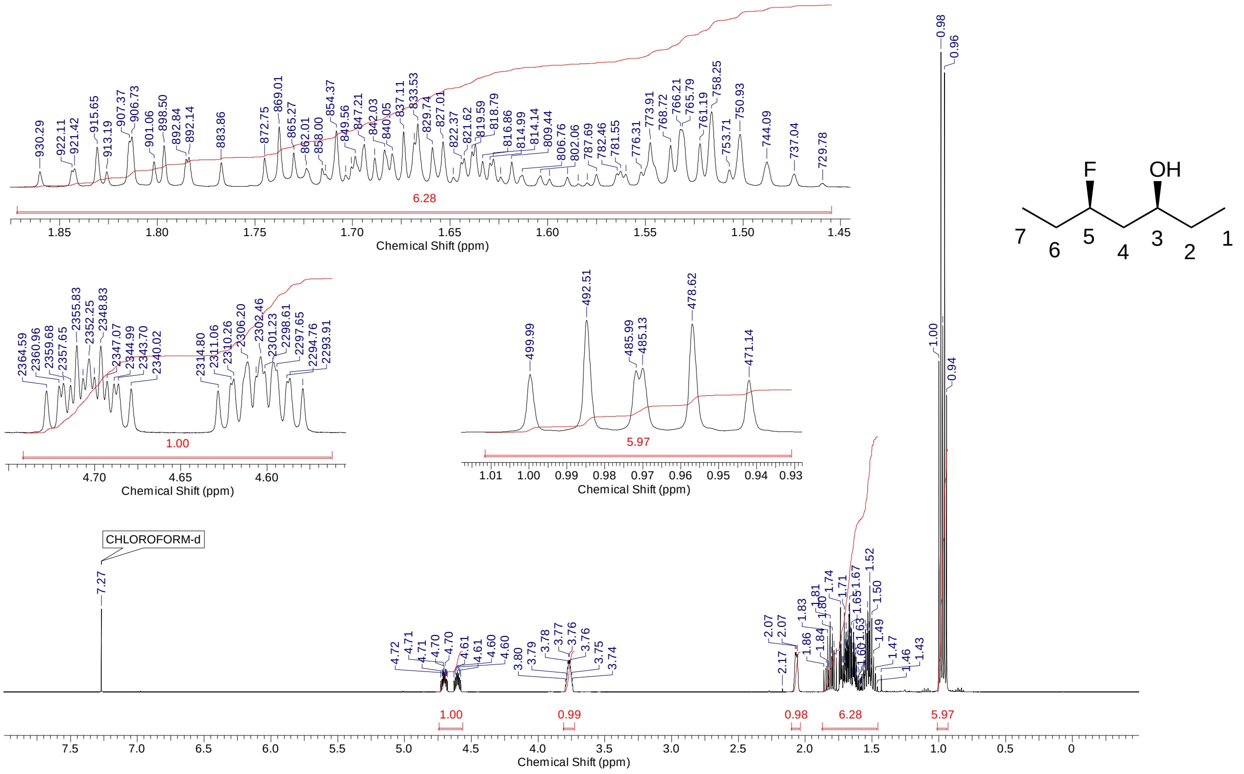

11.6.2 $^{13}C\{1H\}$ NMR ($CDCl_3$, 126 MHz)

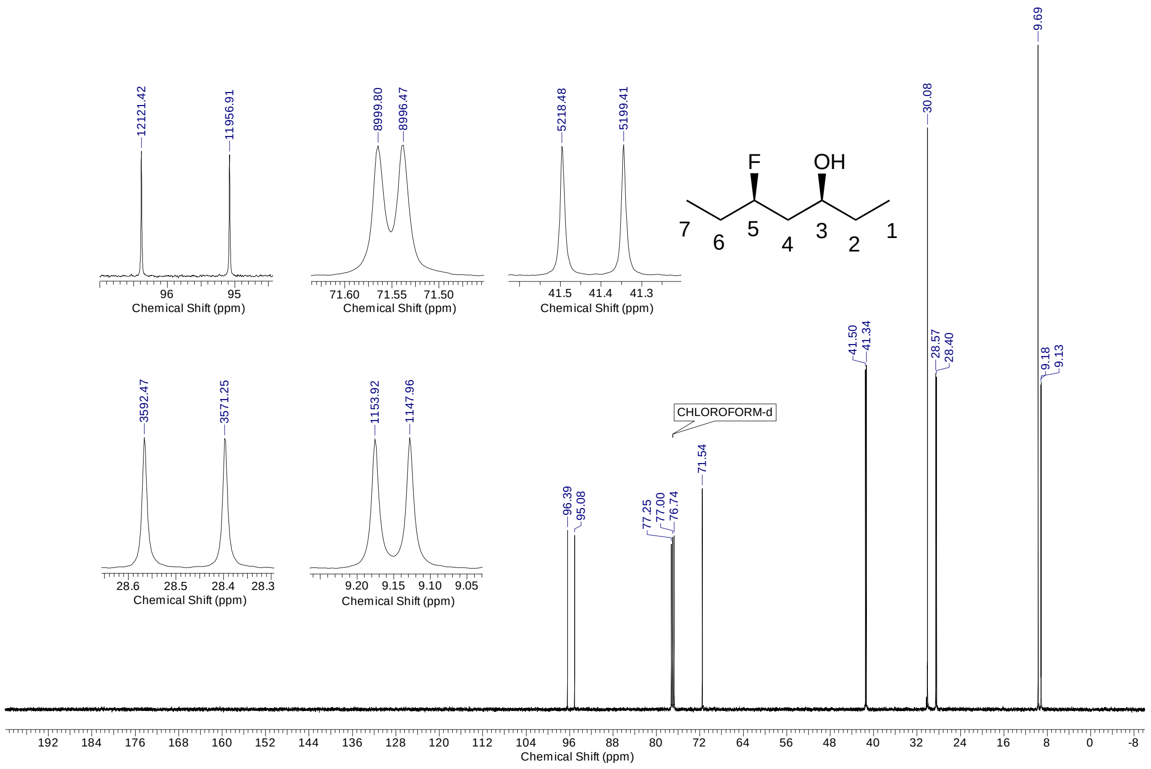

11.6.3 ^{19}F NMR ($CDCl_3$, 471 MHz)

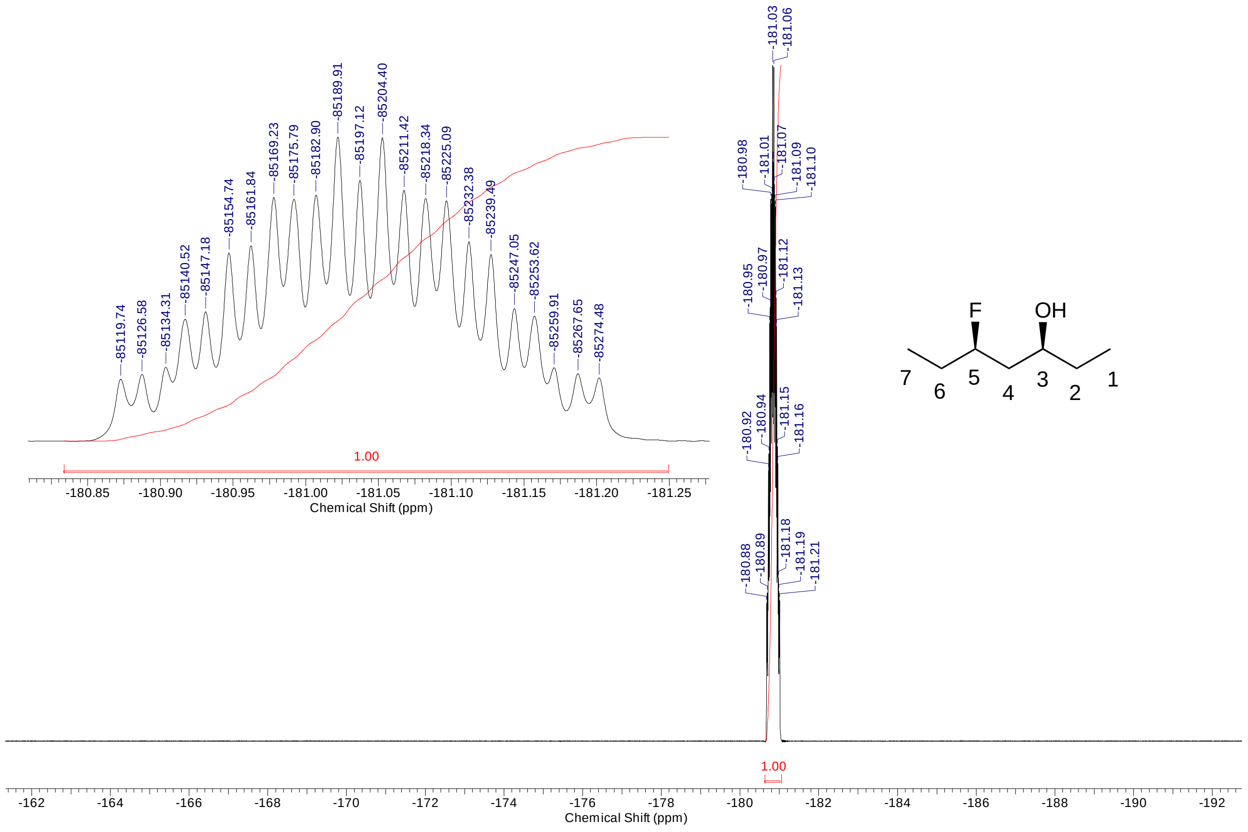

11.6.4 $^{19}F\{^1H\}$ NMR ($CDCl_3$, 471 MHz)

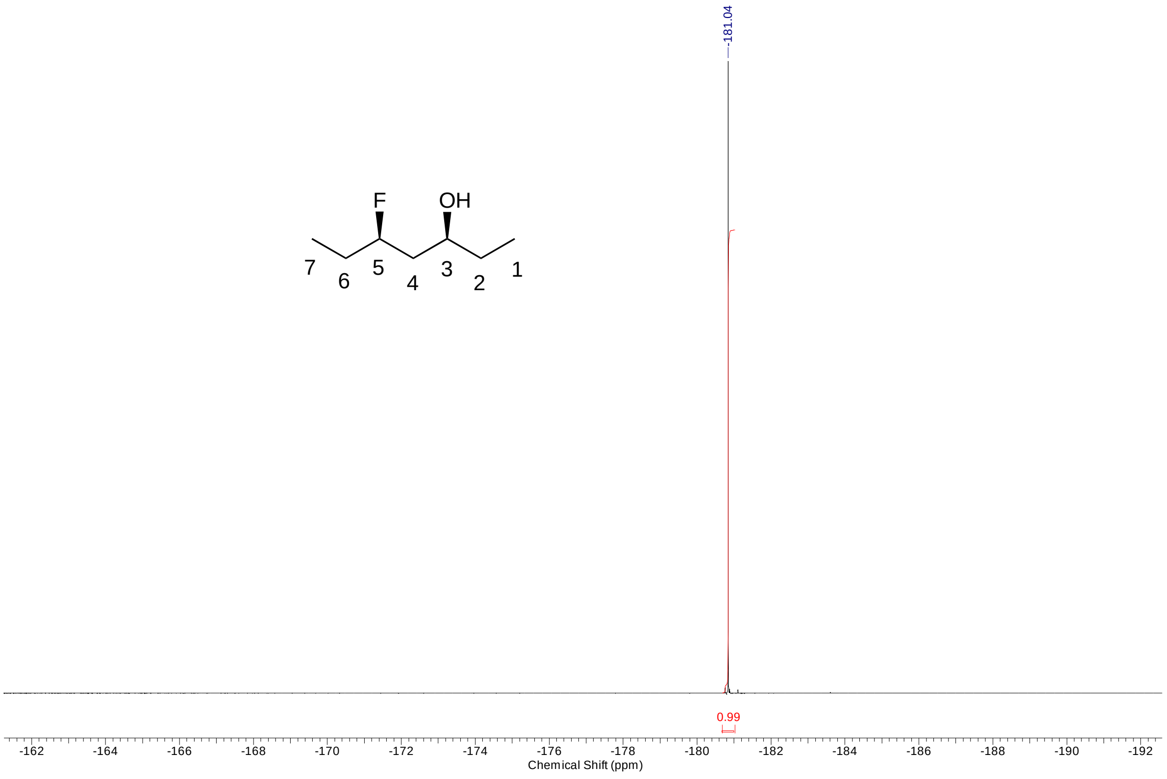

11.7 (±)-*anti*-5-fluoroheptan-3-ol (SI9)

11.7.1 ^{1}H NMR ($CDCl_3$, 500 MHz)

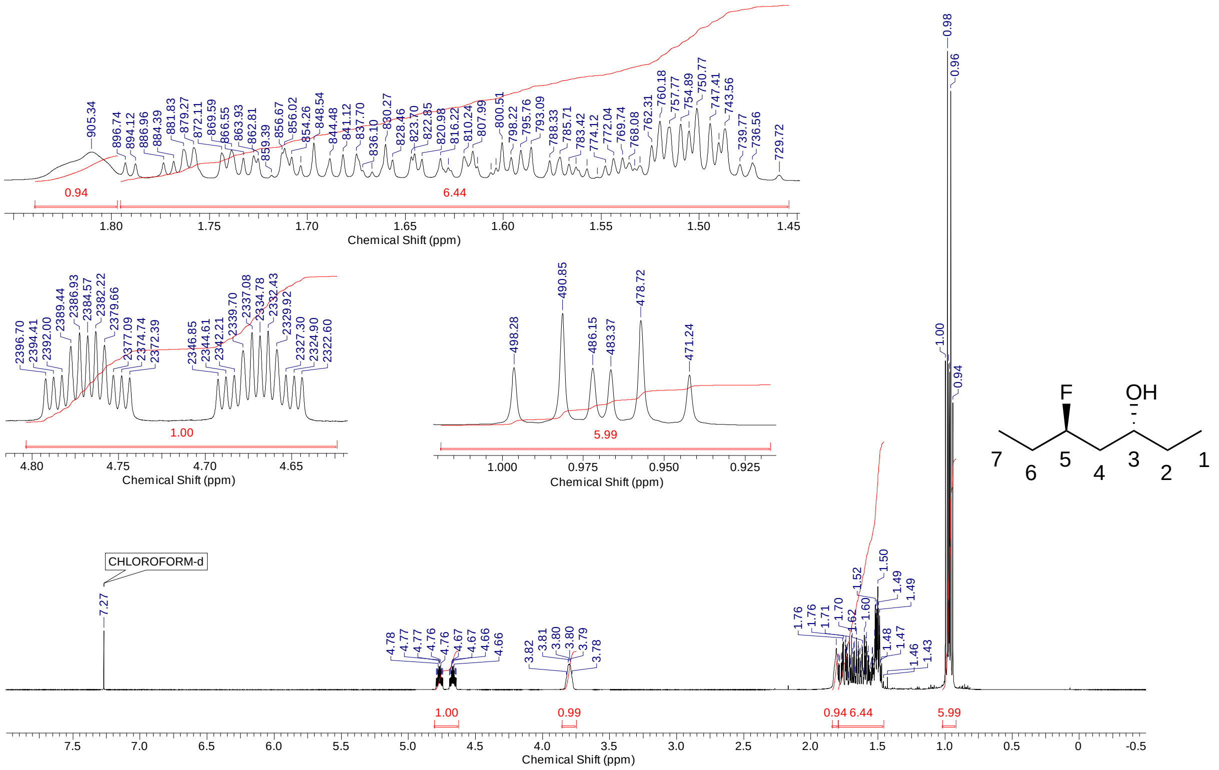

11.7.2 $^{13}C\{1H\}$ NMR ($CDCl_3$, 126 MHz)

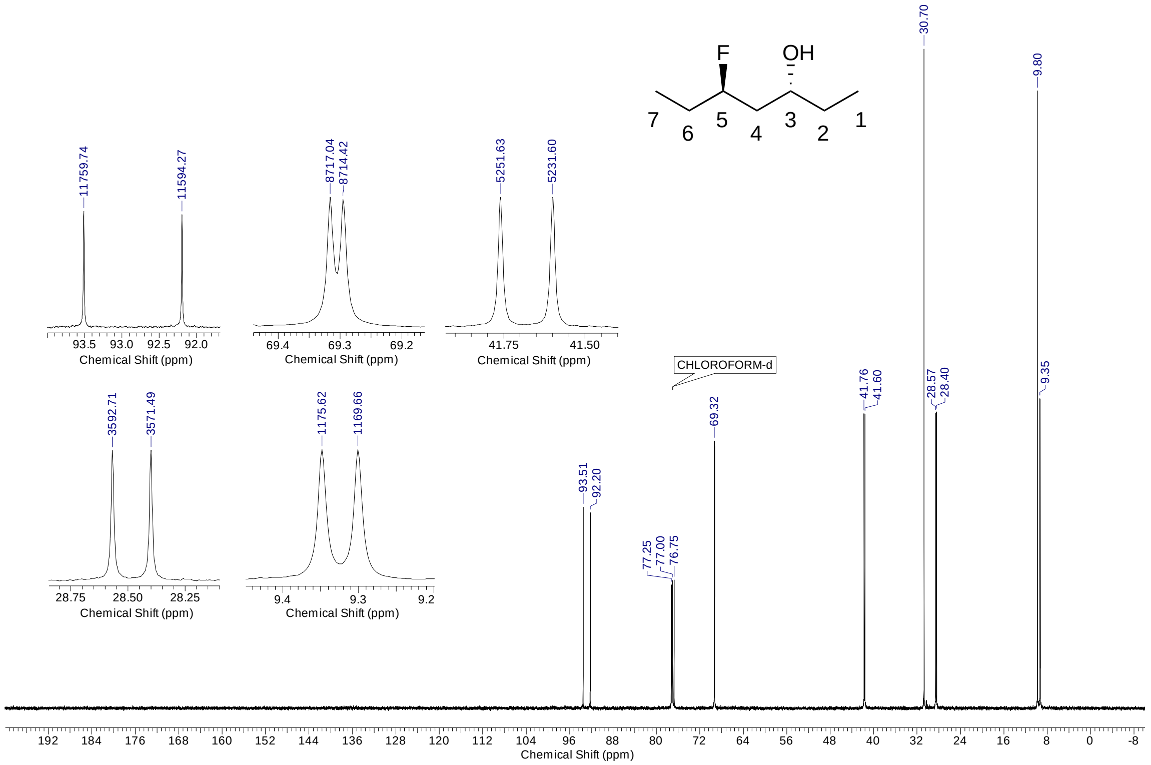

11.7.3 ^{19}F NMR ($CDCl_3$, 471 MHz)

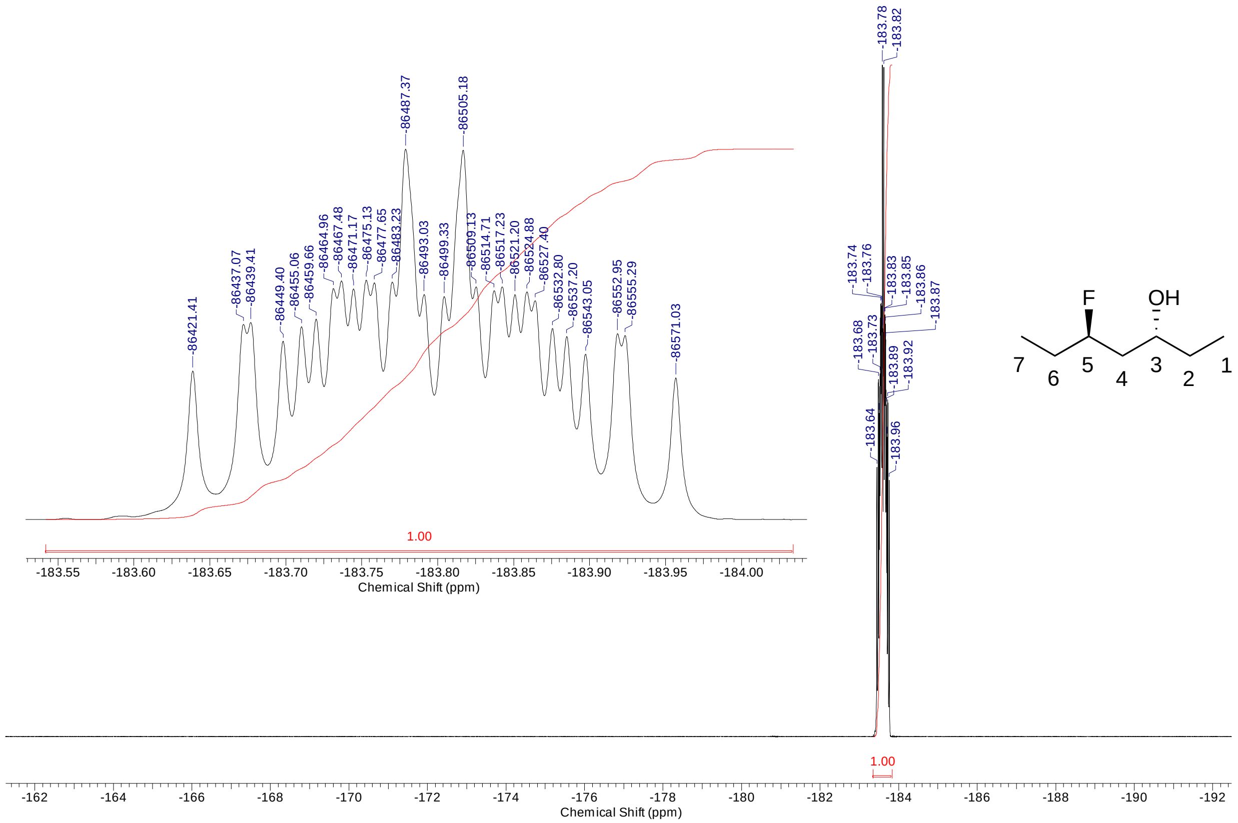

11.7.4 $^{19}F\{^{1}H\}$ NMR ($CDCl_3$, 471 MHz)

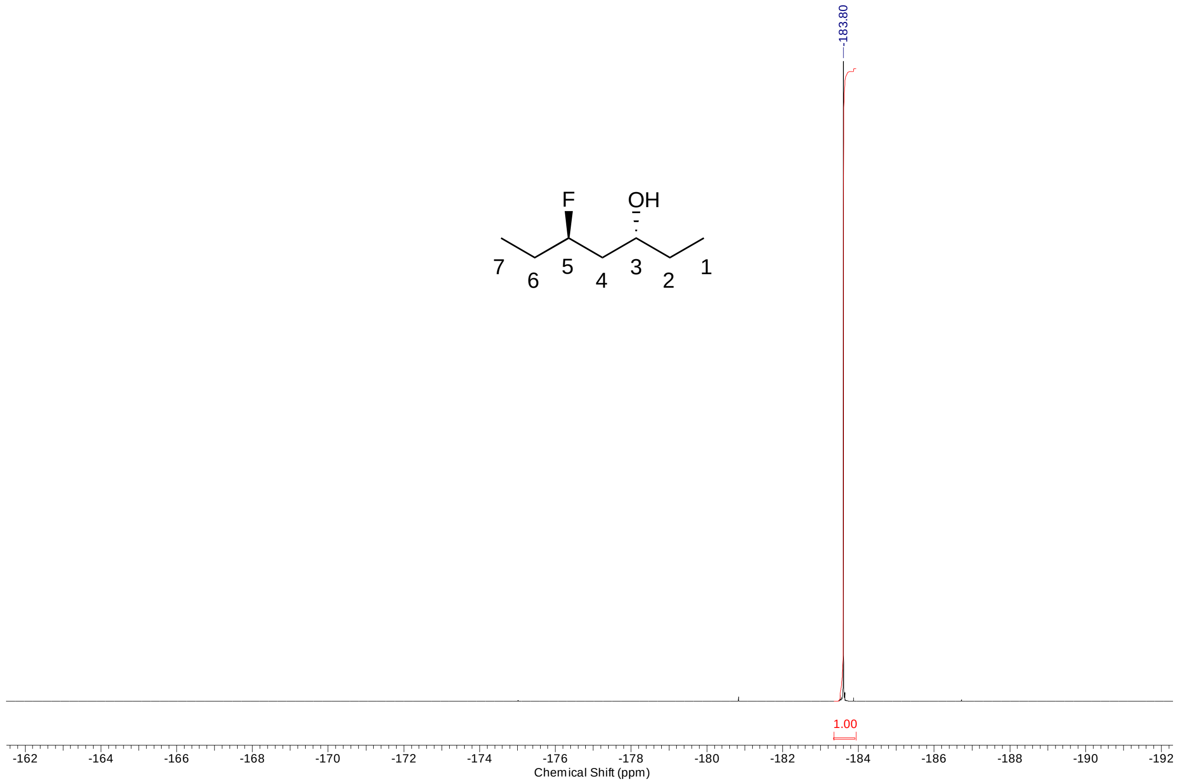

REFERENCES

(1) Chuvatkin, N. N.; Morozova, T. V.; Boguslavskaya, L. S. New Reaction of Substitutive in Situ Fluorination with Chlorine Monofluoride. *Russ. J. Org. Chem.* **1983,** *19*, 990.

(2) Linclau, B.; Peron, F.; Bogdan, E.; Wells, N.; Wang, Z.; Compain, G.; Fontenelle, C. Q.; Galland, N.; Le Questel, J.-Y.; Graton, J. Intramolecular OH•••Fluorine Hydrogen Bonding in Saturated, Acyclic Fluorohydrins: The gamma-Fluoropropanol Motif. *Chem. Eur. J.* **2015,** *21* (49), 17808.

(3) Ravikumar, K. S.; Sinha, S.; Chandrasekaran, S. Diastereoselectivity in the Reduction of Acyclic Carbonyl Compounds with Diisopropoxytitanium(III) Tetrahydroborate. *J. Org. Chem.* **1999,** *64* (16), 5841.

(4) Marinetti, A.; Genêt, J.-P.; Jus, S.; Blanc, D.; Ratovelomanana-Vidal, V. Chiral 1,2-Bis(phosphetano)benzenes: Preparation and Use in the Ru-Catalyzed Hydrogenations of Carbonyl Derivatives. *Chem. Eur. J.* **1999,** *5* (4), 1160.

(5) Hoffmann, R. W.; Weidmann, U. threo/erythro-Assignment of 1,3-diol Derivatives Based on 13C NMR Spectra. *Chem. Ber.* **1985,** *118* (10), 3980.

(6) Kobayashi, Y.; Tan, C.-H.; Kishi, Y. Toward Creation of a Universal NMR Database for Stereochemical Assignment: The Case of 1,3,5-Trisubstituted Acyclic Systems. *Helv. Chim. Acta* **2000,** *83* (9), 2562.

(7) Higashibayashi, S.; Czechtizky, W.; Kobayashi, Y.; Kishi, Y. Universal NMR Databases for Contiguous Polyols. *J. Am. Chem. Soc.* **2003,** *125* (47), 14379.

(8) Bode, S. E.; Wolberg, M.; Müller, M. Stereoselective Synthesis of 1,3-Diols. *Synthesis* **2006,** *2006* (04), 557.

(9) Rychnovsky, S. D.; Skalitzky, D. J. Stereochemistry of Alternating Polyol Chains: 13C NMR Analysis of 1,3-Diol Acetonides. *Tetrahedron Lett.* **1990,** *31* (7), 945.

(10) Rychnovsky, S. D.; Rogers, B.; Yang, G. Analysis of Two Carbon-13 NMR correlations for Determining the Stereochemistry of 1,3-Diol Acetonides. *J. Org. Chem.* **1993,** *58* (13), 3511.

(11) Yoneda, A.; Fukuhara, T.; Hara, S. Selective monofluorination of diols using DFMBA. *Chem. Commun.* **2005**, 3589.

(12) Gryff-Keller, A.; Szczeciński, P. An Efficient DFT Method of Predicting the One-, Two- and Three-bond Indirect Spin–spin Coupling Constants Involving a Fuorine Nucleus in Fluoroalkanes. *RSC Adv.* **2016,** *6* (86), 82783.
