# Supplementary material for: Conformational Analysis of 1,3-Difluorinated Alkanes
Source: J Org Chem. 2024 May 31;89(12):8789–803. doi: 10.1021/acs.joc.4c00670 (PMC11197103; doi:10.1021/acs.joc.4c00670)
Supplement: Supplementary file 2 — jo4c00670_si_004.zip [file jo4c00670_si_004.zip › SI/raw_data/difluoropentane/anti-pentane-raw-water.pdf]

Conformer		Energy (Hart	Energy (kJ/n	Relative Ene	Population	Population %
(G-_G)	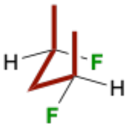 $G^{-}G$	nan	nan	nan	0	0
(G_G)	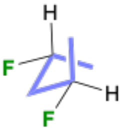 GG	-396.2065	-1040240.3	8.84	0.03	2.13
(A_G)	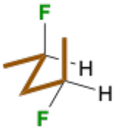 AG	-396.2076	-1040243	6.08	0.09	6.49
(A_A)	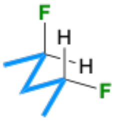 AA	-396.2099	-1040249.1	0	1	75.48
(G_A)	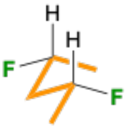 GA	-396.2076	-1040243	6.08	0.09	6.49
(G-_A)	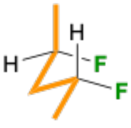 $G^{-}A$	-396.2073	-1040242.2	6.94	0.06	4.58
(G-_G-)	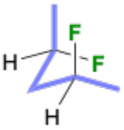 $G^{-}G^{-}$	-396.2045	-1040234.9	14.21	0	0.24
(G_G-)	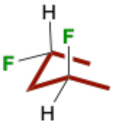 GG^{-}	nan	nan	nan	0	0
(A_G-)	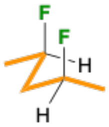 AG^{-}	-396.2073	-1040242.2	6.94	0.06	4.58
