# Supplementary material for: Conformational Analysis of 1,3-Difluorinated Alkanes
Source: J Org Chem. 2024 May 31;89(12):8789–803. doi: 10.1021/acs.joc.4c00670 (PMC11197103; doi:10.1021/acs.joc.4c00670)
Supplement: Supplementary file 2 — jo4c00670_si_004.zip [file jo4c00670_si_004.zip › SI/raw_data/difluoropentane/syn-pentane-raw-water.pdf]

Conformer		Energy (Hart	Energy (kJ/n	Relative Ene	Population	Population %
(G-_G)	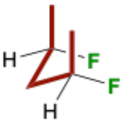 G^-G	-396.2021	-1040228.7	17.46	0	0.03
(G_G)	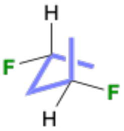 GG	-396.2062	-1040239.5	6.69	0.07	2.01
(A_G)	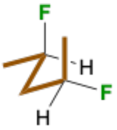 AG	-396.2074	-1040242.6	3.54	0.24	7.17
(A_A)	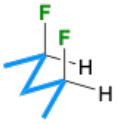 AA	-396.2085	-1040245.4	0.79	0.73	21.76
(G_A)	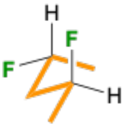 GA	-396.2088	-1040246.1	0	1	29.93
(G-_A)	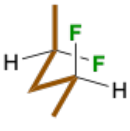 G^-A	-396.2074	-1040242.6	3.54	0.24	7.17
(G-_G-)	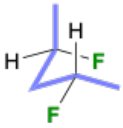 G^-G^-	-396.2062	-1040239.5	6.69	0.07	2.01
(G_G-)	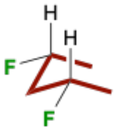 GG^-	nan	nan	nan	0	0
(A_G-)	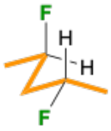 AG^-	-396.2088	-1040246.1	0	1	29.93
