# Supplementary material for: Conformational Analysis of 1,3-Difluorinated Alkanes
Source: J Org Chem. 2024 May 31;89(12):8789–803. doi: 10.1021/acs.joc.4c00670 (PMC11197103; doi:10.1021/acs.joc.4c00670)
Supplement: Supplementary file 2 — jo4c00670_si_004.zip [file jo4c00670_si_004.zip › SI/raw_data/difluoropentane/anti-pentane-raw-vacuum.pdf]

Conformer		Energy (Hart	Energy (kJ/n	Relative Ene	Population	Population %
(G-_G)	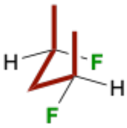 G^-G	nan	nan	nan	0	0
(G_G)	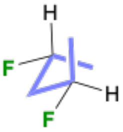 GG	-396.199	-1040220.4	11.76	0.01	0.6
(A_G)	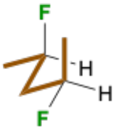 AG	-396.2018	-1040227.9	4.32	0.17	12.8
(A_A)	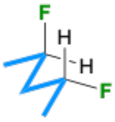 AA	-396.2035	-1040232.2	0	1	73.3
(G_A)	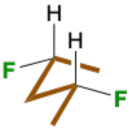 GA	-396.2018	-1040227.9	4.32	0.17	12.8
(G-_A)	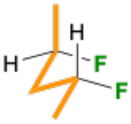 G^-A	-396.1968	-1040214.7	17.52	0	0.1
(G-_G-)	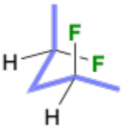 G^-G^-	-396.1983	-1040218.7	13.46	0	0.3
(G_G-)	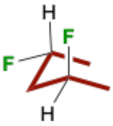 GG^-	nan	nan	nan	0	0
(A_G-)	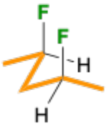 AG^-	-396.1968	-1040214.7	17.52	0	0.1
