# Supplementary material for: Conformational Analysis of 1,3-Difluorinated Alkanes
Source: J Org Chem. 2024 May 31;89(12):8789–803. doi: 10.1021/acs.joc.4c00670 (PMC11197103; doi:10.1021/acs.joc.4c00670)
Supplement: Supplementary file 2 — jo4c00670_si_004.zip [file jo4c00670_si_004.zip › SI/raw_data/difluoropentane/syn-pentane-raw-chloroform.pdf]

Conformer		Energy (Hart	Energy (kJ/n	Relative Ene	Population	Population %
(G-_G)	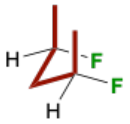 G^-G	-396.2055	-1040237.5	22.32	0	0
(G_G)	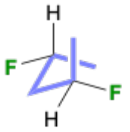 GG	-396.2118	-1040254.2	5.63	0.1	2.96
(A_G)	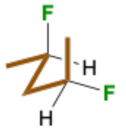 AG	-396.2134	-1040258.3	1.53	0.54	15.5
(A_A)	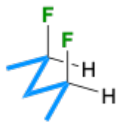 AA	-396.2124	-1040255.8	4.07	0.19	5.56
(G_A)	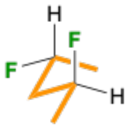 GA	-396.214	-1040259.8	0	1	28.75
(G-_A)	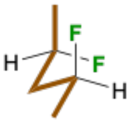 G^-A	-396.2134	-1040258.3	1.53	0.54	15.5
(G-_G-)	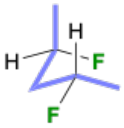 G^-G^-	-396.2118	-1040254.2	5.63	0.1	2.96
(G_G-)	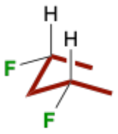 GG^-	nan	nan	nan	0	0
(A_G-)	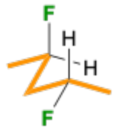 AG^-	-396.214	-1040259.8	0	1	28.75
