# Supplementary material for: Conformational Analysis of 1,3-Difluorinated Alkanes
Source: J Org Chem. 2024 May 31;89(12):8789–803. doi: 10.1021/acs.joc.4c00670 (PMC11197103; doi:10.1021/acs.joc.4c00670)
Supplement: Supplementary file 2 — jo4c00670_si_004.zip [file jo4c00670_si_004.zip › SI/raw_data/difluoropentane/syn-pentane-raw-vacuum.pdf]

Conformer		Energy (Hartrees)	Energy (kJ/mol)	Relative Energy (kJ/mol)	Population	Population %
(G-_G)	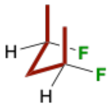 G^-G	-396.1916	-1040201.08	26.2	0	0
(G_G)	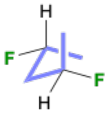 GG	-396.1991	-1040220.73	6.55	0.07	2.02
(A_G)	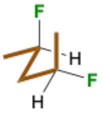 AG	-396.2012	-1040226.36	0.92	0.69	19.56
(A_A)	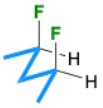 AA	-396.1965	-1040213.97	13.31	0	0.13
(G_A)	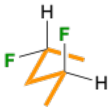 GA	-396.2016	-1040227.28	0	1	28.36
(G-_A)	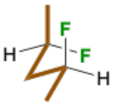 G^-A	-396.2012	-1040226.36	0.92	0.69	19.56
(G-_G-)	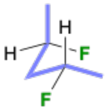 G^-G^-	-396.1991	-1040220.73	6.55	0.07	2.02
(G_G-)	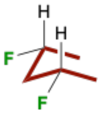 GG^-	nan	nan	nan	0	0
(A_G-)	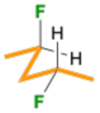 AG^-	-396.2016	-1040227.28	0	1	28.36
