# Supplementary material for: Conformational Analysis of 1,3-Difluorinated Alkanes
Source: J Org Chem. 2024 May 31;89(12):8789–803. doi: 10.1021/acs.joc.4c00670 (PMC11197103; doi:10.1021/acs.joc.4c00670)
Supplement: Supplementary file 2 — jo4c00670_si_004.zip [file jo4c00670_si_004.zip › SI/raw_data/difluoropentane/anti-pentane-raw-chloroform.pdf]

Conformer		Energy (Hart	Energy (kJ/n	Relative Ene	Population	Population %
(G-_G)	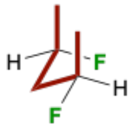 G^-G	nan	nan	nan	0	0
(G_G)	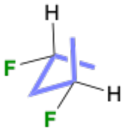 GG	-396.212	-1040254.7	10.39	0.02	1.28
(A_G)	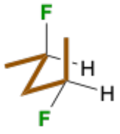 AG	-396.2135	-1040258.6	6.44	0.07	6.31
(A_A)	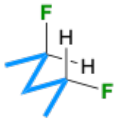 AA	-396.216	-1040265.1	0	1	84.85
(G_A)	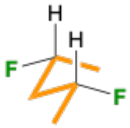 GA	-396.2135	-1040258.6	6.44	0.07	6.31
(G-_A)	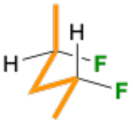 G^-A	-396.2112	-1040252.5	12.51	0.01	0.54
(G-_G-)	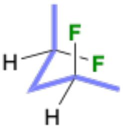 G^-G^-	-396.2101	-1040249.7	15.37	0	0.17
(G_G-)	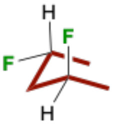 GG^-	nan	nan	nan	0	0
(A_G-)	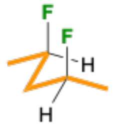 AG^-	-396.2112	-1040252.5	12.51	0.01	0.54

ό
