# Supplementary material for: Conformational Analysis of 1,3-Difluorinated Alkanes
Source: J Org Chem. 2024 May 31;89(12):8789–803. doi: 10.1021/acs.joc.4c00670 (PMC11197103; doi:10.1021/acs.joc.4c00670)
Supplement: Supplementary file 2 — jo4c00670_si_004.zip [file jo4c00670_si_004.zip › SI/raw_data/difluoroheptane/anti-heptane-raw-vacuum.pdf]

Conformer		Energy (Hart	Energy (kJ/n	Relative Ene	Population	Population %
(A_A_A_A)	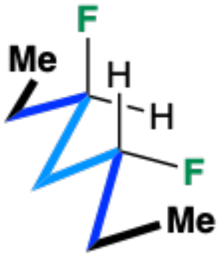 *AAAA*	-474.7849	-1246547.8	0	1	31.24
(A_A_A_G-)	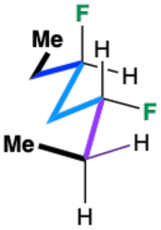 *AAAG⁻* (identical as *G⁻AAA*)	-474.7839	-1246545	2.81	0.32	10.05
(A_A_A_G)	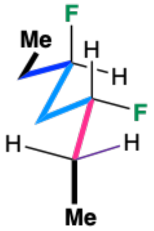 *AAAG* (identical as *GAAA*)	-474.7837	-1246544.7	3.15	0.28	8.76
(A_A_G_A)	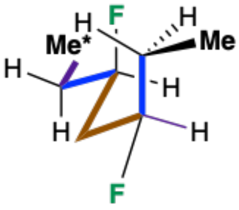 *AAGA* (identical as *AGAA*)	-474.7834	-1246543.9	3.96	0.2	6.32
(A_A_G_G-)	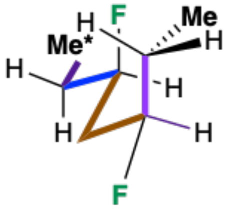 *AAGG⁻* (identical as *G⁻GAA*)	nan	nan	nan	0	0

(A_A_G_G)	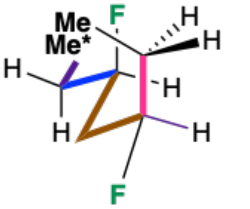<br>AAGG<br>(identical as<br>GGAA)	-474.781	-1246537.6	10.19	0.02	0.51
(A_A_G-_A)	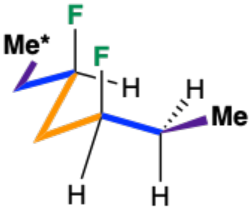<br>AAG-A<br>(identical as<br>AG-AA)	-474.7785	-1246531.1	16.75	0	0.04
(A_A_G-_G-)	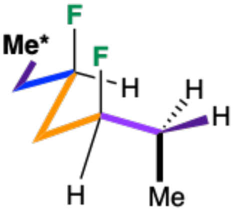<br>AAG-G-<br>(identical as<br>G-G-AA)	-474.7781	-1246529.8	18	0	0.02
(A_A_G-_G)	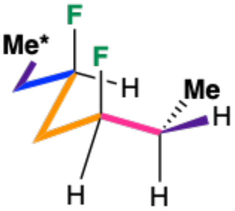<br>AAG-G<br>(identical as<br>GG-AA)	-474.7744	-1246520.1	27.7	0	0
(A_G_A_A)	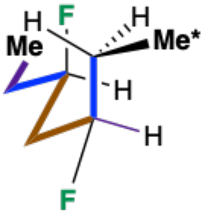<br>AGAA<br>(identical as<br>AAGA)	-474.7834	-1246543.9	3.96	0.2	6.32

(A_G_A_G-)	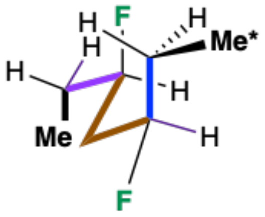<br>AGAG⁻<br>(identical as<br>G⁻AGA)	-474.7818	-1246539.5	8.32	0.03	1.09
(A_G_A_G)	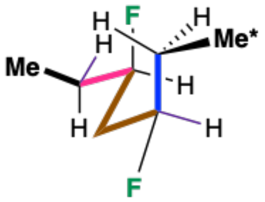<br>AGAG<br>(identical as<br>GAGA)	-474.7818	-1246539.6	8.18	0.04	1.15
(A_G_G_A)	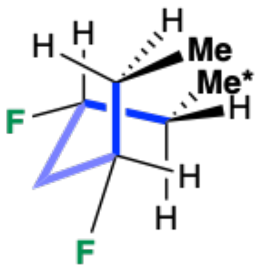<br>AGGA	-474.7802	-1246535.4	12.42	0.01	0.21
(A_G_G_G-)	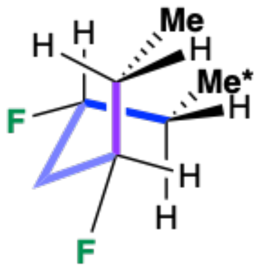<br>AGGG⁻	nan	nan	nan	0	0
(A_G_G_G)	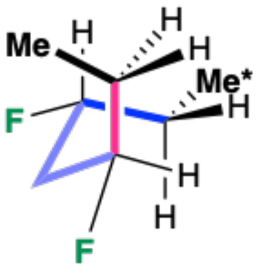<br>AGGG	-474.7797	-1246534.1	13.74	0	0.12

(A_G_G-_A)	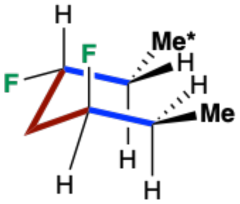 $AGG^{-}A$ (identical as $AG^{-}GA$)	nan	nan	nan	0	0
(A_G_G-_G-)	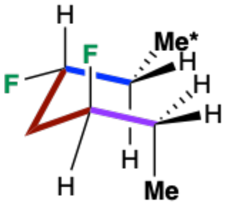 $AGG^{-}G^{-}$ (identical as $G^{-}G^{-}GA$)	nan	nan	nan	0	0
(A_G_G-_G)	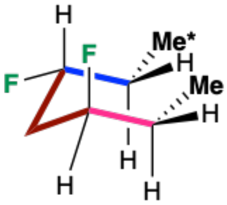 $AGG^{-}G$ (identical as $GG^{-}GA$)	nan	nan	nan	0	0
(A_G-_A_A)	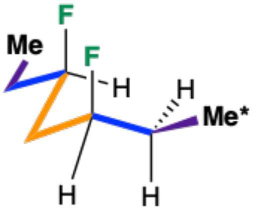 $AG^{-}AA$ (identical as $AAG^{-}A$)	-474.7785	-1246531.1	16.75	0	0.04
(A_G-_A_G-)	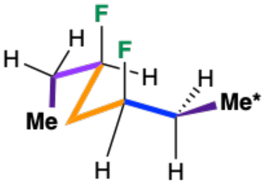 $AG^{-}AG^{-}$ (identical as $G^{-}AG^{-}A$)	-474.7772	-1246527.5	20.37	0	0.01

(A_G-_A_G)	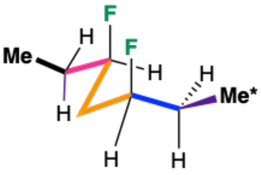 AG^{-}AG (identical as GAG^{-}A)	-474.7769	-1246526.7	21.11	0	0.01
(A_G-_G_A)	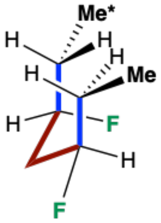 AG^{-}GA (identical as AGG^{-}A)	nan	nan	nan	0	0
(A_G-_G_G-)	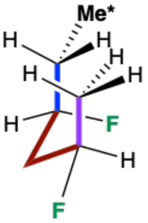 AG^{-}GG^{-} (identical as G^{-}GG^{-}A)	nan	nan	nan	0	0
(A_G-_G_G)	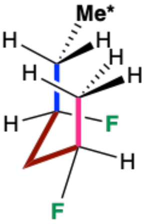 AG^{-}GG (identical as GGG^{-}A)	-474.7769	-1246526.7	21.14	0	0.01
(A_G-_G-_A)	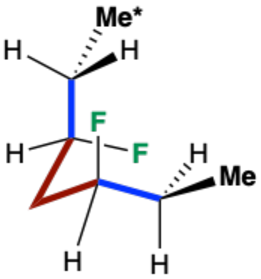 AG^{-}G^{-}A	-474.7799	-1246534.5	13.32	0	0.14

(A_G-_G-_G-)	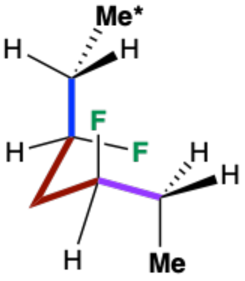<br>$AG^{-}G^{-}G^{-}$	-474.7793	-1246533.2	14.65	0	0.08
(A_G-_G-_G)	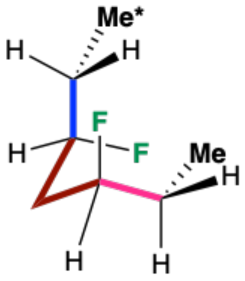<br>$AG^{-}G^{-}G$	-474.774	-1246519.2	28.65	0	0
(G_A_A_A)	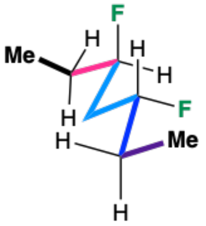<br>GAAA<br>(identical<br>as AAAG)	-474.7837	-1246544.7	3.15	0.28	8.76
(G_A_A_G-)	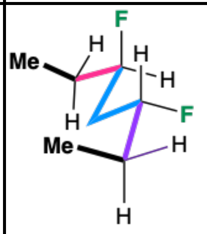<br>$GAAG^{-}$<br>(identical<br>as $G^{-}AAG$)	-474.7826	-1246541.8	6.02	0.09	2.75
(G_A_A_G)	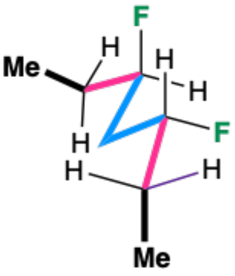<br>GAAG	-474.7824	-1246541.1	6.71	0.07	2.08

(G_A_G_A)	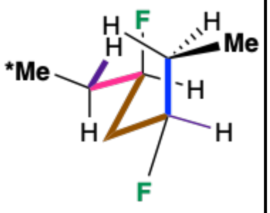 GAGA (identical as AGAG)	-474.7818	-1246539.6	8.18	0.04	1.15
(G_A_G_G-)	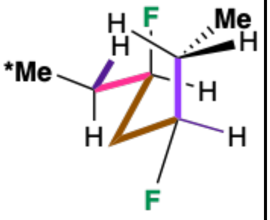 GAGG- (identical as G-GAG)	nan	nan	nan	0	0
(G_A_G_G)	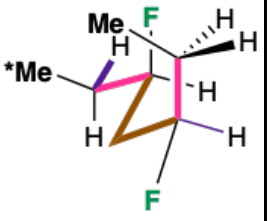 GAGG (identical as GGAG)	-474.7802	-1246535.4	12.45	0.01	0.21
(G_A_G-_A)	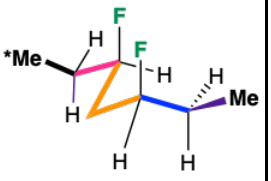 GAG-A (identical as AG-AG)	-474.7769	-1246526.7	21.11	0	0.01
(G_A_G-_G-)	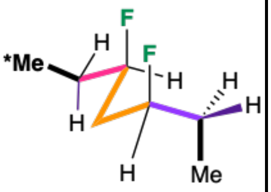 GAG-G- (identical as G-G-AG)	-474.7768	-1246526.4	21.45	0	0.01

(G_A_G-_G)	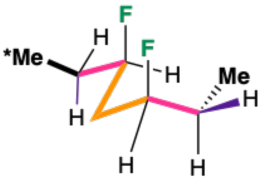 GAG^{-}G (identical as GG^{-}AG)	nan	nan	nan	0	0
(G_G_A_A)	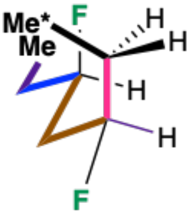 GGAA (identical as AAGG)	-474.781	-1246537.6	10.19	0.02	0.51
(G_G_A_G-)	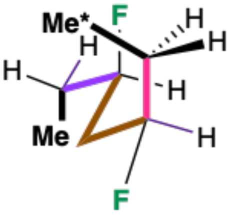 GGAG^{-} (identical as G^{-}AGG)	-474.7805	-1246536.1	11.7	0.01	0.28
(G_G_A_G)	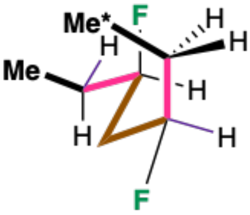 GGAG (identical as GAGG)	-474.7802	-1246535.4	12.45	0.01	0.21
(G_G_G_A)	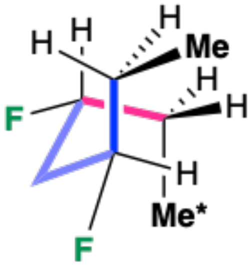 GGGA	-474.7797	-1246534.1	13.74	0	0.12

(G_G_G_G-)	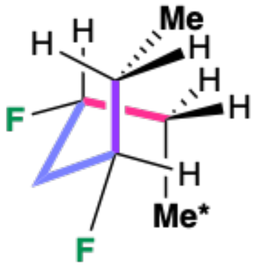 $GGGG^{-}$	nan	nan	nan	0	0
(G_G_G_G)	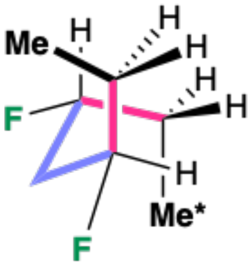 $GGGG$	-474.7779	-1246529.3	18.49	0	0.02
(G_G_G-_A)	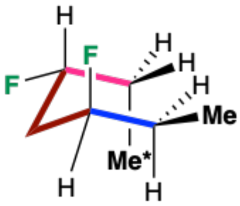 $GGG^{-}A$ (identical as $AG^{-}G^{-}G$)	-474.7769	-1246526.7	21.14	0	0.01
(G_G_G-_G-)	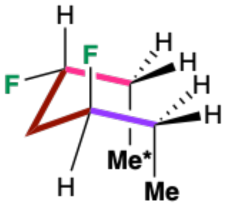 $GGG^{-}G^{-}$ (identical as $G^{-}G^{-}GG$)	-474.7758	-1246523.9	23.88	0	0
(G_G_G-_G)	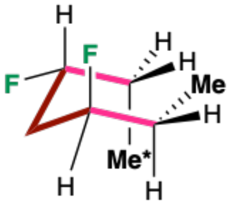 $GGG^{-}G$ (identical as $GG^{-}GG$)	nan	nan	nan	0	0

(G_G-_A_A)	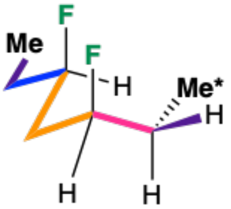 $GG^{-}AA$ (identical as $AAG^{-}G$)	-474.7744	-1246520.1	27.7	0	0
(G_G-_A_G-)	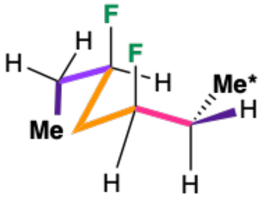 $GG^{-}AG^{-}$ (identical as $G^{-}AG^{-}G$)	-474.7733	-1246517.3	30.51	0	0
(G_G-_A_G)	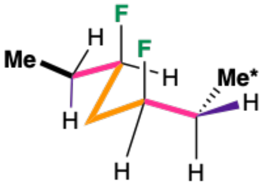 $GG^{-}AG$ (identical as $GAG^{-}G$)	nan	nan	nan	0	0
(G_G-_G_A)	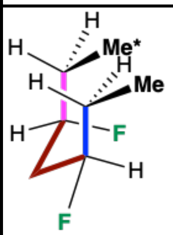 $GG^{-}GA$ (identical as $AGG^{-}G$)	nan	nan	nan	0	0
(G_G-_G_G-)	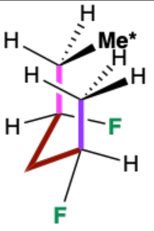 $GG^{-}GG^{-}$ (identical as $G^{-}GG^{-}G$)	nan	nan	nan	0	0

(G_G-_G_G)	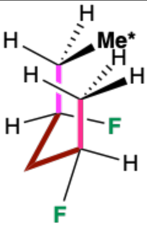 $GG^{-}GG$ (identical as $GGG^{-}G$)	nan	nan	nan	0	0
(G_G-_G-_A)	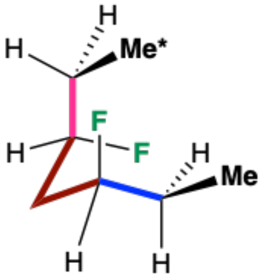 $GG^{-}G^{-}A$	-474.774	-1246519.2	28.65	0	0
(G_G-_G-_G-)	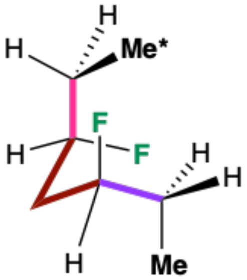 $GG^{-}G^{-}G^{-}$	-474.7739	-1246518.8	29.06	0	0
(G_G-_G-_G)	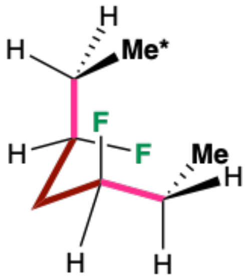 $GG^{-}G^{-}G$	-474.7661	-1246498.4	49.38	0	0
(G-_A_A_A)	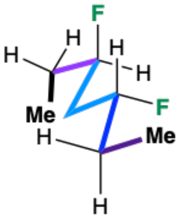 $G^{-}AAA$ (identical as $AAAG^{-}$)	-474.7839	-1246545	2.81	0.32	10.05

(G-_A_A_G-)	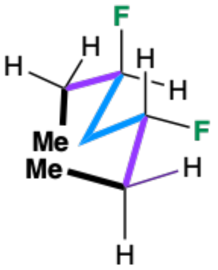 $G^{-}AAG^{-}$	-474.7829	-1246542.4	5.44	0.11	3.48
(G-_A_A_G)	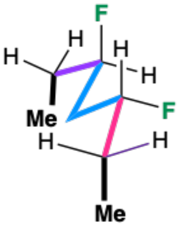 $G^{-}AAG$ (identical as $GAAG^{-}$)	-474.7826	-1246541.8	6.02	0.09	2.75
(G-_A_G_A)	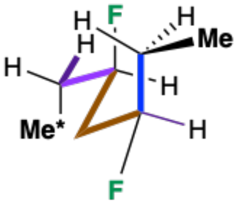 $G^{-}AGA$ (identical as $AGAG^{-}$)	-474.7818	-1246539.5	8.32	0.03	1.09
(G-_A_G_G-)	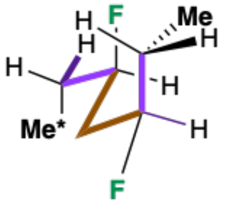 $G^{-}AGG^{-}$ (identical as $G^{-}GAG^{-}$)	nan	nan	nan	0	0
(G-_A_G_G)	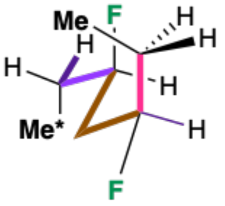 $G^{-}AGG$ (identical as $GGAG^{-}$)	-474.7805	-1246536.1	11.7	0.01	0.28

(G-_A_G-_A)	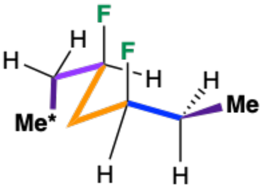<br>G-AG-A<br>(identical as<br>AG-AG-)	-474.7772	-1246527.5	20.37	0	0.01
(G-_A_G-_G-)	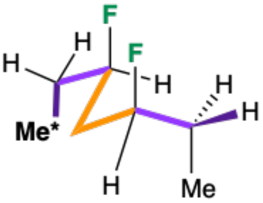<br>G-AG-G-<br>(identical as<br>G-G-AG-)	-474.7769	-1246526.8	21.07	0	0.01
(G-_A_G-_G)	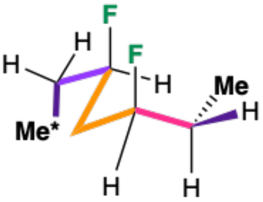<br>G-AG-G<br>(identical as<br>GG-AG-)	-474.7733	-1246517.3	30.51	0	0
(G-_G_A_A)	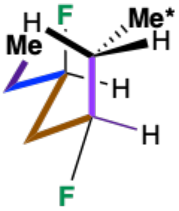<br>G-GAA<br>(identical as<br>AAGG-)	nan	nan	nan	0	0
(G-_G_A_G-)	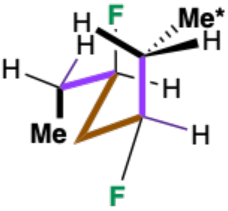<br>G-GAG-<br>(identical as<br>G-AGG-)	nan	nan	nan	0	0

(G-_G_A_G)	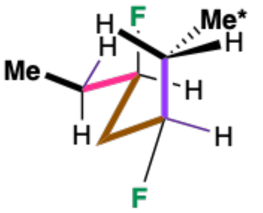 $G^{-}GAG$ (identical as $GAGG^{-}$)	nan	nan	nan	0	0
(G-_G_G_A)	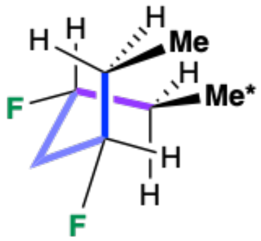 $G^{-}GGA$	nan	nan	nan	0	0
(G-_G_G_G-)	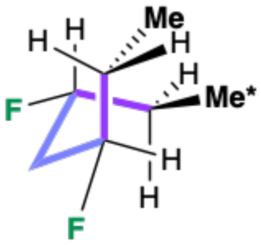 $G^{-}GGG^{-}$	nan	nan	nan	0	0
(G-_G_G_G)	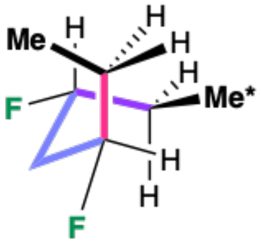 $G^{-}GGG$	nan	nan	nan	0	0
(G-_G_G-_A)	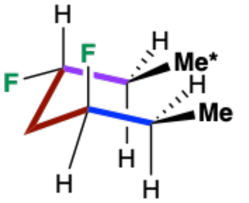 $G^{-}GG^{-}A$ (identical as $AG^{-}GG^{-}$)	nan	nan	nan	0	0

(G-_G_G-_G-)	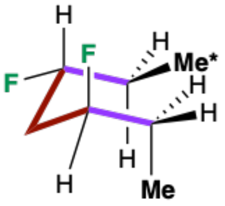 $G^-GG^-G^-$ (identical as $G^-G^-GG^-$)	nan	nan	nan	0	0
(G-_G_G-_G)	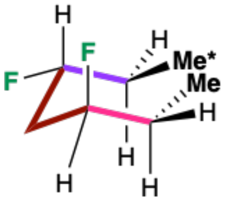 G^-GG^-G (identical as GG^-GG^-)	nan	nan	nan	0	0
(G-_G-_A_A)	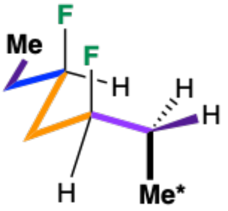 G^-G^-AA (identical as AAG^-G^-)	-474.7781	-1246529.8	18	0	0.02
(G-_G-_A_G-)	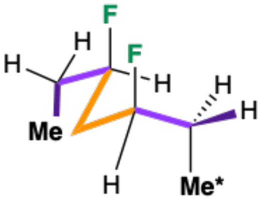 $G^-G^-AG^-$ (identical as $G^-AG^-G^-$)	-474.7769	-1246526.8	21.07	0	0.01
(G-_G-_A_G)	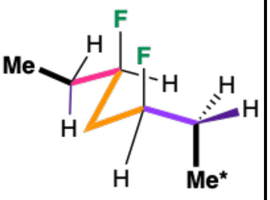 G^-G^-AG (identical as GAG^-G^-)	-474.7768	-1246526.4	21.45	0	0.01

(G-_G-_G_A)	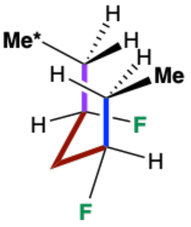<br>$G^{-}G^{-}GA$<br>(identical as $AGG^{-}G^{-}$)	nan	nan	nan	0	0
(G-_G-_G_G-)	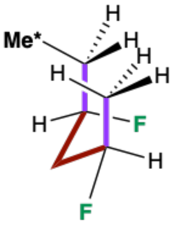<br>$G^{-}G^{-}GG^{-}$<br>(identical as $G^{-}GG^{-}G^{-}$)	nan	nan	nan	0	0
(G-_G-_G_G)	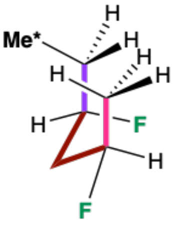<br>$G^{-}G^{-}GG$<br>(identical as $GGG^{-}G^{-}$)	-474.7758	-1246523.9	23.88	0	0
(G-_G-_G-_A)	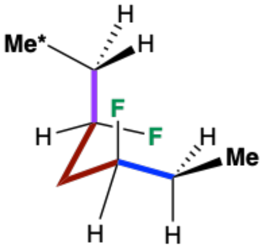<br>$G^{-}G^{-}G^{-}A$	-474.7793	-1246533.2	14.65	0	0.08
(G-_G-_G-_G-)	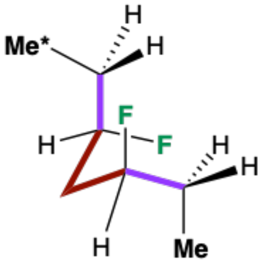<br>$G^{-}G^{-}G^{-}G^{-}$	-474.7778	-1246529.1	18.72	0	0.02

(G-_G-_G-_G)	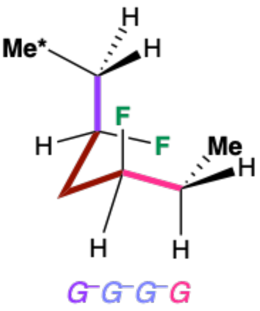	-474.7739	-1246518.8	29.06	0	0
