# Supplementary material for: Conformational Analysis of 1,3-Difluorinated Alkanes
Source: J Org Chem. 2024 May 31;89(12):8789–803. doi: 10.1021/acs.joc.4c00670 (PMC11197103; doi:10.1021/acs.joc.4c00670)
Supplement: Supplementary file 2 — jo4c00670_si_004.zip [file jo4c00670_si_004.zip › SI/raw_data/difluoroheptane/syn-heptane-raw-chloroform.pdf]

Conformer		Energy (Hart	Energy (kJ/n	Relative Ene	Population	Population %
(G-_G-_G-_C	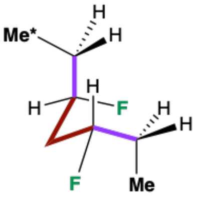 $G^-G^-G^-G^-$ (enantiomeric with GGGG)	-474.7932	-1246569.4	11.6	0.01	0.13
(G-_G-_G-_C	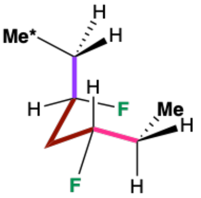 $G^-G^-G^-G$ (enantiomeric with G^-GGG)	nan	nan	nan	0	0
(G-_G-_G-_A	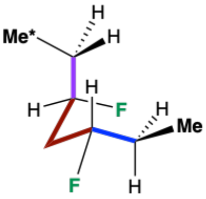 $G^-G^-G^-A$ (enantiomeric with AGGG)	-474.7942	-1246572.1	8.91	0.03	0.38
(G-_G-_G_G	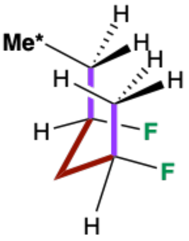 $G^-G^-GG^-$	nan	nan	nan	0	0
(G-_G-_G_G	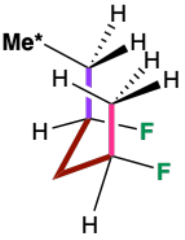 G^-G^-GG	-474.7862	-1246551.2	29.79	0	0
(G-_G-_G_A	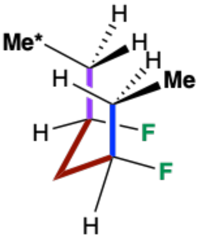 G^-G^-GA	-474.7873	-1246554.1	26.97	0	0

(G-_G-_A_G	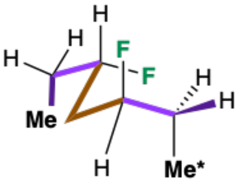 $G^-G^-AG^-$ (enantiomeric with GAGG)	-474.7952	-1246574.7	6.35	0.08	1.07
(G-_G-_A_G	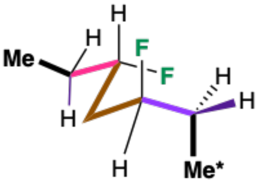 G^-G^-AG (enantiomeric with G^-AGG)	-474.7957	-1246576.1	4.93	0.14	1.89
(G-_G-_A_A)	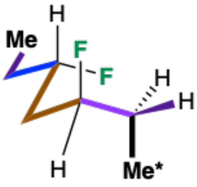 G^-G^-AA (enantiomeric with AAGG)	-474.7955	-1246575.7	5.33	0.12	1.61
(G-_G_G-_G	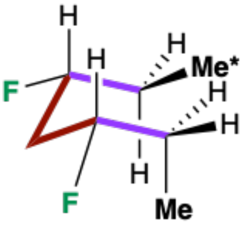 $G^-GG^-G^-$	nan	nan	nan	0	0
(G-_G_G-_G	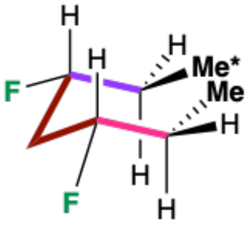 G^-GG^-G	nan	nan	nan	0	0
(G-_G_G-_A	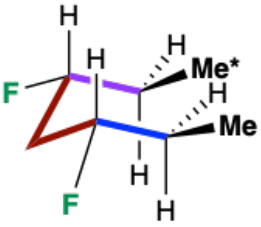 G^-GG^-A	nan	nan	nan	0	0

(G-_G_G_G-	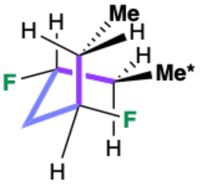 G^-GGG^- (enantiomeric with GG^-G^-G)	nan	nan	nan	0	0
(G-_G_G_G)	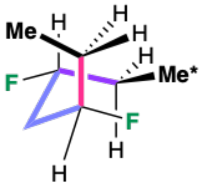 G^-GGG (enantiomeric with $G^-G^-G^-G$)	nan	nan	nan	0	0
(G-_G_G_A)	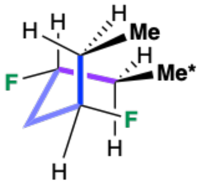 G^-GGA (enantiomeric with AG^-G^-G)	nan	nan	nan	0	0
(G-_G_A_G-	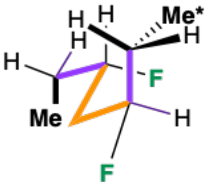 G^-GAG^- (enantiomeric with GAG^-G)	nan	nan	nan	0	0
(G-_G_A_G)	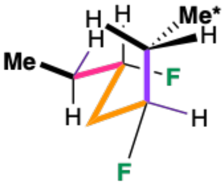 G^-GAG (enantiomeric with G^-AG^-G)	nan	nan	nan	0	0
(G-_G_A_A)	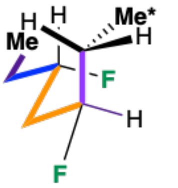 G^-GAA (enantiomeric with AAG^-G)	nan	nan	nan	0	0

(G-_A_G-_G	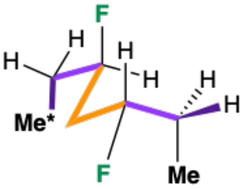 $G^{-}AG^{-}G^{-}$ (enantiomeric with GGAG)	-474.7955	-1246575.5	5.53	0.11	1.48
(G-_A_G-_G	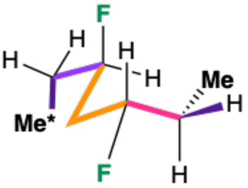 $G^{-}AG^{-}G$ (enantiomeric with $G^{-}GAG$)	nan	nan	nan	0	0
(G-_A_G-_A)	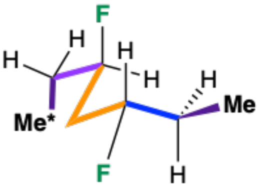 $G^{-}AG^{-}A$ (enantiomeric with AGAG)	-474.7962	-1246577.4	3.61	0.23	3.22
(G-_A_G_G-	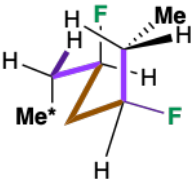 $G^{-}AGG^{-}$ (enantiomeric with $GG^{-}AG$)	-474.7917	-1246565.7	15.35	0	0.03
(G-_A_G_G)	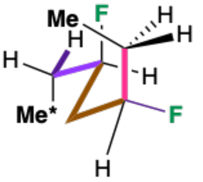 $G^{-}AGG$ (enantiomeric with $G^{-}G^{-}AG$)	-474.7957	-1246576.1	4.93	0.14	1.89
(G-_A_G_A)	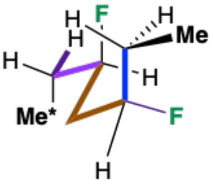 $G^{-}AGA$ (enantiomeric with $AG^{-}AG$)	-474.7959	-1246576.5	4.51	0.16	2.24

(G-_A_A_G-)	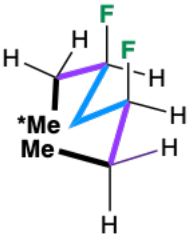 G^-AAG^-	-474.793	-1246569	12.04	0.01	0.11
(G-_A_A_G)	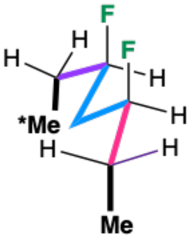 G^-AAG	-474.7936	-1246570.7	10.37	0.02	0.21
(G-_A_A_A)	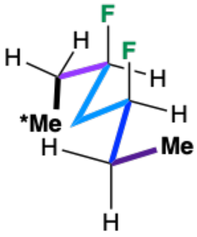 G^-AAA	-474.7943	-1246572.4	8.62	0.03	0.43
(G_G-_G-_G	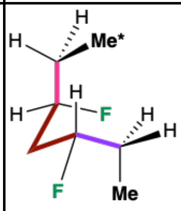 $GG^-G^-G^-$ (enantiomeric with $GGGG^-$)	-474.7884	-1246557	24.05	0	0
(G_G-_G-_G	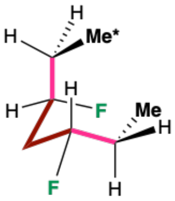 GG^-G^-G (enantiomeric with G^-GGG^-)	nan	nan	nan	0	0
(G_G-_G-_A	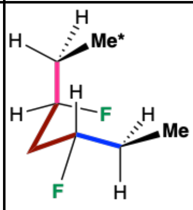 GG^-G^-A (enantiomeric with $AGGG^-$)	-474.7897	-1246560.5	20.56	0	0

(G_G-_G_G-	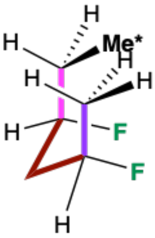 GG$^-$GG$^-$	nan	nan	nan	0	0
(G_G-_G_G)	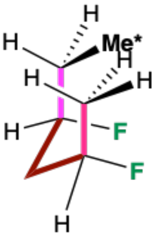 GG$^-$GG	nan	nan	nan	0	0
(G_G-_G_A)	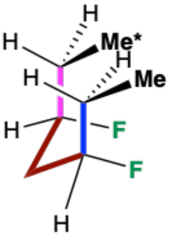 GG$^-$GA	nan	nan	nan	0	0
(G_G-_A_G-	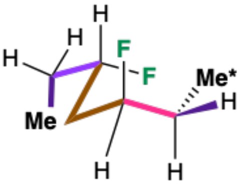 GG$^-$AG$^-$ (enantiomeric with GAGG$^-$)	-474.7904	-1246562.2	18.86	0	0.01
(G_G-_A_G)	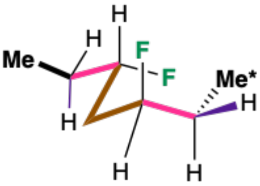 GG$^-$AG (enantiomeric with G$^-$AGG$^-$)	-474.7917	-1246565.7	15.35	0	0.03
(G_G-_A_A)	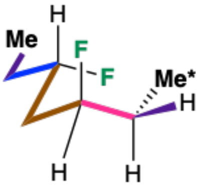 GG$^-$AA (enantiomeric with AAGG$^-$)	-474.7904	-1246562.1	18.93	0	0.01

(G_G_G-_G-	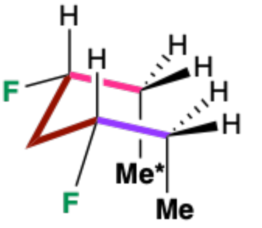 GGG$^-$G$^-$	nan	nan	nan	0	0
(G_G_G-_G)	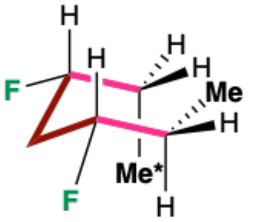 GGG$^-$G	nan	nan	nan	0	0
(G_G_G-_A)	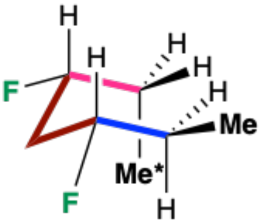 GGG$^-$A	nan	nan	nan	0	0
(G_G_G_G-)	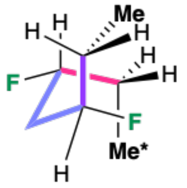 GGGG$^-$ (enantiomeric with GG$^-$G$^-$G$^-$)	-474.7884	-1246557	24.05	0	0
(G_G_G_G)	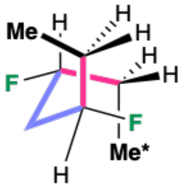 GGGG (enantiomeric with G$^-$G$^-$G$^-$G$^-$)	-474.7932	-1246569.4	11.6	0.01	0.13
(G_G_G_A)	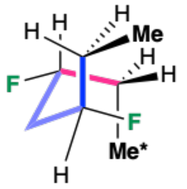 GGGA (enantiomeric with AG$^-$G$^-$G$^-$)	-474.793	-1246569.1	11.98	0.01	0.11

(G_G_A_G-)	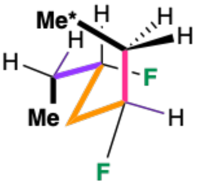 GGAG- (enantiomeric with GAG-G-)	-474.795	-1246574.4	6.66	0.07	0.94
(G_G_A_G)	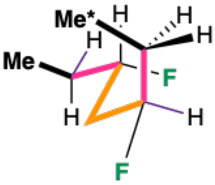 GGAG (enantiomeric with G-AG-G-)	-474.7955	-1246575.5	5.53	0.11	1.48
(G_G_A_A)	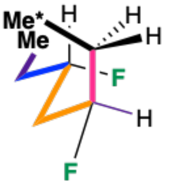 GGAA (enantiomeric with AAG-G-)	-474.7962	-1246577.4	3.64	0.23	3.18
(G_A_G-_G-	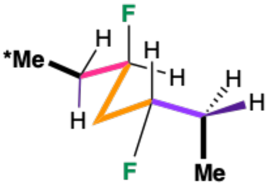 GAG-G- (enantiomeric with GGAG-)	-474.795	-1246574.4	6.66	0.07	0.94
(G_A_G-_G)	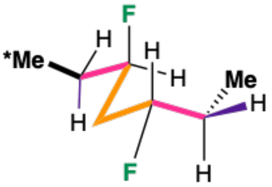 GAG-G (enantiomeric with G-GAG-)	nan	nan	nan	0	0
(G_A_G-_A)	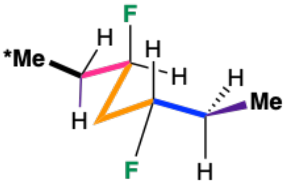 GAG-A (enantiomeric with AGAG-)	-474.7962	-1246577.5	3.49	0.24	3.38

(G_A_G_G-)	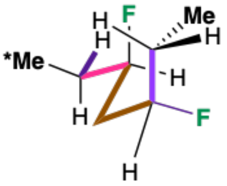 GAGG$^-$ (enantiomeric with GG$^-$AG$^-$)	-474.7904	-1246562.2	18.86	0	0.01
(G_A_G_G)	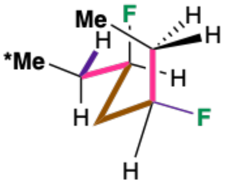 GAGG (enantiomeric with G$^-$G$^-$AG$^-$)	-474.7952	-1246574.7	6.35	0.08	1.07
(G_A_G_A)	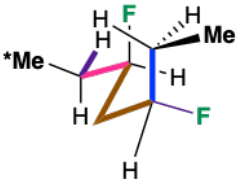 GAGA (enantiomeric with AG$^-$AG$^-$)	-474.7961	-1246577.2	3.81	0.21	2.97
(G_A_A_G-)	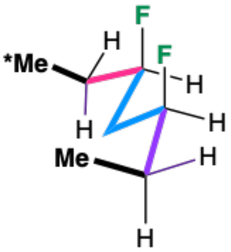 GAAG$^-$	-474.7935	-1246570.2	10.81	0.01	0.18
(G_A_A_G)	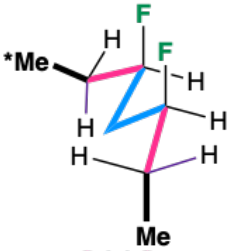 GAAG	-474.793	-1246569	12.04	0.01	0.11
(G_A_A_A)	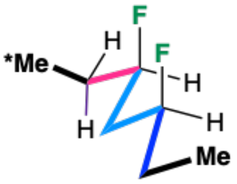 GAAA	-474.7952	-1246574.8	6.24	0.08	1.11

(A_G-_G-_G	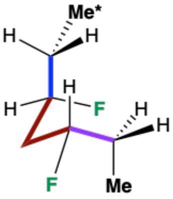 AG$^-$G$^-$G$^-$ (enantiomeric with GGGA)	-474.793	-1246569.1	11.98	0.01	0.11
(A_G-_G-_G	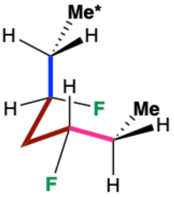 AG$^-$G$^-$G (enantiomeric with G$^-$GGA)	nan	nan	nan	0	0
(A_G-_G-_A)	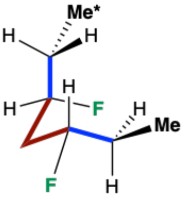 AG$^-$G$^-$A (enantiomeric with AGGA)	-474.7951	-1246574.5	6.5	0.07	1
(A_G-_G_G-	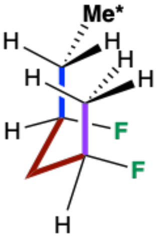 AG$^-$GG$^-$	nan	nan	nan	0	0
(A_G-_G_G)	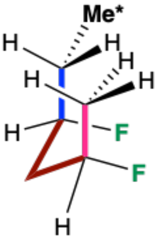 AG$^-$GG	-474.7873	-1246554.1	26.97	0	0
(A_G-_G_A)	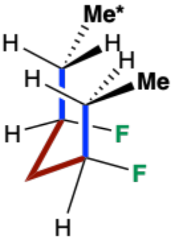 AG$^-$GA	-474.7885	-1246557.2	23.8	0	0

(A_G-_A_G-)	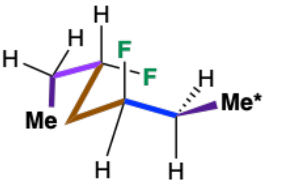 AG^-AG^- (enantiomeric with GAGA)	-474.7961	-1246577.2	3.81	0.21	2.97
(A_G-_A_G)	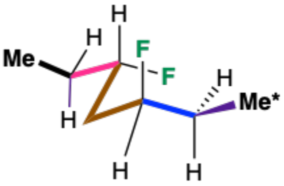 AG^-AG (enantiomeric with G^-AGA)	-474.7959	-1246576.5	4.51	0.16	2.24
(A_G-_A_A)	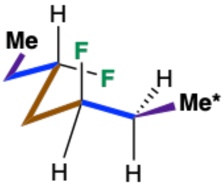 AG^-AA (enantiomeric with AAGA)	-474.7973	-1246580.2	0.79	0.73	10.05
(A_G_G-_G-	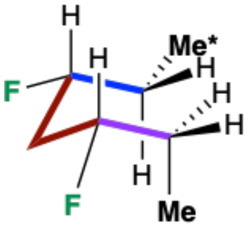 AGG^-G^-	nan	nan	nan	0	0
(A_G_G-_G)	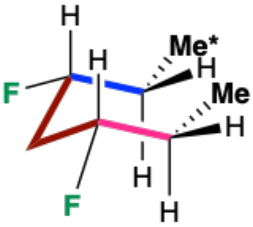 AGG^-G	nan	nan	nan	0	0
(A_G_G-_A)	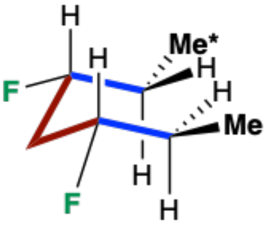 AGG^-A	nan	nan	nan	0	0

(A_G_G_G-)	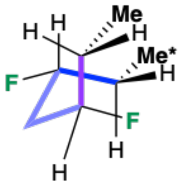 $AGGG^{-}$ (enantiomeric with $GG^{-}G^{-}A$)	-474.7897	-1246560.5	20.56	0	0
(A_G_G_G)	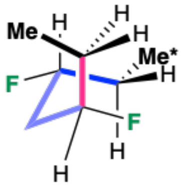 AGGG (enantiomeric with $G^{-}G^{-}G^{-}A$)	-474.7942	-1246572.1	8.91	0.03	0.38
(A_G_G_A)	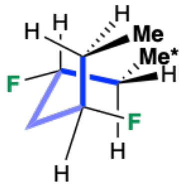 AGGA (enantiomeric with $AG^{-}G^{-}A$)	-474.7951	-1246574.5	6.5	0.07	1
(A_G_A_G-)	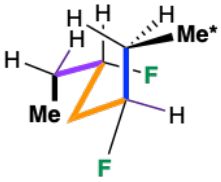 $AGAG^{-}$ (enantiomeric with $GAG^{-}A$)	-474.7962	-1246577.5	3.49	0.24	3.38
(A_G_A_G)	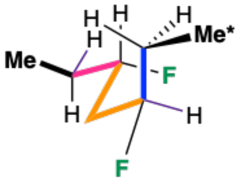 AGAG (enantiomeric with $G^{-}AG^{-}A$)	-474.7962	-1246577.4	3.61	0.23	3.22
(A_G_A_A)	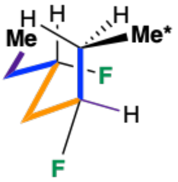 AGAA (enantiomeric with $AAG^{-}A$)	-474.7976	-1246581	0	1	13.83

(A_A_G-_G-)	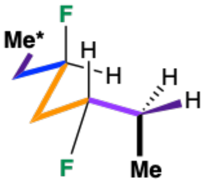 AAG-G- (enantiomeric with GGAA)	-474.7962	-1246577.4	3.64	0.23	3.18
(A_A_G-_G)	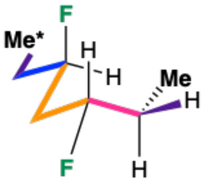 AAG-G (enantiomeric with G-GAA)	nan	nan	nan	0	0
(A_A_G-_A)	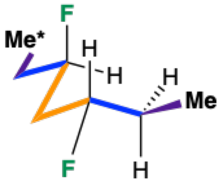 AAG-A (enantiomeric with AGAA)	-474.7976	-1246581	0	1	13.83
(A_A_G_G-)	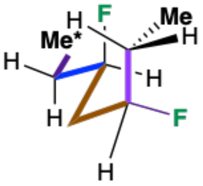 AAGG- (enantiomeric with GG-AA)	-474.7904	-1246562.1	18.93	0	0.01
(A_A_G_G)	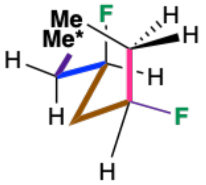 AAGG (enantiomeric with G-G-AA)	-474.7955	-1246575.7	5.33	0.12	1.61
(A_A_G_A)	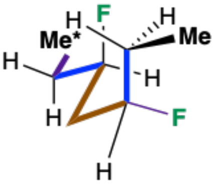 AAGA (enantiomeric with AG-AA)	-474.7973	-1246580.2	0.79	0.73	10.05

(A_A_A_G-)	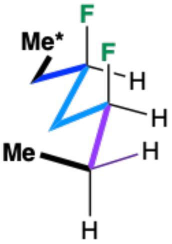 AAAG⁻	-474.7952	-1246574.8	6.24	0.08	1.11
(A_A_A_G)	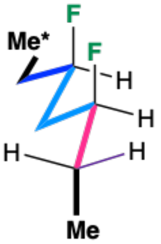 AAAG	-474.7943	-1246572.4	8.62	0.03	0.43
(A_A_A_A)	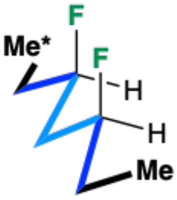 AAAA	-474.7953	-1246575.1	5.98	0.09	1.24
