# Supplementary material for: Conformational Analysis of 1,3-Difluorinated Alkanes
Source: J Org Chem. 2024 May 31;89(12):8789–803. doi: 10.1021/acs.joc.4c00670 (PMC11197103; doi:10.1021/acs.joc.4c00670)
Supplement: Supplementary file 2 — jo4c00670_si_004.zip [file jo4c00670_si_004.zip › SI/raw_data/difluoroheptane/anti-heptane-raw-water.pdf]

| Conformer | | Energy (Hart | Energy (kJ/n | Relative Ene | Population | Population % |
|---|---|---|---|---|---|---|
| (A_A_A_A) | 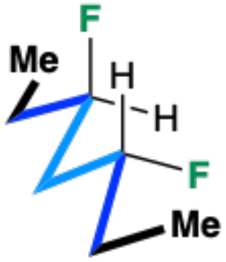 AAAA | -474.7909 | -1246563.4 | 0 | 1 | 32.02 |
| (A_A_A_G-) | 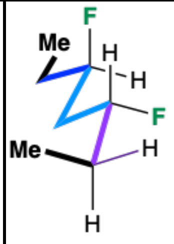 AAAG- (identical as G-AAA) | -474.7897 | -1246560.3 | 3.08 | 0.29 | 9.24 |
| (A_A_A_G) | 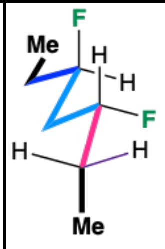 AAAG (identical as GAAA) | -474.79 | -1246561 | 2.36 | 0.39 | 12.35 |
| (A_A_G_A) | 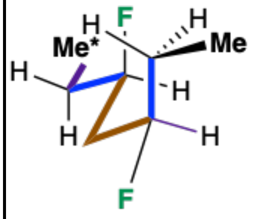 AAGA (identical as AGAA) | -474.7882 | -1246556.4 | 6.97 | 0.06 | 1.92 |
| (A_A_G_G-) | 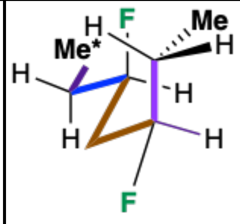 AAGG- (identical as G-GAA) | nan | nan | nan | 0 | 0 |

| | | | | | | |
|---|---|---|---|---|---|---|
| (A_A_G_G) | 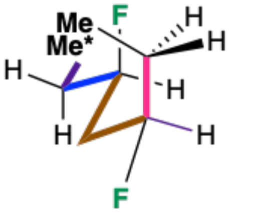 AAGG (identical as GGAA) | -474.7867 | -1246552.6 | 10.83 | 0.01 | 0.4 |
| (A_A_G-_A) | 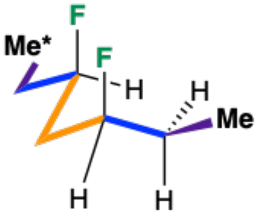 AAG$^-$A (identical as AG$^-$AA) | -474.7869 | -1246552.9 | 10.44 | 0.01 | 0.47 |
| (A_A_G-_G-) | 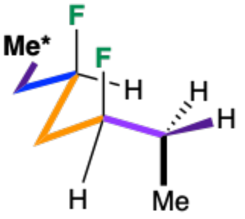 AAG$^-$G$^-$ (identical as G$^-$G$^-$AA) | -474.7873 | -1246554.1 | 9.27 | 0.02 | 0.76 |
| (A_A_G-_G) | 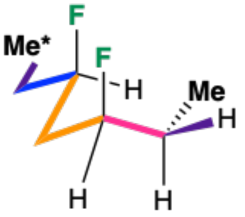 AAG$^-$G (identical as GG$^-$AA) | -474.7834 | -1246543.8 | 19.58 | 0 | 0.01 |
| (A_G_A_A) | 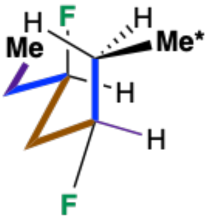 AGAA (identical as AAGA) | -474.7882 | -1246556.4 | 6.97 | 0.06 | 1.92 |

| | | | | | | |
|---|---|---|---|---|---|---|
| (A_G_A_G-) | 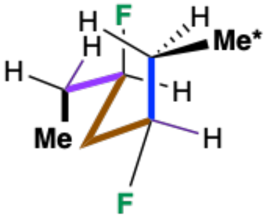 AGAG$^{-}$ (identical as G$^{-}$AGA) | -474.7872 | -1246553.9 | 9.46 | 0.02 | 0.7 |
| (A_G_A_G) | 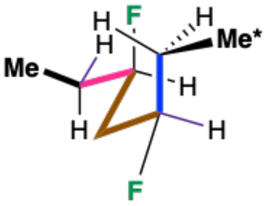 AGAG (identical as GAGA) | -474.7875 | -1246554.5 | 8.85 | 0.03 | 0.9 |
| (A_G_G_A) | 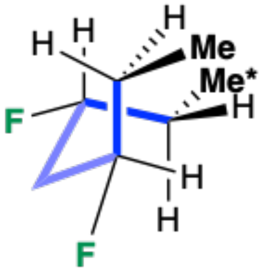 AGGA | -474.7863 | -1246551.6 | 11.83 | 0.01 | 0.27 |
| (A_G_G_G-) | 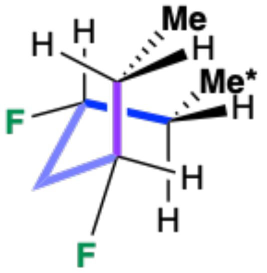 AGGG$^{-}$ | nan | nan | nan | 0 | 0 |
| (A_G_G_G) | 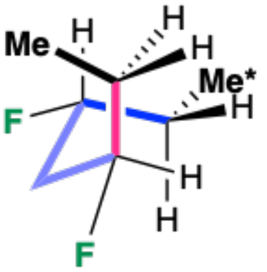 AGGG | -474.7868 | -1246552.7 | 10.71 | 0.01 | 0.42 |

| | | | | | | |
|---|---|---|---|---|---|---|
| (A_G_G-_A) | 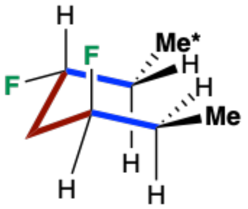 AG$G^-$A (identical as A$G^-$GA) | nan | nan | nan | 0 | 0 |
| (A_G_G-_G- | 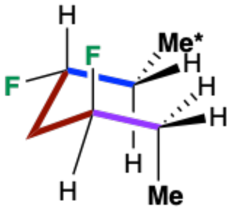 AG$G^-$$G^-$ (identical as $G^-$$G^-$GA) | nan | nan | nan | 0 | 0 |
| (A_G_G-_G) | 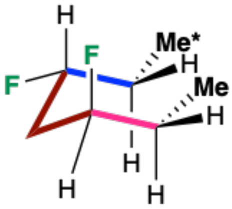 AG$G^-$G (identical as G$G^-$GA) | nan | nan | nan | 0 | 0 |
| (A_G-_A_A) | 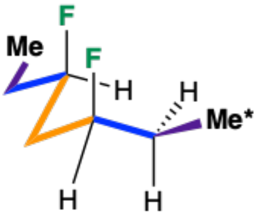 A$G^-$AA (identical as AA$G^-$A) | -474.7869 | -1246552.9 | 10.44 | 0.01 | 0.47 |
| (A_G-_A_G-) | 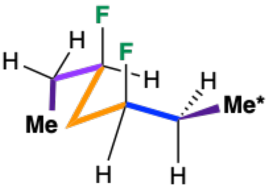 A$G^-$A$G^-$ (identical as $G^-$A$G^-$A) | -474.7871 | -1246553.7 | 9.72 | 0.02 | 0.63 |

| | | | | | | |
|---|---|---|---|---|---|---|
| (A_G-_A_G) | 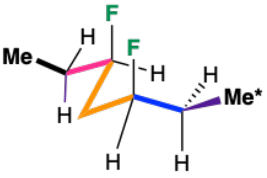 $AG^{-}AG$ (identical as $GAG^{-}A$) | -474.7861 | -1246550.9 | 12.51 | 0.01 | 0.21 |
| (A_G-_G_A) | 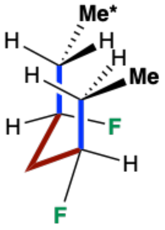 $AG^{-}GA$ (identical as $AGG^{-}A$) | nan | nan | nan | 0 | 0 |
| (A_G-_G_G- | 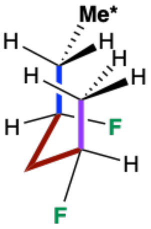 $AG^{-}GG^{-}$ (identical as $G^{-}GG^{-}A$) | nan | nan | nan | 0 | 0 |
| (A_G-_G_G) | 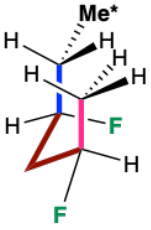 $AG^{-}GG$ (identical as $GGG^{-}A$) | -474.7843 | -1246546.1 | 17.27 | 0 | 0.03 |
| (A_G-_G-_A) | 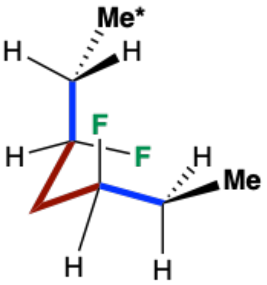 $AG^{-}G^{-}A$ | -474.7856 | -1246549.5 | 13.91 | 0 | 0.12 |

| | | | | | | |
|---|---|---|---|---|---|---|
| (A_G-_G-_G | 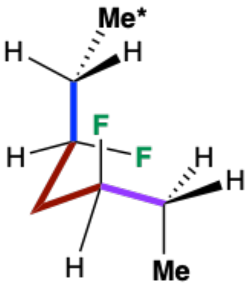<br>$AG^{-}G^{-}G^{-}$ | -474.7847 | -1246547.3 | 16.09 | 0 | 0.05 |
| (A_G-_G-_G | 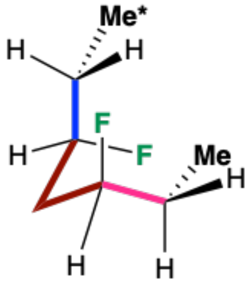<br>$AG^{-}G^{-}G$ | -474.7805 | -1246536.3 | 27.07 | 0 | 0 |
| (G_A_A_A) | 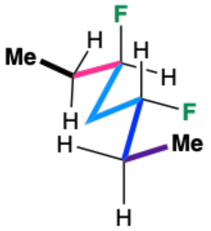<br>GAAA<br>(identical<br>as AAAG) | -474.79 | -1246561 | 2.36 | 0.39 | 12.35 |
| (G_A_A_G-) | 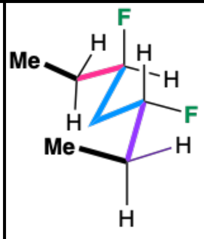<br>$GAAG^{-}$<br>(identical<br>as $G^{-}AAG$) | -474.7884 | -1246556.9 | 6.44 | 0.07 | 2.38 |
| (G_A_A_G) | 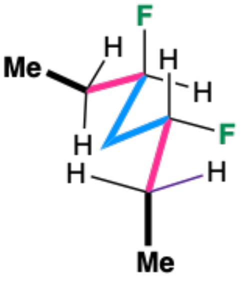<br>GAAG | -474.7882 | -1246556.4 | 6.95 | 0.06 | 1.94 |

| | | | | | | |
|---|---|---|---|---|---|---|
| (G_A_G_A) | 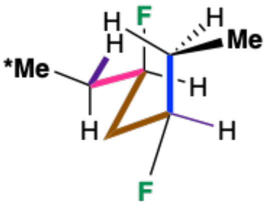 GAGA (identical as AGAG) | -474.7875 | -1246554.5 | 8.85 | 0.03 | 0.9 |
| (G_A_G_G-) | 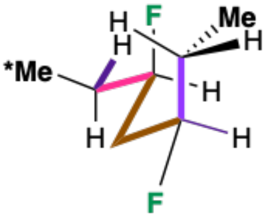 GAGG$^-$ (identical as G$^-$GAG) | -474.7839 | -1246545.2 | 18.21 | 0 | 0.02 |
| (G_A_G_G) | 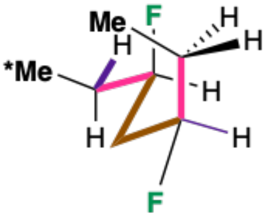 GAGG (identical as GGAG) | -474.7863 | -1246551.4 | 11.98 | 0.01 | 0.25 |
| (G_A_G-_A) | 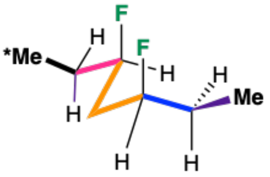 GAG$^-$A (identical as AG$^-$AG) | -474.7861 | -1246550.9 | 12.51 | 0.01 | 0.21 |
| (G_A_G-_G- | 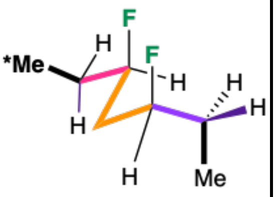 GAG$^-$G$^-$ (identical as G$^-$G$^-$AG) | -474.7863 | -1246551.4 | 11.95 | 0.01 | 0.26 |

| | | | | | | |
|---|---|---|---|---|---|---|
| (G_A_G-_G) | 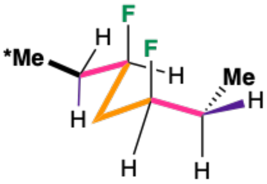 GAG$^-$G (identical as GG$^-$AG) | -474.7827 | -1246542 | 21.37 | 0 | 0.01 |
| (G_G_A_A) | 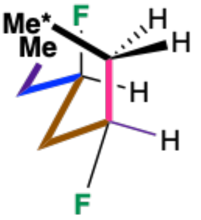 GGAA (identical as AAGG) | -474.7867 | -1246552.6 | 10.83 | 0.01 | 0.4 |
| (G_G_A_G-) | 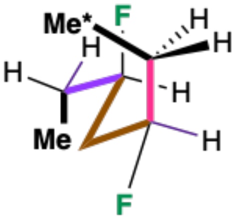 GGAG$^-$ (identical as G$^-$AGG) | -474.7864 | -1246551.7 | 11.67 | 0.01 | 0.29 |
| (G_G_A_G) | 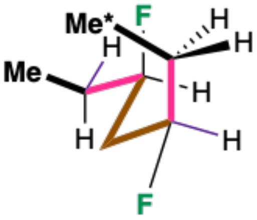 GGAG (identical as GAGG) | -474.7863 | -1246551.4 | 11.98 | 0.01 | 0.25 |
| (G_G_G_A) | 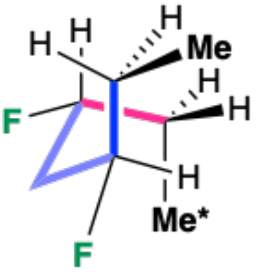 GGGA | -474.7868 | -1246552.7 | 10.71 | 0.01 | 0.42 |

| | | | | | | |
|---|---|---|---|---|---|---|
| (G_G_G_G-) | 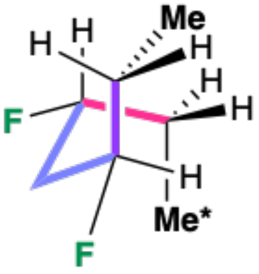 $GGGG^{-}$ | -474.7825 | -1246541.4 | 21.95 | 0 | 0 |
| (G_G_G_G) | 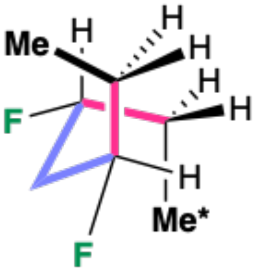 GGGG | -474.7861 | -1246551 | 12.39 | 0.01 | 0.22 |
| (G_G_G-_A) | 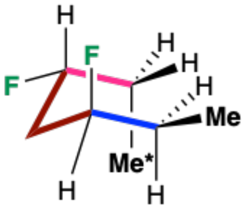 $GGG^{-}A$ (identical as $AG^{-}G^{-}G$) | -474.7843 | -1246546.1 | 17.27 | 0 | 0.03 |
| (G_G_G-_G- | 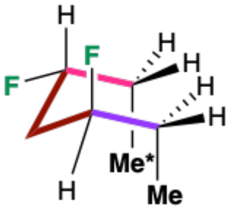 $GGG^{-}G^{-}$ (identical as $G^{-}G^{-}GG$) | -474.7823 | -1246540.9 | 22.49 | 0 | 0 |
| (G_G_G-_G) | 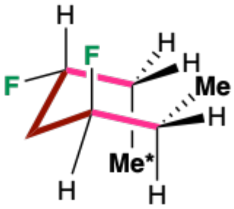 $GGG^{-}G$ (identical as $GG^{-}GG$) | nan | nan | nan | 0 | 0 |

| | | | | | | |
|---|---|---|---|---|---|---|
| (G_G-_A_A) | 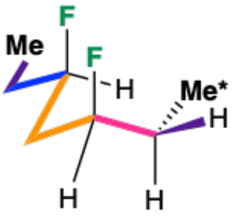 GG$^-$AA (identical as AAG$^-$G) | -474.7834 | -1246543.8 | 19.58 | 0 | 0.01 |
| (G_G-_A_G- | 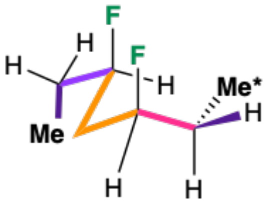 GG$^-$AG$^-$ (identical as G$^-$AG$^-$G) | -474.7829 | -1246542.4 | 20.98 | 0 | 0.01 |
| (G_G-_A_G) | 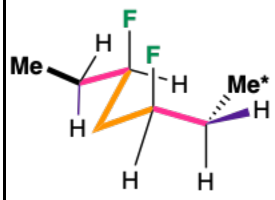 GG$^-$AG (identical as GAG$^-$G) | -474.7827 | -1246542 | 21.37 | 0 | 0.01 |
| (G_G-_G_A) | 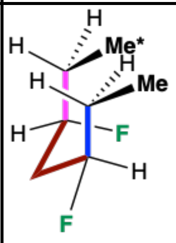 GG$^-$GA (identical as AGG$^-$G) | nan | nan | nan | 0 | 0 |
| (G_G-_G_G- | 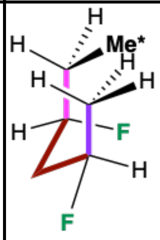 GG$^-$GG$^-$ (identical as G$^-$GG$^-$G) | nan | nan | nan | 0 | 0 |

| | | | | | | |
|---|---|---|---|---|---|---|
| (G_G-_G_G) | 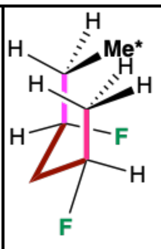<br>$GG^{-}GG$<br>(identical as $GGG^{-}G$) | nan | nan | nan | 0 | 0 |
| (G_G-_G-_A | 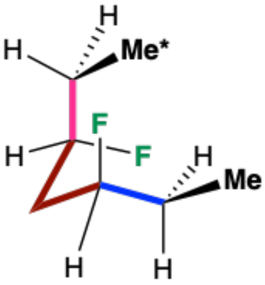<br>$GG^{-}G^{-}A$ | -474.7805 | -1246536.3 | 27.07 | 0 | 0 |
| (G_G-_G-_G | 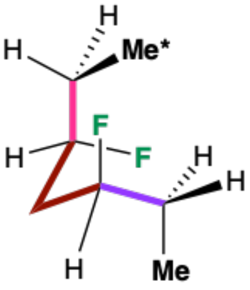<br>$GG^{-}G^{-}G^{-}$ | -474.7792 | -1246532.8 | 30.61 | 0 | 0 |
| (G_G-_G-_G | 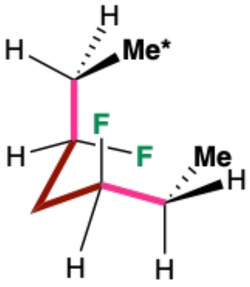<br>$GG^{-}G^{-}G$ | -474.7724 | -1246514.9 | 48.49 | 0 | 0 |
| (G-_A_A_A) | 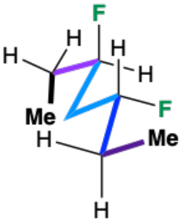<br>$G^{-}AAA$<br>(identical as $AAAG^{-}$) | -474.7897 | -1246560.3 | 3.08 | 0.29 | 9.24 |

| | | | | | | |
|---|---|---|---|---|---|---|
| (G-_A_A_G-) | 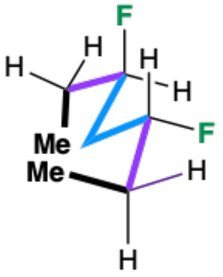 $G^{-}AAG^{-}$ | -474.7882 | -1246556.4 | 6.95 | 0.06 | 1.94 |
| (G-_A_A_G) | 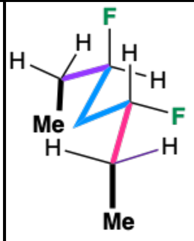 $G^{-}AAG$ (identical as $GAAG^{-}$) | -474.7884 | -1246556.9 | 6.44 | 0.07 | 2.38 |
| (G-_A_G_A) | 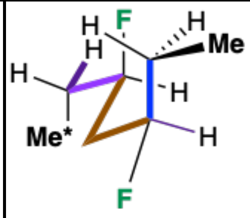 $G^{-}AGA$ (identical as $AGAG^{-}$) | -474.7872 | -1246553.9 | 9.46 | 0.02 | 0.7 |
| (G-_A_G_G-) | 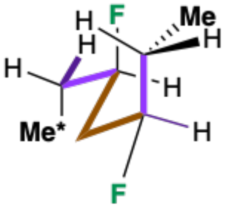 $G^{-}AGG^{-}$ (identical as $G^{-}GAG^{-}$) | -474.7832 | -1246543.2 | 20.2 | 0 | 0.01 |
| (G-_A_G_G) | 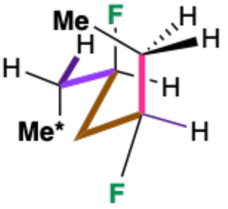 $G^{-}AGG$ (identical as $GGAG^{-}$) | -474.7864 | -1246551.7 | 11.67 | 0.01 | 0.29 |

| | | | | | | |
|---|---|---|---|---|---|---|
| (G-_A_G-_A) | 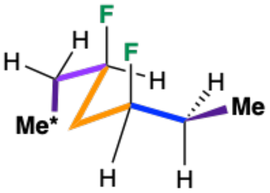 $G^{-}AG^{-}A$ (identical as $AG^{-}AG^{-}$) | -474.7871 | -1246553.7 | 9.72 | 0.02 | 0.63 |
| (G-_A_G-_G | 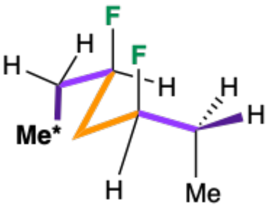 $G^{-}AG^{-}G^{-}$ (identical as $G^{-}G^{-}AG^{-}$) | -474.7867 | -1246552.6 | 10.83 | 0.01 | 0.4 |
| (G-_A_G-_G | 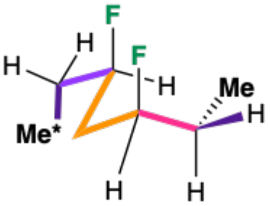 $G^{-}AG^{-}G$ (identical as $GG^{-}AG^{-}$) | -474.7829 | -1246542.4 | 20.98 | 0 | 0.01 |
| (G-_G_A_A) | 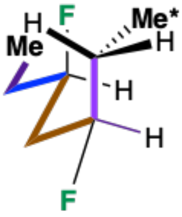 $G^{-}GAA$ (identical as $AAGG^{-}$) | nan | nan | nan | 0 | 0 |
| (G-_G_A_G- | 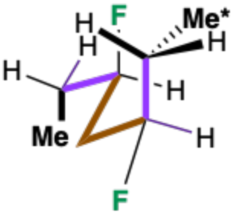 $G^{-}GAG^{-}$ (identical as $G^{-}AGG^{-}$) | -474.7832 | -1246543.2 | 20.2 | 0 | 0.01 |

| | | | | | | |
|---|---|---|---|---|---|---|
| (G-_G_A_G) | 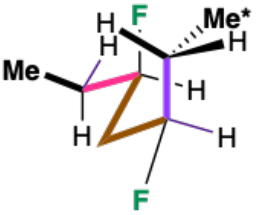 G$^{-}$GAG (identical as GAGG$^{-}$) | -474.7839 | -1246545.2 | 18.21 | 0 | 0.02 |
| (G-_G_G_A) | 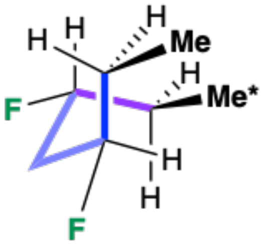 G$^{-}$GGA | nan | nan | nan | 0 | 0 |
| (G-_G_G_G- | 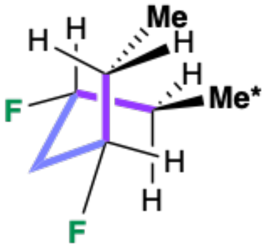 G$^{-}$GGG$^{-}$ | -474.7812 | -1246538.1 | 25.28 | 0 | 0 |
| (G-_G_G_G) | 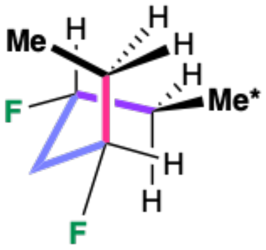 G$^{-}$GGG | -474.7825 | -1246541.4 | 21.95 | 0 | 0 |
| (G-_G_G-_A | 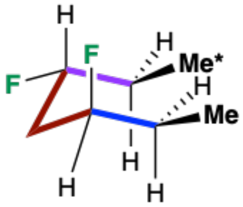 G$^{-}$GG$^{-}$A (identical as AG$^{-}$GG$^{-}$) | nan | nan | nan | 0 | 0 |

| | | | | | | |
|---|---|---|---|---|---|---|
| (G-_G_G-_G | 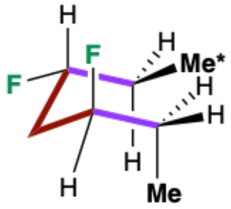 $G^-GG^-G^-$ (identical as $G^-G^-GG^-$) | nan | nan | nan | 0 | 0 |
| (G-_G_G-_G | 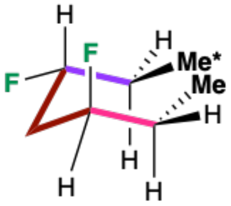 $G^-GG^-G$ (identical as $GG^-GG^-$) | nan | nan | nan | 0 | 0 |
| (G-_G-_A_A) | 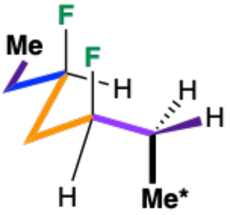 $G^-G^-AA$ (identical as $AAG^-G^-$) | -474.7873 | -1246554.1 | 9.27 | 0.02 | 0.76 |
| (G-_G-_A_G | 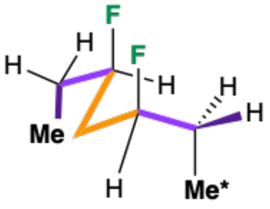 $G^-G^-AG^-$ (identical as $G^-AG^-G^-$) | -474.7867 | -1246552.6 | 10.83 | 0.01 | 0.4 |
| (G-_G-_A_G | 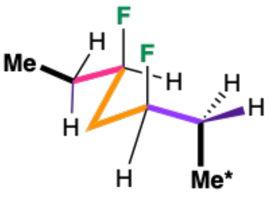 $G^-G^-AG$ (identical as $GAG^-G^-$) | -474.7863 | -1246551.4 | 11.95 | 0.01 | 0.26 |

| | | | | | | |
|---|---|---|---|---|---|---|
| (G-_G-_G_A | 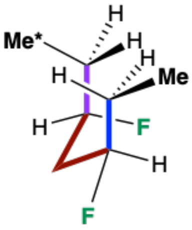 $G^{-}G^{-}GA$ (identical as $AGG^{-}G^{-}$) | nan | nan | nan | 0 | 0 |
| (G-_G-_G_G | 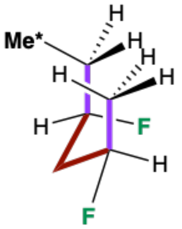 $G^{-}G^{-}GG^{-}$ (identical as $G^{-}GG^{-}G^{-}$) | nan | nan | nan | 0 | 0 |
| (G-_G-_G_G | 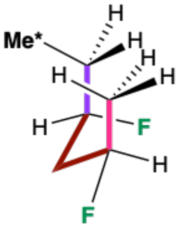 $G^{-}G^{-}GG$ (identical as $GGG^{-}G^{-}$) | -474.7823 | -1246540.9 | 22.49 | 0 | 0 |
| (G-_G-_G-_A | 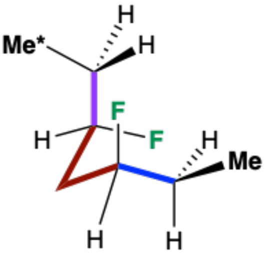 $G^{-}G^{-}G^{-}A$ | -474.7847 | -1246547.3 | 16.09 | 0 | 0.05 |
| (G-_G-_G-_G | 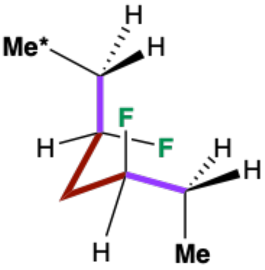 $G^{-}G^{-}G^{-}G^{-}$ | -474.7831 | -1246543.1 | 20.29 | 0 | 0.01 |

| | | | | | | |
|---|---|---|---|---|---|---|
| (G-_G-_G-_G | 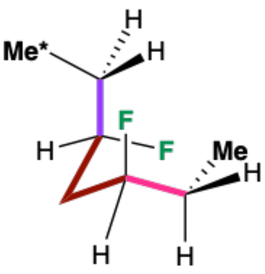 $G^-G^-G^-G$ | -474.7792 | -1246532.8 | 30.61 | 0 | 0 |
