# Supplementary material for: Conformational Analysis of 1,3-Difluorinated Alkanes
Source: J Org Chem. 2024 May 31;89(12):8789–803. doi: 10.1021/acs.joc.4c00670 (PMC11197103; doi:10.1021/acs.joc.4c00670)
Supplement: Supplementary file 2 — jo4c00670_si_004.zip [file jo4c00670_si_004.zip › SI/raw_data/difluoroheptane/syn-heptane-raw-water.pdf]

Conformer		Energy (Hart	Energy (kJ/n	Relative Ene	Population	Population %
(G-_G-_G-_G-)	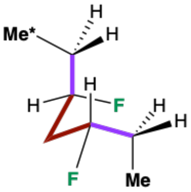<br>G-G-G-G-<br>(enantiomeric with<br>GGGG)	-474.7855	-1246549.3	8.1	0.04	0.27
(G-_G-_G-_G)	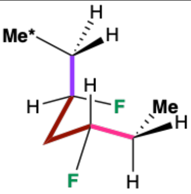<br>G-G-G-G<br>(enantiomeric with<br>G-GGG)	nan	nan	nan	0	0
(G-_G-_G-_A)	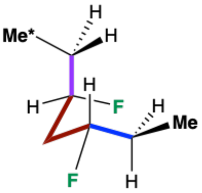<br>G-G-G-A<br>(enantiomeric with<br>AGGG)	-474.7863	-1246551.4	6.03	0.09	0.63
(G-_G-_G_G-)	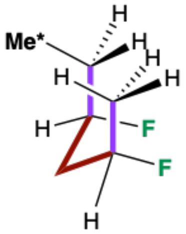<br>G-G-GG-	nan	nan	nan	0	0
(G-_G-_G_G)	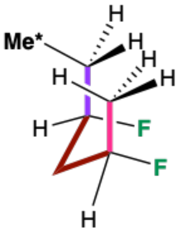<br>G-G-GG	-474.7804	-1246535.9	21.52	0	0
(G-_G-_G_A)	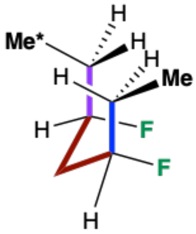<br>G-G-GA	-474.7813	-1246538.3	19.09	0	0

(G-_G-_A_G-)	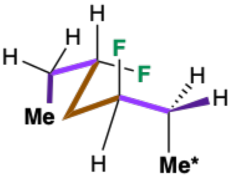 $G^-G^-AG^-$ (enantiomeric with $GAGG$)	-474.7866	-1246552.2	5.17	0.12	0.89
(G-_G-_A_G)	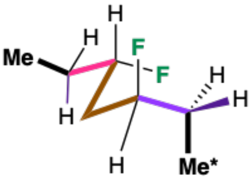 G^-G^-AG (enantiomeric with G^-AGG)	-474.7873	-1246554	3.46	0.25	1.78
(G-_G-_A_A)	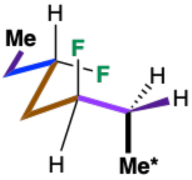 G^-G^-AA (enantiomeric with $AAGG$)	-474.7876	-1246554.8	2.64	0.34	2.48
(G-_G_G-_G-)	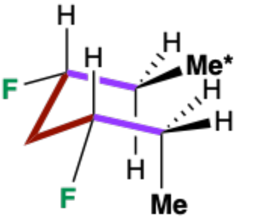 $G^-GG^-G^-$	nan	nan	nan	0	0
(G-_G_G-_G)	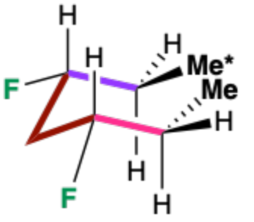 G^-GG^-G	nan	nan	nan	0	0
(G-_G_G-_A)	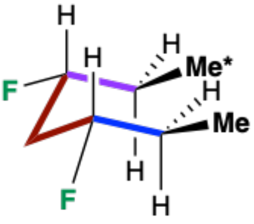 G^-GG^-A	nan	nan	nan	0	0

(G-_G_G_G-)	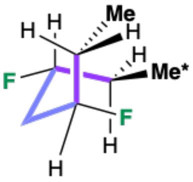 $G^{-}GGG^{-}$ (enantiomeric with $GG^{-}G^{-}G$)	nan	nan	nan	0	0
(G-_G_G_G)	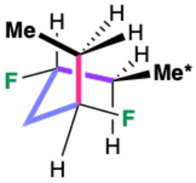 $G^{-}GGG$ (enantiomeric with $G^{-}G^{-}G^{-}G$)	nan	nan	nan	0	0
(G-_G_G_A)	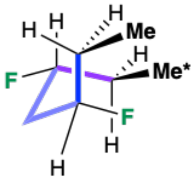 $G^{-}GGA$ (enantiomeric with $AG^{-}G^{-}G$)	nan	nan	nan	0	0
(G-_G_A_G-)	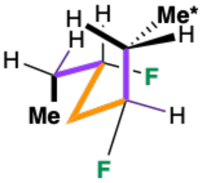 $G^{-}GAG^{-}$ (enantiomeric with $GAG^{-}G$)	nan	nan	nan	0	0
(G-_G_A_G)	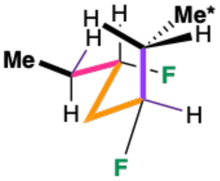 $G^{-}GAG$ (enantiomeric with $G^{-}AG^{-}G$)	nan	nan	nan	0	0
(G-_G_A_A)	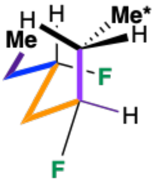 $G^{-}GAA$ (enantiomeric with $AAG^{-}G$)	nan	nan	nan	0	0

(G-_A_G-_G-)	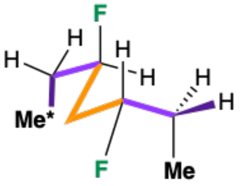<br>$G^-AG^-G^-$<br>(enantiomeric with GGAG)	-474.7873	-1246554.1	3.31	0.26	1.89
(G-_A_G-_G)	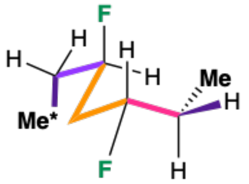<br>G^-AG^-G<br>(enantiomeric with G^-GAG)	nan	nan	nan	0	0
(G-_A_G-_A)	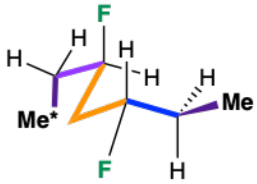<br>G^-AG^-A<br>(enantiomeric with AGAG)	-474.7877	-1246555.1	2.3	0.4	2.84
(G-_A_G_G-)	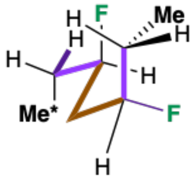<br>G^-AGG^-<br>(enantiomeric with GG^-AG)	-474.783	-1246542.7	14.7	0	0.02
(G-_A_G_G)	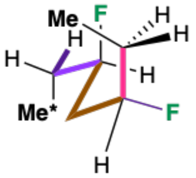<br>G^-AGG<br>(enantiomeric with G^-G^-AG)	-474.7873	-1246554	3.46	0.25	1.78
(G-_A_G_A)	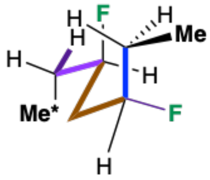<br>G^-AGA<br>(enantiomeric with AG^-AG)	-474.7876	-1246554.9	2.56	0.36	2.56

(G-_A_A_G-)	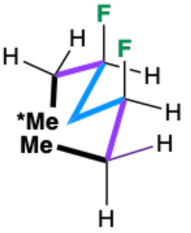 $G^{-}AAG^{-}$	-474.7873	-1246554.1	3.28	0.27	1.91
(G-_A_A_G)	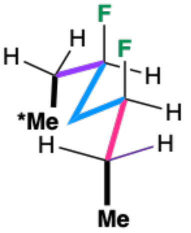 $G^{-}AAG$	-474.7872	-1246553.9	3.52	0.24	1.74
(G-_A_A_A)	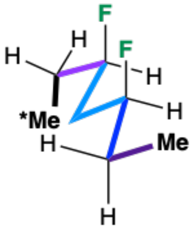 $G^{-}AAA$	-474.7879	-1246555.7	1.7	0.5	3.62
(G_G-_G-_G-)	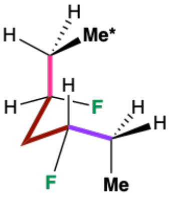 $GG^{-}G^{-}G^{-}$ (enantiomeric with $GGGG^{-}$)	-474.7807	-1246536.7	20.76	0	0
(G_G-_G-_G)	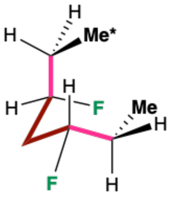 $GG^{-}G^{-}G$ (enantiomeric with $G^{-}GGG^{-}$)	nan	nan	nan	0	0
(G_G-_G-_A)	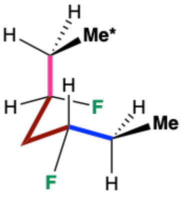 $GG^{-}G^{-}A$ (enantiomeric with $AGGG^{-}$)	-474.7827	-1246542	15.37	0	0.01

(G_G-_G_G-)	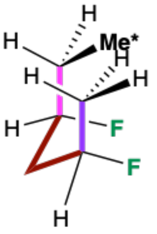<br>$GG^{-}GG^{-}$	nan	nan	nan	0	0
(G_G-_G_G)	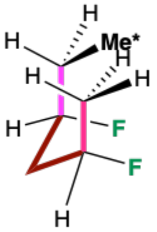<br>$GG^{-}GG$	nan	nan	nan	0	0
(G_G-_G_A)	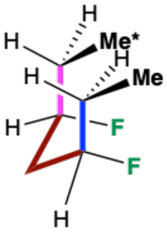<br>$GG^{-}GA$	nan	nan	nan	0	0
(G_G-_A_G-)	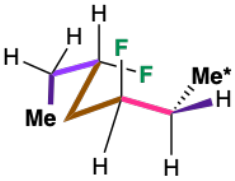<br>$GG^{-}AG^{-}$<br>(enantiomeric with<br>$GAGG^{-}$)	-474.7825	-1246541.4	16.03	0	0.01
(G_G-_A_G)	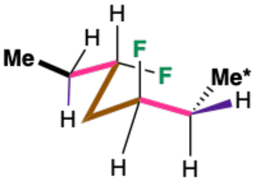<br>$GG^{-}AG$<br>(enantiomeric with<br>$G^{-}AGG^{-}$)	-474.783	-1246542.7	14.7	0	0.02
(G_G-_A_A)	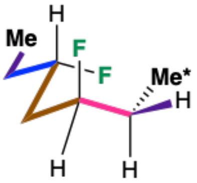<br>$GG^{-}AA$<br>(enantiomeric with<br>$AAGG^{-}$)	-474.7823	-1246540.9	16.5	0	0.01

(G_G_G-_G-)	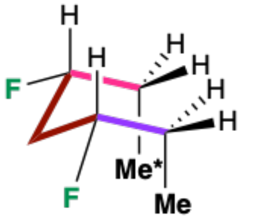 $GGG^{-}G^{-}$	nan	nan	nan	0	0
(G_G_G-_G)	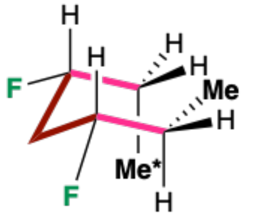 $GGG^{-}G$	nan	nan	nan	0	0
(G_G_G-_A)	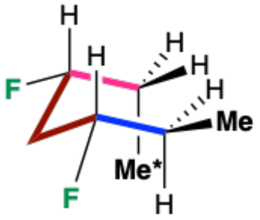 $GGG^{-}A$	nan	nan	nan	0	0
(G_G_G_G-)	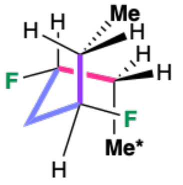 $GGGG^{-}$ (enantiomeric with $GG^{-}G^{-}G^{-}$)	-474.7807	-1246536.7	20.76	0	0
(G_G_G_G)	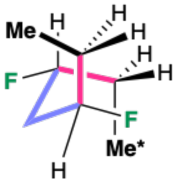 $GGGG$ (enantiomeric with $G^{-}G^{-}G^{-}G^{-}$)	-474.7855	-1246549.3	8.1	0.04	0.27
(G_G_G_A)	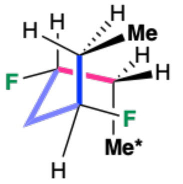 $GGGA$ (enantiomeric with $AG^{-}G^{-}G^{-}$)	-474.7864	-1246551.6	5.82	0.1	0.69

(G_G_A_G-)	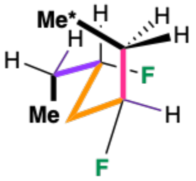<br>GGAG^{-}<br>(enantiomeric with<br>GAG^{-}G^{-})	-474.7873	-1246554.1	3.36	0.26	1.85
(G_G_A_G)	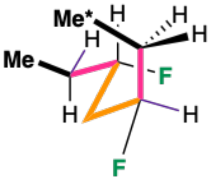<br>GGAG<br>(enantiomeric with<br>G^{-}AG^{-}G^{-})	-474.7873	-1246554.1	3.31	0.26	1.89
(G_G_A_A)	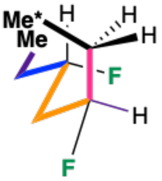<br>GGAA<br>(enantiomeric with<br>AAG^{-}G^{-})	-474.7883	-1246556.6	0.79	0.73	5.22
(G_A_G-_G-)	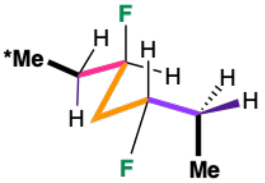<br>GAG^{-}G^{-}<br>(enantiomeric with<br>GGAG^{-})	-474.7873	-1246554.1	3.36	0.26	1.85
(G_A_G-_G)	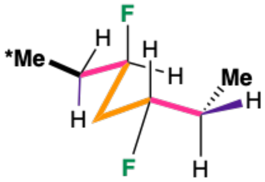<br>GAG^{-}G<br>(enantiomeric with<br>G^{-}GAG^{-})	nan	nan	nan	0	0
(G_A_G-_A)	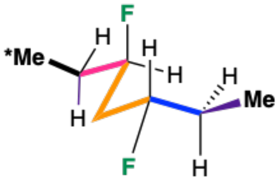<br>GAG^{-}A<br>(enantiomeric with<br>AGAG^{-})	-474.7876	-1246554.9	2.53	0.36	2.59

(G_A_G_G-)	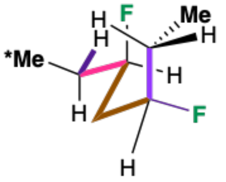 GAGG^{-} (enantiomeric with GG^{-}AG^{-})	-474.7825	-1246541.4	16.03	0	0.01
(G_A_G_G)	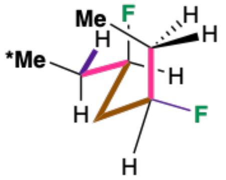 GAGG (enantiomeric with G^{-}G^{-}AG^{-})	-474.7866	-1246552.2	5.17	0.12	0.89
(G_A_G_A)	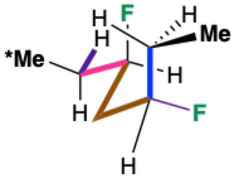 GAGA (enantiomeric with AG^{-}AG^{-})	-474.7876	-1246554.9	2.56	0.36	2.56
(G_A_A_G-)	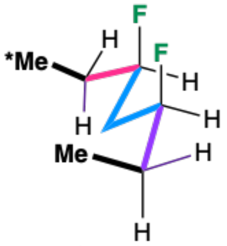 GAAG^{-}	-474.7872	-1246553.7	3.72	0.22	1.6
(G_A_A_G)	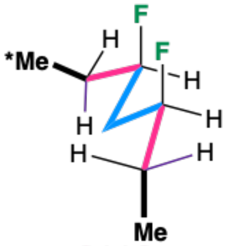 GAAG	-474.7873	-1246554.1	3.28	0.27	1.91
(G_A_A_A)	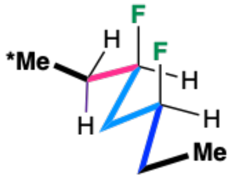 GAAA	-474.7877	-1246555.1	2.28	0.4	2.86

(A_G-_G-_G-)	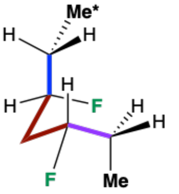 AG$^-$G$^-$G$^-$ (enantiomeric with GGGA)	-474.7864	-1246551.6	5.82	0.1	0.69
(A_G-_G-_G)	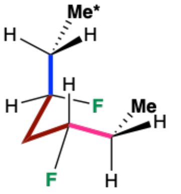 AG$^-$G$^-$G (enantiomeric with G$^-$GGA)	nan	nan	nan	0	0
(A_G-_G-_A)	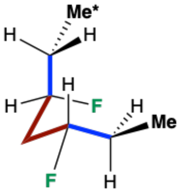 AG$^-$G$^-$A (enantiomeric with AGGA)	-474.7864	-1246551.7	5.76	0.1	0.7
(A_G-_G_G-)	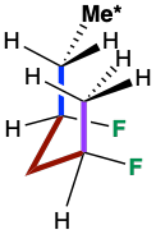 AG$^-$GG$^-$	nan	nan	nan	0	0
(A_G-_G_G)	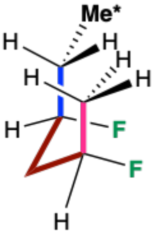 AG$^-$GG	-474.7813	-1246538.3	19.09	0	0
(A_G-_G_A)	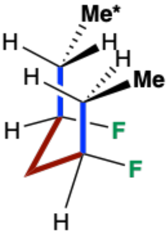 AG$^-$GA	-474.7828	-1246542.3	15.1	0	0.02

(A_G-_A_G-)	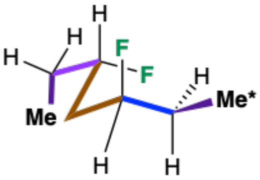<br>AG-AG-<br>(enantiomeric with<br>GAGA)	-474.7876	-1246554.9	2.56	0.36	2.56
(A_G-_A_G)	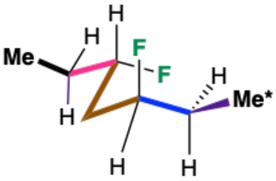<br>AG-AG<br>(enantiomeric with<br>G-AGA)	-474.7876	-1246554.9	2.56	0.36	2.56
(A_G-_A_A)	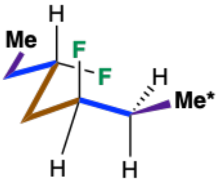<br>AG-AA<br>(enantiomeric with<br>AAGA)	-474.7886	-1246557.4	0	1	7.19
(A_G_G-_G-)	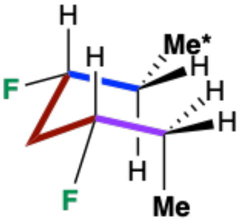<br>AGG-G-	nan	nan	nan	0	0
(A_G_G-_G)	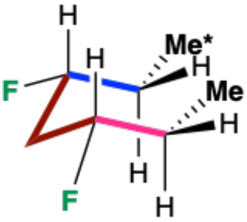<br>AGG-G	nan	nan	nan	0	0
(A_G_G-_A)	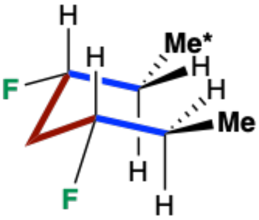<br>AGG-A	nan	nan	nan	0	0

(A_G_G_G-)	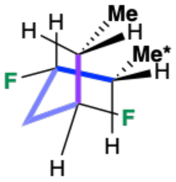 AGGG$^-$ (enantiomeric with GG$^-$G$^-$A)	-474.7827	-1246542	15.37	0	0.01
(A_G_G_G)	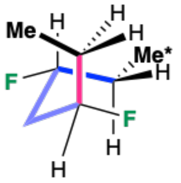 AGGG (enantiomeric with G$^-$G$^-$G$^-$A)	-474.7863	-1246551.4	6.03	0.09	0.63
(A_G_G_A)	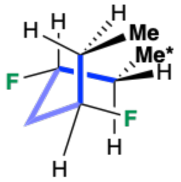 AGGA (enantiomeric with AG$^-$G$^-$A)	-474.7864	-1246551.7	5.76	0.1	0.7
(A_G_A_G-)	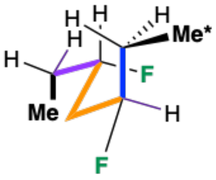 AGAG$^-$ (enantiomeric with GAG$^-$A)	-474.7876	-1246554.9	2.53	0.36	2.59
(A_G_A_G)	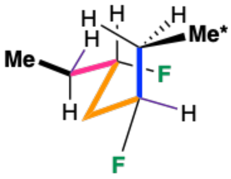 AGAG (enantiomeric with G$^-$AG$^-$A)	-474.7877	-1246555.1	2.3	0.4	2.84
(A_G_A_A)	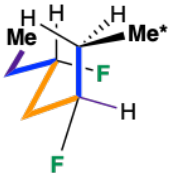 AGAA (enantiomeric with AAG$^-$A)	-474.788	-1246555.9	1.52	0.54	3.89

(A_A_G-_G-)	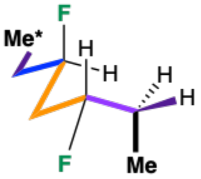 AAG-G- (enantiomeric with GGAA)	-474.7883	-1246556.6	0.79	0.73	5.22
(A_A_G-_G)	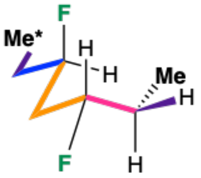 AAG-G (enantiomeric with G-GAA)	nan	nan	nan	0	0
(A_A_G-_A)	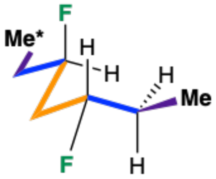 AAG-A (enantiomeric with AGAA)	-474.788	-1246555.9	1.52	0.54	3.89
(A_A_G_G-)	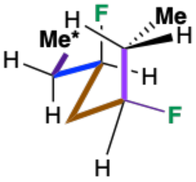 AAGG- (enantiomeric with GG-AA)	-474.7823	-1246540.9	16.5	0	0.01
(A_A_G_G)	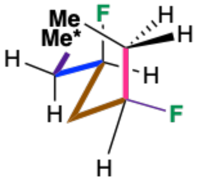 AAGG (enantiomeric with G-G-AA)	-474.7876	-1246554.8	2.64	0.34	2.48
(A_A_G_A)	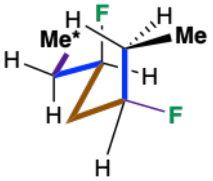 AAGA (enantiomeric with AG-AA)	-474.7886	-1246557.4	0	1	7.19

(A_A_A_G-)	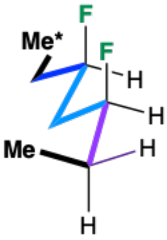 AAAG⁻	-474.7877	-1246555.1	2.28	0.4	2.86
(A_A_A_G)	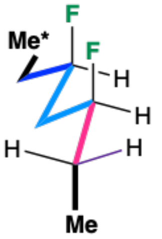 AAAG	-474.7879	-1246555.7	1.7	0.5	3.62
(A_A_A_A)	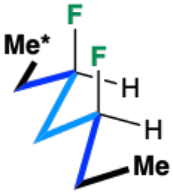 AAAA	-474.788	-1246555.8	1.64	0.52	3.71
