# Supplementary material for: Conformational Analysis of 1,3-Difluorinated Alkanes
Source: J Org Chem. 2024 May 31;89(12):8789–803. doi: 10.1021/acs.joc.4c00670 (PMC11197103; doi:10.1021/acs.joc.4c00670)
Supplement: Supplementary file 2 — jo4c00670_si_004.zip [file jo4c00670_si_004.zip › SI/raw_data/difluoroheptane/anti-heptane-raw-chloroform.pdf]

Conformer		Energy (Hart	Energy (kJ/n	Relative Ene	Population	Population %
(A_A_A_A)	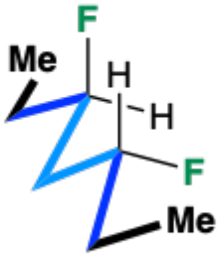 AAAA	-474.7987	-1246583.9	0	1	26.28
(A_A_A_G-)	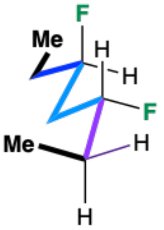 AAAG$^-$ (identical as G$^-$AAA)	-474.7978	-1246581.7	2.24	0.4	10.64
(A_A_A_G)	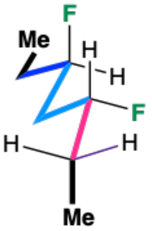 AAAG (identical as GAAA)	-474.798	-1246582.1	1.89	0.47	12.25
(A_A_G_A)	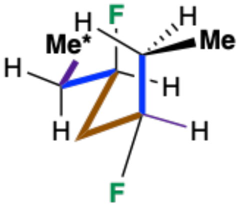 AAGA (identical as AGAA)	-474.7959	-1246576.5	7.43	0.05	1.31
(A_A_G_G-)	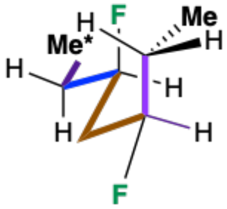 AAGG$^-$ (identical as G$^-$GAA)	nan	nan	nan	0	0

(A_A_G_G)	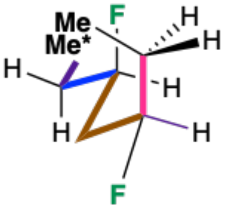<br>AAGG<br>(identical as<br>GGAA)	-474.7948	-1246573.8	10.18	0.02	0.43
(A_A_G-_A)	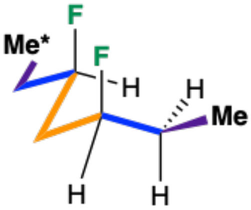<br>$AAG^{-}A$<br>(identical as<br>$AG^{-}AA$)	-474.7936	-1246570.7	13.27	0	0.12
(A_A_G-_G-)	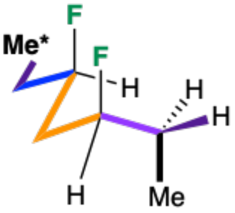<br>$AAG^{-}G^{-}$<br>(identical as<br>$G^{-}G^{-}AA$)	-474.7939	-1246571.3	12.61	0.01	0.16
(A_A_G-_G)	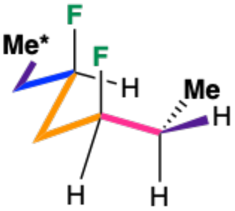<br>$AAG^{-}G$<br>(identical as<br>$GG^{-}AA$)	-474.7904	-1246562.2	21.76	0	0
(A_G_A_A)	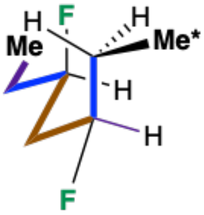<br>AGAA<br>(identical as<br>AAGA)	-474.7959	-1246576.5	7.43	0.05	1.31

(A_G_A_G-)	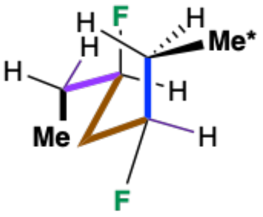 AGAG^{-} (identical as G^{-}AGA)	-474.7953	-1246575.1	8.84	0.03	0.74
(A_G_A_G)	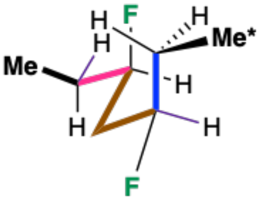 AGAG (identical as GAGA)	-474.7956	-1246575.8	8.17	0.04	0.97
(A_G_G_A)	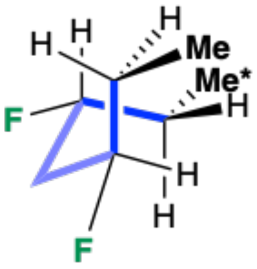 AGGA	-474.7943	-1246572.4	11.54	0.01	0.25
(A_G_G_G-)	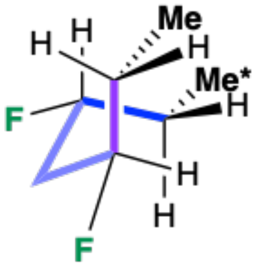 AGGG^{-}	nan	nan	nan	0	0
(A_G_G_G)	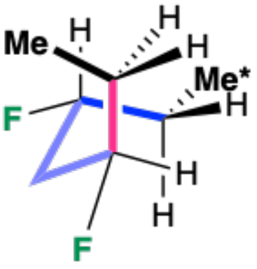 AGGG	-474.7943	-1246572.5	11.47	0.01	0.26

(A_G_G-_A)	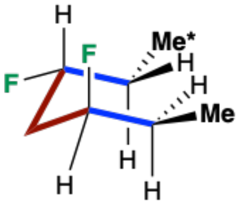<br>AGG^{-}A<br>(identical as<br>AG^{-}GA)	nan	nan	nan	0	0
(A_G_G-_G-)	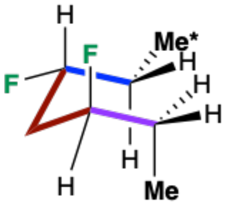<br>AG$G^{-}$$G^{-}$<br>(identical as<br>$G^{-}$$G^{-}$GA)	nan	nan	nan	0	0
(A_G_G-_G)	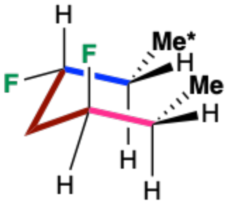<br>AGG^{-}G<br>(identical as<br>GG^{-}GA)	nan	nan	nan	0	0
(A_G-_A_A)	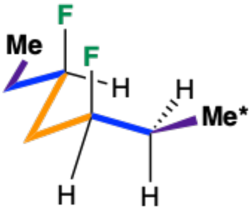<br>AG^{-}AA<br>(identical as<br>AAG^{-}A)	-474.7936	-1246570.7	13.27	0	0.12
(A_G-_A_G-)	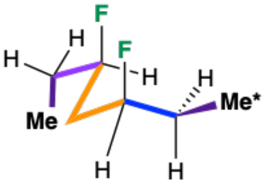<br>AG^{-}AG^{-}<br>(identical as<br>G^{-}AG^{-}A)	-474.7932	-1246569.6	14.34	0	0.08

(A_G-_A_G)	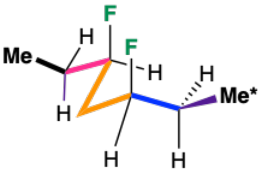 $AG^{-}AG$ (identical as $GAG^{-}A$)	-474.7929	-1246568.7	15.29	0	0.05
(A_G-_G_A)	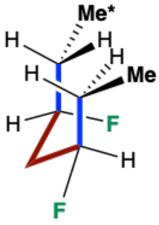 $AG^{-}GA$ (identical as $AGG^{-}A$)	nan	nan	nan	0	0
(A_G-_G_G-)	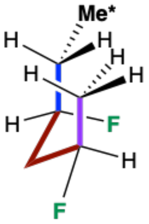 $AG^{-}GG^{-}$ (identical as $G^{-}GG^{-}A$)	nan	nan	nan	0	0
(A_G-_G_G)	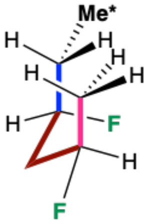 $AG^{-}GG$ (identical as $GGG^{-}A$)	-474.7909	-1246563.4	20.56	0	0.01
(A_G-_G-_A)	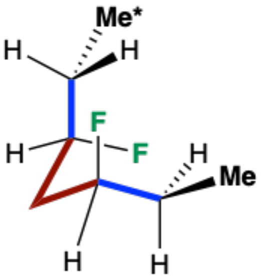 $AG^{-}G^{-}A$	-474.7935	-1246570.4	13.5	0	0.11

(A_G-_G-_G-)	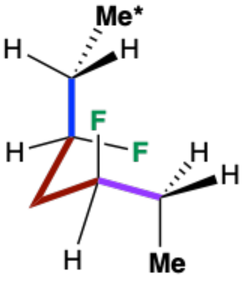<br>$AG^{-}G^{-}G^{-}$	-474.7925	-1246567.7	16.22	0	0.04
(A_G-_G-_G)	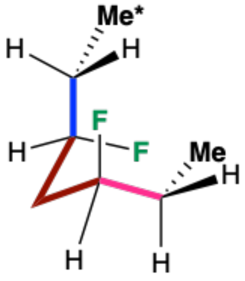<br>$AG^{-}G^{-}G$	-474.7874	-1246554.2	29.72	0	0
(G_A_A_A)	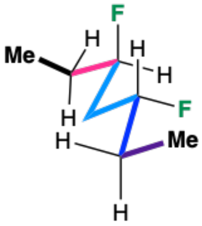<br>*GAAA*<br>(identical as *AAAG*)	-474.798	-1246582.1	1.89	0.47	12.25
(G_A_A_G-)	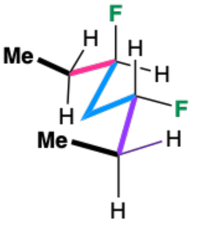<br>$GAAG^{-}$<br>(identical as $G^{-}AAG$)	-474.7973	-1246580.4	3.51	0.24	6.37
(G_A_A_G)	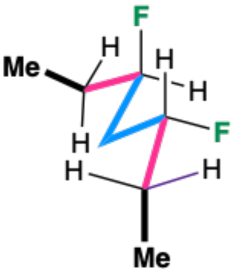<br>*GAAG*	-474.7964	-1246578.1	5.87	0.09	2.46

(G_A_G_A)	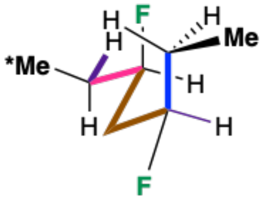<br>GAGA<br>(identical as AGAG)	-474.7956	-1246575.8	8.17	0.04	0.97
(G_A_G_G-)	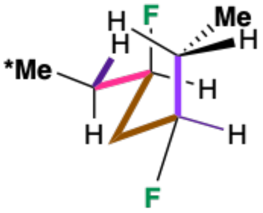<br>GAGG^-<br>(identical as G^-GAG)	nan	nan	nan	0	0
(G_A_G_G)	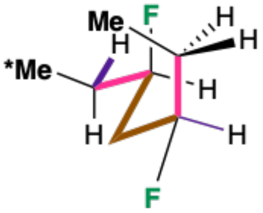<br>GAGG<br>(identical as GGAG)	-474.7946	-1246573.2	10.73	0.01	0.35
(G_A_G-_A)	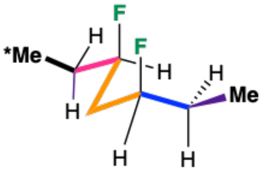<br>GAG^-A<br>(identical as AG^-AG)	-474.7929	-1246568.7	15.29	0	0.05
(G_A_G-_G-)	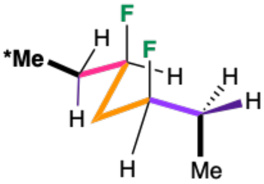<br>GAG^-G^-<br>(identical as G^-G^-AG)	-474.7927	-1246568.1	15.8	0	0.04

(G_A_G-_G)	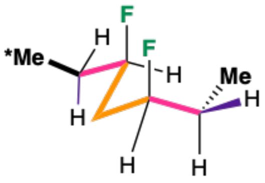 GAG^{-}G (identical as GG^{-}AG)	-474.7894	-1246559.7	24.26	0	0
(G_G_A_A)	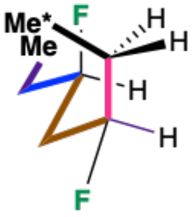 GGAA (identical as AAGG)	-474.7948	-1246573.8	10.18	0.02	0.43
(G_G_A_G-)	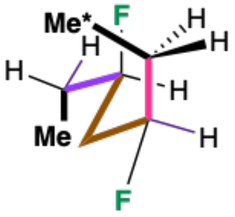 GGAG^{-} (identical as G^{-}AGG)	-474.7944	-1246572.7	11.2	0.01	0.29
(G_G_A_G)	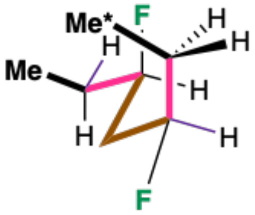 GGAG (identical as GAGG)	-474.7946	-1246573.2	10.73	0.01	0.35
(G_G_G_A)	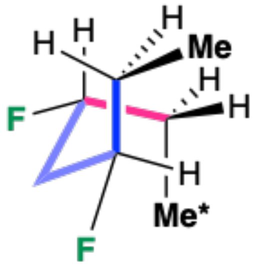 GGGA	-474.7943	-1246572.5	11.47	0.01	0.26

(G_G_G_G-)	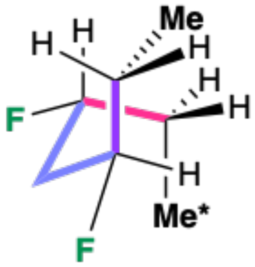 GGGG$^-$	nan	nan	nan	0	0
(G_G_G_G)	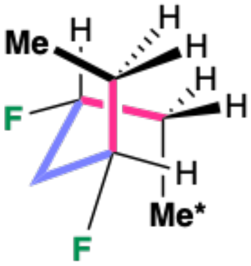 GGGG	-474.7933	-1246569.8	14.19	0	0.09
(G_G_G-_A)	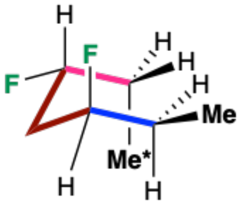 GGG$^-$A (identical as AG$^-$G$^-$G)	-474.7909	-1246563.4	20.56	0	0.01
(G_G_G-_G-)	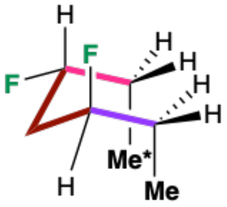 GGG$^-$G$^-$ (identical as G$^-$G$^-$GG)	-474.7894	-1246559.5	24.49	0	0
(G_G_G-_G)	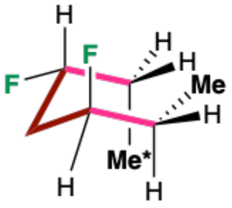 GGG$^-$G (identical as GG$^-$GG)	nan	nan	nan	0	0

(G_G-_A_A)	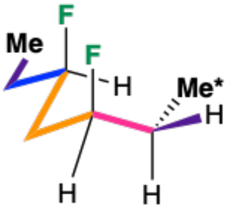 GG-AA (identical as AAG-G)	-474.7904	-1246562.2	21.77	0	0
(G_G-_A_G-)	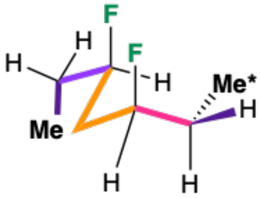 GG-AG- (identical as G-AG-G)	-474.7883	-1246556.7	27.21	0	0
(G_G-_A_G)	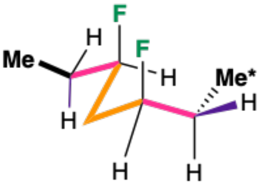 GG-AG (identical as GAG-G)	-474.7894	-1246559.7	24.26	0	0
(G_G-_G_A)	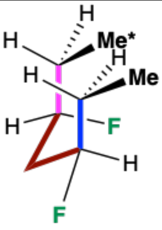 GG-GA (identical as AGG-G)	nan	nan	nan	0	0
(G_G-_G_G-)	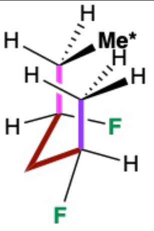 GG-GG- (identical as G-GG-G)	nan	nan	nan	0	0

(G_G-_G_G)	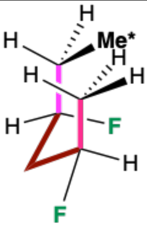 $GG^{-}GG$ (identical as $GGG^{-}G$)	nan	nan	nan	0	0
(G_G-_G-_A)	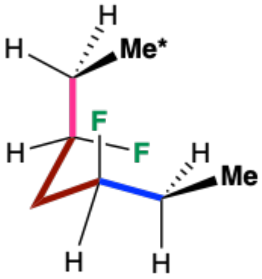 $GG^{-}G^{-}A$	-474.7874	-1246554.2	29.72	0	0
(G_G-_G-_G-)	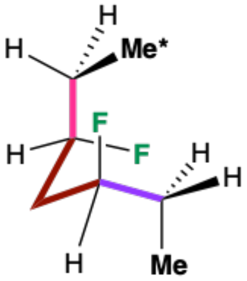 $GG^{-}G^{-}G^{-}$	-474.7872	-1246553.8	30.13	0	0
(G_G-_G-_G)	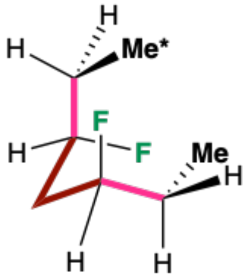 $GG^{-}G^{-}G$	-474.7797	-1246534.2	49.74	0	0
(G-_A_A_A)	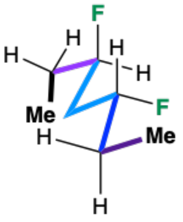 $G^{-}AAA$ (identical as $AAAG^{-}$)	-474.7978	-1246581.7	2.24	0.4	10.64

(G-_A_A_G-)	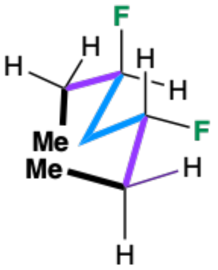<br>$G^{-}AAG^{-}$	-474.7964	-1246578	5.9	0.09	2.43
(G-_A_A_G)	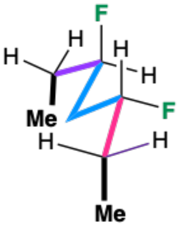<br>$G^{-}AAG$<br>(identical as $GAAG^{-}$)	-474.7973	-1246580.4	3.51	0.24	6.37
(G-_A_G_A)	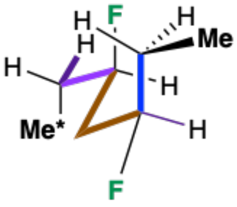<br>$G^{-}AGA$<br>(identical as $AGAG^{-}$)	-474.7953	-1246575.1	8.84	0.03	0.74
(G-_A_G_G-)	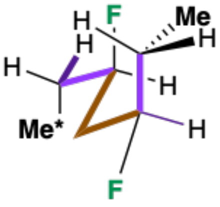<br>$G^{-}AGG^{-}$<br>(identical as $G^{-}GAG^{-}$)	-474.7918	-1246565.8	18.15	0	0.02
(G-_A_G_G)	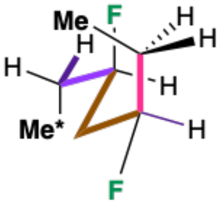<br>$G^{-}AGG$<br>(identical as $GGAG^{-}$)	-474.7944	-1246572.7	11.2	0.01	0.29

(G-_A_G-_A)	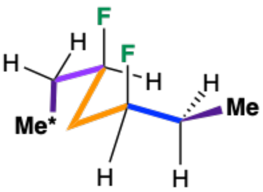<br>$G^{-}AG^{-}A$<br>(identical as<br>$AG^{-}AG^{-}$)	-474.7932	-1246569.6	14.34	0	0.08
(G-_A_G-_G-)	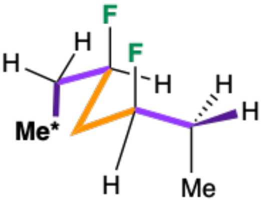<br>$G^{-}AG^{-}G^{-}$<br>(identical as<br>$G^{-}G^{-}AG^{-}$)	-474.7928	-1246568.4	15.55	0	0.05
(G-_A_G-_G)	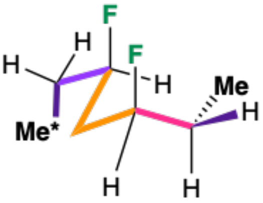<br>$G^{-}AG^{-}G$<br>(identical as<br>$GG^{-}AG^{-}$)	-474.7883	-1246556.7	27.2	0	0
(G-_G_A_A)	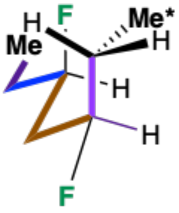<br>$G^{-}GAA$<br>(identical as<br>$AAGG^{-}$)	nan	nan	nan	0	0
(G-_G_A_G-)	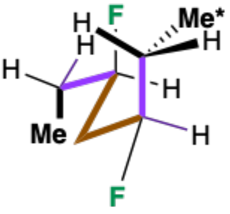<br>$G^{-}GAG^{-}$<br>(identical as<br>$G^{-}AGG^{-}$)	-474.7918	-1246565.8	18.15	0	0.02

(G-_G_A_G)	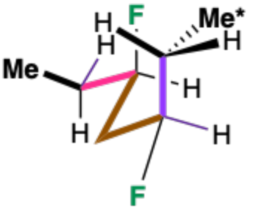 G^{-}GAG (identical as GAGG^{-})	nan	nan	nan	0	0
(G-_G_G_A)	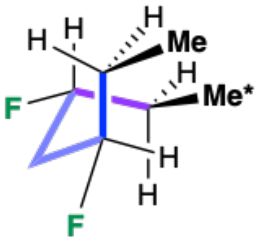 G^{-}GGA	nan	nan	nan	0	0
(G-_G_G_G-)	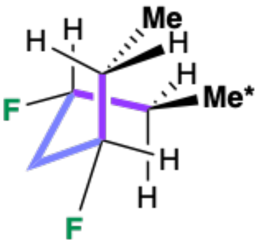 G^{-}GGG^{-}	nan	nan	nan	0	0
(G-_G_G_G)	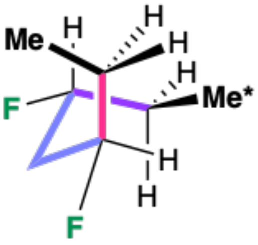 G^{-}GGG	nan	nan	nan	0	0
(G-_G_G-_A)	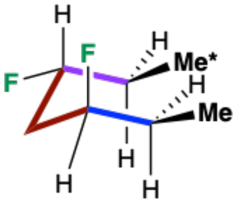 G^{-}GG^{-}A (identical as AG^{-}GG^{-})	nan	nan	nan	0	0

(G-_G_G-_G-)	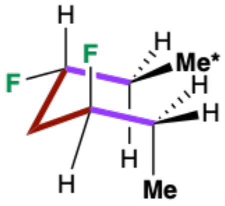 $G^-GG^-G^-$ (identical as $G^-G^-GG^-$)	nan	nan	nan	0	0
(G-_G_G-_G)	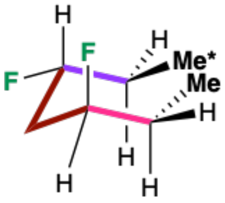 G^-GG^-G (identical as GG^-GG^-)	nan	nan	nan	0	0
(G-_G-_A_A)	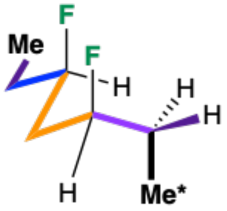 G^-G^-AA (identical as AAG^-G^-)	-474.7939	-1246571.3	12.61	0.01	0.16
(G-_G-_A_G-)	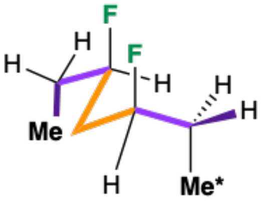 $G^-G^-AG^-$ (identical as $G^-AG^-G^-$)	-474.7928	-1246568.4	15.55	0	0.05
(G-_G-_A_G)	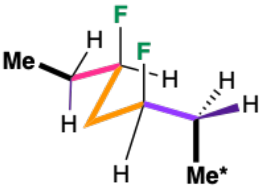 G^-G^-AG (identical as GAG^-G^-)	-474.7927	-1246568.1	15.8	0	0.04

(G-_G-_G_A)	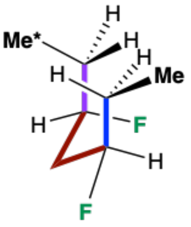<br>$G^{-}G^{-}GA$<br>(identical as $AGG^{-}G^{-}$)	nan	nan	nan	0	0
(G-_G-_G_G-)	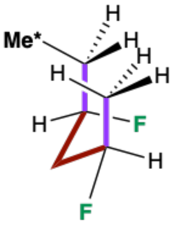<br>$G^{-}G^{-}GG^{-}$<br>(identical as $G^{-}GG^{-}G^{-}$)	nan	nan	nan	0	0
(G-_G-_G_G)	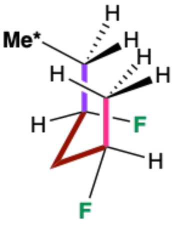<br>$G^{-}G^{-}GG$<br>(identical as $GGG^{-}G^{-}$)	-474.7894	-1246559.5	24.49	0	0
(G-_G-_G-_A)	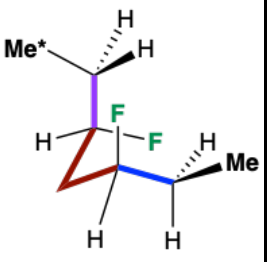<br>$G^{-}G^{-}G^{-}A$	-474.7925	-1246567.7	16.22	0	0.04
(G-_G-_G-_G-)	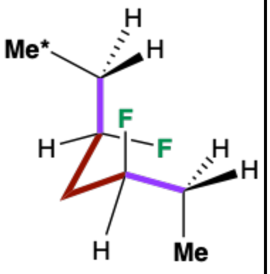<br>$G^{-}G^{-}G^{-}G^{-}$	-474.791	-1246563.9	20.09	0	0.01

(G-_G-_G-_G)	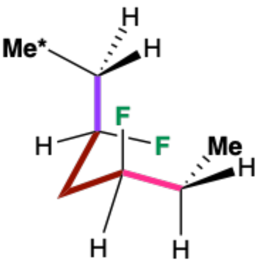 $G^{-}G^{-}G^{-}G$	-474.7872	-1246553.8	30.13	0	0
