# Supplementary material for: Conformational Analysis of 1,3-Difluorinated Alkanes
Source: J Org Chem. 2024 May 31;89(12):8789–803. doi: 10.1021/acs.joc.4c00670 (PMC11197103; doi:10.1021/acs.joc.4c00670)
Supplement: Supplementary file 2 — jo4c00670_si_004.zip [file jo4c00670_si_004.zip › SI/raw_data/difluoroheptane/syn-heptane-raw-vacuum.pdf]

| Conformer | | Energy (Hart | Energy (kJ/n | Relative Ene | Population | Population % |
|---|---|---|---|---|---|---|
| (G-_G-_G-_G-) | 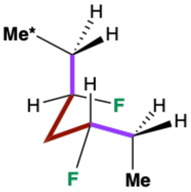<br>$G^{-}G^{-}G^{-}G^{-}$<br>(enantiomeric with<br>GGGG) | -474.7793 | -1246533 | 9.33 | 0.02 | 0.22 |
| (G-_G-_G-_G) | 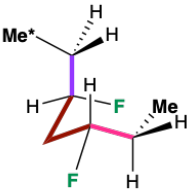<br>$G^{-}G^{-}G^{-}G$<br>(enantiomeric with<br>$G^{-}GGG$) | nan | nan | nan | 0 | 0 |
| (G-_G-_G-_A) | 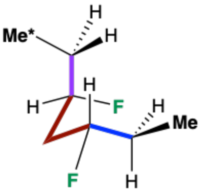<br>$G^{-}G^{-}G^{-}A$<br>(enantiomeric with<br>AGGG) | -474.78 | -1246534.8 | 7.5 | 0.05 | 0.46 |
| (G-_G-_G_G-) | 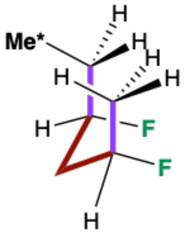<br>$G^{-}G^{-}GG^{-}$ | nan | nan | nan | 0 | 0 |
| (G-_G-_G_G) | 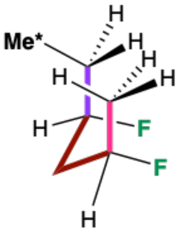<br>$G^{-}G^{-}GG$ | -474.7707 | -1246510.6 | 31.74 | 0 | 0 |
| (G-_G-_G_A) | 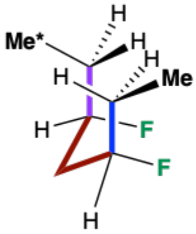<br>$G^{-}G^{-}GA$ | -474.7723 | -1246514.6 | 27.72 | 0 | 0 |

| | | | | | | |
|---|---|---|---|---|---|---|
| (G-_G-_A_G-) | 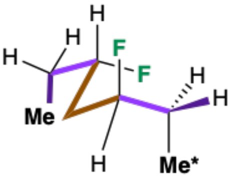 $G^{-}G^{-}AG^{-}$ (enantiomeric with GAGG) | -474.7814 | -1246538.5 | 3.8 | 0.22 | 2.03 |
| (G-_G-_A_G) | 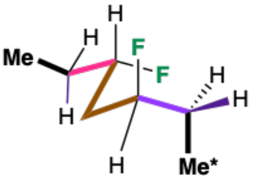 $G^{-}G^{-}AG$ (enantiomeric with $G^{-}AGG$) | -474.7817 | -1246539.5 | 2.87 | 0.31 | 2.95 |
| (G-_G-_A_A) | 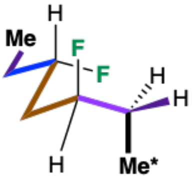 $G^{-}G^{-}AA$ (enantiomeric with AAGG) | -474.7826 | -1246541.6 | 0.72 | 0.75 | 7.03 |
| (G-_G_G-_G-) | 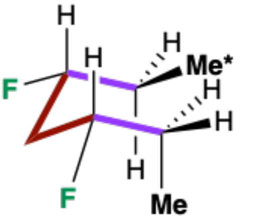 $G^{-}GG^{-}G^{-}$ | nan | nan | nan | 0 | 0 |
| (G-_G_G-_G) | 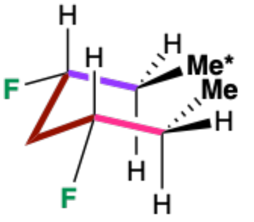 $G^{-}GG^{-}G$ | nan | nan | nan | 0 | 0 |
| (G-_G_G-_A) | 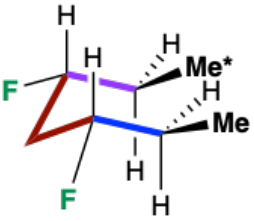 $G^{-}GG^{-}A$ | nan | nan | nan | 0 | 0 |

| | | | | | | |
|---|---|---|---|---|---|---|
| (G-_G_G_G-) | 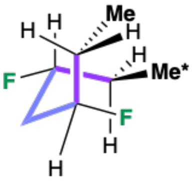 $G^-GGG^-$ (enantiomeric with $GG^-G^-G$) | nan | nan | nan | 0 | 0 |
| (G-_G_G_G) | 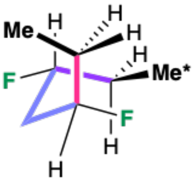 $G^-GGG$ (enantiomeric with $G^-G^-G^-G$) | nan | nan | nan | 0 | 0 |
| (G-_G_G_A) | 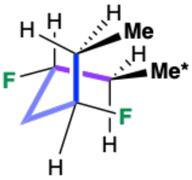 $G^-GGA$ (enantiomeric with $AG^-G^-G$) | nan | nan | nan | 0 | 0 |
| (G-_G_A_G-) | 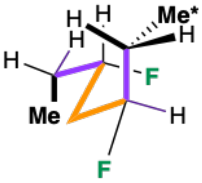 $G^-GAG^-$ (enantiomeric with $GAG^-G$) | nan | nan | nan | 0 | 0 |
| (G-_G_A_G) | 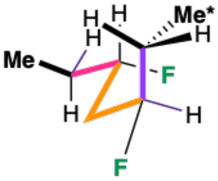 $G^-GAG$ (enantiomeric with $G^-AG^-G$) | nan | nan | nan | 0 | 0 |
| (G-_G_A_A) | 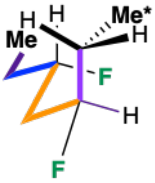 $G^-GAA$ (enantiomeric with $AAG^-G$) | nan | nan | nan | 0 | 0 |

| | | | | | | |
|---|---|---|---|---|---|---|
| (G-_A_G-_G-) | 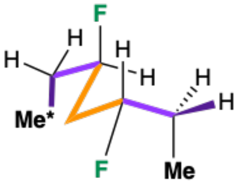 $G^-AG^-G^-$ (enantiomeric with GGAG) | -474.7807 | -1246536.8 | 5.51 | 0.11 | 1.02 |
| (G-_A_G-_G) | 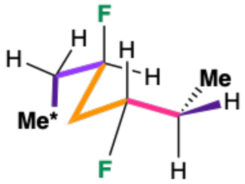 $G^-AG^-G$ (enantiomeric with $G^-GAG$) | nan | nan | nan | 0 | 0 |
| (G-_A_G-_A) | 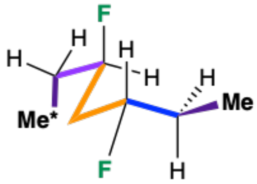 $G^-AG^-A$ (enantiomeric with AGAG) | -474.7818 | -1246539.7 | 2.68 | 0.34 | 3.19 |
| (G-_A_G_G-) | 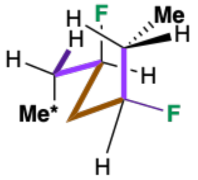 $G^-AGG^-$ (enantiomeric with $GG^-AG$) | -474.7776 | -1246528.5 | 13.85 | 0 | 0.04 |
| (G-_A_G_G) | 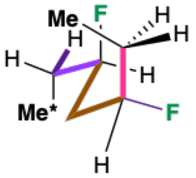 $G^-AGG$ (enantiomeric with $G^-G^-AG$) | -474.7817 | -1246539.5 | 2.87 | 0.31 | 2.95 |
| (G-_A_G_A) | 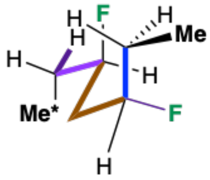 $G^-AGA$ (enantiomeric with $AG^-AG$) | -474.7821 | -1246540.3 | 2.02 | 0.44 | 4.16 |

| | | | | | | |
|---|---|---|---|---|---|---|
| (G-_A_A_G-) | 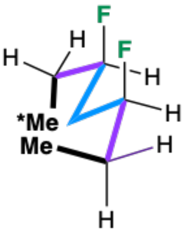<br>$G^{-}AAG^{-}$ | -474.7775 | -1246528.4 | 13.98 | 0 | 0.03 |
| (G-_A_A_G) | 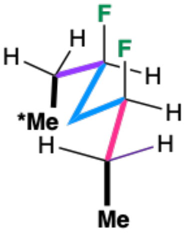<br>$G^{-}AAG$ | -474.7784 | -1246530.6 | 11.77 | 0.01 | 0.08 |
| (G-_A_A_A) | 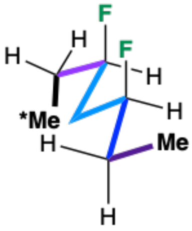<br>$G^{-}AAA$ | -474.7782 | -1246530.3 | 12.08 | 0.01 | 0.07 |
| (G_G-_G-_G-) | 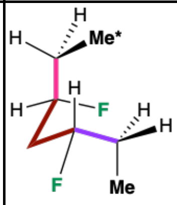<br>$GG^{-}G^{-}G^{-}$ (enantiomeric with $GGGG^{-}$) | -474.7745 | -1246520.5 | 21.82 | 0 | 0 |
| (G_G-_G-_G) | 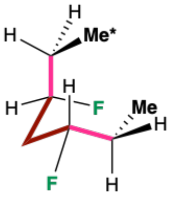<br>$GG^{-}G^{-}G$ (enantiomeric with $G^{-}GGG^{-}$) | nan | nan | nan | 0 | 0 |
| (G_G-_G-_A) | 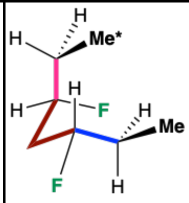<br>$GG^{-}G^{-}A$ (enantiomeric with $AGGG^{-}$) | -474.776 | -1246524.5 | 17.85 | 0 | 0.01 |

| | | | | | | |
|---|---|---|---|---|---|---|
| (G_G-_G_G-) | 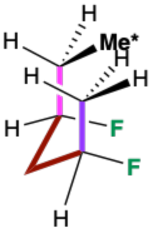 $GG^{-}GG^{-}$ | nan | nan | nan | 0 | 0 |
| (G_G-_G_G) | 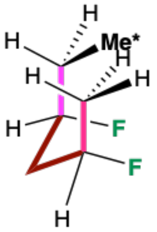 $GG^{-}GG$ | nan | nan | nan | 0 | 0 |
| (G_G-_G_A) | 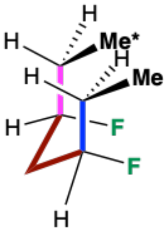 $GG^{-}GA$ | nan | nan | nan | 0 | 0 |
| (G_G-_A_G-) | 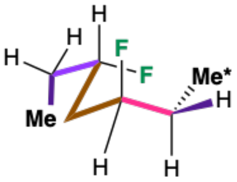 $GG^{-}AG^{-}$ (enantiomeric with $GAGG^{-}$) | -474.7769 | -1246526.7 | 15.67 | 0 | 0.02 |
| (G_G-_A_G) | 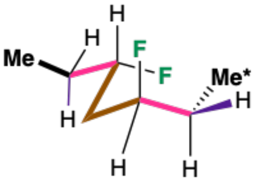 $GG^{-}AG$ (enantiomeric with $G^{-}AGG^{-}$) | -474.7776 | -1246528.5 | 13.85 | 0 | 0.04 |
| (G_G-_A_A) | 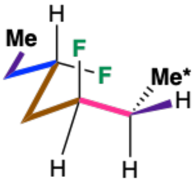 $GG^{-}AA$ (enantiomeric with $AAGG^{-}$) | -474.7774 | -1246528 | 14.39 | 0 | 0.03 |

| | | | | | | |
|---|---|---|---|---|---|---|
| (G_G_G-_G-) | 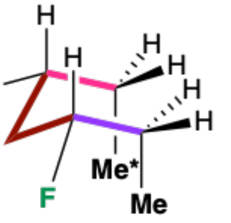 GGG-G- | nan | nan | nan | 0 | 0 |
| (G_G_G-_G) | 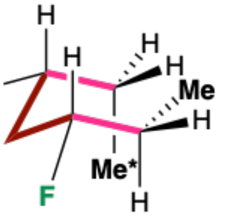 GGG-G | nan | nan | nan | 0 | 0 |
| (G_G_G-_A) | 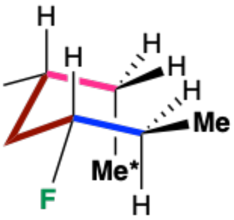 GGG-A | nan | nan | nan | 0 | 0 |
| (G_G_G_G-) | 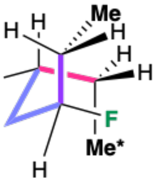 GGGG- (enantiomeric with GG-G-G-) | -474.7745 | -1246520.5 | 21.82 | 0 | 0 |
| (G_G_G_G) | 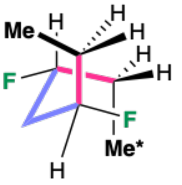 GGGG (enantiomeric with G-G-G-G-) | -474.7793 | -1246533 | 9.33 | 0.02 | 0.22 |
| (G_G_G_A) | 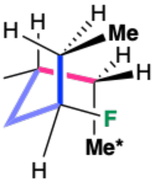 GGGA (enantiomeric with AG-G-G-) | -474.7794 | -1246533.3 | 9.04 | 0.03 | 0.24 |

| | | | | | | |
|---|---|---|---|---|---|---|
| (G_G_A_G-) | 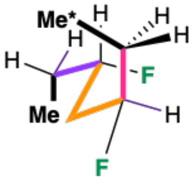<br>GGAG$^{-}$<br>(enantiomeric with<br>GAG$^{-}$G$^{-}$) | -474.7806 | -1246536.6 | 5.76 | 0.1 | 0.92 |
| (G_G_A_G) | 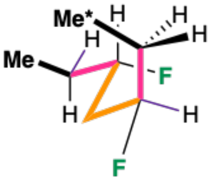<br>GGAG<br>(enantiomeric with<br>G$^{-}$AG$^{-}$G$^{-}$) | -474.7807 | -1246536.8 | 5.51 | 0.11 | 1.02 |
| (G_G_A_A) | 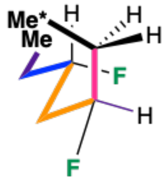<br>GGAA<br>(enantiomeric with<br>AAG$^{-}$G$^{-}$) | -474.7821 | -1246540.3 | 2 | 0.45 | 4.19 |
| (G_A_G-_G-) | 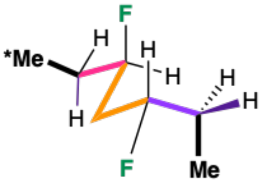<br>GAG$^{-}$G$^{-}$<br>(enantiomeric with<br>GGAG$^{-}$) | -474.7806 | -1246536.6 | 5.76 | 0.1 | 0.92 |
| (G_A_G-_G) | 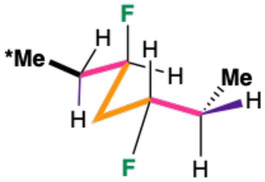<br>GAG$^{-}$G<br>(enantiomeric with<br>G$^{-}$GAG$^{-}$) | nan | nan | nan | 0 | 0 |
| (G_A_G-_A) | 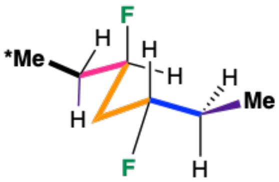<br>GAG$^{-}$A<br>(enantiomeric with<br>AGAG$^{-}$) | -474.7815 | -1246538.8 | 3.55 | 0.24 | 2.24 |

| | | | | | | |
|---|---|---|---|---|---|---|
| (G_A_G_G-) | 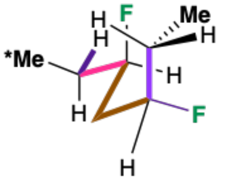<br>GA$G^{-}$G$^{-}$ → GAG$G^{-}$<br>(enantiomeric with<br>G$G^{-}$A$G^{-}$) | -474.7769 | -1246526.7 | 15.67 | 0 | 0.02 |
| (G_A_G_G) | 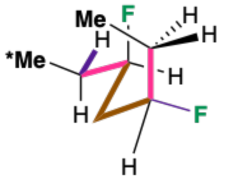<br>GAGG<br>(enantiomeric with<br>$G^{-}$$G^{-}$A$G^{-}$) | -474.7814 | -1246538.5 | 3.8 | 0.22 | 2.03 |
| (G_A_G_A) | 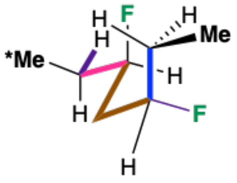<br>GAGA<br>(enantiomeric with<br>A$G^{-}$A$G^{-}$) | -474.7818 | -1246539.7 | 2.68 | 0.34 | 3.19 |
| (G_A_A_G-) | 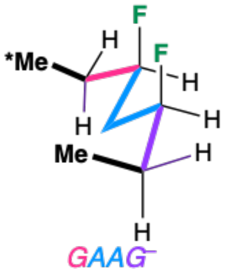<br>GAA$G^{-}$ | -474.7777 | -1246528.9 | 13.49 | 0 | 0.04 |
| (G_A_A_G) | 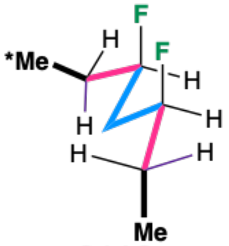<br>GAAG | -474.7775 | -1246528.4 | 13.98 | 0 | 0.03 |
| (G_A_A_A) | 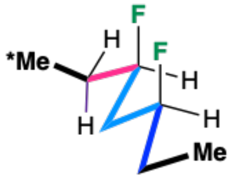<br>GAAA | -474.7786 | -1246531.2 | 11.14 | 0.01 | 0.1 |

| | | | | | | |
|---|---|---|---|---|---|---|
| (A_G-_G-_G-) | 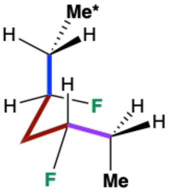 $AG^-G^-G^-$ (enantiomeric with GGGA) | -474.7794 | -1246533.3 | 9.04 | 0.03 | 0.24 |
| (A_G-_G-_G) | 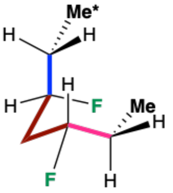 $AG^-G^-G$ (enantiomeric with $G^-GGA$) | nan | nan | nan | 0 | 0 |
| (A_G-_G-_A) | 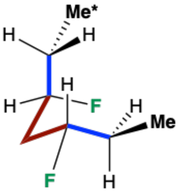 $AG^-G^-A$ (enantiomeric with AGGA) | -474.7804 | -1246536 | 6.34 | 0.08 | 0.73 |
| (A_G-_G_G-) | 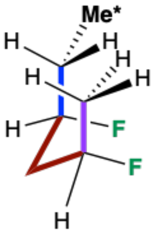 $AG^-GG^-$ | nan | nan | nan | 0 | 0 |
| (A_G-_G_G) | 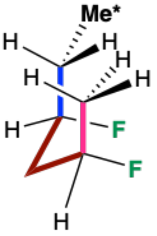 $AG^-GG$ | -474.7723 | -1246514.6 | 27.72 | 0 | 0 |
| (A_G-_G_A) | 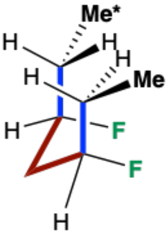 $AG^-GA$ | -474.7729 | -1246516.3 | 26.05 | 0 | 0 |

| | | | | | | |
|---|---|---|---|---|---|---|
| (A_G-_A_G-) | 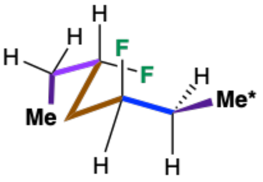<br>AG$^-$AG$^-$<br>(enantiomeric with<br>GAGA) | -474.7818 | -1246539.7 | 2.68 | 0.34 | 3.19 |
| (A_G-_A_G) | 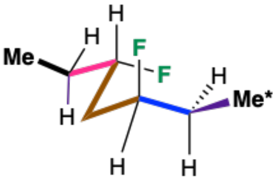<br>AG$^-$AG<br>(enantiomeric with<br>G$^-$AGA) | -474.7821 | -1246540.3 | 2.02 | 0.44 | 4.16 |
| (A_G-_A_A) | 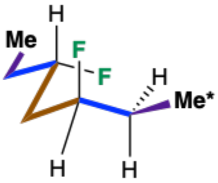<br>AG$^-$AA<br>(enantiomeric with<br>AAGA) | -474.7826 | -1246541.8 | 0.54 | 0.8 | 7.56 |
| (A_G_G-_G-) | 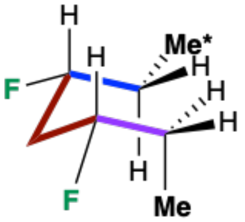<br>AGG$^-$G$^-$ | nan | nan | nan | 0 | 0 |
| (A_G_G-_G) | 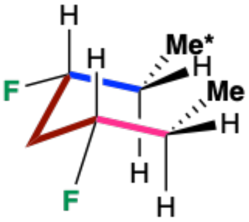<br>AGG$^-$G | nan | nan | nan | 0 | 0 |
| (A_G_G-_A) | 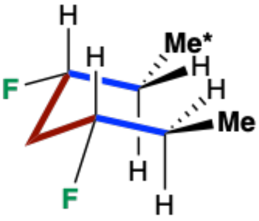<br>AGG$^-$A | nan | nan | nan | 0 | 0 |

| | | | | | | |
|---|---|---|---|---|---|---|
| (A_G_G_G-) | 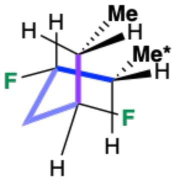 AGGG$^-$ (enantiomeric with GG$^-$G$^-$A) | -474.776 | -1246524.5 | 17.85 | 0 | 0.01 |
| (A_G_G_G) | 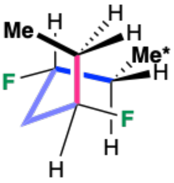 AGGG (enantiomeric with G$^-$G$^-$G$^-$A) | -474.78 | -1246534.8 | 7.5 | 0.05 | 0.46 |
| (A_G_G_A) | 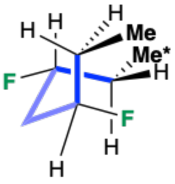 AGGA (enantiomeric with AG$^-$G$^-$A) | -474.7804 | -1246536 | 6.34 | 0.08 | 0.73 |
| (A_G_A_G-) | 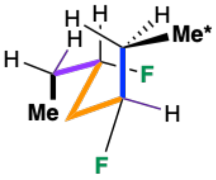 AGAG$^-$ (enantiomeric with GAG$^-$A) | -474.7815 | -1246538.8 | 3.55 | 0.24 | 2.24 |
| (A_G_A_G) | 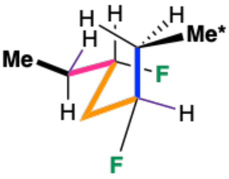 AGAG (enantiomeric with G$^-$AG$^-$A) | -474.7818 | -1246539.7 | 2.68 | 0.34 | 3.19 |
| (A_G_A_A) | 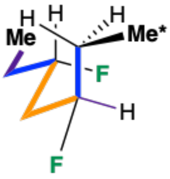 AGAA (enantiomeric with AAG$^-$A) | -474.7828 | -1246542.3 | 0 | 1 | 9.4 |

| | | | | | | |
|---|---|---|---|---|---|---|
| (A_A_G-_G-) | 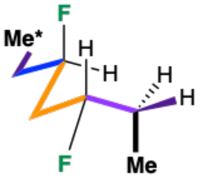 $AAG^{-}G^{-}$ (enantiomeric with $GGAA$) | -474.7821 | -1246540.3 | 2 | 0.45 | 4.19 |
| (A_A_G-_G) | 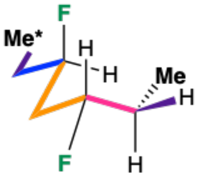 $AAG^{-}G$ (enantiomeric with $G^{-}GAA$) | nan | nan | nan | 0 | 0 |
| (A_A_G-_A) | 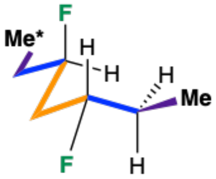 $AAG^{-}A$ (enantiomeric with $AGAA$) | -474.7828 | -1246542.3 | 0 | 1 | 9.4 |
| (A_A_G_G-) | 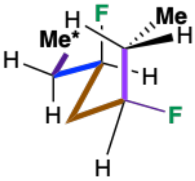 $AAGG^{-}$ (enantiomeric with $GG^{-}AA$) | -474.7774 | -1246528 | 14.39 | 0 | 0.03 |
| (A_A_G_G) | 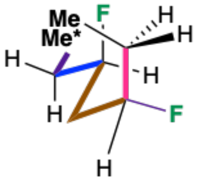 $AAGG$ (enantiomeric with $G^{-}G^{-}AA$) | -474.7826 | -1246541.6 | 0.72 | 0.75 | 7.03 |
| (A_A_G_A) | 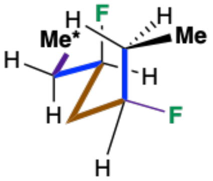 $AAGA$ (enantiomeric with $AG^{-}AA$) | -474.7826 | -1246541.8 | 0.54 | 0.8 | 7.56 |

| | | | | | | |
|---|---|---|---|---|---|---|
| (A_A_A_G-) | 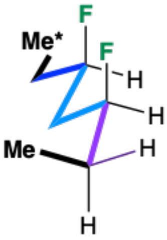 AAAG- | -474.7786 | -1246531.2 | 11.14 | 0.01 | 0.1 |
| (A_A_A_G) | 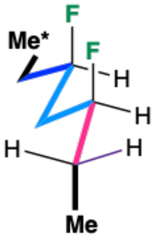 AAAG | -474.7782 | -1246530.3 | 12.08 | 0.01 | 0.07 |
| (A_A_A_A) | 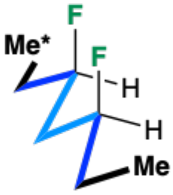 AAAA | -474.7793 | -1246533.1 | 9.28 | 0.02 | 0.22 |
