# Supplementary material for: Conformational Analysis of 1,3-Difluorinated Alkanes
Source: J Org Chem. 2024 May 31;89(12):8789–803. doi: 10.1021/acs.joc.4c00670 (PMC11197103; doi:10.1021/acs.joc.4c00670)
Supplement: Supplementary file 2 — jo4c00670_si_004.zip [file jo4c00670_si_004.zip › SI/raw_data/difluoropropane/difluoro-propane-raw-vacuum.pdf]

Conformer		Energy (Hart	Energy (kJ/n	Relative Ene	Population	Population %
(G-_G)	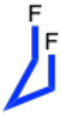 gg(u)	-317.6024	-833865.15	11.81	0.01	0.3
(G-_A)	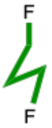 ga	-317.6054	-833872.89	4.07	0.19	6.88
(G-_G-)	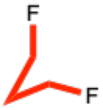 g^-g^-	-317.6069	-833876.96	0	1	35.58
(G_G-)	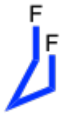 gg(u)	-317.6024	-833865.15	11.81	0.01	0.3
(G_A)	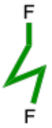 ga	-317.6054	-833872.89	4.07	0.19	6.88
(G_G)	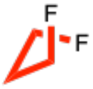 gg	-317.6069	-833876.96	0	1	35.58
(A_G)	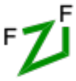 ag	-317.6054	-833872.89	4.07	0.19	6.88
(A_A)	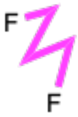 aa	-317.6032	-833867.19	9.77	0.02	0.69
(A_G-)	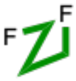 ag	-317.6054	-833872.89	4.07	0.19	6.88
