# Supplementary material for: Conformational Analysis of 1,3-Difluorinated Alkanes
Source: J Org Chem. 2024 May 31;89(12):8789–803. doi: 10.1021/acs.joc.4c00670 (PMC11197103; doi:10.1021/acs.joc.4c00670)
Supplement: Supplementary file 2 — jo4c00670_si_004.zip [file jo4c00670_si_004.zip › SI/raw_data/difluoropropane/difluoro-propane-raw-water.pdf]

Conformer		Energy (Hart	Energy (kJ/n	Relative Ene	Population	Population %
(G-_G)	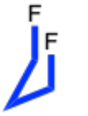 gg(u)	-317.6128	-833892.41	2.92	0.31	7.8
(G-_A)	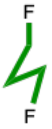 ga	-317.6128	-833892.47	2.86	0.32	7.99
(G-_G-)	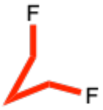 $g^{-}g^{-}$	-317.6139	-833895.33	0	1	25.34
(G_G-)	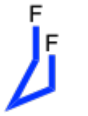 gg(u)	-317.6128	-833892.41	2.92	0.31	7.8
(G_A)	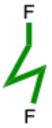 ga	-317.6128	-833892.47	2.86	0.32	7.99
(G_G)	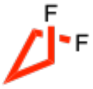 gg	-317.6139	-833895.33	0	1	25.34
(A_G)	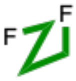 ag	-317.6128	-833892.47	2.86	0.32	7.99
(A_A)	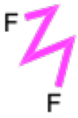 aa	-317.6114	-833888.74	6.59	0.07	1.77
(A_G-)	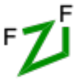 ag	-317.6128	-833892.47	2.86	0.32	7.99
