# Supplementary material for: Conformational Analysis of 1,3-Difluorinated Alkanes
Source: J Org Chem. 2024 May 31;89(12):8789–803. doi: 10.1021/acs.joc.4c00670 (PMC11197103; doi:10.1021/acs.joc.4c00670)
Supplement: Supplementary file 2 — jo4c00670_si_004.zip [file jo4c00670_si_004.zip › SI/raw_data/difluoropropane/difluoro-propane-raw-chloroform.pdf]

Conformer		Energy (Hart	Energy (kJ/n	Relative Ene	Population	Population %
(G-_G)	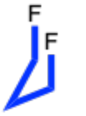 gg(u)	-317.6153	-833898.86	5.47	0.11	3.37
(G-_A)	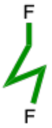 ga	-317.616	-833900.82	3.51	0.24	7.43
(G-_G-)	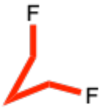 g^-g^-	-317.6173	-833904.33	0	1	30.65
(G_G-)	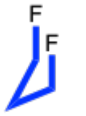 gg(u)	-317.6153	-833898.86	5.47	0.11	3.37
(G_A)	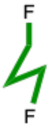 ga	-317.616	-833900.82	3.51	0.24	7.43
(G_G)	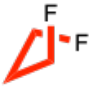 gg	-317.6173	-833904.33	0	1	30.65
(A_G)	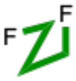 ag	-317.616	-833900.82	3.51	0.24	7.43
(A_A)	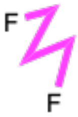 aa	-317.6149	-833897.85	6.48	0.07	2.24
(A_G-)	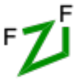 ag	-317.616	-833900.82	3.51	0.24	7.43
